# A Novel Drug Combination of Mangiferin and Cinnamic Acid Alleviates Rheumatoid Arthritis by Inhibiting TLR4/NFκB/NLRP3 Activation-Induced Pyroptosis

**DOI:** 10.3389/fimmu.2022.912933

**Published:** 2022-06-21

**Authors:** Weijie Li, Kexin Wang, Yudong Liu, Hao Wu, Yan He, Congchong Li, Qian Wang, Xiaohui Su, Shikai Yan, Weiwei Su, Yanqiong Zhang, Na Lin

**Affiliations:** ^1^ Institute of Chinese Materia Medica, China Academy of Chinese Medical Sciences, Beijing, China; ^2^ Guangdong Engineering and Technology Research Center for Quality and Efficacy Reevaluation of Post-Market Traditional Chinese Medicine, Guangdong Provincial Key Laboratory of Plant Resources, School of Life Sciences, Sun Yat-sen University, Guangzhou, China; ^3^ State Key Laboratory of Natural and Biomimetic Drugs, School of Pharmaceutical Sciences, Peking University, Beijing, China; ^4^ School of Pharmacy, Shanghai Jiao Tong University, Shanghai, China

**Keywords:** rheumatoid arthritis (RA), traditional Chinese medicine-originated disease-modifying anti-rheumatic prescription, pyroptosis, bioactive compound, NLRP3 inflammasome

## Abstract

Growing evidence shows that Baihu-Guizhi decoction (BHGZD), a traditional Chinese medicine (TCM)-originated disease-modifying anti-rheumatic prescription, may exert a satisfying clinical efficacy for rheumatoid arthritis (RA) therapy. In our previous studies, we verified its immunomodulatory and anti-inflammatory activities. However, bioactive compounds (BACs) of BHGZD and the underlying mechanisms remain unclear. Herein, an integrative research strategy combining UFLC-Q-TOF-MS/MS, gene expression profiling, network calculation, pharmacokinetic profiling, surface plasmon resonance, microscale thermophoresis, and pharmacological experiments was carried out to identify the putative targets of BHGZD and underlying BACs. After that, both *in vitro* and *in vivo* experiments were performed to determine the drug effects and pharmacological mechanisms. As a result, the calculation and functional modularization based on the interaction network of the “RA-related gene–BHGZD effective gene” screened the TLR4/PI3K/AKT/NFκB/NLRP3 signaling-mediated pyroptosis to be one of the candidate effective targets of BHGZD for reversing the imbalance network of “immune-inflammation” during RA progression. In addition, both mangiferin (MG) and cinnamic acid (CA) were identified as representative BACs acting on that target, for the strong binding affinities between compounds and target proteins, good pharmacokinetic features, and similar pharmacological effects to BHGZD. Notably, both BHGZD and the two-BAC combination of MG and CA effectively alleviated the disease severity of the adjuvant-induced arthritis-modified rat model, including elevating pain thresholds, relieving joint inflammation and bone erosion *via* inhibiting NF-κB *via* TLR4/PI3K/AKT signaling to suppress the activation of the NLRP3 inflammasome, leading to the downregulation of downstream caspase-1, the reduced release of IL-1β and IL-18, and the modulation of GSDMD-mediated pyroptosis. Consistent data were obtained based on the *in vitro* pyroptosis cellular models of RAW264.7 and MH7A cells induced by LPS/ATP. In conclusion, these findings offer an evidence that the MG and CA combination identified from BHGZD may interact with TLR4/PI3K/AKT/NFκB signaling to inhibit NLRP3 inflammasome activation and modulate pyroptosis, which provides the novel representative BACs and pharmacological mechanisms of BHGZD against active RA. Our data may shed new light on the mechanisms of the TCM formulas and promote the modernization development of TCM and drug discovery.

## Introduction

Rheumatoid arthritis (RA), characterized by hyperplasia and inflammation of the synovial joints, is an intractable and highly prevalent autoimmune disease with unknown pathogenic triggers (1, 2). Currently, the key therapeutic approaches to RA are mainly based on drugs and surgeries (3), including disease-modifying anti-rheumatic drugs (DMARDs), non-steroidal anti-inflammatory drugs (NSAIDs), glucocorticoids, and biological response modifiers, which may reduce synovitis and systemic inflammation (4). However, there are no specific drugs for RA therapy due to its various comorbidities, such as cardiovascular diseases (5–8), osteoporosis (9, 10), interstitial lung disease (9, 11), malignancies (11, 12), and hypertension (13), as well as the heterogeneous and complex clinical manifestations with wide individual differences. Traditional Chinese medicine (TCM), as a complementary and alternative medicine, has its unique advantages in the clinical treatment of RA, especially at the active stage with moist heat arthralgia spasm syndrome (active RA), which is characterized by the hyperactive immune response and excessive inflammatory cytokines, subsequently leading to osteoclast differentiation and active joint inflammation (14, 15). Among TCM-originated DMARDs, Baihu-Guizhi decoction (BHGZD) has been indicated to efficiently reverse the aggressive progression of active RA with moist heat arthralgia spasm syndrome and to reach a remarkable response rate (90%) (16–19). Our previous studies identified the chemical profiling of BHGZD *in vitro* and verified its immunomodulatory and anti-inflammatory activities (20–22). However, the bioactive compounds (BACs) and the underlying mechanisms of BHGZD against active RA have not been fully elucidated.

In the current study, the candidate BACs of BHGZD were identified by the ultra-fast liquid chromatography-quadrupole-time-of-flight tandem mass spectrometry (UFLC-Q-TOF-MS/MS) system *in vivo*. Then, we carried out transcriptomic profiling, target prediction, and network calculation to screen the candidate targets of BHGZD for reversing the imbalance network of “immune-inflammation” during the progression of active RA. After that, the binding affinities between candidate BACs and candidate targets of BHGZD were calculated using the molecular docking simulation, the results of which were subsequently verified by surface plasmon resonance (SPR) assay and microscale thermophoresis (MST) analysis. Moreover, the *in vivo* biodistribution and pharmacokinetic characteristics of candidate BACs were also determined. Furthermore, the pharmacological effects and the molecular mechanisms of BHGZD, and its representative BACs against active RA were further validated by a series of experiments based on the adjuvant-induced arthritis-modified rat model (AIA-M) as well as LPS/ATP-induced pyroptosis cellular models on both RAW264.7 macrophage and MH7A cells.

## Materials and Methods

### Preparation of BHGZD

Crude drugs of BHGZD formula were purchased from Beijing Tongrentang Co., Ltd. (Beijing, China). BHGZD was prepared according to the original composition of the formula recorded in Chinese pharmacopoeia 2020 edition and our previous study (22) (**Supplementary Materials Section 1**, and **Table S1**).

### RA Modeling, Grouping, and Treatment

A total of 49 male Lewis rats (200 ± 20 g in weight, 6–8 weeks old) were obtained from Beijing Vital River Laboratory Animal Technology Co., Ltd. (SCXK 2019-0003, Beijing, China) and randomly divided into two independent clusters: a discovery cluster (*n* = 24) and a validation cluster (*n* = 25). All animals were maintained in specific pathogen-free conditions with a constant temperature of 24 ± 1°C with a 12-h light/dark cycle, and had free access to standard rodent diet and water. The discovery and validation clusters were respectively used for gene expression profiling and experimental validation. In the discovery cluster, 24 rats were randomly divided into three groups [3 per group for microarray and 5 per group for mRNA sequencing (mRNA-Seq)] (1): the normal control group, (2) the AIA-M model group, and (3) the BHGZD treatment group. In the validation cluster, 25 rats were randomly divided into five groups (5 per group): (1) the normal control group, (2) the AIA-M model group, (3) the BHGZD treatment group, (4) the MG+CA treatment group, and (5) the methotrexate (MTX) treatment group.

The AIA-M rat model was established simulating the pathological changes and characteristics of active RA with moist heat arthralgia spasm syndrome based on our previous studies (20–23). Please see the detailed information on the AIA-M rat model establishment in **Supplementary Materials Section 2**. The dosage of BHGZD for the corresponding treatment group was 21.4 g/kg, equivalent to two times the clinical dose, which has been proved to exert the most prominent therapeutic efficacy in our previous studies (21, 22). The dosages for the two-BAC combination of MG and CA for the corresponding treatment group were respectively 600.912 mg/kg and 46.652 mg/kg, equivalent to their content in 21.4 g/kg BHGZD. The dosage for MTX was 0.2 mg/kg. All treatments were performed for 30 days *via* oral administration from the day of the primary immunization (20–23).

On the 31st day, all rats were anesthetized by intraperitoneal injection of pentobarbital sodium (50 mg/kg). The blood samples were collected using one-time anticoagulant negative pressure blood collection tubes, and then placed at room temperature for 20 min and centrifuged at 3,000 rpm for 15 min. The sera were collected, vortexed, and centrifuged again at high speed at low temperature (4°C) for 15 min, and stored at −20°C for subsequent assays. Whole blood cells were freshly isolated from blood, then frozen in liquid nitrogen overnight, and stored at −80°C for mRNA-Seq. The synovium tissues were isolated and immediately frozen in liquid nitrogen for microarray analysis. The right joints of rats were removed and preserved in 4% paraformaldehyde (PFA) combined with phosphate-buffered saline (PBS) for 72 h for subsequent assays. The left joints and organs of rats were kept at −80°C for subsequent assays.

### Assessment of Arthritis Severity

The severity of arthritis was evaluated by arthritis incidence, hind paw thickness (the diameter of limb), arthritis score, and arthritis surface temperature as in our previous studies (20–24), and is provided in **Supplementary Materials Section 3**. Pain thresholds were evaluated by mechanical-, acetone-, and thermal-induced hyperalgesia as previously described (20–25), and are provided in **Supplementary Materials Section 4**.

### Viscera Index and Histology

The viscera indexes were calculated by the weight of the thymus, spleen, liver, and kidney relative to total brain weight. Histological changes were examined using hematoxylin and eosin (H&E), safranin O-fast green (Solarbio, Beijing, China), and Masson trichrome staining (Baso, Wuhan, China) according to routine protocols. Please see detailed information on the protocol in **Supplementary Materials Section 5**.

### Micro-Computed Tomography Analysis

To quantitatively evaluate bone formation within the defects, the specimens were scanned using a micro-CT instrument (GE healthcare, USA) at a resolution of 45 μm. The x-ray settings and cylinder region of interest (ROI) are provided in **Supplementary Materials Section 6**.

### Gene Expression Profiling

Whole blood cells and the synovium tissues representing the pathological characteristics of systemic disease and target organs during active RA progression were respectively used for whole rat genome microarray analysis (Agilent Technologies, design ID: 014879, Santa Clara, CA, USA) and mRNA-Seq Illumina NovaSeq 6000 (Illumina, CA, USA) to screen RA-related genes [the significant differentially expressed genes (DEGs) between the AIA-M model group and the normal control group] and BHGZD therapeutic effect-related genes (DEGs between the BHGZD treatment group and the AIA-M model group). The gene expression data have been uploaded and are publicly available in the NCBI GEO database (GEO No. GSE189942, https://www.ncbi.nlm.nih.gov/geo/query/acc.cgi?acc=GSE189942, December 3, 2021, and GEO No. GSE190523, https://www.ncbi.nlm.nih.gov/geo/query/acc.cgi?acc=GSE190523, December 11, 2021), and the DEGs were identified by referring to the criteria of *t*-test *p*-value < 0.05 and fold change (FC)>2/<0.5. Please see the detailed information in **Supplementary Materials Section 7**.

### Prediction of Putative BHGZD Targets

Drugs with a high similarity score (>0.80) to structures of composite compounds of each ingredient contained in BHGZD were selected based on the TCMIP v2.0 database (Integrative Pharmacology-based Research Platform of Traditional Chinese Medicine, last update in 2021-09-10, http://www.tcmip.cn/TCMIP/index.php/Home) and the Encyclopedia of Traditional Chinese Medicine (ETCM, http://www.tcmip.cn/ETCM/index.php/Home/) (26). Therapeutic targets of the similar drugs approved by the Food and Drug Administration (FDA) according to DrugBank Online (Version 5.1.8, released 2021-01-03, https://go.drugbank.com/) were identified as putative targets of BHGZD.

### Network Construction and Analysis

The RA-related gene interaction network was constructed using the links among the DEGs between the AIA-M model and normal control groups to identify the key RA-related genes. Then, the interaction network of the “key RA-related gene–BHGZD-effective gene” was constructed using the links between the key RA-related genes and BHGZD effective genes. The gene–gene interaction data were collected from the String database (version 10.0, http://string-db.org/) (27), and the interaction networks were all visualized by the Cytoscape platform (version 3.9.0, https://cytoscape.org/) (28). The network topological properties of nodes, including “Degree”, “Betweenness”, and “Closeness”, were calculated for screening the key network targets according to our previous descriptions (20–25, 29) and **Supplementary Materials Section 8**.

### Determining the Chemical Compounds of BHGZD and Absorption Distribution Metabolism Excretion Evaluation

A rapid, sensitive, and reliable method by the UFLC-Q-TOF-MS/MS system was used to identify the chemical profiling of BHGZD as described in **Supplementary Materials Section 9** (30). The reference standards of these compounds (**Supplementary Table S2**) were purchased from Chengdu Must Bio-technology Co., Ltd. (Chengdu, China), the National Institute for the Control of Pharmaceutical and Biological Products (Beijing, China), and Chengdu DeSiTe Biological Technology Co., Ltd. (Chengdu, China). Then, the ADME information on 19 BACs included in BHGZD was collected from the ETCM database (The Encyclopedia of Traditional Chinese Medicine, http://www.tcmip.cn/ETCM/) (26). In addition, *in silico* ADME models were used to identify the candidate BACs of BHGZD by calculating the passive intestinal permeability of the Caco-2 module, the oral bioavailability, and the apparent permeability coefficient (Papp) of each BACs as described in **Supplementary Materials Section 10**.

### Molecular Docking Simulation

The molecular docking and virtual screening program was carried out to investigate the direct binding efficiencies of the two BACs contained in BHGZD and the corresponding putative targets. The structures of human TLR4 (PDB ID: 2z65), AKT1 (PDB ID: 3o96), and NFκB (PDB ID: 1nfi) were obtained from the PDB website (The Protein Data Bank, https://www.rcsb.org/). For docking analysis, the ligand mangiferin (MG) and cinnamic acid (CA) were downloaded from the ZINC database (last update in 2018-02-14, http://zinc.docking.org/) (31) in mol2 format and converted to pdb.files using OpenBabel GUI (last update in 2016-09-21, version 2.4.1, http://openbabel.org/wiki/Main_Page). AutoDock Tools (Version 1.5.6, https://ccsb.scripps.edu/mgltools/) was used to convert pdb to pdbqt format. Docking calculations were performed using AutoDock Vina (The Scripps Research Institute, version 1.1.2) and AutoDock (The Scripps Research Institute, version 4.2.6). The visualization and analysis of the results were obtained from AutoDock Vina by PyMOL (Version 2.5, https://pymol.org/2/).

### SPR Assay

To confirm the binding affinity of recombinant AKT1 protein and MG, SPR assay was performed with Biacore 8K (Biacore, Cytiva), and the *K*_D_ value was calculated using the Biacore 8K evaluation software 2.0 (GE Healthcare). Detailed information on SPR assay is provided in **Supplementary Materials Section 11**.

### MST Assay

The binding affinity between recombinant TLR4 protein and CA was measured by a NanoTemper Monolith NT.115 instrument (NanoTemper Technologies, Germany). The *K*_D_ value was fitted by NanoTemper Monolith affinity software (NanoTemper Technologies, Germany) using 1:1 binding mode. Detailed information on the MST assay is provided in **Supplementary Materials Section 12**.

### *In Vivo* Pharmacokinetic Analysis

To detect the pharmacokinetic parameters of MG and CA *in vivo*, quantification analysis was performed by an ultra-high-performance liquid chromatography (Shimadzu Corp., Japan) tandem ion trap quadrupole QTRAP 6500 plus mass spectrometry (AB Sciex, USA). The quantitative analyses for MG and CA were approved by the Guidance for Bioanalytical Method Validation issued by the Chinese Pharmacopoeia Commission in 2020. The pharmacokinetic parameters were calculated by Drug and Statistic software (Shanghai, China). Detailed information on the collection of blood samples and UHPLC-QTRAP-MS/MS conditions is provided in **Supplementary Materials Section 13**.

### Cell Culture, Induction, and Treatment

Mouse macrophage cell line (RAW264.7 cells, Bio-Swamp, Wuhan, China) and fibroblast-like synoviocytes in patients with RA (MH7A cells, Riken cell bank, Ibaraki, Japan) at passage numbers four to eight were used in the current experiment validations. The culture conditions are provided in **Supplementary Materials Section 14**.

The conventional NLRP3 inflammasome activation cellular model was induced by lipopolysaccharide [LPS, *Escherichia coli* (O111:B4), Sigma-Aldrich, St Louis, MO, USA] and adenosine triphosphate (ATP disodium salt hydrate, Sigma-Aldrich, St Louis, MO, USA) as in our previous study (22) (**Supplementary Materials Section 15**).

For the drug treatment, both RAW264.7 and MH7A cells were divided into the following groups: (1) Normal control group: with no stimulation and treatment; (2) LPS/ATP-induced cellular model group: cells were stimulated with the corresponding concentrations of LPS and ATP; (3) BHGZD treatment group: after stimulation, cells were treated with 28.54 μg/ml BHGZD for 24 h, which was determined to be the most effective as in our previous study (22); (4) MG+CA treatment group: after stimulation, cells were treated with the two-BAC combination (1.69 ng/ml MG and 0.13 ng/ml CA, with the same content as that in the 5 μg/ml BHGZD formula) for 24 h; (5) MG treatment group: after stimulation, cells were treated with 1.69 ng/ml MG for 24 h; and (6) CA treatment group: after stimulation, cells were treated with 0.13 ng/ml CA for 24 h. The concentration of DMSO was less than 1‰ of the solution for the *in vitro* experiment.

### Cell Viability Assay

Cell viability was analyzed with the cell counting kit-8 (CCK-8) assay kit (Beyotime Biotechnology, Shanghai, China) as described in **Supplementary Materials Section 16**.

### Flow Cytometry

To identify and quantify the pyroptosis population of RAW264.7 cells, FITC Apoptosis Detection kit I staining with both the Annexin V-fluorescein isothiocyanate and propidium iodide (PI, BD Biosciences, San Jose, CA, USA) was used according to the manufacturer’s guidelines. Flow cytometry was performed using NovoCyte 2040R (ACEA Bioscience, San Diego, CA, USA), and analyzed using the NovoExpress 1.4.1 software (ACEA Bioscience, San Diego, CA, USA). Detailed information of this experiment is provided in **Supplementary Materials Section 17**.

### Terminal Deoxynucleotidyl Transferase-Mediated dUTP Biotin Nick End Labeling Staining

The occurrence of pyroptosis in both MH7A cells and synovium tissues in AIA-M rats was determined by *in situ* TUNEL staining (TUNEL Andy Fluor™ 594 Apoptosis Detection Kit, ABP Biosciences, Wuhan, China) in accordance with the manufacturer’s protocol. Fluorescence images were visualized and photographed with inverted fluorescence microscopes (MF53, MSHOT, Guangzhou, China; Olympus, BX51, Tokyo, Japan). Detailed information on TUNEL assay is provided in **Supplementary Materials Section 18**.

### FAM-FLICA Caspase-1 Assay

Active caspase-1 was visualized by a FAM-FLICA caspase-1 assay kit using a FAM-YVAD-FMK inhibitor probe (ImmunoChemistry Technologies, Bloomington, MN, USA), according to the manufacturer’s guidelines. Fluorescence images were visualized and photographed with a confocal microscope (Zeiss LSM 880, Carl Zeiss, Jena, Germany). Detailed information on FAM-FLICA capase-1 assay is provided in **Supplementary Materials Section 19**.

### Immunofluorescence Staining

Double fluorescence staining was performed as described previously (22) (**Supplementary Materials Section 20**). The images were visualized and photographed with a confocal microscope (Zeiss LSM 880, Carl Zeiss, Jena, Germany) or an inverted fluorescence microscope (MF53, MSHOT, Guangzhou, China).

### Immunohistochemical Staining

To detect the expression levels of NLPR3/ASC of the knee joints in AIA-M rats and corresponding treatment groups, immunohistochemical staining was carried out using a DAB kit (Cat No. AR1027, Boster Biological Technology Co., Ltd., Wuhan, China) and a rabbit/mouse two-step detection kit (Cat No. SV0002/SV0001, Boster Biological Technology Co., Ltd., Wuhan, China) according to the routine protocols. Immunohistochemistry quantification was performed using ImageJ (Image Progressing and Analysis in Java, version 1.42q, https://imagej.nih.gov/ij/), following the ImageJ User Guide.

### Western Blotting Analysis

To evaluate the regulation of BHGZD on the candidate targets in arthritic tissue samples, as well as RAW264.7 and MH7A cells, Western blotting analysis was carried out following the protocol as in our previous studies (21, 24, 29). TLR4, phospho-PI3K (p-PI3K), PI3K, p-AKT, p-NFκB-p65, NFκB-p65, NLRP3, ASC, caspase-1, the N-terminal domain of GSDMD (GSDMD-NT), and IL-1β antibodies were used as shown in **Supplementary Table S3**. GAPDH (GAPDH Mouse monoclonal antibody, Abcam, Cambridge, UK) and β-actin (Anti-beta-Actin/β-Actin Antibody, Boster Biological Technology, California, USA) were used as loading controls for arthritic tissue samples and cultured cells, respectively.

### Cytokine Analysis and Lactate Dehydrogenase Measurement

To evaluate the therapeutic effects of two BACs from BHGZD on the regulation of the “immune-inflammation” system, the levels of cytokines, proteins, and LDH release in RAW264.7 and MH7A cellular supernatant, as well as the sera of AIA-M rats were determined using enzyme-linked immunosorbent assay (ELISA) to assess the integrity of membranes in accordance with the manufacturer’s guidelines.

### Statistical Analyses

Statistical analyses were performed using GraphPad Prism 8.0 Software (San Diego, CA, USA). Data are expressed as the mean ± SD and analyzed by one-way ANOVA with Bonferroni’s or Dunnett’s post-hoc test for comparison of multiple columns. Differences were considered statistically significant when the *p*-value was less than 0.05.

## Results

### BHGZD Reverses the Imbalance of the “Immune-Inflammation” System in Active RA Rats

The AIA-M rat model was successfully established (the achievement ratio of this model was 100%) and verified according to its reliable and rapid-onset characteristics, and severe disease progression by external stimulus of wind, dampness, and heat, which may be the major pathological changes and features of active RA with moist heat arthralgia spasm syndrome (22). In contrast, the administration of BHGZD effectively alleviated the disease severity in arthritis of active RA rats. Detailed information on the characterizations of the active RA rat model and the therapeutic effects of BHGZD in active RA severity based on this model was reported in our previous study (22).

**Figure 1:**
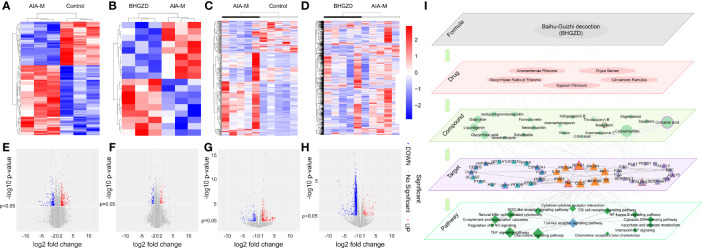
Transcriptomic profiling-based differential data analysis and biomolecular network-based investigation of AIA-M candidate markers and candidate targets of Baihu-Guizhi decoction (BHGZD) against AIA-M (Control, AIA-M, BHGZD). **(A)** The heatmap of the significant differentially expressed genes (DEGs) between the AIA-M model group and the normal control group in synovium using microarray analysis (*n* = 3 per group). **(B)** The heatmap of DEGs between the BHGZD treatment group and the AIA-M model group in synovium using microarray analysis (*n* = 3 per group). **(C)** The heatmap of the DEGs between the AIA-M model group and the normal control group in whole blood cells using RNA sequencing (RNA-Seq) analysis (*n* = 5 per group). **(D)** The heatmap of DEGs between the BHGZD treatment group and the AIA-M model group in whole blood cells using RNA-Seq analysis (*n* = 5 per group). **(E)** The volcano plot of the DEGs between the AIA-M model group and the normal control group in synovium using microarray analysis (*n* = 3 per group). **(F)** The volcano plot of the DEGs between the BHGZD treatment group and the AIA-M model group in synovium using microarray analysis (*n* = 3 per group). **(G)** The volcano plot of the DEGs between the AIA-M model group and the normal control group in whole blood cells using RNA sequencing (RNA-Seq) analysis (*n* = 5 per group). **(H)** The volcano plot of the DEGs between the BHGZD treatment group and the AIA-M model group in whole blood cells using RNA-Seq analysis (*n* = 5 per group). **(I)** The subnetwork of the “formula-drug-compound-target-pathway” network is constructed using the links between key RA-related genes and BHGZD effective genes. The ellipse refers to BHGZD. Light orange hexagons refer to drugs contained in BHGZD. Green circles refer to compounds contained in BHGZD. Triangles refer to targets. Green diamonds refer to pathways. Triangles with red circles refer to the targets of mangiferin (MG). Triangles with purple circles refer to the targets cinnamic acid (CA).

To determine the candidate targets of BHGZD against active RA, the gene expression profiles of whole blood cells and synovium tissues were carried out. A total of 473 RA-related genes (275 upregulated and 178 downregulated genes) and 1,802 BHGZD therapeutic effect-related genes (456 upregulated and 1,346 downregulated genes) were identified. The heatmap and volcano plot of DEGs are shown in **Figures 1A–H**, suggesting a distinct separation between the AIA-M model and normal control groups, as well as the BHGZD treatment and AIA-M model groups. Among them, there were 135 RA-related genes reversely regulated by the treatment of BHGZD (**Table 1**). Detailed information on the RA-related genes and BHGZD therapeutic effect-related genes is respectively provided in **Supplementary Tables S4** and **S5**.

**Table 1:** The information on 135 RA-related genes reversely regulated by the treatment of Baihu-Guizhi decoction (BHGZD).

Samples	Gene Symbol	AIA-M vs. Con	*p*-value	log2 Fold Change	BHGZD vs. AIA-M	*p*-value	log2 Fold Change
Synovium	ACKR4	Down	0.0389	−1.4327	Up	0.0011	1.1346
Synovium	AQP3	Down	0.0419	−4.6637	Up	0.0003	4.6114
Synovium	ARSI	Up	0.0157	1.1593	Down	0.0043	−1.9063
Synovium	C7	Up	0.0046	1.0014	Down	0.0142	−1.2088
Synovium	CAR13	Up	0.0411	1.3595	Down	0.0136	−1.9177
Synovium	CFB	Up	0.0087	2.3269	Down	0.0100	−1.9143
Synovium	CHL1	Up	0.0266	1.3184	Down	0.0082	−1.7127
Synovium	CUBN	Down	0.0440	−1.5011	Up	0.0272	2.0115
Synovium	CYP26A1	Up	0.0410	1.2507	Down	0.0032	−1.5831
Synovium	CYP2W1	Up	0.0302	1.6286	Down	0.0320	−2.3456
Synovium	EGLN3	Down	0.0224	−1.5984	Up	0.0216	1.4110
Synovium	EXPH5	Down	0.0254	−1.2262	Up	0.0204	1.6917
Synovium	GAS2	Up	0.0374	3.1797	Down	0.0478	−1.2633
Synovium	MARCKS	Up	0.0360	1.1328	Down	0.0142	−1.3571
Synovium	HHIP	Down	0.0135	−1.6433	Up	0.0307	1.4088
Synovium	ID4	Down	0.0244	−1.6145	Up	0.0089	1.3183
Synovium	PKHD1L1	Down	0.0199	−1.6321	Up	0.0298	1.6867
Synovium	TNMD	Up	0.0418	3.3639	Down	0.0245	−5.1964
Synovium	TUBB3	Up	0.0089	1.5756	Down	0.0031	−2.1800
Synovium	UNC5CL	Down	0.0172	−1.8548	Up	0.0164	1.3891
Synovium	UTS2R	Up	0.0234	3.6773	Down	0.0391	−3.3398
Synovium	VSNL1	Down	0.0251	−1.1707	Up	0.0161	1.0142
Whole Blood Cells	ADCY5	Up	1.5517	0.0011	Down	−1.4196	0.0001
Whole Blood Cells	AGRP	Up	1.4030	0.0234	Down	−2.2359	0.0001
Whole Blood Cells	ALDOC	Up	2.4691	0.0010	Down	−1.3832	0.0036
Whole Blood Cells	ANGPTL2	Up	3.7609	0.0417	Down	−2.6154	0.0384
Whole Blood Cells	ASTN1	Up	1.1875	0.0034	Down	−1.4422	0.0000
Whole Blood Cells	ATF5	Up	1.7382	0.0006	Down	−1.7147	0.0144
Whole Blood Cells	BFSP2	Up	1.3618	0.0039	Down	−1.5460	0.0004
Whole Blood Cells	BGLAP	Up	1.1948	0.0072	Down	−1.3003	0.0003
Whole Blood Cells	BRICD5	Up	4.9630	0.0180	Down	−4.5770	0.0112
Whole Blood Cells	BTLA	Down	−1.0068	0.0352	Up	1.0449	0.0059
Whole Blood Cells	CASP12	Down	−3.1532	0.0011	Up	2.2173	0.0236
Whole Blood Cells	CBLN2	Up	1.1423	0.0021	Down	−1.5381	0.0000
Whole Blood Cells	CCDC92	Up	1.7220	0.0000	Down	−1.7041	0.0001
Whole Blood Cells	CD177	Down	−1.9882	0.0031	Up	2.0804	0.0098
Whole Blood Cells	CD79AL	Down	−1.1535	0.0081	Up	1.1442	0.0045
Whole Blood Cells	CDCA2	Down	−3.2077	0.0117	Up	2.8039	0.0013
Whole Blood Cells	CELA1	Up	1.5977	0.0012	Down	−1.6140	0.0007
Whole Blood Cells	CLU	Up	1.0155	0.0039	Down	−1.4820	0.0000
Whole Blood Cells	CRABP1	Up	1.9562	0.0243	Down	−1.9772	0.0081
Whole Blood Cells	CRABP2	Down	−4.8634	0.0057	Up	3.8683	0.0101
Whole Blood Cells	CTDSPL	Up	1.0889	0.0348	Down	−1.0297	0.0089
Whole Blood Cells	CTTN	Up	1.0143	0.0137	Down	−1.9100	0.0000
Whole Blood Cells	CXCL2	Up	1.9025	0.0012	Down	−1.2825	0.0001
Whole Blood Cells	CXXC4	Up	1.1896	0.0126	Down	−1.5581	0.0000
Whole Blood Cells	DACT1	Up	3.5048	0.0091	Down	−2.7562	0.0059
Whole Blood Cells	DDX25	Down	−3.1180	0.0396	Up	2.9352	0.0077
Whole Blood Cells	DENND2C	Up	1.1059	0.0242	Down	−1.3200	0.0006
Whole Blood Cells	DNAH1	Up	1.1723	0.0186	Down	−1.6980	0.0002
Whole Blood Cells	DOK4	Up	1.2929	0.0109	Down	−1.2531	0.0079
Whole Blood Cells	ECE2	Up	3.5704	0.0297	Down	−7.0144	0.0000
Whole Blood Cells	EHD2	Up	1.0293	0.0323	Down	−1.7746	0.0001
Whole Blood Cells	ELN	Up	4.4083	0.0335	Down	−6.1763	0.0005
Whole Blood Cells	ERFE	Up	3.3489	0.0315	Down	−2.7202	0.0101
Whole Blood Cells	F11R	Up	1.1213	0.0037	Down	−1.5369	0.0000
Whole Blood Cells	F2RL3	Up	1.1166	0.0056	Down	−1.5841	0.0000
Whole Blood Cells	FAM184A	Up	1.6124	0.0416	Down	−1.5781	0.0007
Whole Blood Cells	GADD45G	Up	1.1221	0.0463	Down	−1.1012	0.0309
Whole Blood Cells	GAS2L1	Up	1.1011	0.0021	Down	−1.4515	0.0001
Whole Blood Cells	GNAZ	Up	1.0121	0.0086	Down	−1.4201	0.0000
Whole Blood Cells	GP1BB	Up	1.9090	0.0008	Down	−1.5758	0.0431
Whole Blood Cells	GP9	Up	1.0026	0.0038	Down	−1.4034	0.0002
Whole Blood Cells	GRB10	Up	2.6721	0.0005	Down	−1.3209	0.0028
Whole Blood Cells	GSTA1	Up	1.0067	0.0053	Down	−1.3929	0.0008
Whole Blood Cells	HIVEP3	Down	−1.7890	0.0017	Up	1.0399	0.0267
Whole Blood Cells	KATNAL1	Up	1.5441	0.0248	Down	−1.2866	0.0069
Whole Blood Cells	KCNS3	Up	1.0348	0.0105	Down	−1.4733	0.0003
Whole Blood Cells	KLHL13	Up	2.3729	0.0216	Down	−2.3771	0.0009
Whole Blood Cells	KLRA22	Down	−2.1066	0.0132	Up	1.6770	0.0175
Whole Blood Cells	LARGE1	Up	1.0049	0.0112	Down	−1.4699	0.0001
Whole Blood Cells	LHFPL6	Up	2.1809	0.0305	Down	−1.2276	0.0290
Whole Blood Cells	LIPG	Down	−1.2935	0.0374	Up	1.3060	0.0134
Whole Blood Cells	LOC100912228	Up	1.0055	0.0074	Down	−1.4317	0.0003
Whole Blood Cells	LOC102556092	Up	1.1719	0.0026	Down	−1.5513	0.0000
Whole Blood Cells	LOXL3	Up	1.3740	0.0022	Down	−1.4840	0.0002
Whole Blood Cells	LRRC71	Up	1.4124	0.0178	Down	−1.5799	0.0003
Whole Blood Cells	LY6G6F	Up	1.0222	0.0043	Down	−1.4740	0.0000
Whole Blood Cells	LYVE1	Up	1.0159	0.0023	Down	−1.3934	0.0000
Whole Blood Cells	MAPK8IP1	Up	2.5304	0.0317	Down	−2.4659	0.0016
Whole Blood Cells	MCPT1L1	Up	1.8206	0.0003	Down	−1.7125	0.0000
Whole Blood Cells	MEST	Up	1.5656	0.0296	Down	−1.7263	0.0007
Whole Blood Cells	MMRN1	Up	1.0609	0.0026	Down	−1.5474	0.0000
Whole Blood Cells	MMRN2	Down	−2.8201	0.0275	Up	3.0209	0.0015
Whole Blood Cells	MRVI1	Up	1.0361	0.0113	Down	−1.4740	0.0000
Whole Blood Cells	MSRA	Up	1.8396	0.0266	Down	−1.1511	0.0301
Whole Blood Cells	MYCT1	Up	1.1718	0.0058	Down	−1.5488	0.0000
Whole Blood Cells	MYL9	Up	1.2004	0.0009	Down	−1.5114	0.0000
Whole Blood Cells	NRGN	Up	1.1404	0.0008	Down	−1.4248	0.0001
Whole Blood Cells	NRIP3	Up	1.1768	0.0188	Down	−1.0978	0.0016
Whole Blood Cells	OLFM4	Down	−1.0349	0.0065	Up	1.1084	0.0336
Whole Blood Cells	PCDHGB5	Down	−3.9559	0.0262	Up	3.5018	0.0274
Whole Blood Cells	PDE3A	Up	1.0241	0.0116	Down	−1.4264	0.0001
Whole Blood Cells	PF4	Up	1.2675	0.0007	Down	−1.4629	0.0004
Whole Blood Cells	PKIA	Up	1.2370	0.0016	Down	−1.3519	0.0002
Whole Blood Cells	PLA2G2A	Up	1.3067	0.0003	Down	−1.5044	0.0001
Whole Blood Cells	PLOD2	Up	1.3673	0.0338	Down	−1.8337	0.0000
Whole Blood Cells	PLTP	Up	1.4100	0.0003	Down	−1.3760	0.0012
Whole Blood Cells	PPIF	Up	1.0631	0.0030	Down	−1.3334	0.0001
Whole Blood Cells	PROSER2	Up	2.4378	0.0106	Down	−1.4237	0.0073
Whole Blood Cells	PTPN13	Up	4.1086	0.0158	Down	−2.6389	0.0331
Whole Blood Cells	RASL10A	Up	1.1205	0.0028	Down	−1.3945	0.0001
Whole Blood Cells	REEP2	Up	1.0181	0.0048	Down	−1.4944	0.0000
Whole Blood Cells	RPAP1	Up	1.0304	0.0027	Down	−1.4851	0.0000
Whole Blood Cells	RPP25	Up	1.8057	0.0048	Down	−1.5779	0.0320
Whole Blood Cells	RT1-HA	Down	−1.3847	0.0248	Up	1.1581	0.0230
Whole Blood Cells	RTP4	Down	−1.1421	0.0350	Up	1.1822	0.0025
Whole Blood Cells	SCAI	Down	−1.2765	0.0173	Up	1.1195	0.0207
Whole Blood Cells	SCARF1	Up	1.7774	0.0006	Down	−1.5817	0.0020
Whole Blood Cells	SEC14L5	Up	1.3750	0.0134	Down	−1.8066	0.0013
Whole Blood Cells	SEMA5A	Up	2.8501	0.0110	Down	−4.0576	0.0003
Whole Blood Cells	SEPT10	Down	−1.7620	0.0426	Up	1.5958	0.0271
Whole Blood Cells	SERPINE2	Up	2.2082	0.0383	Down	−1.4497	0.0397
Whole Blood Cells	Sh3bgr	Up	2.4272	0.0004	Down	−1.3007	0.0077
Whole Blood Cells	SHPK	Up	1.0305	0.0005	Down	−1.1246	0.0000
Whole Blood Cells	SLC6A4	Up	1.0946	0.0021	Down	−1.5201	0.0000
Whole Blood Cells	SMPDL3B	Up	1.0133	0.0071	Down	−1.0129	0.0042
Whole Blood Cells	STON2	Up	1.8772	0.0063	Down	−1.6397	0.0004
Whole Blood Cells	SYT5	Up	1.1579	0.0003	Down	−1.3477	0.0002
Whole Blood Cells	SYTL4	Up	1.6712	0.0402	Down	−1.6984	0.0001
Whole Blood Cells	TAL1	Up	1.0319	0.0073	Down	−1.5858	0.0000
Whole Blood Cells	THBS1	Up	1.0434	0.0086	Down	−1.5613	0.0000
Whole Blood Cells	TMEM56	Up	1.7935	0.0015	Down	−2.3856	0.0015
Whole Blood Cells	TP53TG5	Up	4.6235	0.0109	Down	−3.3568	0.0168
Whole Blood Cells	TREML1	Up	1.0076	0.0069	Down	−1.4372	0.0002
Whole Blood Cells	TRIM17	Up	1.6098	0.0278	Down	−1.1120	0.0370
Whole Blood Cells	TYRO3	Up	1.0424	0.0372	Down	−1.7972	0.0000
Whole Blood Cells	UNC13B	Up	1.2061	0.0047	Down	−1.4375	0.0000
Whole Blood Cells	VSIG2	Up	1.4849	0.0204	Down	−1.2263	0.0039
Whole Blood Cells	VWF	Up	1.0792	0.0005	Down	−1.4591	0.0000
Whole Blood Cells	WNT2	Up	2.4945	0.0406	Down	−2.3451	0.0055
Whole Blood Cells	XKR8	Up	1.0216	0.0050	Down	−1.4189	0.0002
Whole Blood Cells	YAP1	Up	2.6466	0.0060	Down	−1.9990	0.0002
Whole Blood Cells	ZCCHC12	Up	3.7146	0.0010	Down	−2.1065	0.0024
Whole Blood Cells	ZFP780B-PS1	Down	−1.6098	0.0081	Up	1.3243	0.0131

AIA-M vs. Con: the significant differentially expressed genes (DEGs) between the AIA-M model group and the normal control group. BHGZD vs. BHGZD: the DEGs between the BHGZD treatment group and the AIA-M model group.

In addition, 238 putative targets of *Anemarrhena asphodeloides* Bge., *Cinnamomum cassia* Presl, and *Glycyrrhiza uralensis* Fisch., and 197 putative targets of Gypsum, contained in BHGZD with a high similarity score (>0.80), were predicted based on the TCMIP v2.0 platform, such as NAHD, HSD17B1, CFTR, TTGR, MIF, OXYR, CYP1A2, ESRRA, JAK1, ESRRB, CSNK2A1, ANXA5, ARSA, ATSK, C3L, CACNA1C, CACNA1S, CACNA2D1, CACNB1, CACNB2, CACNG1, CCKAR, CCL5, CD1D, CGIA, CRCA, CSLA, CSLB, and EGF. On the basis of the ETCM database, 184 putative targets of 31 bioactive chemical compounds contained in BHGZD, and 53 putative targets of *Oryza sativa* L. were collected, including ACO2, AKR1B1, ANG, APRT, BHMT, C8G, CPB1, CS, CTDSP1, GNMT, HGS, HS3ST3A1, VDR, CYP27B1, KCND1, KCNA3, PRKAB1, ADH1A, KCNA10, and GAMT. There were a total of 635 genes, including RA-related genes reversely regulated by BHGZD, putative targets of bioactive chemical compounds, and the known therapeutic targets of drug contained in BHGZD, considered as BHGZD effective genes.

Then, the interaction network of the “RA-related gene–BHGZD effective gene” was constructed using the links among RA-related genes and BHGZD effective genes. Following the calculation of the nodes’ topological features in the network (the median values of “Degree”, “Betweenness”, and “Closeness” were 7.0000, 0.0009, and 0.2860, respectively), four BHGZD candidate targets (PI3K, AKT1, NFκB, and IL-1β) against active RA (**Figure 1I**, and **Supplementary Table S6**) were identified due to their topological importance. In addition, previous data obtained from our research group and from other research groups indicated that BHGZD may restore the imbalance of the “immune-inflammation” system *via* inhibiting TLR4-induced NLRP3 inflammasome signaling (21, 22), and TLR4/NFκB signaling may play a vital role in the regulation of the inflammatory response (32, 33). Moreover, PI3K/AKT signaling is involved in the process and release of pro-inflammatory cytokines (34, 35), and the activation of this signaling leads to autoimmunity, showing the increased activity in some autoimmune diseases, including RA (36). On this basis, we hypothesize that BHGZD might reverse the main pathological changes of active RA, including synovial inflammation, cartilage damage, and bone erosion, *via* regulating TLR4/PI3K/AKT/NFκB/NLRP3 signaling.

### MG and CA May Be the Representative BACs Contained in BHGZD Against Active RA

To screen the potential BACs contained in BHGZD against active RA, herein we identified the chemical profiling of this herbal formula in sera using the UFLC-Q-TOF-MS/MS system. As a result, a total of 31 chemical compounds were identified in the serum samples 2 h after the treatment of BHGZD. Among them, 14, 16, and 1 chemical compound were from *Anemarrhenae Rhizoma*, *Glycyrrhizae Radix et Rhizoma*, and *Cinnamomi Ramulus*, respectively, and belong to glycosides, flavonoids, organic acid, triterpenoids, and saponins. Quantitatively, timosaponin B II, mangiferin, glycyrrhizic acid, neomangiferin, 7-O-methyl mangiferin, anemarrhensaponin I, vitexin, formononetin, liquiritigenin, isoliquiritin, isoliquiritin apioside, isoschaftoside, and isovitexin were all detected at 30 min after the treatment of BHGZD. Detailed information on chemical profiling is provided in **Supplementary Table S7**, and the quantitative detection data are provided in **Supplementary Table S8**. In addition, the drug-like properties of the chemical compounds contained in BHGZD, including the intestinal absorption rate and oral bioavailability, were evaluated using ADME models *in silico*, and a total of 8 candidate BACs with good drug-likeness were identified (**Supplementary Table S9**).

**Figure 2:**
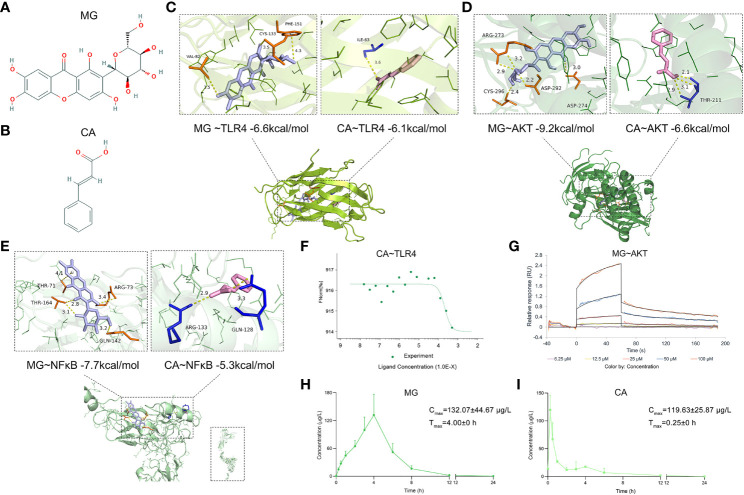
Chemical structures of MG and CA, molecular docking simulation of the binding patterns of them with corresponding proteins, as well as the mean plasma concentration–time curves of MG and CA after the treatment of BHGZD. **(A)** Chemical structure of MG (obtained from PubChem databases, CID 5281647). **(B)** Chemical structure of CA (obtained from PubChem databases, CID 444539). **(C)** Molecular docking simulation of the binding patterns of TLR4 with MG and CA, respectively. **(D)** Molecular docking simulation of the binding pattern of AKT with MG and CA, respectively. **(E)** Molecular docking simulation of the binding pattern of NFκB with MG and CA, respectively. **(F)** Microscale thermophoresis (MST)-determined binding affinity between CA and TLR4 protein. **(G)** Surface plasmon resonance (SPR) assay of the interaction between MG and AKT1 protein. **(H)** The mean plasma concentration–time curves of MG after the treatment of BHGZD for 12 days at the dosage of 21.4 g/kg each day (*n* = 3 per group). **(I)** The mean plasma concentration–time curves of CA after the treatment of BHGZD for 12 days at the dosage of 21.4 g/kg each day (*n* = 3 per group).

After that, molecular docking was performed to determine the binding affinities of candidate BACs contained in BHGZD to corresponding proteins of TLR4/PI3K/AKT/NFκB/NLRP3 signaling. As a result, a total of 9 chemical compounds were found to bind these proteins with binding affinities of more than −4.0 kcal/mol (**Supplementary Table S10**). In particular, MG (**Figure 2A**) from *Anemarrhenae Rhizoma* and CA (**Figure 2B**) from *Cinnamomi Ramulus* docked well into the TLR4, AKT, and NFκB binding cavity with more robust interactions and stronger binding affinity (MG–TLR4 −6.6, CA–TLR4 −6.1, MG–AKT −9.2, CA–AKT −6.6, MG–NFκB −7.7, and CA–NFκB −5.3 kcal/mol, **Figures 2C–E**). Experimentally, MST and SPR assays were carried out to verify the direct binding efficiency of CA to TLR4 and MG to AKT, respectively. As shown in **Figures 2F** and **G**, the mean *K*_D_ values of 2.11e−4 and 3.83e−5 M for CA–TLR4 and MG–AKT, respectively, indicated strong binding affinities.

Moreover, the content of MG and CA was determined to be 336.96 ng and 26.14 ng in 1 mg of BHGZD lyophilized powder, respectively, with a conversion ratio of 8.33% (calculated by the weight of BHGZD lyophilized powder divided by the weight of crude herbs contained in BHGZD; 1 g of BHGZD lyophilized powder is equivalent to the amount of 12 g of BHGZD).

The UHPLC-QTRAP-MS/MS system was further used to detect the pharmacokinetic properties of MG and CA in sera after the treatment of BHGZD (**Supplementary Figure S1**). A summary of MG and CA pharmacokinetic parameters using a non-compartment model analysis is presented in **Table 2**, and the mean plasma concentration–time profiles from time 0 to 24 h after the treatment of BHGZD at a dosage of 21.4 g/kg each day are shown in **Figures 2H** and **I**. Briefly, the maximum plasma concentration (C_max_) of MG and CA was 132.01 μg/L and 119.63 μg/L, respectively. The time to reach the maximum plasma concentration (T_max_) for MG and CA in rats receiving BHGZD was 4.00 h and 0.25 h, respectively. The plasma concentration–time curve AUC _(0-24h)_ of MG and CA was 578.42 μg/L·h and 102.48 μg/L·h, respectively. The apparent elimination half-life (T_1/2_z) and mean retention time (MRT) of MG (1.26 h and 4.23 h, respectively) and CA (1.65 h and 1.93 h, respectively) in rats after the treatment of BHGZD were measured.

**Table 2:** Pharmacokinetic parameters analyzed by the UHPLC-QTRAP-MS/MS system.

Pharmacokinetic Parameter	Mangiferin (MG)		Cinnamic acid (CA)	
	Mean ± SD	RSD/%	Mean ± SD	RSD/%
C_max_ (μg/L)	132.07 ± 44.67	33.80	119.63 ± 25.87	21.60
T_max_ (h)	4.00 ± 0.00	0.00	0.25 ± 0.00	0.00
AUC_(0-t)_ (μg/L*h)	578.42 ± 182.51	31.60	102.48 ± 67.77	66.10
AUC_(0-∞)_ (μg/L*h)	579.07 ± 181.70	31.40	144.23 ± 30.36	21.00
t_1/2_z (h)	1.26 ± 0.23	17.90	1.65 ± 1.34	81.10
MRT_(0-t)_ (h)	4.23 ± 0.30	7.10	1.93 ± 0.35	18.30
MRT_(0-∞)_ (h)	4.25 ± 0.28	6.50	2.26 ± 0.80	35.20

C_max_, the maximum plasma concentration. T_max_, the time to reach the maximum plasma concentration. AUC (0-t), area under the concentration–time curve from zero to the last sampling time. AUC (0-∞), area under the concentration–time curve from zero to infinity. T_1/2_z, apparent elimination half-life. MRT (0-t), mean residence time from zero to the last sampling time. MRT (0-∞), mean residence time from zero to infinity. Data are expressed as the mean ± SD.

### Both BHGZD and the Two-BAC Combination of MG and CA Improve Arthritis Severity in Active RA Rats

**Figure 3:**
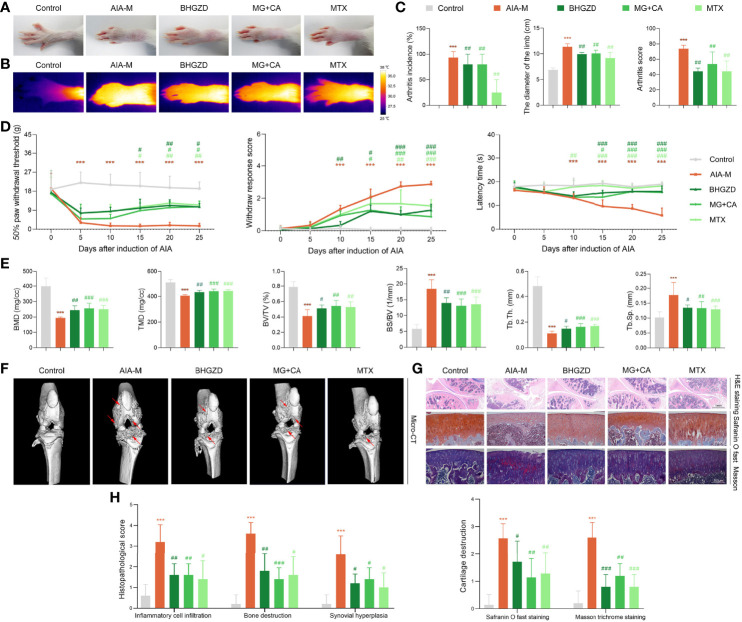
Therapeutic effects of BHGZD, two-BAC combination, and MTX on the severity of arthritis in AIA-M rats (Control, AIA-M, BHGZD, MG+CA, and MTX). **(A)** Representative images of arthritis. **(B)** Infrared thermography. **(C)** Arthritis incidence (*n* = 3 per group), the diameter of the limb (*n* = 5 per group), and arthritis score on the 27th day after immunization (*n* ≥ 4 per group, all experiments were performed in triplicate). **(D)** The pain thresholds (mechanical-, acetone-, and thermal-induced hyperalgesia, *n* ≥ 4 per group, all experiments were performed in triplicate). **(E)** Quantitative micro-computed tomography (micro-CT) analysis of bone mineral density (BMD), tissue mineral density (TMD), bone volume/tissue volume (BV/TV), bone surface/bone volume (BS/BV), trabecular separation (Tb.Sp), and trabecular thickness (Tb.Th) on the 31st day after immunization (*n* ≥ 5 per group, all experiments were performed in triplicate). **(F)** Representative micro-CT images of knee joints showing bone erosion levels (the red arrow indicates the position of the bone destruction). **(G)** Pathological changes in the knee joints using hematoxylin and eosin (H&E, scale bar represents 1 mm), safranin O fast green (scale bar represents 200 μm), and Masson trichrome staining (scale bar represents 200 μm) in different groups (*n* = 5 per group). **(H)** Quantitative analysis of H&E staining in inflammatory cell infiltration, bone destruction, and synovial hyperplasia. Cartilage destruction of safranin O fast, and Masson trichrome staining (*n* = 5 per group). Data are expressed as the mean ± SD. ***, *p* < 0.001, comparison with the normal control group; #, ##, ###, *p* < 0.05, *p* < 0.01, *p* < 0.001, respectively, comparison with the AIA-M model group.

To verify the pharmacological effects of the two-BAC combination of MG and CA against active RA, the *in vivo* experiments were performed based on the AIA-M rat model (22, 24). Similar pathological characteristics and changes in AIA-M rats were observed from the validation cluster to the discovery cluster (**Figure 3**). As a result, the ankles and knuckle joints of AIA-M rats with severe redness and swelling were remarkably improved by the treatment of the two-BAC combination (equivalent to BHGZD at a dose of 21.4 g/kg) and BHGZD (dose of 21.4 g/kg, **Figures 3A** and **B**). Importantly, both the treatment of BHGZD and the two-BAC combination significantly alleviated disease severity, including reducing arthritis incidence, the diameter of the limb, and arthritis score (all *p* < 0.05, on the 27th day after immunization), and simultaneously elevated pain thresholds (mechanical-, acetone-, and thermal-induced hyperalgesia), all of which were similar to the pharmacological effects of MTX (positive drug, dose of 0.2 mg/kg, **Figures 3C** and **D**).

In order to investigate the joint destruction and synovial inflammation, both the ankle and knee joints of AIA-M rats in different groups were scanned using Micro-CT analysis on the 31st day after immunization. The quantified data revealed that BMD, TMD, BV/TV ratio, and Tb.Th were dramatically decreased, and BS/BV ratio and Tb.Sp was observably increased in the AIA-M model group (all *p* < 0.05, **Figure 3E** and **Supplementary Figure S2B**) with rough bone surfaces and severe bone erosion (**Figure 3F**, and **Supplementary Figure S2A**). As shown in **Figure 3F**, both BHGZD and the two-BAC combination treatment groups showed smooth bone surface with a significantly increased BMD, TMD, BV/TV ratio, and Tb.Th, as well as a significantly decreased BS/BV ratio and Tb.Sp (all *p* < 0.05, **Figure 3E** and **Supplementary Figure S2B**), suggesting that the two-BAC combination may efficiently reverse bone erosion, which was similar to the pharmacological effects of MTX.

Moreover, H&E, safranin O fast green, and Masson trichrome staining were carried out to evaluate the degree of joint lesions in AIA-M rats. As shown in **Figures 3G** and **H**, the treatment of the two-BAC combination, BHGZD, and MTX all apparently reversed the histopathological changes of knee joints in AIA-M rats, including the decreased inflammatory cell infiltration, the prevention of cartilage and bone destruction, and synovial hyperplasia (all *p* < 0.05). Consistently, a significant reduction of the safranin O fast green-positive area and an elevation of the Masson-positive area were observed on the cartilage surface of AIA-M rats, reflecting a loss of articular cartilage, but reversed by the two-BAC combination, similar to the pharmacological effects of BHGZD and MTX (all *p* < 0.05, **Figures 3G** and **H**). These findings revealed that BHGZD and the two-BAC combination of MG and CA may effectively alleviate the progression of bone damages, repair bone erosion, and simultaneously improve pathological changes of articular cartilage and synovial inflammation in the arthritic joints of AIA-M rats.

In terms of the response to inflammation, pathological changes of the thymus and spleen and viscera indexes in different groups were examined. As shown in **Figure S2C**, the lower proportion of white pulp in the spleen with decreased cell density lymphatic sheath, lymphoid follicular hyperplasia, marginal zone, red pulp, and germinal center was observed in AIA-M rats, which were significantly improved by the treatment of BHGZD and the two-BAC combination (all *p* < 0.05, **Supplementary Figure S2D**). A thinner thymic cortex, less thymic lobule, an unclear boundary, and more vacuoles in the cytoplasm of epithelial reticular cells were also observed in AIA-M rats, which were remarkably improved by the treatment of BHGZD and the two-BAC combination (**Supplementary Figure S2C).** The treatment with BHGZD and the two-BAC combination protected the morphological structure of the spleen and thymus, and simultaneously decreased spleen and thymus indexes significantly (all *p* < 0.05, **Supplementary Figure S2E**). Furthermore, H&E-stained tissues of liver and kidney, and the liver and kidney indexes showed no toxic damages (**Supplementary Figures S2C** and **F**).

### Both BHGZD and the Two-BAC Combination of MG and CA Suppress NLRP3 Inflammasome-Induced Pyroptosis *Via* Regulating TLR4/PI3K/AKT/NFκB Signaling

**Figure 4:**
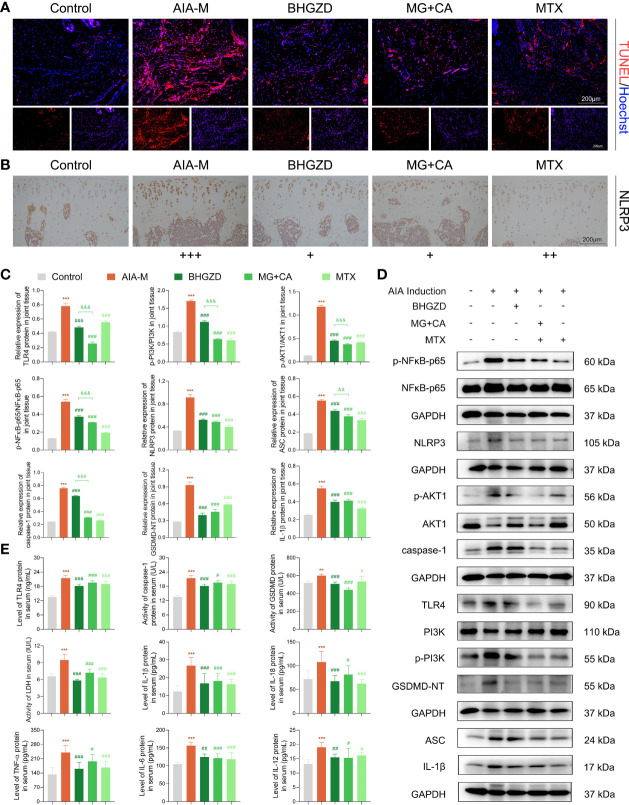
Regulatory effects of BHGZD, the two-BAC combination, and MTX on the expression of TLR4, p-PI3K/PI3K, p-AKT1/AKT1, p-NFκB-p65/NFκB-p65, NLRP3, ASC, caspase-1, GSDMD-NT, IL-1β, IL-18, IL-6, TNF-α, and IL-12 in AIA-M rats of different groups (Control, AIA-M, BHGZD, MG+CA, and MTX). **(A)** Representative images of pyroptosis in the knee joints of AIA-M rats detected by deoxynucleotidyltransferase-mediated UTP end labeling (TUNEL) assay (scale bar represents 200 μm, TUNEL red, Hoechst blue). **(B)** The expression of NLRP3 protein in the knee joints of AIA-M rats (scale bar represents 200 μm). **(C, D)** The protein expression of TLR4, p-PI3K/PI3K, p-AKT1/AKT1, p-NFκB-p65/NFκB-p65, NLRP3, ASC, caspase-1, GSDMD-NT, and IL-1β in the knee joints of AIA-M rats using Western botting analysis (*n* = 3 per group). **(E)** The expression levels of TLR4, TNF-α, IL-6, IL-1β, IL-18, and IL-12 in the sera of AIA-M rats using enzyme-linked immunosorbent assay (ELISA) analysis, and the activities of caspse-1, GSDMD-NT, and LDH in the sera of AIA-M rats using ELISA analysis (*n* ≥ 3 per group, all experiments were performed in triplicate). Data are expressed as the mean ± SD. **, ***, *p* < 0.01, *p* < 0.001, respectively, comparison with the normal control group; #, ##, ###, *p* < 0.05, *p* < 0.01, *p* < 0.001, respectively, comparison with the AIA-M model group; &&, &&&, *p* < 0.01, *p* < 0.001, comparison with the treatment of BHGZD.

The above network-based data imply that BHGZD might reverse the imbalance of the “immune-inflammation” system during active RA progression *via* regulating TLR4/PI3K/AKT/NFκB/NLRP3 signaling. To the best of our knowledge, pyroptosis is a kind of NLRP3 inflammasome-induced inflammatory cell death, characterized by cell swelling and release of pro-inflammatory cytokines depending on the activation of caspase-1, contributing to inflammation in arthritis (37). The formation of the NLRP3 inflammasome complex requires the interaction of NLRP3 with ASC and caspase-1, which are vital for the assembly and activation of the NLRP3 inflammasome. In our previous study, the immunomodulatory and anti-inflammatory activities of BHGZD were verified, especially in inhibiting pyroptotic death, which may be attributed to the activation of TLR4-NLRP3 inflammasome signaling (22). Currently, our TUNEL data showed a significant proportion of pyroptosis cells in the arthritic joints of AIA-M rats, which was improved by the treatment of BHGZD and the two-BAC combination (**Figure 4A**). Similar to the pharmacological effects of MTX, high expression levels of NLRP3 and ASC, high expression and enhanced activity of caspase-1, and increased expression of IL-1β and IL-18 (the signature inflammatory cytokines in pyroptosis) in the AIA-M model group (all *p* < 0.05) were all significantly decreased by the treatment of BHGZD and the two-BAC combination (all *p* < 0.05, **Figures 4B–E**). Pyroptosis was assessed by LDH activity, which may be used to verify cell membrane integrity and the release of intracellular soluble component. Consistently, the enhancing activity of LDH in the sera of AIA-M rats was observed, which was significantly decreased by the treatment of BHGZD, the two-BAC combination, and MTX (all *p* < 0.05, **Figure 4E**), suggesting the alleviation of downstream membrane damage.

**Figure 5:**
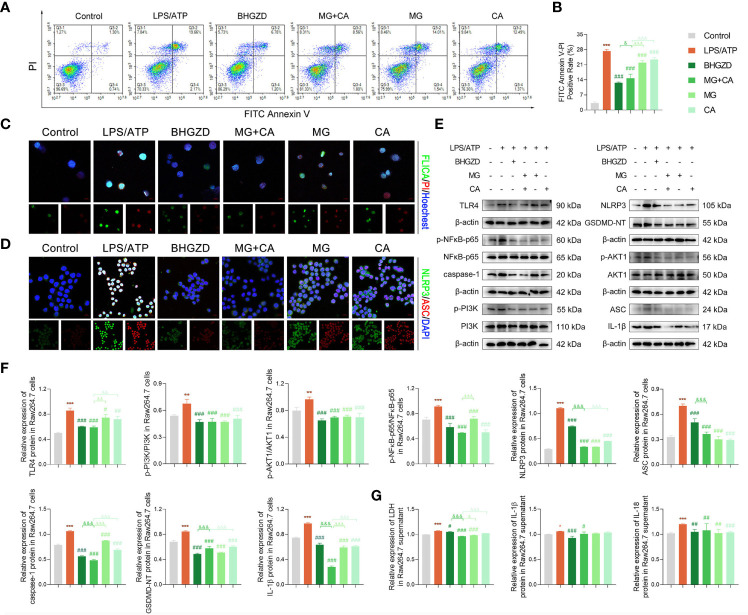
BHGZD inhibits LPS/ATP-induced pyroptosis in RAW264.7 macrophage by downregulating TLR4, p-PI3K/PI3K, p-AKT1/AKT1, p-NFκB-p65/NFκB-p65, NLRP3, ASC, caspase-1, GSDMD-NT, IL-1β, and IL-18 (Control, AIA-M, BHGZD, MG+CA, MG, and CA). **(A, B)** Flow cytometry analysis for Annexin V/PI staining in RAW264.7 cells (*n* ≥ 3 per group, all experiments were performed in triplicate). **(C)** Representative images of FAM-FLICA Casapse-1 that binds only to activated caspase-1 (scale bar represents 50 μm, FAM-FLICA green, PI red, Hoechst blue). **(D)** Expression of NLRP3 and ASC protein measured by immunofluorescent staining and confocal microscopy in RAW264.7 cells (scale bar represents 50 μm, NLRP3 FITC green, ASC CY3 red, DAPI blue). **(E, F)** Expression levels of TLR4, p-PI3K/PI3K, p-AKT1/AKT1, p-NFκB-p65/NFκB-p65, NLRP3, ASC, caspase-1, GSDMD-NT, and IL-1β in RAW264.7 cells measured by Western blotting (*n* = 3 per group). **(G)** Levels of IL-1β, IL-18, and LDH release in RAW264.7 cells detected by ELISA (*n* ≥ 3 per group, all experiments were performed in triplicate). *, **, ***, *p* < 0.05, *p* < 0.01, *p* < 0.001, respectively, comparison with the normal control group; #, ##, ###, *p* < 0.05, *p* < 0.01, *p* < 0.001, respectively, comparison with LPS/ATP-induced model; &, &&&, *p* < 0.05, *p* < 0.001, comparison with the treatment of BHGZD; △, △△, △△△, *p* < 0.005, *p* < 0.01, *p* < 0.001, comparison with the treatment of the two-BAC combination.

**Figure 6:**
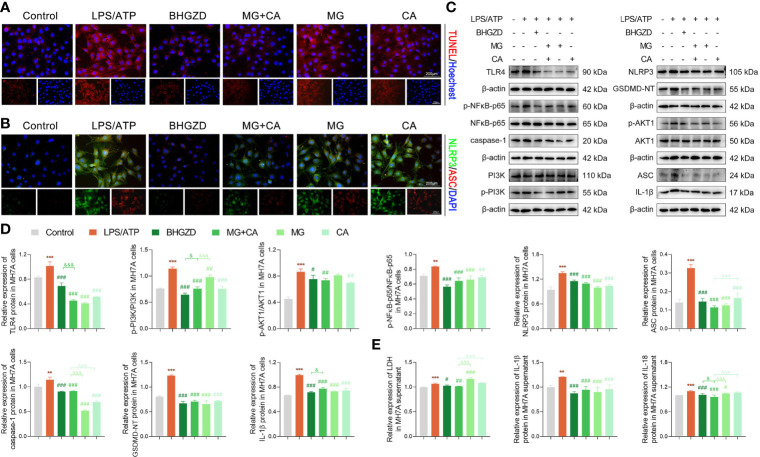
BHGZD inhibits LPS/ATP-induced pyroptosis in MH7A cells by downregulating TLR4, p-PI3K/PI3K, p-AKT1/AKT1, p-NFκB-p65/NFκB-p65, NLRP3, ASC, caspase-1, GSDMD-NT, IL-1β, and IL-18 (Control, AIA-M, BHGZD, MG+CA, MG, and CA). **(A)** TUNEL staining of MH7A cells (scale bar represents 200 μm, TUNEL red, Hoechst blue). **(B)** Expression of NLRP3 and ASC protein measured by immunofluorescent staining and confocal microscopy in MH7A cells (scale bar represents 200 μm, NLRP3 FITC green, ASC CY3 red, DAPI blue). **(C, D)** Expression levels of TLR4, p-PI3K/PI3K, p-AKT1/AKT1, p-NFκB-p65/NFκB-p65, NLRP3, ASC, caspase-1, GSDMD-NT, and IL-1β in MH7A cells measured by Western blotting (*n* = 3 per group). **(E)** Levels of IL-1β, IL-18, and LDH release in MH7A cells evaluated by ELISA (*n* ≥ 3 per group, all experiments were performed in triplicate). **, ***, *p* < 0.01, *p* < 0.001, respectively, comparison with the normal control group; #, ##, ###, *p* < 0.05, *p* < 0.01, *p* < 0.001, respectively, comparison with LPS/ATP-induced model; &, &&&, *p* < 0.05, *p* < 0.001, comparison with the treatment of BHGZD; △△△, *p* < 0.001, comparison with the treatment of two-BAC combination.

To verify the *in vivo* findings based on AIA-M rats, an established method (LPS plus ATP) was applied to induce NLRP3 inflammasome activation in both RAW264.7 and MH7A cells. The cell viability on RAW264.7 cells with the treatment of MG and CA was initially examined using the CCK8 assay, and the results exhibited no cell toxicity under 0.21–6.74 ng/ml MG or 0.02–0.52 ng/ml CA treatment alone, as shown in **Supplementary Figure S2G**. Thus, 1.69 ng/ml MG and 0.13 ng/ml CA treatment were chosen in the following assays (the same content as that in the 5 μg/ml BHGZD formula). Flow cytometry analysis revealed that MG, CA, and the two-BAC combination treatment prominently reduced the PI [a marker of cells that stains necrotic, dead, and membrane-compromised cells (38)] positive cell rate of RAW264.7 cells induced by LPS/ATP, which was inferior to that in the BHGZD treatment group (all *p* < 0.05, **Figures 5A** and **B**). As shown in **Figures 5C** and **6A**, LPS/ATP induced an increase in the number of both FLICA-positive RAW264.7 cells and TUNEL-positive MH7A cells, which was reduced by the treatment of BHGZD, MG, CA, and the two-BAC combination. Intriguingly, the inhibition of MG or CA alone on cell pyroptosis induced by LPS/ATP was weaker than that in the two-BAC combination treatment group (all *p* < 0.05, **Figures 5A–C** and **6A**). In addition, the expression levels of NLRP3, ASC, and caspase-1 were significantly increased in both RAW264.7 and MH7A cells induced by LPS/ATP, the same as the levels of IL-1β, IL-18, and LDH release in the supernatant of RAW264.7 and MH7A cells, which were all reduced by the treatment of BHGZD, MG, CA, and the two-BAC combination (all *p* < 0.05, **Figures 5D–G** and **6B–E**). GSDMD, a crucial mediator of pyroptosis downstream of canonical and non-canonical inflammasomes (39–41), is cleaved by caspase-1 at a specific site (GSDMD-NT) and subsequently causes cell lysis and IL-1β release (42, 43). In the current study, the activities of GSDMD-NT in sera and its levels in knee joints were both significantly elevated in AIA-M rats, which was reduced by the treatment of BHGZD, the two-BAC combination, and MTX (all *p* < 0.05, **Figures 4C–E**). Notably, GSDMD-NT occurred in both RAW264.7 and MH7A cells induced by LPS/ATP, which was decreased by the treatment of BHGZD, MG, CA, and the two-BAC combination (**Figures 5E, F** and **6C, D**).

After determining the inhibitory effects of BHGZD and the two-BAC combination on NLRP3 inflammasome-induced pyroptosis, we further investigated the changes of its upstream determinant TLR4/PI3K/AKT/NFκB signaling in different groups. Importantly, the expression levels of TLR4 in both sera and knee joints of AIA-M rats were increased, and subsequently were effectively reversed by the treatment of the two-BAC combination, with similar trends to BHGZD and MTX (all *p* < 0.05, **Figures 4C–E**). The protein expression ratios of p-PI3K/PI3K, p-NFκB-p65/NFκB-p65, and p-AKT1/AKT1 were all significantly increased in AIA-M rats, which were distinctively reduced by the treatment of BHGZD and the two-BAC combination (all *p* < 0.05, **Figures 4C, D**), indicating their inhibitory effects on TLR4/PI3K/AKT/NFκB signaling activation. In addition to the increased accumulation of inflammatory cells, the expression levels of inflammatory cytokines in AIA-M rats sera, including TNF-α, IL-6, and IL-12, were abnormally elevated in AIA-M rats, and reduced by the treatment of the two-BAC combination, in accordance with the effects of BHGZD and MTX (all *p* < 0.05, **Figure 4E**). Interestingly, we also achieved the same findings in the *in vitro* experiment validations based on both RAW264.7 and MH7A cells induced by LPS/ATP (all *p* < 0.05, **Figures 5E–G** and **6C–E**).

These findings demonstrated that both BHGZD and the two-BAC combination of MG and CA may suppress TLR4/PI3K/AKT/NFκB signaling-related protein activation, and subsequently inhibit NLRP3 inflammasome-induced pyroptosis.

## Discussion

Growing clinical evidence shows that various immune cells sustainably influx and migrate into the joints *via* secreting different types of immunomodulatory cytokines, leading to pyroptosis-induced persistent synovitis and cartilage degradation (44, 45). Thus, reversing the imbalance of the “immune-inflammation” system, especially alleviating pyroptosis, may be a promising therapeutic strategy for active RA. The current study performed an integrative research combining UFLC-Q-TOF-MS/MS, gene expression profiling, network calculation, pharmacokinetic profiling, SPR/MST assay, and pharmacological experiment validations, and identified TLR4/PI3K/AKT/NFκB/NLRP3 signaling-induced pyroptosis as one of the candidate effective targets of BHGZD for reversing the imbalance network of “immune-inflammation” during active RA progression. In addition, both MG and CA were identified as representative BACs acting on that target, for the strong binding affinities between compounds and target proteins, good pharmacokinetic features, and similar pharmacological effects to BHGZD. Notably, both BHGZD and the two-BAC combination of MG and CA effectively improved disease severity of active RA rats including elevating pain thresholds, relieving joint inflammation and bone erosion *via* inhibiting TLR4/PI3K/AKT/NFκB signaling to suppress the activation of the NLRP3 inflammasome, leading to the downregulation of downstream caspase-1, the reduced release of IL-1β, and the modulation of GSDMD-mediated pyroptosis. Consistent data were obtained based on the *in vitro* pyroptosis models of RAW264.7 and MH7A cells induced by LPS/ATP. Thus, RAW264.7 macrophages might be the probable immune cells targeted by both BHGZD and the two-BAC combination of MG and CA.

Currently, the AIA-M rat model was established simulating the pathogenetic characteristics of active RA with moist heat arthralgia spasm syndrome on the basis of male Lewis rats according to our previous studies (20–23). The abnormal changes in indicators reflecting the imbalance of the “immune-inflammation” system, including distinct redness and swelling, an increase in arthritis surface temperature, arthritis incidence, the diameter of the limb, arthritis score, pathologic changes of the thymus and spleen, as well as severe inflammatory cell infiltration, cartilage and bone destruction, synovial hyperplasia, and the high levels of inflammatory mediators, such as TLR4, IL-6, IL-12, IL-1β, IL-18, and TNF-α, were observed as the distinctive characteristics and biological basis of AIA-M rats, which may be in line with the clinical manifestations in active RA patients. Following the transcriptomic profiling and biomolecular network analyses, a series of active RA-related genes were also identified, and functionally involved in the regulation of the “immune-inflammation” system accordingly.

Considering that TCM contributed to the multi-target interactions of the complex ingredients of its herbal drugs (46–48), pharmaceutical development has consistently been an urgent challenge. Therefore, a new method that combines BACs contained in herbal formulas has been strongly indicated for new drug discovery. Herein, the two BACs, namely, MG from *Anemarrhenae Rhizoma* and CA from *Cinnamomi Ramulus*, were identified as the representative BACs of BHGZD for the strong binding affinities between compounds and target proteins, good pharmacokinetic properties, the high content and the importance of the formula, and similar pharmacological effects to BHGZD. Interestingly, the treatment of MG or CA alone did not exert prominently therapeutic effects compared to that of the two-BAC combination. Similar to the BHGZD formula, the two-BAC combination treatment of MG and CA may exert satisfying therapeutic efficacy on both *in vivo* and *in vitro* experiments *via* regulating TLR4/PI3K/AKT/NFκB signaling, which plays a vital role in synovial inflammation, cartilage degradation, and bone erosion by regulating inflammation response, immune disorder cells, and pyroptosis (37, 49–52). More importantly, the two-BAC combination of MG and CA offers key potential advantages over BHGZD for the following points. Firstly, the two-BAC combination may exert similar pharmacological effects in treating AIA-M rats with a definite material basis. Secondly, the two-BAC combination may be flexible in design, easily synthesized on a large scale, easily absorbed, and relatively stable, not dependent on the cultivations of crude herbs. Thirdly, it is better to understand the underlying molecular mechanisms of TCM-based RA therapeutics.

**Figure 7:**
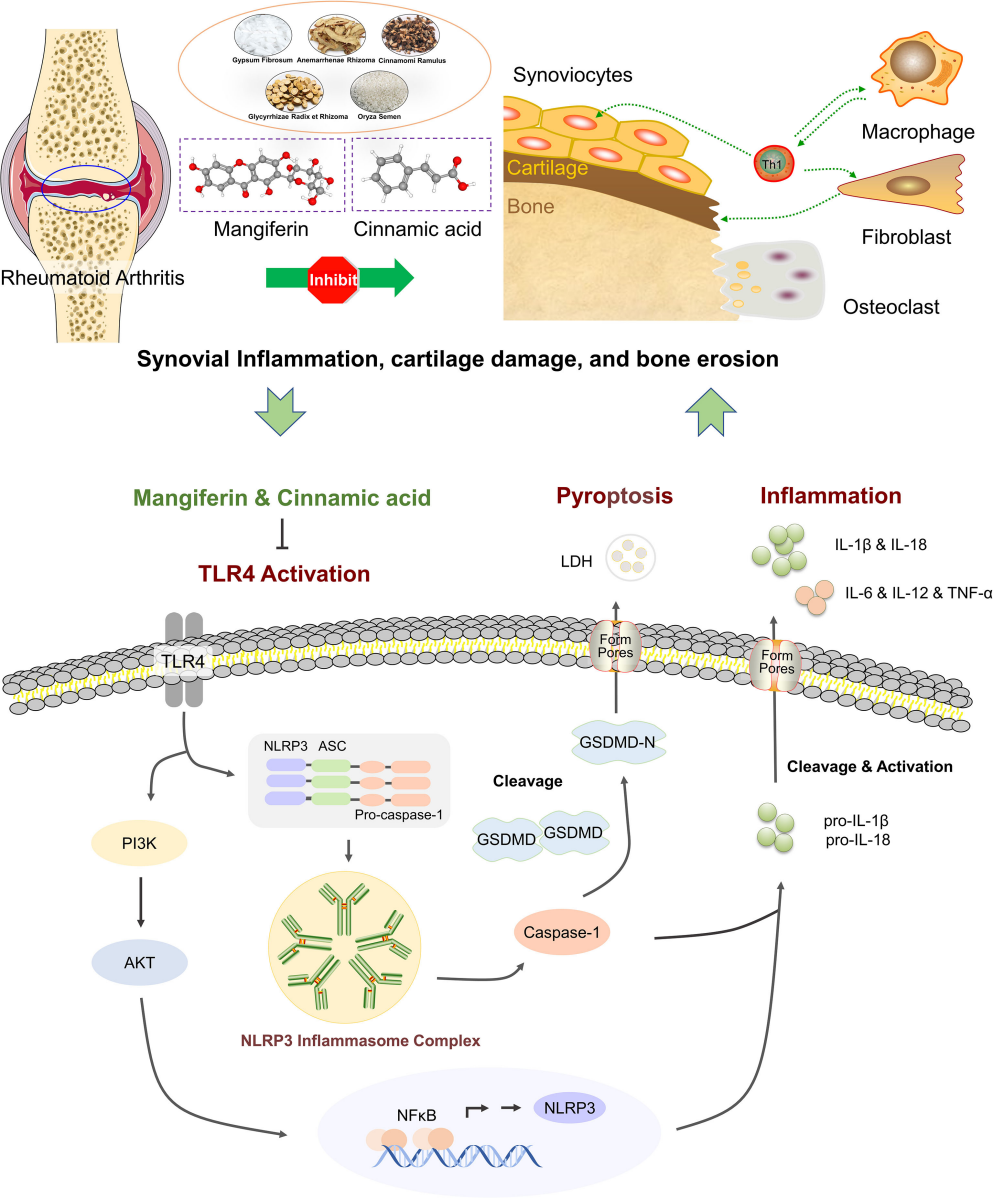
The schematic diagram of the underlying mechanisms of the two-BAC combination of mangiferin (MG) and (CA) against active RA *via* reversing the imbalance of the “immune-inflammation” system by inhibiting TLR4/NFκB/NLRP3 activation-induced pyroptosis. “⟂“: Inhibition. “→“: Activation.

In conclusion, our data offer an evidence that the MG and CA combination from BHGZD may interact with TLR4/PI3K/AKT/NFκB signaling to inhibit NLRP3 inflammasome activation and modulate pyroptosis, which provides the novel representative BACs and pharmacological mechanisms of BHGZD against active RA (**Figure 7**). The findings may shed new light on the mechanisms of the TCM formula, and promote the modernization development of TCM and drug discovery.

## Data Availability Statement

The datasets presented in this study can be found in online repositories. The name of the repository and accession number(s) can be found below: NCBI Gene Expression Omnibus; GSE190523, GSE189942.

## Ethics Statement

The study was approved by the Research Ethics Committee of the Institute of Chinese Materia Medica, China Academy of Chinese Medical Sciences, Beijing, China [Ethics Approval Number: 2019-026 and IBTCMCACMS21-2105-01, certificate number of the facility: SYXK (Beijing) 2021-0017]. All animal studies were treated in accordance with the guidelines and regulations for the use and care of animals of the Center for Laboratory Animal Care, China Academy of Chinese Medical Sciences. All animal-handling procedures were performed according to the *Guide for the Care and Use of Laboratory Animals* of the National Institutes of Health and followed the guidelines of the Animal Welfare Act.

## Author Contributions

NL and YZ engaged in study design and coordination, material support for obtained funding, and supervised study. YZ designed the experimental validation and revised the manuscript. WL performed most of the experiments and statistical analysis, as well as wrote the manuscript. SY and WS performed parts of the experiments, reviewed and approved the final manuscript. The other authors performed parts of the experiments. All authors contributed to the article and approved the final manuscript.

## Funding

This study is funded by the National Natural Science Foundation of China (81630107), the Scientific and Technological Innovation Project of China Academy of Chinese Medical Sciences (CI2021A03808 and CI2021A01508), and the National Key Research and Development Program of China (2018YFC1705201). No study sponsors are involved in the research process of this project.

## Conflict of Interest

The authors declare that the research was conducted in the absence of any commercial or financial relationships that could be construed as a potential conflict of interest.

## Publisher’s Note

All claims expressed in this article are solely those of the authors and do not necessarily represent those of their affiliated organizations, or those of the publisher, the editors and the reviewers. Any product that may be evaluated in this article, or claim that may be made by its manufacturer, is not guaranteed or endorsed by the publisher.

## Glossary

ADME: absorption distribution metabolism excretion
AIA: adjuvant-induced arthritis
AKT1: RAC: alpha serine/threonine-protein kinase
ANOVA: analysis of variance
ASC: apoptosis-associated speck-like protein containing a CARD
ATP: adenosine triphosphate
BAC: bovine serum albumin
BACs: bioactive compounds
BHGZD: Baihu-Guizhi decoction
BMD: bone mineral density
BS/BV: bone surface/bone volume
BV/TV: bone volume/tissue volume
CA: cinnamic acid
CASP1: caspase-1
CCK-8: cell counting kit-8
Cmax: the maximum plasma concentration
DAPI: 4’, 6-diamidino-2-phenylindole
DMARDs: disease-modifying anti-rheumatic drugs
ELISA: enzyme-linked immunosorbent assay
FBS: fetal bovine serum
FDA: Food and Drug Administration
GSDMD: gasdermin
H&E staining: hematoxylin and eosin staining
IL-12: interleukin-12
IL-18: interleukin-18
IL-1β: interleukin-1 beta
IL-6: interleukin-6
LDH: lactate dehydrogenase
LPS: lipopolysaccharide
MG: mangiferin
Micro-CT: micro-computed tomography
MST: Microscale thermophoresis
MTX: methotrexate
NFκB-P65: NF-kappa B transcription factor p65 subunit
NLRP3: NOD-like receptor pyrin domain containing 3
NSAIDs: non-steroidal anti-inflammatory drugs
PBS: phosphate-buffered saline
PFA: paraformaldehyde
PI3K: phosphatidylinositol 3 kinase
PIK3CG: phosphatidylinositol 4, 5-bisphosphate 3-kinase catalytic subunit gamma isoform
PK: pharmacokinetic
RA: rheumatoid arthritis
RNA-Seq: RNA sequencing
ROI: region of interest
SPR: surface plasmon resonance
Tb.Sp: trabecular separation
Tb.Th: trabecular thickness
TCM: traditional Chinese medicine
TLR4: toll-like receptor 4
Tmax: the time to reach the maximum plasma concentration
TMD: tissue mineral density
TNF: tumor necrosis factor
TUNEL assay: deoxynucleotidyltransferase-mediated UTP end labeling
TwHF: Tripterygium wilfordii Hook F
UFLC-Q-TOF-MS/MS: the ultra-fast liquid chromatography-quadrupole-time-of-flight tandem mass spectrometry
WTD: Wu-Tou decoction
