# Supplementary material for: A Novel Drug Combination of Mangiferin and Cinnamic Acid Alleviates Rheumatoid Arthritis by Inhibiting TLR4/NFκB/NLRP3 Activation-Induced Pyroptosis
Source: Front Immunol. 2022 Jun 21;13:912933. doi: 10.3389/fimmu.2022.912933 (PMC9253268; doi:10.3389/fimmu.2022.912933)
Supplement: Supplementary file 1 [file DataSheet_1.pdf]

Supplementary Materials for A Novel Drug Combination of Mangiferin and Cinnamic Acid Alleviates Rheumatoid Arthritis by Inhibiting TLR4/NFκB/NLRP3 Activation-induced Pyroptosis

List of Supplementary Materials

Section 1 Preparation of Baihu-Guizhi decoction (BHGZD)

Section 2 AIA-M rat model induction

Section 3 Severity assessment of arthritis

Section 4 Measurement of mechanical-, acetone- and thermal-induced hyperalgesia

Section 5 Hematoxylin and eosin (H&E) staining

Section 6 Micro-computed tomography (micro-CT) analysis

Section 7 The significant differentially expressed genes (DEGs) screening

Section 8 Defining network topological feature set

Section 9 Identification of the bioactive compounds (BACs) contained in Baihu-Guizhi decoction (BHGZD)

Section 10 Prediction of absorbed compounds and their metabolites using *in silico* absorption distribution metabolism excretion (ADME) models

Section 11 Surface plasmon resonance (SPR) detection

Section 12 Microscale thermophoresis (MST) analysis

Section 13 Pharmacokinetic analysis

Section 14 The culture conditions of Raw264.7 and MH7A cells

Section 15 NLRP3 inflammasome activation

Section 16 Cell proliferation assay

Section 17 Flow cytometry

Section 18 Terminal deoxynucleotidyl transferase-mediated dUTP biotin nick end labeling (TUNEL) staining

Section 19 FAM-FLICA caspase-1 assay

Section 20 Immunofluorescence staining

Section 21 Characterizations and biological basis of active RA rat model

Section 22 BHGZD alleviates the disease severity in arthritis of active RA rats

Table S1 Composition of Baihu-Guizhi Decoction (BHGZD)

Table S2 The detailed information on reference standards

Table S3 The antibodies used for western blotting analysis

Table S4 The significant differentially expressed genes (DEGs) identified by microarray analysis

Table S5 The significant differentially expressed genes (DEGs) identified by mRNA Sequencing (mRNA-Seq)

Table S6 The topological feature of nodes in the interaction network of RA-candidate-marker gene-BHGZD-effective gene

Table S7 The chemical profiling of Baihu-Guizhi Decoction (BHGZD) using UFLC-Q-TOF-MS/MS system.

Table S8 13 chemical compounds were quantitatively determined using UFLC-Q-TOF-MS/MS system

Table S9 The absorption distribution metabolism excretion (ADME) information on 19 bioactive compounds (BACs) included in Baihu-Guizhi Decoction (BHGZD)

Table S10 Molecular docking results by Autodock Vina

Figure S1 Total ion chromatogram for sera in normal control group after the treatment of Baihu-Guizhi decoction (BHGZD) by UHPLC-QTRAP-MS/MS system.

Figure S2 Micro-computed tomography (micro-CT) analysis and pathological changes in the liver, kidney, spleen, and thymus of different groups using hematoxylin and eosin (H&E) staining based on AIA-M rats. And the cell viability on Raw264.7 cells with the treatment of MG and CA tested using cell counting kit-8 (CCK8) assay.

Section 1 Preparation of Baihu-Guizhi decoction (BHGZD)

BHGZD was prepared according to the original composition of this formula recorded in Chinese Pharmacopoeia 2020 edition (**Supplementary Materials Table S1**), which were purchased from Beijing Tongrentang Co., Ltd (Beijing, China). Five crude drugs [*Gypsum Fibrosum* (60g), *Anemarrhenae Rhizoma* (15g), *Cinnamomi Ramulus* (10g), *Oryza Semen* (30g), and *Glycyrrhizae Radix et Rhizoma* (5g)] were separately soaked with pure water for 1h. Then, *Gypsum Fibrosum* was added to 1200 mL (1:10, *g/v*) of boiling pure water and boiled for 30 min. The herb mixture was added and boiled for 30min. The filtrates were collected, and the residues were decocted in 600mL (1: 5, *g/v*) of pure water for 20min. The filtrates from each decoction were combined and concentrated to 2g/mL using rotary evaporator. The obtained BHGZD was kept at -20℃ for preparation. Combined and concentrated the filtrate under reduced pressure and dried out in 70℃ oven overnight to obtain BHGZD powder.

Section 2 AIA-M rat model induction

Induction of AIA model was performed as previous reported (1-3), and AIA-M rat model was established to simulate the pathological changes and characteristics of active RA after AIA model induction. Briefly, male Lewis rats were injected intradermally at the base of tail with 10mg/mL M *tuberculosis* H37 Ra (Difco, BD company, New Jersey, US) suspended in liquid paraffin (Freund's complete adjuvant, CFA). From the day of primary immunization, male Lewis rats were kept in the artificial climate box (production license no. RXZ-380A-LED) for 2h daily with certain wind velocity (6m/s), temperature (37℃) and

humidity (90%) for a period of 15 days.

Section 3 Severity assessment of arthritis

Rats were observed once every day after primary immunization. Arthritis severity was evaluated by arthritis incidence, the diameter of limb, arthritis score, and arthritis surface temperature, which were performed by two independent, blinded observers. The arthritis score was the total of the scores for all 4 limbs (maximum possible arthritis score 80). Arthritis incidence values are the number positive/total number in groups for 11 days consecutively. The diameter of limb was measured with a vernier caliper.

The temperature of the articular surface, awarded to the left hind paw of male Lewis rats, was measured using an infrared thermal imager (TESTO-875, Testo AG, Schwarzwald, Germany) once a day from the day when the first signs of inflammatory were observed.

Section 4 Measurement of mechanical-, acetone- and thermal-induced hyperalgesia

Mechanical-induced hyperalgesia was assessed with Von Frey filaments by using Dixon's up-and-down paradigm (4). The rats were habituated in individual clear boxes on a wire-mesh platform, to allow access to the ventral surface of the hind paws, and mechanical hypersensitivity was assessed by the sensitivity to the application of von Frey hairs (Stoelting Co., Chicago, USA). The von Frey filaments (1.4~180g) were presented perpendicularly to the plantar surface of the injected paw and held in this position for 2~3s with enough force to cause a slight bend in the filament. Positive responses included an abrupt withdrawal of the hind paw or flinching behaviors immediately following removal of the stimulus, and 50% paw withdrawal threshold was determined at 0, 5, 10, 15, 25th day

after BHGZD administration (5), respectively.

The rats were acclimatized to an apparatus consisting of individual perspex boxes. With the aid of syringe (1mL), 500μL acetone was sprayed gently from a short distance to the middle of the plantar surface of hind paw. The acetone quickly spread over the proximal half of the plantar surface of the foot. The amount of time that rats were licking and/or shaking the hind paw was recorded during 5min after acetone application and was used as an index of nocifensive response for cold hypersensitivity (6). The licking and/or shaking time was measured at 0, 5, 10, 15, 25th day after BHGZD administration, respectively.

For thermal-induced hyperalgesia, the rats were placed for 30minin an apparatus consisting of individual perspex boxes with Hargreaves radiant heat apparatus (Ugo Basile Srl, Comerio VA, Italy) and an infrared radiant heat (40W) source was directed to middle part of the plantar surface of the hind paw. A cutoff time of 20s was set to prevent tissue injury. The paw withdrawal latency was measured at 0, 5, 10, 15, 25th day after BHGZD administration, respectively. The interval of the paw withdrawal latency measurement was 5min (7).

Section 5 Histology

Hematoxylin and eosin (H&E) staining was conducted according to routine protocols. Briefly, after deparaffinization and rehydration, tissue sections were stained with hematoxylin solution (ZSGB-BIO, China) for 5min followed by 5 dips in 1% acid ethanol (1% HCL in 75% ethanol) and then rinsed in distilled water. Then the sections were stained with eosin solution (ZSGB-BIO, China) for 3min and followed by dehydration with graded

alcohol and clearing in xylene. Images were captured and analyzed by K-Viewer software (version 1.5.3.1; KFBIO; http://www.kfbio.cn).

Samples were cut into 5-μm-thick longitudinally oriented sections and processed for H&E, Masson trichrome staining, and Safranine O-Fast Green staining.

Section 6 Micro-computed tomography (micro-CT) analysis

To quantitatively evaluate bone formation within the defects, the specimens were scanned using a micro-CT instrument (GE healthcare, USA) at a resolution of 45μm. The X-ray settings were standardized to 80kVp and 450μA with an exposure time of 400ms. The 3D images were reconstructed to analyze the new bone formation by micro-CT analysis software (Micro View ™, Version 2.2). The cylinder region of interest (ROI) was quantified by the values of the bone mineral density (BMD), tissue mineral density (TMD), bone volume to tissue volume (BV/TV), bone volume/tissue volume (BS/BV), the trabecular thickness (Tb.Th.), and trabecular separation (Tb.Sp.).

Section 7 The significant differentially expressed genes (DEGs) screening

1) DEGs in the synovium

Synovium of joint of Control, AIA-M model and BHGZD treatment rats were dissected under aseptic conditions, fixed immediately in Trizol (n=3 per group), taking into consideration the comprehensive factors such as the arthritis score and pain sensitivity of AIA-M rats.

RNA extraction and purification

Total RNA was extracted using TAKARA RNAiso Plus#9109 following the manufacturer's

instructions and checked for a RIN number to inspect RNA integrity by an Agilent Bioanalyzer 2100 (Agilent technologies, Santa Clara, CA, US). Qualified total RNA was further purified by RNeasy micro kit (Cat#74004, QIAGEN, GmBH, Germany) and RNase-Free DNase Set (Cat#79254, QIAGEN, GmBH, Germany).

RNA amplification and labeling

Total RNA was amplified and labeled by Low Input Quick Amp Labeling Kit, One-Color (Cat.# 5190-2305, Agilent technologies, Santa Clara, CA, US), following the manufacturer's instructions. Labeled cRNA were purified by RNeasy mini kit (Cat.# 74106, QIAGEN, GmBH, Germany).

Microarray hybridization

Each slide was hybridized with 1.65μg Cy3-labeled cRNA using Gene Expression Hybridization Kit (Cat.# 5188-5242, Agilent technologies, Santa Clara, CA, US) in Hybridization Oven (Cat.# G2545A, Agilent technologies, Santa Clara, CA, US), according to the manufacturer's instructions. After 17h hybridization, slides were washed in staining dishes (Cat.# 121, Thermo Shandon, Waltham, MA, US) with Gene Expression Wash Buffer Kit (Cat.# 5188-5327, Agilent technologies, Santa Clara, CA, US), followed the manufacturer's instructions.

Data acquisition

Slides were scanned by Agilent Microarray Scanner (Cat#G2565CA, Agilent technologies, Santa Clara, CA, US) with default settings, Dye channel: Green, Scan resolution=3μm, PMT 100%, 20bit. Data were extracted with Feature Extraction software 10.7 (Agilent technologies, Santa Clara, CA, US). Raw data were normalized by Quantile algorithm,

limma packages in R.

2) DEGs in the whole blood cells

Whole blood cell of Control, AIA-M model and BHGZD treatment rats were collected and isolated (n=5 per group), taking into consideration the comprehensive factors such as the arthritis score and pain sensitivity of AIA-M rats.

Total RNA was isolated using RNeasy mini kit (Qiagen, Germany). Paired-end libraries were synthesized by using the TruSeq™ RNA Sample Preparation Kit (Illumina, CA, USA) following TruSeq™ RNA Sample Preparation Guide. Briefly, the poly-A containing mRNA molecules were purified using poly-T oligo-attached magnetic beads.

Following purification, the mRNA is fragmented into small pieces using divalent cations under 94℃ for 8 min. The cleaved RNA fragments are copied into first strand cDNA using reverse transcriptase and random primers. This is followed by second strand cDNA synthesis using DNA Polymerase I and RNase H. These cDNA fragments then go through an end repair process, the addition of a single 'A' base, and then ligation of the adapters. The products are then purified and enriched with PCR to create the final cDNA library. Purified libraries were quantified by Qubit® 2.0 Fluorometer (Life Technologies, USA) and validated by Agilent 2100 bioanalyzer (Agilent Technologies, USA) to confirm the insert size and calculate the mole concentration. Cluster was generated by cBot with the library diluted to 10pM and then were sequenced on the Illumina NovaSeq 6000 (Illumina, CA, USA).

The library construction and sequencing were performed at Shanghai Sinomics Corporation.

Section 8 Defining network topological feature set

For each node i in interaction network, we defined three measures for assessing its topological property: (1) "Degree" is defined as the number of links to node i; (2) "Betweenness" is defined as the number of shortest paths between pairs of nodes that run through node i. (3) "Closeness" is defined as the inverse of the farness which is the sum of node i distances to all other nodes. The Closeness centrality can be regarded as a measure of how long it will take to spread information from node i to all other nodes sequentially. Degree, node betweenness and closeness centralities can measure a node’s topological importance in the network. The larger a node’s degree/betweenness/closeness centrality is, the more important the node is in the interaction network (8).

Section 9 Identification of the bioactive compounds (BACs) contained in Baihu-Guizhi decoction (BHGZD)

1) Preparation of BHGZD samples

Serum sample (100μL) was precipitated with 200μL of pre-freezing acetonitrile (containing D4-naringin 50ng/mL, IS). After vortexed for 2min, all samples subsequently centrifuged at 15000rpm for 20min at 4℃. The supernatants were subjected for qualitative and quantitative analysis.

2) UFLC-Q-TOF-MS/MS conditions

Chemical analysis was conducted on a connected system of UFLC XR (Shimadzu Corp., Japan)-hybrid triple quadruple time-of-flight mass spectrometer (Triple TOF^{TM} 5600^{+}, AB

Sciex, Forster City, USA) with electrospray ionization source (ESI). Kinetex C18 column (Phenomenex, 3.0×150 mm, 2.6μm, 100Å) was used to perform chromatographic separation with a flow rate of 0.3mL/min at 40℃. A linear gradient program with mobile phase system including solvent A (deionized water with 0.1% formic acid, *v/v*) and solvent B (methanol) was described in details: solvent B (5~95%) for 40min, isocratic eluted at 95% B for 5min，95%~5% B (40-45min), followed a post-run of 2min to equilibrate the system.

The instrumental settings of Q-TOF-MS/MS were described in the previous study (9).Samples were analyzed in both positive and negative ionization modes with scanning mas-to-charge (*m/z)* range from 50 to 1500. Data was collected in information-dependent acquisition (IDA) mode and analyzed by PeakView® 2.2 software (AB Sciex, Foster City, CA, USA). Chemical identifications were based on reference standards and mass spectral library (Natural Products HR-MS/MS Spectral Library, Version 1.0, AB Sciex, Forster City, USA).

Section 10 Prediction of absorbed compounds and their metabolites using in silico absorption distribution metabolism excretion (ADME) models

Structural information (*.mol or *.sdf files) relating to the bioactive components (identified by UFLC-Q-TOF-MS/MS) were downloaded from ChemSpider (http://www.chemspider.com/). ADME evaluation of these compounds was carried out using ACD/Percepta software 5.07 (ACD/Labs, Toronto, Canada), including the passive intestinal permeability of Caco-2 module and the pharmacokinetic explorer module to predict their oral bioavailability. The apparent permeability coefficient (Papp) of one

compound was greater than 9.0×10^{6}cm/s and 40% which indicated drug-like characteristics.

Section 11 Surface plasmon resonance (SPR) assay

SPR binding assays were analyzed with Biacore 8K (Biacore, Cytiva). Materials and reagents used in SPR assays were purchased from Cytiva. The histidine-tagged AKT1 (Cat No. ab116412, Abcam, Cambridge, UK) was immobilized on the surface of Carboxymethylated (CM5) sensor chip through the standard amino coupling method. The carboxyl groups of the sensor surface were activated by injection of a solution containing 0.2M N-ethyl-N9-(3-dimethylaminopropyl) carbodiimide (EDC) and 0.05M N-hydroxysuccinimide (NHS), AKT1 in 10mM sodium acetate buffer was then injected at a flow rate of 10μL/min to couple to the sensor surface, and the remaining active sites in the flow cell were finally blocked by 1M ethanolamine (EA). Sensor chips were primed twice with degassed physiological running buffer (PBS buffer). A reference flow cell was activated and blocked in the absence of AKT1. In the direct binding, the AKT1 immobilization level was fixed at 9000RU, and then different concentrations of AKT1 in the corresponding buffer containing 5%DMSO *(v:v)* were serially injected into the channel to evaluate binding affinity. MG was dissolved in DMSO, and diluted to a concentration series using PBS buffer, and then injected. The Biacore 8K evaluation software 2.0 (GE Healthcare) was used for calculating the related K_D value.

Section 12 Microscale thermophoresis (MST) analysis

The binding affinity between recombinant TLR4 (Cat No. ab233665, Abcam, Cambridge,

UK) and CA contained in BHGZD was measured by NanoTemper Monolith NT.115 instrument (NanoTemper Technologies, Germany). TLR4 was firstly adjusted to a concentration of 10μM and then labeled with Monolith NT.115 Protein Labeling Kit RED-NHS (NanoTemper Technologies, Germany) according to the protocol of the manufacture. After the protein labeling experiment was completed, TLR4 was diluted with binding buffer (20mM HEPES, pH 7.4, 100mM NaCl and 0.005% Tween 20) to ensure that the fluorescence intensity of TLR4 during the MST assay was about 500 RU. In this assay, the final concentration of TLR4 after mixing with CA was 20nM. In the following, CA were serially diluted in binding buffer (16 points, 1:2 dilutions, from 0.5mM), then mixed and incubated with an equal volume of the diluted TLR4 at room temperature for 3, 8, and 16h. After incubation, the samples were loaded into the premium treated capillaries and measured in NanoTemper Monolith NT.115 instrument (NanoTemper Technologies, Germany). The K_D value was fitted by NanoTemper Monolith affinity software (NanoTemper Technologies, Germany) using 1:1 binding mode.

Section 13 Pharmacokinetic analysis

To detect the pharmacokinetics parameter of MG and CA, respectively, after oral administration of BHGZD, ultra-fast liquid chromatography coupled with triple quadrupole-linear ion trap composite mass spectrometry (UHPLC-QTRAP-MS/MS) system was performed. The quantitative analysis for MG and CA were approved by the Guidance for Bioanalytical Method Validation issued by Chinese Pharmacopoeia Commission in 2020. The pharmacokinetic parameters were calculated by Drug and Statistic software (Shanghai, China).

1) Collection of blood samples

Male Lewis rats (n=6, 6-8 weeks-old, 200±20g in weight) were purchased from Beijing Vital River Laboratory Animal Technology Co., Ltd. (SCCK 2016-0006, Beijing, China; n=3 per group, including normal control group and normal control+BHGZD treatment group). Diet was prohibited for 12h before the experiment but water was freely available. Blood samples (~0.25mL) were collected from the retinal venous plexus into heparinized 1.5mL polythene tubes before administration and 0, 0.25, 0.5, 1, 2, 3, 4, 6, 8,12 and 24h after administration of BHGZD. The plasma samples were obtained after centrifuging at 12000rpm for 15min, and stored at -20℃ prior to analysis.

2) UHPLC-QTRAP-MS/MS conditions

Quantification analysis were performed on an ultra-high-performance liquid chromatography (Shimadzu Corp., Japan) tandem ion trap quadrupole QTRAP 6500 plus mass spectrometry (AB Sciex, USA). Chromatographic separation was conducted using a Poroshell 120 EC-C18 column (3.0×50mm, 2.7μm) at 40℃. The mobile phase was consisted of 0.1% formic acid–water (*v/v*, A) and methanol (B) and elution program were: 0-0.5min, 20%-40%B; 0.5-1.0min, 40%B; 1.0-3.5min, 40%-90%B; 3.5-5.0min, 90%B; 5.0-5.3min, 90%-20%B; 5.3-8.0min, 20%B.

Mass spectrometry detector were conducted in negative mode and the detecting parameters were set as follows: curtain gas, 35 psi; collision gas, medium; ionspray voltage, 4500 V; temperature, 550℃; ion source gas 1 and 2, 55psi. Quantitative ion pairs were optimized for MG: *m/z* 421.2-301.1 (declustering potential: 63V, CE: 30eV) and for CA: *m/z* 147.0-77.0 (declustering potential:40V, CE: 30eV). Data acquisition and analysis

were performed on OS-Q software (AB Sciex, USA).

Section 14 The culture conditions of Raw264.7 and MH7A cells

Raw264.7 cells were maintained in DMEM (Hyclone, Logan, UT, USA), supplemented with 10% fetal bovine serum (FBS, Gibco, Carlsbad, CA, USA) and 100U/mL penicillin, 100μg/mL streptomycin and 2mM L-glutamine (TBD, Tianjin, China) in a humidified 5% CO_2 incubator at the temperature of 37℃. The Raw264.7 cells at passage numbers four to eight were used in the current experiment validations.

MH7A cells were cultured in sterile synoviocyte growth medium (Cell Applications, Inc., San Diego, CA) supplemented with 10% synoviocyte growth supplement (Cell Applications, Inc., San Diego, CA), 100U/mL penicillin, 100μg/mL streptomycin and 2mM L-glutamine (TBD, Tianjin, China) in a humidified 5% CO_2 incubator at the temperature of 37℃. MH7A at passage numbers four to eight were used in the current experiment validations.

Section 15 NLRP3 inflammasome activation

To induce a conventional NLRP3 inflammasome activation, Raw264.7cells was induced with 0.2μg/mL lipopolysaccharide [LPS, Escherichia coli (O111:B4), Sigma-Aldrich, St Louis, MO, USA] for 4h, followed with a 3mM ATP adenosine triphosphate (ATP disodium salt hydrate, Sigma-Aldrich, St Louis, MO, USA) for 1h of incubation at 37℃ with 5% CO_2 (10).

For MH7A cells LPS/ATP-induced pyrotosis, MH7A cells were induced with 1μg/mL LPS for 6h, followed with a 3mM ATP for 1h of incubation at 37℃ with 5% CO_2 (11).

Section 16 Cell proliferation assay

Cell viability was analyzed with Cell Counting Kit-8 (CCK-8) assay kit (Beyotime Biotechnology, China). Raw264.7 cells were seeded into 96-well plate, and after adherence overnight, we treated with 0.21, 0.42, 0.84, 1.69, 3.37, and 6.74ng/mL MG, as well as 0.02, 0.34, 0.07, 0.13, 0.26, and 0.54ng/mL CA, respective (as the same content of that in 0.62, 1.25, 2.49, 5.02, 10.00, and 20.00μg/mL BHGZD). Then, 10μL of CCK-8 solution was added to each well. The absorbance was measured after 4h of incubation at 37℃ with 5% CO_2.

Section 17 Flow cytometry

To identify and quantify of pyroptosis population of Raw264.7 cells, FITC Apoptosis Detection kit I staining with both the Annexin V-fluorescein isothiocyanate and propidium iodide (PI, BD Biosciences, San Jose, CA, USA) was used in accordance with manufacturer's guidelines. Briefly, Raw264.7 cells was washed twice with cold PBS and then resuspended in 1×binding buffer at a concentration of $1x10^6$ cells/mL. Then, 100μL of the solution ($1x10^5$ cells) was transferred to a 5 mL culture tube. Subsequently, 5μL of FITC Annexin V and 5μL PI were added into cultured tube, and vortexed and incubated for 15 min at room temperature in the dark. Finally, 400μL 1×binding buffer was added into each tube, and analyzed by flow cytometry (ACEA Bioscience, San Diego, CA, USA) and NovoExpress 1.4.1 software (ACEA Bioscience, San Diego, CA, USA) within 1h.

Section 18 Terminal deoxynucleotidyl transferase-mediated dUTP biotin nick end labeling (TUNEL) staining

Cell apoptosis was determined by in situ terminal deoxynucleotidyl transferase-mediated dUTP-biotin nick end-labeling reaction (TUNEL) staining (TUNEL Andy Fluor™ 594 Apoptosis Detection Kit, ABP Biosciences, Wuhan, China) in accordance with the manufacturer's protocol.

Briefly, cells were fixed with 4% formaldehyde in PBA (pH7.4) at 4 ℃ for 30min. Then, they were permeabilized in PBS containing 0.2% Triton X-100 for 30min at room temperature. After incubating with 100μL TdT reaction buffer for 10 min, cells were added 20μL TdT reaction cocktail to each sample. For cell staining, incubate for 60 min at 37℃, protected from light. Subsequently, cells were added 100μL Andy Fluor™ 594-Streptavidin staining solution, and incubated for 30min at room temperature, protected from light. Finally, cells were stained with Hoechst 33342 for 5 min and the images were acquired using an inverted fluorescence microscope (MF53, MSHOT, Guangzhou, China).

Section 19 FAM-FLICA caspase-1 assay

Active caspase-1 was visualized by a FAM-FLICA caspase-1 assay kit using FAM-YVAD-FMK inhibitor probe (ImmunoChemistry Technologies, Bloomington, MN, USA), according to manufacturer's guidelines. In brief, cells were added diluted FLICA to each sample at 1:30 and incubated approximately 1h. Subsequently, they were labelled with Hoechst 33342 staining and the images were analyzed with a Zeiss LSM 880 confocal microscope (Zeiss LSM 880, Carl Zeiss, Jena, Germany).

Section 20 Immunofluorescence staining

Double fluorescence staining was performed as described previously (1). Rabbit anti-NLRP3 (dilution 1:100, ABclonal Technology, Wuhan, China) and mouse anti-ASC (dilution 1:50, Santa Cruz Biotechnology, Santa Cruz, CA, USA) primary antibody were used for NLRP3/ASC double immunofluorescence. Sections were then labeled with FITC- and Cy3-conjugated secondary antibodies (dilution 1:200, Servicebio, Wuhan, China) for 2h at room temperature protecting from light. Subsequently, they were labelled with 4',6-diamidino-2-phenylindole (DAPI) staining solution (Servicebio, Wuhan, China) for 4 min at room temperature, and the images were visualized and photographed with a confocal microscope (Zeiss LSM 880, Carl Zeiss, Jena, Germany) or an inverted fluorescence microscope (MF53, MSHOT, Guangzhou, China).

Table S1 Composition of Baihu-Guizhi Decoction

Pharmaceutical name	Botanical source/family	Medicinal Portions	Traditional actions/uses	Amount (g)
Gypsum	Gypsum Fibrosum	Ore ($CaSO_4 \cdot 2H_2O$)	Clearing away heat and fire, relieving restlessness and thirst, promoting the regeneration of the tissue and wound healing	60
Anemarrhena asphodeloides Bge.	*Anemarrhema asphodeloides* Bge.	Dried rhizome	Clearing away heat and fire, nourishing the Yin and moisturizing dryness	15
Cinnamomum cassia Presl	*Cinnamomum cassia* Preal	Dried shoot	Inducing sweating to dispel exopathogens, warming the channels to clear obstruction, and reinforcing Yang to promote qi	10
Oryza sativa L.	Oryza sativa	Dried kernel	Reinforcing spleen, regulating stomach, and clearing away the lung-heat	30

Glycyrrhiza uralensis Fisch	*Glycytthiza uralensis* Fisch., *Glycytthiza inflate* Bat.,and *Glycytthiza glabra* L.	Dried root and rhizome	Tonifying the heart and spleen, moistening the lung to arrest cough, purging fire to remove toxins, relieving spasm to alleviate pain and harmonizing all drugs	5

Table S2 The detailed information on reference standards

Chemical name	Cas No.	Source	Lot No.	Purity
Timosaponin BⅡ	136656-07-0	Chengdu Must Bio-technology Co., Ltd (Chendu, China)	MUST-18052605	HPLC≥98%
Mangiferin	4773-96-0	The National Institute for the Control of Pharmaceutical and Biological Products (Beijing, China)	11607-201704	HPLC≥98.1%
Glycyrrhizic acid	1405-86-3	Chengdu Must Bio-technology Co., Ltd (Chendu, China)	MUST-18060805	HPLC≥98%
Neomangiferin	64809-67-2	Chengdu Must Bio-technology Co., Ltd (Chendu, China)	MUST-18032315	HPLC≥98%
7-O-methyl mangiferin	31002-12-7	Chengdu Must Bio-technology Co., Ltd (Chendu, China)	MUST-17101202	HPLC≥99.8%
Anemarrhensaponin Ⅰ	163047-21-0	Chengdu Must Bio-technology Co., Ltd (Chendu, China)	MUST-18032402	HPLC≥98%
Vitexin	3681-93-4	Chengdu Must Bio-technology Co., Ltd (Chendu, China)	A0358	HPLC≥98%
Formononetin	485-72-3	The National Institute for the Control of Pharmaceutical and Biological Products (Beijing, China)	111703-201504	HPLC≥98%

Liquiritigenin	578-86-9	Chengdu DeSiTe Biological Technology Co.,Ltd (Chendu, China)	DST180719-086	HPLC≥98%
Isoliquiritin	5041-81-6	Chengdu DeSiTe Biological Technology Co., Ltd (Chendu, China)	DST180314-015	HPLC≥98%
Cinnamic acid	621-82-9	The National Institute for the Control of Pharmaceutical and Biological Products (Beijing, China)	110786-201604	HPLC≥98.8%

Table S3 The antibodies used for western blotting analysis

Antibody	Cat No.	Manufacturers	Host	Description	Specificity	Molecular Weight	Dilution
TLR4	bs-20594R	Bioss Antibodies	Rabbit	Rabbit Anti-TLR4 Antibody	Mouse, Rat, Pig, Cow, Horse, Rabbit, Sheep	90/120kDa	1/1000
NLRP3	A5652	ABclonal	Rabbit	NLRP3 Rabbit pAb	Human, Mouse, Rat	110kDa	1/1000
p-NFκB-p65	YP0191	ImmunoWay Biotechnology Company	Rabbit	NFκB-p65 (phospho Ser536) Polyclonal Antibody	Human, Mouse, Rat, Monkey	60kDa	1/1000
NFκB-p65	sc-8008	Santa Cruz	Mouse	NFκB p65 Antibody (F-6)	Mouse, Rat, Human	65kDa	1/1000
caspase-1	3866	Cell Signaling Technology	Rabbit	caspase-1 (D7F10) rabbit antibody	Mouse, Rat, Human	48/20kDa	1/1000

caspase-1	ab179515	Abcam	Rabbit	Rabbit Monoclonal (EPR16883) to pro Caspase-1+p10+p12	Mouse, Rat, Human	45/42/35/12/10kDa	1/1000
IL-1β	A16288	ABclonal	Rabbit	IL1 beta Rabbit pAb	Human, Mouse, Rat	17kDa/35kDa	1/1000
PI3K	4249	Cell Signaling Technology	Rabbit	PI3 Kinase p110α (C73F8) Rabbit mAb	Human, Mouse, Rat, Cow	110kDa	1/1000
p-PI3K	bs-3332R	Bioss Antibodies	Rabbit	Rabbit Anti-phospho-PI3 kinase p85 alpha + gamma (Tyr467+Tyr199) Antibody	Human, Mouse, Rat	85/55kDa	1/1000
AKT1	ab89402	Abcam	Mouse	Anti-AKT1 Antibody [9A4]	Mouse, Human	56kDa	1/1000
p-AKT1	ab81283	Abcam	Rabbit	Recombinant Anti-AKT1 (phospho S473) Antibody [EP2109Y]	Mouse, Rat, Human	56kDa	1/1000
GSDMD	ab215191	Abcam	Rabbit	Recombinant Anti-DFNA5/GSDME Antibody [EPR19859]-N-terminal	Mouse, Human	55kDa	1/1000
ASC	sc-514414	Santa Cruz	Mouse	ASC Antibody (B-3)	Mouse, Rat, Human	24kDa	1/1000

*β-actin	BM0627	Boster Biological Technology	Mouse	Anti-beta-Actin/β-Actin Antibody	Human, Mouse, Rat	42kDa	1/1000
#GAPDH	ab8245	Abcam	Mouse	GAPDH Mouse Monoclonal Antibody	Mouse, Rat, Human	37kDa	1/1000

* As loading control in cultured cells sample; # As loading control in arthritic tissue sample.

Table S4 The significant differentially expressed genes (DEGs) identified by microarray analysis

Groups	Symbol	P-value	Foldchange	Regulation
AIA-M *vs.* Con	ABLIM2	0.0314	0.1894	down
AIA-M *vs.* Con	ACKR4	0.0389	0.3704	down
AIA-M *vs.* Con	ACTRT2	0.0273	0.0177	down
AIA-M *vs.* Con	AQP3	0.0419	0.0395	down
AIA-M *vs.* Con	ARC	0.0437	0.4174	down
AIA-M *vs.* Con	ARSI	0.0157	2.2335	up
AIA-M *vs.* Con	ATP2B2	0.0260	0.0152	down
AIA-M *vs.* Con	C3	0.0197	9.3385	up
AIA-M *vs.* Con	C7	0.0046	2.0019	up
AIA-M *vs.* Con	CACNA1S	0.0199	0.1810	down
AIA-M *vs.* Con	CAR13	0.0411	2.5659	up
AIA-M *vs.* Con	CD3G	0.0113	4.0476	up
AIA-M *vs.* Con	CD6	0.0118	3.0542	up
AIA-M *vs.* Con	CD8A	0.0386	2.4200	up
AIA-M *vs.* Con	CFB	0.0087	5.0173	up
AIA-M *vs.* Con	CHAC1	0.0308	2.3841	up
AIA-M *vs.* Con	CHL1	0.0266	2.4939	up
AIA-M *vs.* Con	CMAH	0.0476	3.9053	up
AIA-M *vs.* Con	COL1A1	0.0390	3.5456	up
AIA-M *vs.* Con	CUBN	0.0440	0.3533	down
AIA-M *vs.* Con	CYP26A1	0.0410	2.3796	up
AIA-M *vs.* Con	CYP2W1	0.0302	3.0920	up
AIA-M *vs.* Con	EGLN3	0.0224	0.3302	down

AIA-M *vs.* Con	EPHA7	0.0300	7.8100	up
AIA-M *vs.* Con	EXPH5	0.0254	0.4274	down
AIA-M *vs.* Con	FKBP5	0.0114	2.8834	up
AIA-M *vs.* Con	FOXN1	0.0158	2.0873	up
AIA-M *vs.* Con	GAS2	0.0374	9.0614	up
AIA-M *vs.* Con	HHIP	0.0135	0.3201	down
AIA-M *vs.* Con	ID4	0.0244	0.3266	down
AIA-M *vs.* Con	IFIT1LB	0.0089	3.2677	up
AIA-M *vs.* Con	IL1B	0.0121	4.4585	up
AIA-M *vs.* Con	IRAK2	0.0264	0.0374	down
AIA-M *vs.* Con	ITK	0.0278	6.5331	up
AIA-M *vs.* Con	KRT79	0.0403	0.3235	down
AIA-M *vs.* Con	LOC100361018	0.0252	0.3629	down
AIA-M *vs.* Con	LOC100364265	0.0423	2.9746	up
AIA-M *vs.* Con	LOC102556269	0.0399	2.2409	up
AIA-M *vs.* Con	LOC681325	0.0421	2.8642	up
AIA-M *vs.* Con	MAP2	0.0094	0.2879	down
AIA-M *vs.* Con	MARCKS	0.0360	2.1929	up
AIA-M *vs.* Con	MNDA	0.0268	11.8457	up
AIA-M *vs.* Con	NAPSA	0.0398	2.8142	up
AIA-M *vs.* Con	NCKAP5	0.0403	0.4702	down
AIA-M *vs.* Con	NKG7	0.0184	3.9782	up
AIA-M *vs.* Con	NRAP	0.0015	0.2919	down
AIA-M *vs.* Con	NRBP2	0.0020	2.1317	up
AIA-M *vs.* Con	OLR322	0.0466	0.0440	down
AIA-M *vs.* Con	PADI4	0.0321	4.1635	up
AIA-M *vs.* Con	PCDH15	0.0458	0.0293	down

AIA-M *vs.* Con	PHF11B	0.0241	2.9681	up
AIA-M *vs.* Con	PIK3CD	0.0214	2.2368	up
AIA-M *vs.* Con	PKHD1L1	0.0199	0.3226	down
AIA-M *vs.* Con	PLAC9	0.0322	0.4364	down
AIA-M *vs.* Con	PLAGL1	0.0099	2.4027	up
AIA-M *vs.* Con	PLXDC1	0.0114	0.4035	down
AIA-M *vs.* Con	PLXNC1	0.0438	3.5885	up
AIA-M *vs.* Con	PRF1	0.0465	3.7938	up
AIA-M *vs.* Con	RERG	0.0352	0.3471	down
AIA-M *vs.* Con	RET	0.0365	6.0356	up
AIA-M *vs.* Con	RGD1359108	0.0101	0.2924	down
AIA-M *vs.* Con	RGD1561841	0.0076	2.3339	up
AIA-M *vs.* Con	RGD1563194	0.0490	2.0764	up
AIA-M *vs.* Con	RGD1563636	0.0379	2.0667	up
AIA-M *vs.* Con	RPS26	0.0402	2.6209	up
AIA-M *vs.* Con	S100A8	0.0179	8.7199	up
AIA-M *vs.* Con	S100A9	0.0085	8.1027	up
AIA-M *vs.* Con	SCD	0.0027	0.2437	down
AIA-M *vs.* Con	SCG5	0.0397	0.4742	down
AIA-M *vs.* Con	SERPINA3N	0.0451	2.3516	up
AIA-M *vs.* Con	SIT1	0.0328	6.1876	up
AIA-M *vs.* Con	SKA1	0.0235	2.3227	up
AIA-M *vs.* Con	SLURP1	0.0017	0.3149	down
AIA-M *vs.* Con	STC2	0.0392	0.3961	down
AIA-M *vs.* Con	TBC1D10C	0.0137	3.3673	up
AIA-M *vs.* Con	TENM3	0.0346	5.9259	up
AIA-M *vs.* Con	TNFRSF14	0.0146	10.3978	up

AIA-M *vs.* Con	TNMD	0.0418	10.2952	up
AIA-M *vs.* Con	TUBB3	0.0089	2.9806	up
AIA-M *vs.* Con	UBD	0.0133	10.2848	up
AIA-M *vs.* Con	UNC5CL	0.0172	0.2765	down
AIA-M *vs.* Con	UTS2R	0.0234	12.7936	up
AIA-M *vs.* Con	VSNL1	0.0251	0.4442	down
AIA-M *vs.* Con	WDR76	0.0151	0.1236	down
AIA-M *vs.* Con	WSCD1	0.0267	0.3755	down
AIA-M *vs.* Con	ZC3HAV1L	0.0259	3.3161	up
BHGZD *vs.* AIA-M	AACS	0.0420	2.0649	Up
BHGZD *vs.* AIA-M	ABCC8	0.0469	4.1955	Up
BHGZD *vs.* AIA-M	ABCG1	0.0027	2.4178	Up
BHGZD *vs.* AIA-M	ABCG2	0.0033	4.4883	Up
BHGZD *vs.* AIA-M	ABCG3L4	0.0458	0.4781	Down
BHGZD *vs.* AIA-M	ACHE	0.0038	0.4856	Down
BHGZD *vs.* AIA-M	ACIN1	0.0036	0.4096	Down
BHGZD *vs.* AIA-M	ACKR4	0.0011	2.1956	Up
BHGZD *vs.* AIA-M	ACR	0.0415	0.4025	Down
BHGZD *vs.* AIA-M	ACSM5	0.0482	8.8784	Up
BHGZD *vs.* AIA-M	ACVR2B	0.0458	2.1767	Up
BHGZD *vs.* AIA-M	ADAP1	0.0228	2.5009	Up
BHGZD *vs.* AIA-M	ADCY5	0.0021	0.3591	Down
BHGZD *vs.* AIA-M	ADCY7	0.0122	0.4698	Down
BHGZD *vs.* AIA-M	ADORA3	0.0208	0.1566	Down
BHGZD *vs.* AIA-M	ADRA1B	0.0029	3.1109	Up
BHGZD *vs.* AIA-M	AFAP1L1	0.0087	2.6193	Up
BHGZD *vs.* AIA-M	AFAP1L2	0.0076	3.0280	Up

BHGZD *vs.* AIA-M	AFF2	0.0104	0.2968	Down
BHGZD *vs.* AIA-M	AGRP	0.0000	2.0484	Up
BHGZD *vs.* AIA-M	AIG1	0.0183	2.0030	Up
BHGZD *vs.* AIA-M	AKAP1	0.0076	2.1160	Up
BHGZD *vs.* AIA-M	AKAP12	0.0369	2.0229	Up
BHGZD *vs.* AIA-M	ALG8	0.0345	2.0937	Up
BHGZD *vs.* AIA-M	ALG9	0.0389	2.5043	Up
BHGZD *vs.* AIA-M	ALOXE3	0.0049	2.1676	Up
BHGZD *vs.* AIA-M	ALS2	0.0455	2.4480	Up
BHGZD *vs.* AIA-M	AMY1A	0.0162	4.7174	Up
BHGZD *vs.* AIA-M	ANGPT1	0.0150	0.3922	Down
BHGZD *vs.* AIA-M	ANKRD1	0.0103	2.3998	Up
BHGZD *vs.* AIA-M	ANKRD17	0.0025	0.3725	Down
BHGZD *vs.* AIA-M	ANKRD33B	0.0049	2.6067	Up
BHGZD *vs.* AIA-M	ANKRD55	0.0065	2.2078	Up
BHGZD *vs.* AIA-M	AOC2-PS1	0.0130	3.5339	Up
BHGZD *vs.* AIA-M	AP3D1	0.0002	0.4907	Down
BHGZD *vs.* AIA-M	AP5Z1	0.0127	0.4092	Down
BHGZD *vs.* AIA-M	APOOL	0.0495	2.0187	Up
BHGZD *vs.* AIA-M	AQP2	0.0052	0.2274	Down
BHGZD *vs.* AIA-M	AQP3	0.0003	24.4447	Up
BHGZD *vs.* AIA-M	ARHGAP30	0.0130	0.3442	Down
BHGZD *vs.* AIA-M	ARHGAP44	0.0106	0.4718	Down
BHGZD *vs.* AIA-M	ARHGEF37	0.0238	2.5520	Up
BHGZD *vs.* AIA-M	ARSI	0.0043	0.2668	Down
BHGZD *vs.* AIA-M	ARSJ	0.0373	0.4336	Down
BHGZD *vs.* AIA-M	ART1	0.0075	0.1971	Down

BHGZD *vs.* AIA-M	ASCC3	0.0181	0.3358	Down
BHGZD *vs.* AIA-M	ASF1B	0.0175	0.1314	Down
BHGZD *vs.* AIA-M	ASIP	0.0009	0.0831	Down
BHGZD *vs.* AIA-M	ASPH	0.0085	0.4026	Down
BHGZD *vs.* AIA-M	ASS1	0.0242	2.3491	Up
BHGZD *vs.* AIA-M	ATAD2	0.0105	0.4846	Down
BHGZD *vs.* AIA-M	ATMIN	0.0006	2.0549	Up
BHGZD *vs.* AIA-M	ATP1B2	0.0404	3.4896	Up
BHGZD *vs.* AIA-M	ATP5HL1	0.0189	0.4710	Down
BHGZD *vs.* AIA-M	ATXN1	0.0164	0.4638	Down
BHGZD *vs.* AIA-M	BATF2	0.0450	0.4370	Down
BHGZD *vs.* AIA-M	BCAN	0.0334	0.2133	Down
BHGZD *vs.* AIA-M	BCAS1	0.0049	2.4368	Up
BHGZD *vs.* AIA-M	BCL2L15	0.0174	6.7447	Up
BHGZD *vs.* AIA-M	BMP4	0.0054	2.5915	Up
BHGZD *vs.* AIA-M	BMP6	0.0087	2.6252	Up
BHGZD *vs.* AIA-M	BNIPL	0.0102	0.4762	Down
BHGZD *vs.* AIA-M	BRD1	0.0119	0.3592	Down
BHGZD *vs.* AIA-M	C2CD4B	0.0046	0.1731	Down
BHGZD *vs.* AIA-M	C3AR1	0.0148	0.4868	Down
BHGZD *vs.* AIA-M	C7	0.0142	0.4326	Down
BHGZD *vs.* AIA-M	CA1	0.0498	5.3994	Up
BHGZD *vs.* AIA-M	CADM4	0.0050	0.3239	Down
BHGZD *vs.* AIA-M	CALCA	0.0022	0.4193	Down
BHGZD *vs.* AIA-M	CALCOCO2	0.0237	0.2221	Down
BHGZD *vs.* AIA-M	CALU	0.0405	0.4266	Down
BHGZD *vs.* AIA-M	CAMP	0.0427	22.2667	Up

BHGZD *vs.* AIA-M	CAMSAP2	0.0003	0.4937	Down
BHGZD *vs.* AIA-M	CAR13	0.0136	0.2647	Down
BHGZD *vs.* AIA-M	CBFA2T3	0.0038	2.0248	Up
BHGZD *vs.* AIA-M	CBLN1	0.0268	0.2227	Down
BHGZD *vs.* AIA-M	CCDC120	0.0014	0.4876	Down
BHGZD *vs.* AIA-M	CCDC18	0.0178	3.8304	Up
BHGZD *vs.* AIA-M	CCER1	0.0318	0.4680	Down
BHGZD *vs.* AIA-M	CCK	0.0009	0.2685	Down
BHGZD *vs.* AIA-M	CCNO	0.0014	0.3250	Down
BHGZD *vs.* AIA-M	CCT8L1	0.0398	2.1465	Up
BHGZD *vs.* AIA-M	CD177	0.0201	25.6675	Up
BHGZD *vs.* AIA-M	CD200R1L	0.0251	3.6960	Up
BHGZD *vs.* AIA-M	CD3D	0.0341	0.4932	Down
BHGZD *vs.* AIA-M	CD7	0.0022	0.4302	Down
BHGZD *vs.* AIA-M	CD79B	0.0135	12.5255	Up
BHGZD *vs.* AIA-M	CDC42BPA	0.0142	2.5970	Up
BHGZD *vs.* AIA-M	CDH19	0.0053	3.1988	Up
BHGZD *vs.* AIA-M	CDK14	0.0151	0.4757	Down
BHGZD *vs.* AIA-M	CDKL4	0.0001	2.2403	Up
BHGZD *vs.* AIA-M	CDS1	0.0098	2.6792	Up
BHGZD *vs.* AIA-M	CDT1	0.0038	5.8794	Up
BHGZD *vs.* AIA-M	CEPT1	0.0207	2.0075	Up
BHGZD *vs.* AIA-M	CES2A	0.0017	0.3715	Down
BHGZD *vs.* AIA-M	CFB	0.0100	0.2653	Down
BHGZD *vs.* AIA-M	CFI	0.0370	0.0520	Down
BHGZD *vs.* AIA-M	CHCHD10	0.0488	4.0023	Up
BHGZD *vs.* AIA-M	CHD1L	0.0126	3.2825	Up

BHGZD *vs.* AIA-M	CHD9	0.0167	0.4806	Down
BHGZD *vs.* AIA-M	CHIT1	0.0454	3.3368	Up
BHGZD *vs.* AIA-M	CHL1	0.0082	0.3051	Down
BHGZD *vs.* AIA-M	CHODL	0.0181	3.2106	Up
BHGZD *vs.* AIA-M	CHRNA1	0.0138	5.4908	Up
BHGZD *vs.* AIA-M	CHRNB1	0.0033	5.2704	Up
BHGZD *vs.* AIA-M	CILP2	0.0238	0.0283	Down
BHGZD *vs.* AIA-M	CISH	0.0404	2.3668	Up
BHGZD *vs.* AIA-M	CITED1	0.0352	2.5536	Up
BHGZD *vs.* AIA-M	CLDN1	0.0045	2.1085	Up
BHGZD *vs.* AIA-M	CLDN19	0.0380	2.4652	Up
BHGZD *vs.* AIA-M	CLDN23	0.0014	0.2124	Down
BHGZD *vs.* AIA-M	CLDN3	0.0010	0.3343	Down
BHGZD *vs.* AIA-M	CLEC3A	0.0049	11.2914	Up
BHGZD *vs.* AIA-M	CLN3	0.0404	2.4998	Up
BHGZD *vs.* AIA-M	CML5	0.0495	2.0109	Up
BHGZD *vs.* AIA-M	CMTM4	0.0033	2.5920	Up
BHGZD *vs.* AIA-M	COL11A1	0.0471	0.0516	Down
BHGZD *vs.* AIA-M	COL11A2	0.0434	0.3066	Down
BHGZD *vs.* AIA-M	COL28A1	0.0029	3.3414	Up
BHGZD *vs.* AIA-M	COL2A1	0.0410	13.0263	Up
BHGZD *vs.* AIA-M	COL9A1	0.0253	0.1424	Down
BHGZD *vs.* AIA-M	CORO2A	0.0092	2.9709	Up
BHGZD *vs.* AIA-M	CPA6	0.0476	2.9989	Up
BHGZD *vs.* AIA-M	CPLX2	0.0145	2.4911	Up
BHGZD *vs.* AIA-M	CRB3	0.0217	4.4336	Up
BHGZD *vs.* AIA-M	CREB3L1	0.0014	0.3957	Down

BHGZD *vs.* AIA-M	CRYGD	0.0036	0.3783	Down
BHGZD *vs.* AIA-M	CSRNP2	0.0185	0.4489	Down
BHGZD *vs.* AIA-M	CTDSPL	0.0440	2.2062	Up
BHGZD *vs.* AIA-M	CTNND2	0.0216	2.1167	Up
BHGZD *vs.* AIA-M	CTSG	0.0108	61.1718	Up
BHGZD *vs.* AIA-M	CTSLL3	0.0207	0.4663	Down
BHGZD *vs.* AIA-M	CUBN	0.0272	4.0319	Up
BHGZD *vs.* AIA-M	CYP26A1	0.0032	0.3338	Down
BHGZD *vs.* AIA-M	CYP26B1	0.0051	3.2568	Up
BHGZD *vs.* AIA-M	CYP2E1	0.0121	13.2940	Up
BHGZD *vs.* AIA-M	CYP2W1	0.0320	0.1967	Down
BHGZD *vs.* AIA-M	DCAF5	0.0393	0.2492	Down
BHGZD *vs.* AIA-M	DCAF6	0.0088	0.3410	Down
BHGZD *vs.* AIA-M	DCLK3	0.0273	2.2365	Up
BHGZD *vs.* AIA-M	DDB2	0.0010	2.0550	Up
BHGZD *vs.* AIA-M	DEFA5	0.0314	31.7178	Up
BHGZD *vs.* AIA-M	DEFB22	0.0165	0.3771	Down
BHGZD *vs.* AIA-M	DEGS2	0.0315	11.0218	Up
BHGZD *vs.* AIA-M	DERL3	0.0328	6.1441	Up
BHGZD *vs.* AIA-M	DET1	0.0043	2.3127	Up
BHGZD *vs.* AIA-M	DIO3	0.0315	0.4337	Down
BHGZD *vs.* AIA-M	DMXL2	0.0046	2.2822	Up
BHGZD *vs.* AIA-M	DNAJC6	0.0191	0.4396	Down
BHGZD *vs.* AIA-M	DNHD1-PS1	0.0032	2.0913	Up
BHGZD *vs.* AIA-M	DNM1L	0.0003	0.4528	Down
BHGZD *vs.* AIA-M	DOCK2	0.0300	0.4374	Down
BHGZD *vs.* AIA-M	DPYD	0.0291	3.0118	Up

BHGZD *vs.* AIA-M	DUSP8	0.0204	0.1786	Down
BHGZD *vs.* AIA-M	DYRK1B	0.0435	0.3234	Down
BHGZD *vs.* AIA-M	E4F1	0.0434	2.1545	Up
BHGZD *vs.* AIA-M	ECH1	0.0389	2.5236	Up
BHGZD *vs.* AIA-M	EDA	0.0262	0.3909	Down
BHGZD *vs.* AIA-M	EDN1	0.0228	2.2404	Up
BHGZD *vs.* AIA-M	EFCAB12	0.0458	3.8189	Up
BHGZD *vs.* AIA-M	EFCAB4A	0.0352	2.3815	Up
BHGZD *vs.* AIA-M	EFHD1	0.0231	2.6581	Up
BHGZD *vs.* AIA-M	EFR3B	0.0099	2.7331	Up
BHGZD *vs.* AIA-M	EGLN3	0.0216	2.6592	Up
BHGZD *vs.* AIA-M	EIF5A2	0.0006	0.4062	Down
BHGZD *vs.* AIA-M	ELANE	0.0034	40.3800	Up
BHGZD *vs.* AIA-M	EMX2	0.0457	0.4756	Down
BHGZD *vs.* AIA-M	ENO4	0.0371	2.8538	Up
BHGZD *vs.* AIA-M	ENPP6	0.0222	2.8345	Up
BHGZD *vs.* AIA-M	EPHB3	0.0430	0.3579	Down
BHGZD *vs.* AIA-M	EPHX1	0.0409	3.0003	Up
BHGZD *vs.* AIA-M	ERAS	0.0032	3.4764	Up
BHGZD *vs.* AIA-M	ERGIC2	0.0282	0.2718	Down
BHGZD *vs.* AIA-M	ERP27	0.0355	4.8229	Up
BHGZD *vs.* AIA-M	ETV3	0.0010	0.1127	Down
BHGZD *vs.* AIA-M	EXPH5	0.0204	3.2304	Up
BHGZD *vs.* AIA-M	FAM114A1	0.0118	0.2573	Down
BHGZD *vs.* AIA-M	FAM114A1L1	0.0246	0.3412	Down
BHGZD *vs.* AIA-M	FAM195B	0.0052	0.4199	Down
BHGZD *vs.* AIA-M	FAM212A	0.0131	2.9141	Up

BHGZD *vs.* AIA-M	FAM214A	0.0271	2.2309	Up
BHGZD *vs.* AIA-M	FAM221A	0.0251	3.5070	Up
BHGZD *vs.* AIA-M	FAM63B	0.0330	2.0027	Up
BHGZD *vs.* AIA-M	FAM83H	0.0003	7.5046	Up
BHGZD *vs.* AIA-M	FBN1	0.0299	0.3944	Down
BHGZD *vs.* AIA-M	FBP1	0.0051	0.3027	Down
BHGZD *vs.* AIA-M	FBRSL1	0.0440	0.2479	Down
BHGZD *vs.* AIA-M	FBXO3	0.0160	2.8668	Up
BHGZD *vs.* AIA-M	FBXO41	0.0001	0.4450	Down
BHGZD *vs.* AIA-M	FES	0.0029	2.8656	Up
BHGZD *vs.* AIA-M	FGFR3	0.0034	0.2075	Down
BHGZD *vs.* AIA-M	FHL5	0.0408	2.2809	Up
BHGZD *vs.* AIA-M	FIZ1	0.0042	3.0234	Up
BHGZD *vs.* AIA-M	FMO1	0.0243	4.5763	Up
BHGZD *vs.* AIA-M	FMO2	0.0198	3.9013	Up
BHGZD *vs.* AIA-M	FMOD	0.0200	0.1050	Down
BHGZD *vs.* AIA-M	FNDC8	0.0230	0.3472	Down
BHGZD *vs.* AIA-M	FOSB	0.0117	0.2191	Down
BHGZD *vs.* AIA-M	FOXC2	0.0085	0.4409	Down
BHGZD *vs.* AIA-M	FOXD4	0.0138	0.3323	Down
BHGZD *vs.* AIA-M	FOXE1	0.0002	0.2849	Down
BHGZD *vs.* AIA-M	FRY	0.0481	2.1531	Up
BHGZD *vs.* AIA-M	FRZB	0.0002	2.0461	Up
BHGZD *vs.* AIA-M	FST	0.0234	2.8832	Up
BHGZD *vs.* AIA-M	FUBP1	0.0002	0.4689	Down
BHGZD *vs.* AIA-M	GABRQ	0.0109	0.3230	Down
BHGZD *vs.* AIA-M	GABRR2	0.0352	2.3828	Up

BHGZD *vs.* AIA-M	GALNT15	0.0252	2.0482	Up
BHGZD *vs.* AIA-M	GAS2	0.0478	0.4166	Down
BHGZD *vs.* AIA-M	GAST	0.0140	0.4536	Down
BHGZD *vs.* AIA-M	GATM	0.0021	2.0118	Up
BHGZD *vs.* AIA-M	GBF1	0.0131	0.4187	Down
BHGZD *vs.* AIA-M	GBP1	0.0323	0.3213	Down
BHGZD *vs.* AIA-M	GBP5	0.0475	0.4970	Down
BHGZD *vs.* AIA-M	GDNF	0.0046	0.4219	Down
BHGZD *vs.* AIA-M	GIPR	0.0184	2.5706	Up
BHGZD *vs.* AIA-M	GKN3	0.0330	7.6944	Up
BHGZD *vs.* AIA-M	GLS	0.0158	0.4916	Down
BHGZD *vs.* AIA-M	GLYCTK	0.0457	2.9514	Up
BHGZD *vs.* AIA-M	GNL3L	0.0000	0.4296	Down
BHGZD *vs.* AIA-M	GOLGA3	0.0202	0.4566	Down
BHGZD *vs.* AIA-M	GOT1L1	0.0115	7.4215	Up
BHGZD *vs.* AIA-M	GP1BA	0.0169	0.4026	Down
BHGZD *vs.* AIA-M	GPC3	0.0093	2.5380	Up
BHGZD *vs.* AIA-M	GPM6A	0.0172	6.5083	Up
BHGZD *vs.* AIA-M	GPR160	0.0445	2.4305	Up
BHGZD *vs.* AIA-M	GPR18	0.0337	2.4722	Up
BHGZD *vs.* AIA-M	GPR19	0.0418	3.8369	Up
BHGZD *vs.* AIA-M	GPR37L1	0.0428	4.0642	Up
BHGZD *vs.* AIA-M	GPRIN1	0.0116	0.2939	Down
BHGZD *vs.* AIA-M	GPSM1	0.0017	0.1818	Down
BHGZD *vs.* AIA-M	GPT2	0.0078	4.1682	Up
BHGZD *vs.* AIA-M	GPX8	0.0412	0.4292	Down
BHGZD *vs.* AIA-M	GRHL1	0.0225	2.4351	Up

BHGZD *vs.* AIA-M	GRIA3	0.0149	0.3570	Down
BHGZD *vs.* AIA-M	GRIN2D	0.0135	0.1066	Down
BHGZD *vs.* AIA-M	GRM2	0.0059	0.4723	Down
BHGZD *vs.* AIA-M	GRM3	0.0003	0.3983	Down
BHGZD *vs.* AIA-M	GULO	0.0052	10.3961	Up
BHGZD *vs.* AIA-M	GYLTL1B	0.0024	0.3108	Down
BHGZD *vs.* AIA-M	HAPLN4	0.0161	0.4897	Down
BHGZD *vs.* AIA-M	HCN4	0.0109	0.3192	Down
BHGZD *vs.* AIA-M	HDC	0.0069	2.1009	Up
BHGZD *vs.* AIA-M	HERC3	0.0332	2.7606	Up
BHGZD *vs.* AIA-M	HES7	0.0003	0.2000	Down
BHGZD *vs.* AIA-M	HHIP	0.0307	2.6551	Up
BHGZD *vs.* AIA-M	HIST1H2BD	0.0183	0.4561	Down
BHGZD *vs.* AIA-M	HIVEP3	0.0276	0.4063	Down
BHGZD *vs.* AIA-M	HMGCS2	0.0008	7.2208	Up
BHGZD *vs.* AIA-M	HNRNPH2	0.0018	0.4961	Down
BHGZD *vs.* AIA-M	HORMAD2	0.0425	2.7430	Up
BHGZD *vs.* AIA-M	HOXA2	0.0318	2.3086	Up
BHGZD *vs.* AIA-M	HPDL	0.0394	3.0256	Up
BHGZD *vs.* AIA-M	HRK	0.0484	3.5924	Up
BHGZD *vs.* AIA-M	HS2ST1	0.0026	4.8195	Up
BHGZD *vs.* AIA-M	HS3ST1	0.0046	2.1463	Up
BHGZD *vs.* AIA-M	HSD17B13	0.0078	2.4612	Up
BHGZD *vs.* AIA-M	HSP90B1	0.0332	0.3565	Down
BHGZD *vs.* AIA-M	HSPA12A	0.0006	2.1937	Up
BHGZD *vs.* AIA-M	HSPA4	0.0202	0.2225	Down
BHGZD *vs.* AIA-M	HSPB7	0.0032	2.2063	Up

BHGZD *vs.* AIA-M	HTR3B	0.0322	0.3268	Down
BHGZD *vs.* AIA-M	ID4	0.0089	2.4937	Up
BHGZD *vs.* AIA-M	IFITM5	0.0411	3.0939	Up
BHGZD *vs.* AIA-M	IFITM6	0.0188	10.0514	Up
BHGZD *vs.* AIA-M	IGFBP1	0.0007	0.4857	Down
BHGZD *vs.* AIA-M	IGFBP3	0.0013	2.0178	Up
BHGZD *vs.* AIA-M	IGFBP5	0.0167	0.4013	Down
BHGZD *vs.* AIA-M	IL12A	0.0009	9.0202	Up
BHGZD *vs.* AIA-M	IL1R2	0.0158	2.2287	Up
BHGZD *vs.* AIA-M	IL2RA	0.0169	6.6070	Up
BHGZD *vs.* AIA-M	ILVBL	0.0433	2.0790	Up
BHGZD *vs.* AIA-M	IMPACT	0.0470	2.1961	Up
BHGZD *vs.* AIA-M	INHBB	0.0040	0.2756	Down
BHGZD *vs.* AIA-M	INPP4A	0.0076	2.1110	Up
BHGZD *vs.* AIA-M	IQCF1	0.0021	0.3203	Down
BHGZD *vs.* AIA-M	IQUB	0.0077	2.5733	Up
BHGZD *vs.* AIA-M	IRS1	0.0263	0.4812	Down
BHGZD *vs.* AIA-M	ITSN1	0.0102	3.9862	Up
BHGZD *vs.* AIA-M	IVNS1ABP	0.0098	2.1596	Up
BHGZD *vs.* AIA-M	JPH1	0.0365	0.3936	Down
BHGZD *vs.* AIA-M	JPH3	0.0222	0.2022	Down
BHGZD *vs.* AIA-M	KALRN	0.0251	2.4222	Up
BHGZD *vs.* AIA-M	KCNA1	0.0139	2.5517	Up
BHGZD *vs.* AIA-M	KCNG1	0.0438	0.3453	Down
BHGZD *vs.* AIA-M	KCNG2	0.0007	0.3515	Down
BHGZD *vs.* AIA-M	KCNIP3	0.0095	0.3152	Down
BHGZD *vs.* AIA-M	KCNJ14	0.0140	3.0219	Up

BHGZD *vs.* AIA-M	KCNMB4	0.0345	2.0004	Up
BHGZD *vs.* AIA-M	KDM8	0.0265	0.4231	Down
BHGZD *vs.* AIA-M	KERA	0.0061	0.0303	Down
BHGZD *vs.* AIA-M	KIF1B	0.0415	0.4430	Down
BHGZD *vs.* AIA-M	KIF21A	0.0185	2.0443	Up
BHGZD *vs.* AIA-M	KIT	0.0031	5.2042	Up
BHGZD *vs.* AIA-M	KLF5	0.0038	2.4390	Up
BHGZD *vs.* AIA-M	KLHL21	0.0464	2.7108	Up
BHGZD *vs.* AIA-M	KLHL26	0.0346	3.4262	Up
BHGZD *vs.* AIA-M	KLK5L	0.0166	0.3200	Down
BHGZD *vs.* AIA-M	KLRA5	0.0415	0.3271	Down
BHGZD *vs.* AIA-M	KLRC2	0.0112	0.4492	Down
BHGZD *vs.* AIA-M	KRT13	0.0118	2.3269	Up
BHGZD *vs.* AIA-M	LDHC	0.0063	13.5105	Up
BHGZD *vs.* AIA-M	LEKR1	0.0272	0.4488	Down
BHGZD *vs.* AIA-M	LEPR	0.0018	2.2323	Up
BHGZD *vs.* AIA-M	LIN28A	0.0422	0.4847	Down
BHGZD *vs.* AIA-M	LIX1	0.0143	13.2468	Up
BHGZD *vs.* AIA-M	LOXL2	0.0085	0.4491	Down
BHGZD *vs.* AIA-M	LPCAT4	0.0374	2.2411	Up
BHGZD *vs.* AIA-M	LPPR3	0.0008	0.1284	Down
BHGZD *vs.* AIA-M	LRRC58	0.0113	2.3397	Up
BHGZD *vs.* AIA-M	LRRC66	0.0028	7.2584	Up
BHGZD *vs.* AIA-M	LRRC71	0.0044	0.3072	Down
BHGZD *vs.* AIA-M	LRRN2	0.0094	2.2147	Up
BHGZD *vs.* AIA-M	MAL	0.0017	2.0132	Up
BHGZD *vs.* AIA-M	MANEAL	0.0128	0.2761	Down

BHGZD *vs.* AIA-M	MAP10	0.0349	2.4321	Up
BHGZD *vs.* AIA-M	MAP1A	0.0036	0.4473	Down
BHGZD *vs.* AIA-M	MAP3K1	0.0216	0.4372	Down
BHGZD *vs.* AIA-M	MAP3K13	0.0272	3.2712	Up
BHGZD *vs.* AIA-M	MAPK8IP3	0.0068	0.3345	Down
BHGZD *vs.* AIA-M	MAPT	0.0191	3.6639	Up
BHGZD *vs.* AIA-M	MARCKS	0.0142	0.3904	Down
BHGZD *vs.* AIA-M	MARS2	0.0012	2.5011	Up
BHGZD *vs.* AIA-M	MAS1	0.0439	2.2098	Up
BHGZD *vs.* AIA-M	MCPT8L3	0.0442	10.1938	Up
BHGZD *vs.* AIA-M	MCPT9	0.0163	16.5660	Up
BHGZD *vs.* AIA-M	MDM1	0.0372	2.6490	Up
BHGZD *vs.* AIA-M	MECOM	0.0021	2.0404	Up
BHGZD *vs.* AIA-M	MEDAG	0.0046	0.3204	Down
BHGZD *vs.* AIA-M	MEF2A	0.0016	0.4772	Down
BHGZD *vs.* AIA-M	MEGF10	0.0486	3.8842	Up
BHGZD *vs.* AIA-M	MESDC1	0.0188	3.3461	Up
BHGZD *vs.* AIA-M	METAP1D	0.0259	2.0867	Up
BHGZD *vs.* AIA-M	MISP	0.0031	2.1665	Up
BHGZD *vs.* AIA-M	MIXL1	0.0417	0.3528	Down
BHGZD *vs.* AIA-M	MLPH	0.0410	2.7069	Up
BHGZD *vs.* AIA-M	MMP16	0.0054	0.4452	Down
BHGZD *vs.* AIA-M	MMP28	0.0139	2.1507	Up
BHGZD *vs.* AIA-M	MMP8	0.0285	20.6114	Up
BHGZD *vs.* AIA-M	MOV10	0.0247	0.1779	Down
BHGZD *vs.* AIA-M	MPZL1	0.0010	2.8057	Up
BHGZD *vs.* AIA-M	MSH5	0.0012	0.3499	Down

BHGZD *vs.* AIA-M	MSL3L2	0.0128	0.3244	Down
BHGZD *vs.* AIA-M	MSLNL	0.0031	0.3928	Down
BHGZD *vs.* AIA-M	MSX2	0.0441	2.3150	Up
BHGZD *vs.* AIA-M	MTHFR	0.0276	0.4959	Down
BHGZD *vs.* AIA-M	MTMR14	0.0436	2.4186	Up
BHGZD *vs.* AIA-M	MTMR3	0.0150	3.5544	Up
BHGZD *vs.* AIA-M	MTMR7	0.0127	2.2391	Up
BHGZD *vs.* AIA-M	MTSS1L	0.0282	0.1768	Down
BHGZD *vs.* AIA-M	MYBL2	0.0488	2.6690	Up
BHGZD *vs.* AIA-M	MYCBP2	0.0153	0.4521	Down
BHGZD *vs.* AIA-M	MYH10	0.0356	0.4167	Down
BHGZD *vs.* AIA-M	MYH15	0.0186	0.4766	Down
BHGZD *vs.* AIA-M	NADSYN1	0.0011	0.3890	Down
BHGZD *vs.* AIA-M	NCOA2	0.0026	0.4213	Down
BHGZD *vs.* AIA-M	NDUFC2	0.0365	0.3246	Down
BHGZD *vs.* AIA-M	NEDD4	0.0084	2.1043	Up
BHGZD *vs.* AIA-M	NEURL4	0.0030	0.4975	Down
BHGZD *vs.* AIA-M	NEUROD6	0.0268	0.3060	Down
BHGZD *vs.* AIA-M	NFIX	0.0241	0.4251	Down
BHGZD *vs.* AIA-M	NIACR1	0.0068	11.4308	Up
BHGZD *vs.* AIA-M	NID2	0.0004	0.4863	Down
BHGZD *vs.* AIA-M	NKD1	0.0079	0.3812	Down
BHGZD *vs.* AIA-M	NKG7	0.0068	3.5416	Up
BHGZD *vs.* AIA-M	NKX2-4	0.0000	0.4715	Down
BHGZD *vs.* AIA-M	NLGN3	0.0026	0.4480	Down
BHGZD *vs.* AIA-M	NOG	0.0227	3.1112	Up
BHGZD *vs.* AIA-M	NOS1AP	0.0118	2.2813	Up

BHGZD *vs.* AIA-M	NOTCH2	0.0202	0.4669	Down
BHGZD *vs.* AIA-M	NP4	0.0210	13.1493	Up
BHGZD *vs.* AIA-M	NPAS2	0.0342	0.4876	Down
BHGZD *vs.* AIA-M	NPB	0.0145	2.4925	Up
BHGZD *vs.* AIA-M	NPM1	0.0169	2.0764	Up
BHGZD *vs.* AIA-M	NPVF	0.0014	0.3926	Down
BHGZD *vs.* AIA-M	NR1D1	0.0101	2.0326	Up
BHGZD *vs.* AIA-M	NRN1L	0.0439	2.1784	Up
BHGZD *vs.* AIA-M	NTF3	0.0411	2.9258	Up
BHGZD *vs.* AIA-M	OBP3	0.0238	2.3166	Up
BHGZD *vs.* AIA-M	OLR1	0.0356	0.4156	Down
BHGZD *vs.* AIA-M	OLR1256	0.0319	0.4217	Down
BHGZD *vs.* AIA-M	OLR1416	0.0002	0.4409	Down
BHGZD *vs.* AIA-M	OLR1423	0.0013	0.4626	Down
BHGZD *vs.* AIA-M	OLR1572	0.0486	0.4973	Down
BHGZD *vs.* AIA-M	OLR1631	0.0138	0.4369	Down
BHGZD *vs.* AIA-M	OLR1658	0.0040	0.3781	Down
BHGZD *vs.* AIA-M	OLR183	0.0150	0.3161	Down
BHGZD *vs.* AIA-M	OLR3	0.0275	6.4064	Up
BHGZD *vs.* AIA-M	OLR309	0.0133	0.4789	Down
BHGZD *vs.* AIA-M	OLR384	0.0394	0.3866	Down
BHGZD *vs.* AIA-M	OLR4	0.0059	0.3154	Down
BHGZD *vs.* AIA-M	OLR588	0.0257	0.4641	Down
BHGZD *vs.* AIA-M	OLR660	0.0011	0.3730	Down
BHGZD *vs.* AIA-M	OLR663	0.0118	0.4785	Down
BHGZD *vs.* AIA-M	OLR776	0.0083	0.4927	Down
BHGZD *vs.* AIA-M	OLR791	0.0215	0.4762	Down

BHGZD *vs.* AIA-M	OPN4	0.0170	5.6110	Up
BHGZD *vs.* AIA-M	OPRD1	0.0082	0.2358	Down
BHGZD *vs.* AIA-M	ORC1	0.0362	2.5349	Up
BHGZD *vs.* AIA-M	OXNAD1	0.0019	0.2802	Down
BHGZD *vs.* AIA-M	P2RX1	0.0017	2.2555	Up
BHGZD *vs.* AIA-M	PAFAH2	0.0469	2.4106	Up
BHGZD *vs.* AIA-M	PAPLN	0.0413	2.3330	Up
BHGZD *vs.* AIA-M	PAQR5	0.0359	2.2310	Up
BHGZD *vs.* AIA-M	PARD3	0.0222	2.2885	Up
BHGZD *vs.* AIA-M	PATZ1	0.0270	2.5516	Up
BHGZD *vs.* AIA-M	PCSK1	0.0168	2.8247	Up
BHGZD *vs.* AIA-M	PDCD1LG2	0.0225	3.6308	Up
BHGZD *vs.* AIA-M	PDE4D	0.0116	0.4725	Down
BHGZD *vs.* AIA-M	PDE6H	0.0119	10.5006	Up
BHGZD *vs.* AIA-M	PDK2	0.0223	2.2880	Up
BHGZD *vs.* AIA-M	PDLIM5	0.0244	0.4974	Down
BHGZD *vs.* AIA-M	PDXP	0.0057	0.4122	Down
BHGZD *vs.* AIA-M	PDZD3	0.0330	2.5735	Up
BHGZD *vs.* AIA-M	PEG10	0.0392	0.4244	Down
BHGZD *vs.* AIA-M	PER3	0.0175	2.8615	Up
BHGZD *vs.* AIA-M	PFKFB2	0.0248	2.2403	Up
BHGZD *vs.* AIA-M	PFN2	0.0274	0.2697	Down
BHGZD *vs.* AIA-M	PFN3	0.0096	0.1256	Down
BHGZD *vs.* AIA-M	PGC	0.0150	0.4261	Down
BHGZD *vs.* AIA-M	PHF3	0.0211	2.0180	Up
BHGZD *vs.* AIA-M	PHKA2	0.0024	5.0034	Up
BHGZD *vs.* AIA-M	PHLDA3	0.0274	2.1964	Up

BHGZD *vs.* AIA-M	PHTF1	0.0050	0.4431	Down
BHGZD *vs.* AIA-M	PIEZO2	0.0424	0.1577	Down
BHGZD *vs.* AIA-M	PIGO	0.0134	0.2049	Down
BHGZD *vs.* AIA-M	PIGZL1	0.0398	0.3939	Down
BHGZD *vs.* AIA-M	PIK3CB	0.0386	2.2420	Up
BHGZD *vs.* AIA-M	PIK3R1	0.0003	0.3996	Down
BHGZD *vs.* AIA-M	PIWIL2	0.0281	3.3478	Up
BHGZD *vs.* AIA-M	PKHD1L1	0.0298	3.2192	Up
BHGZD *vs.* AIA-M	PKP1	0.0090	0.2573	Down
BHGZD *vs.* AIA-M	PLA2G2A	0.0408	2.9803	Up
BHGZD *vs.* AIA-M	PLA2R1	0.0142	2.4487	Up
BHGZD *vs.* AIA-M	PLEK2	0.0251	4.1387	Up
BHGZD *vs.* AIA-M	PLEKHA6	0.0317	2.3145	Up
BHGZD *vs.* AIA-M	PLEKHH1	0.0435	2.0994	Up
BHGZD *vs.* AIA-M	PLEKHO2	0.0003	0.1727	Down
BHGZD *vs.* AIA-M	PLIN4	0.0382	3.2501	Up
BHGZD *vs.* AIA-M	PLS1	0.0135	5.5189	Up
BHGZD *vs.* AIA-M	PLXNB3	0.0075	3.3997	Up
BHGZD *vs.* AIA-M	PNPLA7	0.0012	4.0875	Up
BHGZD *vs.* AIA-M	POU2AF1	0.0312	6.2493	Up
BHGZD *vs.* AIA-M	PPFIBP2	0.0161	0.4515	Down
BHGZD *vs.* AIA-M	PPL	0.0387	2.1173	Up
BHGZD *vs.* AIA-M	PPP1R3C	0.0130	3.4418	Up
BHGZD *vs.* AIA-M	PRG2	0.0339	21.0664	Up
BHGZD *vs.* AIA-M	PRIMA1	0.0058	0.4585	Down
BHGZD *vs.* AIA-M	PRKACB	0.0105	0.4158	Down
BHGZD *vs.* AIA-M	PRL3D4	0.0328	0.3757	Down

BHGZD *vs.* AIA-M	PROB1	0.0498	2.3304	Up
BHGZD *vs.* AIA-M	PROKR2	0.0224	0.4339	Down
BHGZD *vs.* AIA-M	PRPS1	0.0229	2.4804	Up
BHGZD *vs.* AIA-M	PRR15	0.0194	2.5309	Up
BHGZD *vs.* AIA-M	PRR5L	0.0239	2.2503	Up
BHGZD *vs.* AIA-M	PRRX1	0.0456	0.4283	Down
BHGZD *vs.* AIA-M	PRRX2	0.0452	0.4448	Down
BHGZD *vs.* AIA-M	PRSS2	0.0027	0.4177	Down
BHGZD *vs.* AIA-M	PRTN3	0.0362	5.1241	Up
BHGZD *vs.* AIA-M	PSIP1	0.0021	2.2954	Up
BHGZD *vs.* AIA-M	PSX1	0.0050	0.4872	Down
BHGZD *vs.* AIA-M	PTCHD1	0.0269	4.5268	Up
BHGZD *vs.* AIA-M	PTGER2	0.0365	2.5549	Up
BHGZD *vs.* AIA-M	PTMA	0.0051	0.4691	Down
BHGZD *vs.* AIA-M	PTPN1	0.0411	0.4424	Down
BHGZD *vs.* AIA-M	PTPRC	0.0368	0.4755	Down
BHGZD *vs.* AIA-M	PTPRD	0.0122	0.4441	Down
BHGZD *vs.* AIA-M	PTPRG	0.0402	2.0259	Up
BHGZD *vs.* AIA-M	PXT1	0.0135	3.6844	Up
BHGZD *vs.* AIA-M	PYY	0.0135	9.3718	Up
BHGZD *vs.* AIA-M	RAB3A	0.0336	2.8845	Up
BHGZD *vs.* AIA-M	RAB40C	0.0157	2.8799	Up
BHGZD *vs.* AIA-M	RAB8A	0.0224	0.4884	Down
BHGZD *vs.* AIA-M	RAD21L1	0.0183	0.1914	Down
BHGZD *vs.* AIA-M	RAP1GAP2	0.0085	4.4876	Up
BHGZD *vs.* AIA-M	RASA3	0.0419	0.4978	Down
BHGZD *vs.* AIA-M	RASGEF1C	0.0180	2.8349	Up

BHGZD *vs.* AIA-M	RASGRF1	0.0099	0.4270	Down
BHGZD *vs.* AIA-M	RASGRP3	0.0006	2.4959	Up
BHGZD *vs.* AIA-M	RASL12	0.0160	2.0039	Up
BHGZD *vs.* AIA-M	RATNP-3B	0.0246	32.3119	Up
BHGZD *vs.* AIA-M	RAVER2	0.0053	0.4768	Down
BHGZD *vs.* AIA-M	RB1	0.0216	2.2383	Up
BHGZD *vs.* AIA-M	RBM15B	0.0251	0.4698	Down
BHGZD *vs.* AIA-M	RBM4B	0.0457	2.7466	Up
BHGZD *vs.* AIA-M	RELN	0.0105	2.0713	Up
BHGZD *vs.* AIA-M	RFX2	0.0478	0.4451	Down
BHGZD *vs.* AIA-M	RGD1305202	0.0247	2.2941	Up
BHGZD *vs.* AIA-M	RGD1306941	0.0409	0.2374	Down
BHGZD *vs.* AIA-M	RGD1307947	0.0349	0.4859	Down
BHGZD *vs.* AIA-M	RGD1309049	0.0011	0.2922	Down
BHGZD *vs.* AIA-M	RGD1309762	0.0002	2.1732	Up
BHGZD *vs.* AIA-M	RGD1310262	0.0035	0.4943	Down
BHGZD *vs.* AIA-M	RGD1560958	0.0013	3.6757	Up
BHGZD *vs.* AIA-M	RGD1561517	0.0443	3.4994	Up
BHGZD *vs.* AIA-M	RGD1563354	0.0256	3.4591	Up
BHGZD *vs.* AIA-M	RGD1564664	0.0062	0.1560	Down
BHGZD *vs.* AIA-M	RGD1565143	0.0312	2.1589	Up
BHGZD *vs.* AIA-M	RGD1566186	0.0247	0.4376	Down
BHGZD *vs.* AIA-M	RGD1566264	0.0264	0.4979	Down
BHGZD *vs.* AIA-M	RGS20	0.0267	2.5602	Up
BHGZD *vs.* AIA-M	RHAG	0.0061	42.1004	Up
BHGZD *vs.* AIA-M	RN45S	0.0010	0.2593	Down
BHGZD *vs.* AIA-M	RNF112	0.0010	0.3182	Down

BHGZD *vs.* AIA-M	RNF150	0.0037	0.4448	Down
BHGZD *vs.* AIA-M	RNF182	0.0005	0.3687	Down
BHGZD *vs.* AIA-M	RNF24	0.0089	0.4769	Down
BHGZD *vs.* AIA-M	RPGRIP1L	0.0237	0.4714	Down
BHGZD *vs.* AIA-M	RPS6KL1	0.0481	2.2331	Up
BHGZD *vs.* AIA-M	RSPO3	0.0059	2.3392	Up
BHGZD *vs.* AIA-M	RT1-CE5	0.0087	0.4982	Down
BHGZD *vs.* AIA-M	RT1-T18	0.0196	0.4889	Down
BHGZD *vs.* AIA-M	RTN4RL1	0.0459	0.3734	Down
BHGZD *vs.* AIA-M	RTN4RL2	0.0000	0.2195	Down
BHGZD *vs.* AIA-M	RUNX3	0.0416	0.3738	Down
BHGZD *vs.* AIA-M	RXFP1	0.0184	2.7295	Up
BHGZD *vs.* AIA-M	S100A5	0.0223	4.7934	Up
BHGZD *vs.* AIA-M	S100A8	0.0291	15.7462	Up
BHGZD *vs.* AIA-M	SACS	0.0358	3.0474	Up
BHGZD *vs.* AIA-M	SALL3	0.0003	0.1025	Down
BHGZD *vs.* AIA-M	SAMD9L	0.0170	0.3759	Down
BHGZD *vs.* AIA-M	SC5D	0.0170	2.1292	Up
BHGZD *vs.* AIA-M	SCAF1	0.0004	0.4155	Down
BHGZD *vs.* AIA-M	SCARA3	0.0124	0.4780	Down
BHGZD *vs.* AIA-M	SCD	0.0362	0.1810	Down
BHGZD *vs.* AIA-M	SCRG1	0.0018	6.9662	Up
BHGZD *vs.* AIA-M	SDS	0.0109	0.3728	Down
BHGZD *vs.* AIA-M	SEC23A	0.0300	0.4989	Down
BHGZD *vs.* AIA-M	SEC24D	0.0226	0.3923	Down
BHGZD *vs.* AIA-M	SEMA3A	0.0197	0.3510	Down
BHGZD *vs.* AIA-M	SEMA7A	0.0046	0.4820	Down

BHGZD *vs.* AIA-M	SERINC2	0.0218	4.6493	Up
BHGZD *vs.* AIA-M	SERPINB10	0.0093	20.8117	Up
BHGZD *vs.* AIA-M	SERPINB7	0.0045	0.2457	Down
BHGZD *vs.* AIA-M	SERPINF2	0.0158	3.3191	Up
BHGZD *vs.* AIA-M	SFRP2	0.0440	0.3608	Down
BHGZD *vs.* AIA-M	SGCG	0.0245	0.4864	Down
BHGZD *vs.* AIA-M	SH2D5	0.0329	4.6893	Up
BHGZD *vs.* AIA-M	SH3BP4	0.0183	2.0800	Up
BHGZD *vs.* AIA-M	SHANK1	0.0088	0.4785	Down
BHGZD *vs.* AIA-M	SHBG	0.0042	0.1729	Down
BHGZD *vs.* AIA-M	SHMT1	0.0225	2.1147	Up
BHGZD *vs.* AIA-M	SHOC2	0.0001	0.4317	Down
BHGZD *vs.* AIA-M	SIGLEC10	0.0203	2.2390	Up
BHGZD *vs.* AIA-M	SIRT4	0.0243	2.2070	Up
BHGZD *vs.* AIA-M	SLC12A7	0.0013	0.1628	Down
BHGZD *vs.* AIA-M	SLC14A2	0.0178	0.3743	Down
BHGZD *vs.* AIA-M	SLC16A10	0.0148	3.3071	Up
BHGZD *vs.* AIA-M	SLC16A12	0.0408	2.1570	Up
BHGZD *vs.* AIA-M	SLC16A14	0.0009	0.3495	Down
BHGZD *vs.* AIA-M	SLC19A2	0.0054	2.5607	Up
BHGZD *vs.* AIA-M	SLC22A5	0.0025	2.9357	Up
BHGZD *vs.* AIA-M	SLC26A10	0.0208	3.9026	Up
BHGZD *vs.* AIA-M	SLC26A2	0.0171	0.4651	Down
BHGZD *vs.* AIA-M	SLC27A1	0.0120	2.6400	Up
BHGZD *vs.* AIA-M	SLC30A3	0.0015	0.4097	Down
BHGZD *vs.* AIA-M	SLC31A1	0.0279	0.3400	Down
BHGZD *vs.* AIA-M	SLC35A2	0.0001	0.4099	Down

BHGZD *vs.* AIA-M	SLC39A13	0.0391	0.4962	Down
BHGZD *vs.* AIA-M	SLC45A4	0.0114	0.2303	Down
BHGZD *vs.* AIA-M	SLC46A3	0.0270	2.6161	Up
BHGZD *vs.* AIA-M	SLC4A10	0.0190	4.3808	Up
BHGZD *vs.* AIA-M	SLC5A4	0.0057	0.2314	Down
BHGZD *vs.* AIA-M	SLC6A6	0.0007	2.8127	Up
BHGZD *vs.* AIA-M	SLC7A6OS	0.0137	2.7576	Up
BHGZD *vs.* AIA-M	SLC9A6	0.0261	2.1014	Up
BHGZD *vs.* AIA-M	SLIT1	0.0086	0.4706	Down
BHGZD *vs.* AIA-M	SLIT2	0.0238	0.4182	Down
BHGZD *vs.* AIA-M	SNED1	0.0000	0.2447	Down
BHGZD *vs.* AIA-M	SNX18	0.0286	0.4541	Down
BHGZD *vs.* AIA-M	SOX18	0.0344	2.0132	Up
BHGZD *vs.* AIA-M	SOX4	0.0299	0.4587	Down
BHGZD *vs.* AIA-M	SPATA2	0.0200	0.3291	Down
BHGZD *vs.* AIA-M	SPATA31D3	0.0406	0.4402	Down
BHGZD *vs.* AIA-M	SPATA6L	0.0322	2.6870	Up
BHGZD *vs.* AIA-M	SPRY1	0.0099	2.2946	Up
BHGZD *vs.* AIA-M	SPSB4	0.0190	0.2411	Down
BHGZD *vs.* AIA-M	SPTA1	0.0363	9.5993	Up
BHGZD *vs.* AIA-M	SRCIN1	0.0037	0.2349	Down
BHGZD *vs.* AIA-M	SRGN	0.0382	2.6861	Up
BHGZD *vs.* AIA-M	SRRM3	0.0012	2.2917	Up
BHGZD *vs.* AIA-M	ST6GALNAC3	0.0436	2.0062	Up
BHGZD *vs.* AIA-M	STAB2	0.0374	3.8511	Up
BHGZD *vs.* AIA-M	STAR	0.0213	2.6284	Up
BHGZD *vs.* AIA-M	STAU2	0.0019	3.5801	Up

BHGZD *vs.* AIA-M	SULF1	0.0124	2.1566	Up
BHGZD *vs.* AIA-M	SURF6	0.0154	0.3473	Down
BHGZD *vs.* AIA-M	SUSD5	0.0129	0.3962	Down
BHGZD *vs.* AIA-M	SUV39H1	0.0096	3.8594	Up
BHGZD *vs.* AIA-M	SVS3B	0.0368	0.4953	Down
BHGZD *vs.* AIA-M	SYNE1	0.0198	2.2951	Up
BHGZD *vs.* AIA-M	SYVN1	0.0350	2.0462	Up
BHGZD *vs.* AIA-M	TAC3	0.0045	0.4511	Down
BHGZD *vs.* AIA-M	TAOK3	0.0391	0.4979	Down
BHGZD *vs.* AIA-M	TBC1D1	0.0292	0.4385	Down
BHGZD *vs.* AIA-M	TBC1D15	0.0111	2.2861	Up
BHGZD *vs.* AIA-M	TBC1D22B	0.0088	2.1601	Up
BHGZD *vs.* AIA-M	TBC1D23	0.0001	0.4320	Down
BHGZD *vs.* AIA-M	TBX4	0.0237	2.2216	Up
BHGZD *vs.* AIA-M	TCF7	0.0008	0.4774	Down
BHGZD *vs.* AIA-M	TEKT3	0.0108	0.3935	Down
BHGZD *vs.* AIA-M	TERC	0.0167	3.0392	Up
BHGZD *vs.* AIA-M	TEX22	0.0002	0.1478	Down
BHGZD *vs.* AIA-M	TFPI	0.0129	2.2050	Up
BHGZD *vs.* AIA-M	TFRC	0.0338	3.0530	Up
BHGZD *vs.* AIA-M	TGFA	0.0328	3.0885	Up
BHGZD *vs.* AIA-M	TGFBR2	0.0381	0.4674	Down
BHGZD *vs.* AIA-M	THBS1	0.0022	0.1141	Down
BHGZD *vs.* AIA-M	THEG	0.0411	0.4334	Down
BHGZD *vs.* AIA-M	TIAM1	0.0046	2.2586	Up
BHGZD *vs.* AIA-M	TLX3	0.0027	0.2204	Down
BHGZD *vs.* AIA-M	TMEM101	0.0423	2.1184	Up

BHGZD *vs.* AIA-M	TMEM116	0.0199	3.1051	Up
BHGZD *vs.* AIA-M	TMEM120A	0.0302	2.6476	Up
BHGZD *vs.* AIA-M	TMEM136	0.0034	2.1245	Up
BHGZD *vs.* AIA-M	TMEM170B	0.0004	0.4647	Down
BHGZD *vs.* AIA-M	TMEM229A	0.0147	0.2337	Down
BHGZD *vs.* AIA-M	TMEM65	0.0345	0.4447	Down
BHGZD *vs.* AIA-M	TNFAIP2	0.0118	0.4574	Down
BHGZD *vs.* AIA-M	TNMD	0.0245	0.0273	Down
BHGZD *vs.* AIA-M	TPD52L1	0.0082	3.4924	Up
BHGZD *vs.* AIA-M	TRAF3IP2	0.0241	2.0413	Up
BHGZD *vs.* AIA-M	TRIM26	0.0091	0.4391	Down
BHGZD *vs.* AIA-M	TRPS1	0.0295	0.4616	Down
BHGZD *vs.* AIA-M	TRPV4	0.0330	0.1894	Down
BHGZD *vs.* AIA-M	TTC28	0.0001	0.4974	Down
BHGZD *vs.* AIA-M	TTL	0.0131	0.4912	Down
BHGZD *vs.* AIA-M	TUBB3	0.0031	0.2207	Down
BHGZD *vs.* AIA-M	TWF1	0.0391	0.4871	Down
BHGZD *vs.* AIA-M	TWIST2	0.0148	0.1320	Down
BHGZD *vs.* AIA-M	TYRP1	0.0240	0.0728	Down
BHGZD *vs.* AIA-M	UCK1	0.0490	2.1211	Up
BHGZD *vs.* AIA-M	UCMA	0.0215	0.4842	Down
BHGZD *vs.* AIA-M	UGT2B37	0.0086	0.4247	Down
BHGZD *vs.* AIA-M	UNC5CL	0.0164	2.6192	Up
BHGZD *vs.* AIA-M	UPK1B	0.0059	2.3892	Up
BHGZD *vs.* AIA-M	UPRT	0.0394	0.3654	Down
BHGZD *vs.* AIA-M	USB1	0.0096	2.4116	Up
BHGZD *vs.* AIA-M	USP25	0.0091	0.4834	Down

BHGZD *vs.* AIA-M	UTP18	0.0202	0.3060	Down
BHGZD *vs.* AIA-M	UTP20	0.0053	0.3535	Down
BHGZD *vs.* AIA-M	UTS2R	0.0391	0.0988	Down
BHGZD *vs.* AIA-M	VAMP2	0.0169	2.1739	Up
BHGZD *vs.* AIA-M	VANGL2	0.0201	0.3083	Down
BHGZD *vs.* AIA-M	VARS	0.0318	2.7269	Up
BHGZD *vs.* AIA-M	VKORC1L1	0.0261	2.1558	Up
BHGZD *vs.* AIA-M	VOM2R44	0.0292	3.5320	Up
BHGZD *vs.* AIA-M	VPREB3	0.0033	16.4933	Up
BHGZD *vs.* AIA-M	VPS37B	0.0113	2.2007	Up
BHGZD *vs.* AIA-M	VSNL1	0.0161	2.0197	Up
BHGZD *vs.* AIA-M	VWA8	0.0001	2.2325	Up
BHGZD *vs.* AIA-M	WDFY3	0.0050	0.2116	Down
BHGZD *vs.* AIA-M	WDR93	0.0341	2.2703	Up
BHGZD *vs.* AIA-M	WIF1	0.0373	4.4304	Up
BHGZD *vs.* AIA-M	WNT11	0.0049	4.6054	Up
BHGZD *vs.* AIA-M	WWTR1	0.0382	0.4475	Down
BHGZD *vs.* AIA-M	YKT6	0.0072	0.4340	Down
BHGZD *vs.* AIA-M	YTHDF2	0.0006	0.1848	Down
BHGZD *vs.* AIA-M	ZAK	0.0025	0.4285	Down
BHGZD *vs.* AIA-M	ZBTB20	0.0278	0.2387	Down
BHGZD *vs.* AIA-M	ZBTB24	0.0120	6.3954	Up
BHGZD *vs.* AIA-M	ZCCHC10	0.0022	0.4698	Down
BHGZD *vs.* AIA-M	ZCCHC14	0.0236	0.4881	Down
BHGZD *vs.* AIA-M	ZDHHC18	0.0167	0.1401	Down
BHGZD *vs.* AIA-M	ZDHHC23	0.0059	2.7416	Up
BHGZD *vs.* AIA-M	ZFAND4	0.0104	2.5769	Up

BHGZD *vs.* AIA-M	ZFP185	0.0091	0.2521	Down
BHGZD *vs.* AIA-M	ZFP213	0.0376	3.9199	Up
BHGZD *vs.* AIA-M	ZFP39	0.0480	2.0992	Up
BHGZD *vs.* AIA-M	ZFP458	0.0092	0.4990	Down
BHGZD *vs.* AIA-M	ZFP575	0.0047	0.3078	Down
BHGZD *vs.* AIA-M	ZFP667	0.0409	2.2474	Up
BHGZD *vs.* AIA-M	ZFP93	0.0062	0.4933	Down
BHGZD *vs.* AIA-M	ZFP956	0.0131	5.0045	Up
BHGZD *vs.* AIA-M	ZFPM2	0.0343	0.2962	Down
BHGZD *vs.* AIA-M	ZFYVE27	0.0295	2.3899	Up
BHGZD *vs.* AIA-M	ZNRF4	0.0016	0.1113	Down
BHGZD *vs.* AIA-M	ZXDC	0.0168	0.2641	Down

Table S5 The significant differentially expressed genes (DEGs) identified by mRNA Sequencing (mRNA-Seq)

Groups	Symbol	P-value	log2FoldChange	Regulation
AIA-M *vs.* Con_mRNA_Seq	AA926063	0.0103	4.6924	Up
AIA-M *vs.* Con_mRNA_Seq	ABCA4	0.0333	-1.0431	Down
AIA-M *vs.* Con_mRNA_Seq	ACSBG1	0.0137	2.3568	Up
AIA-M *vs.* Con_mRNA_Seq	ADAD1	0.0436	-5.1493	Down
AIA-M *vs.* Con_mRNA_Seq	ADAM3A	0.0437	-5.1437	Down
AIA-M *vs.* Con_mRNA_Seq	ADAP2	0.0010	3.0242	Up
AIA-M *vs.* Con_mRNA_Seq	ADCY5	0.0011	1.5517	Up
AIA-M *vs.* Con_mRNA_Seq	AGRP	0.0234	1.4030	Up
AIA-M *vs.* Con_mRNA_Seq	AHRR	0.0004	-4.3563	Down
AIA-M *vs.* Con_mRNA_Seq	AKAP12	0.0020	-1.5834	Down
AIA-M *vs.* Con_mRNA_Seq	ALDOC	0.0010	2.4691	Up
AIA-M *vs.* Con_mRNA_Seq	ANGPTL2	0.0417	3.7609	Up
AIA-M *vs.* Con_mRNA_Seq	ANTXR2	0.0337	1.0409	Up
AIA-M *vs.* Con_mRNA_Seq	AOC3	0.0145	-5.7090	Down
AIA-M *vs.* Con_mRNA_Seq	APC2	0.0097	4.6623	Up
AIA-M *vs.* Con_mRNA_Seq	APOBEC1	0.0122	1.5453	Up
AIA-M *vs.* Con_mRNA_Seq	ARHGAP18	0.0033	1.0271	Up
AIA-M *vs.* Con_mRNA_Seq	ARHGEF4	0.0259	1.8822	Up
AIA-M *vs.* Con_mRNA_Seq	ART4	0.0004	-1.3477	Down
AIA-M *vs.* Con_mRNA_Seq	ASS1	0.0048	1.0208	Up
AIA-M *vs.* Con_mRNA_Seq	ASTN1	0.0034	1.1875	Up
AIA-M *vs.* Con_mRNA_Seq	ATF3	0.0002	1.8320	Up
AIA-M *vs.* Con_mRNA_Seq	ATF5	0.0006	1.7382	Up

AIA-M *vs.* Con_mRNA_Seq	AVPI1	0.0312	1.0902	Up
AIA-M *vs.* Con_mRNA_Seq	BATF	0.0023	1.0684	Up
AIA-M *vs.* Con_mRNA_Seq	BCL2L15	0.0424	-1.7395	Down
AIA-M *vs.* Con_mRNA_Seq	BCL7A	0.0081	4.3723	Up
AIA-M *vs.* Con_mRNA_Seq	BFSP2	0.0039	1.3618	Up
AIA-M *vs.* Con_mRNA_Seq	BGLAP	0.0072	1.1948	Up
AIA-M *vs.* Con_mRNA_Seq	BRICD5	0.0180	4.9630	Up
AIA-M *vs.* Con_mRNA_Seq	BRMS1L	0.0383	-1.0459	Down
AIA-M *vs.* Con_mRNA_Seq	BTBD8	0.0350	-1.5747	Down
AIA-M *vs.* Con_mRNA_Seq	BTD	0.0066	1.2074	Up
AIA-M *vs.* Con_mRNA_Seq	BTLA	0.0352	-1.0068	Down
AIA-M *vs.* Con_mRNA_Seq	BTNL10	0.0128	-1.7170	Down
AIA-M *vs.* Con_mRNA_Seq	CAMK1	0.0379	3.2749	Up
AIA-M *vs.* Con_mRNA_Seq	CASP12	0.0011	-3.1532	Down
AIA-M *vs.* Con_mRNA_Seq	CASTOR2	0.0201	-2.4924	Down
AIA-M *vs.* Con_mRNA_Seq	CBLN2	0.0021	1.1423	Up
AIA-M *vs.* Con_mRNA_Seq	CCDC92	0.0000	1.7220	Up
AIA-M *vs.* Con_mRNA_Seq	CCHCR1	0.0030	-1.3041	Down
AIA-M *vs.* Con_mRNA_Seq	CCL3	0.0003	1.9617	Up
AIA-M *vs.* Con_mRNA_Seq	CCL4	0.0475	1.1632	Up
AIA-M *vs.* Con_mRNA_Seq	CCL6	0.0000	1.7045	Up
AIA-M *vs.* Con_mRNA_Seq	CD101	0.0000	1.1296	Up
AIA-M *vs.* Con_mRNA_Seq	CD177	0.0031	-1.9882	Down
AIA-M *vs.* Con_mRNA_Seq	CD300LB	0.0021	1.2526	Up
AIA-M *vs.* Con_mRNA_Seq	CD79AL	0.0081	-1.1535	Down
AIA-M *vs.* Con_mRNA_Seq	CDC20	0.0236	-1.1730	Down
AIA-M *vs.* Con_mRNA_Seq	CDC6	0.0311	-1.4314	Down

AIA-M *vs.* Con_mRNA_Seq	CDCA2	0.0117	-3.2077	Down
AIA-M *vs.* Con_mRNA_Seq	CDK1	0.0221	-1.7974	Down
AIA-M *vs.* Con_mRNA_Seq	CEBPD	0.0041	1.0660	Up
AIA-M *vs.* Con_mRNA_Seq	CELA1	0.0012	1.5977	Up
AIA-M *vs.* Con_mRNA_Seq	CENPT	0.0286	-1.4661	Down
AIA-M *vs.* Con_mRNA_Seq	CENPU	0.0019	-1.4502	Down
AIA-M *vs.* Con_mRNA_Seq	CEP70	0.0064	-1.3041	Down
AIA-M *vs.* Con_mRNA_Seq	CEP83	0.0108	-1.1933	Down
AIA-M *vs.* Con_mRNA_Seq	CFAP126	0.0037	2.6429	Up
AIA-M *vs.* Con_mRNA_Seq	CFAP70	0.0109	1.3155	Up
AIA-M *vs.* Con_mRNA_Seq	CFAP97	0.0000	-1.5669	Down
AIA-M *vs.* Con_mRNA_Seq	CHRD	0.0100	2.3895	Up
AIA-M *vs.* Con_mRNA_Seq	CLEC11A	0.0133	4.4798	Up
AIA-M *vs.* Con_mRNA_Seq	CLEC4B2	0.0016	1.6781	Up
AIA-M *vs.* Con_mRNA_Seq	CLEC5A	0.0014	1.1640	Up
AIA-M *vs.* Con_mRNA_Seq	CLU	0.0039	1.0155	Up
AIA-M *vs.* Con_mRNA_Seq	COL20A1	0.0469	1.0120	Up
AIA-M *vs.* Con_mRNA_Seq	COL4A3	0.0000	-2.4803	Down
AIA-M *vs.* Con_mRNA_Seq	CRABP1	0.0243	1.9562	Up
AIA-M *vs.* Con_mRNA_Seq	CRABP2//LOC100911902	0.0057	-4.8634	Down
AIA-M *vs.* Con_mRNA_Seq	CRISPLD2	0.0062	1.3742	Up
AIA-M *vs.* Con_mRNA_Seq	CSF1	0.0065	1.2866	Up
AIA-M *vs.* Con_mRNA_Seq	CTDSPL	0.0348	1.0889	Up
AIA-M *vs.* Con_mRNA_Seq	CTTN	0.0137	1.0143	Up
AIA-M *vs.* Con_mRNA_Seq	CXCL17	0.0164	3.2503	Up
AIA-M *vs.* Con_mRNA_Seq	CXCL2	0.0012	1.9025	Up

AIA-M *vs.* Con_mRNA_Seq	CXXC4	0.0126	1.1896	Up
AIA-M *vs.* Con_mRNA_Seq	CYB5D1	0.0367	-1.0541	Down
AIA-M *vs.* Con_mRNA_Seq	CYP26C1	0.0388	4.4533	Up
AIA-M *vs.* Con_mRNA_Seq	CYP2U1	0.0365	-1.0153	Down
AIA-M *vs.* Con_mRNA_Seq	DACT1	0.0091	3.5048	Up
AIA-M *vs.* Con_mRNA_Seq	DCLK1	0.0123	-5.0458	Down
AIA-M *vs.* Con_mRNA_Seq	DDX25	0.0396	-3.1180	Down
AIA-M *vs.* Con_mRNA_Seq	DEFB24	0.0345	2.8901	Up
AIA-M *vs.* Con_mRNA_Seq	DENND2C	0.0242	1.1059	Up
AIA-M *vs.* Con_mRNA_Seq	DHFR	0.0419	-1.1018	Down
AIA-M *vs.* Con_mRNA_Seq	DNAH1	0.0186	1.1723	Up
AIA-M *vs.* Con_mRNA_Seq	DNAJC4	0.0372	1.0106	Up
AIA-M *vs.* Con_mRNA_Seq	DOK4	0.0109	1.2929	Up
AIA-M *vs.* Con_mRNA_Seq	DUSP1	0.0000	1.4543	Up
AIA-M *vs.* Con_mRNA_Seq	DUSP6	0.0000	1.1605	Up
AIA-M *vs.* Con_mRNA_Seq	DUSP7	0.0073	1.1521	Up
AIA-M *vs.* Con_mRNA_Seq	EBF1	0.0117	-1.2113	Down
AIA-M *vs.* Con_mRNA_Seq	ECE2	0.0297	3.5704	Up
AIA-M *vs.* Con_mRNA_Seq	ECT2	0.0095	4.5099	Up
AIA-M *vs.* Con_mRNA_Seq	EDN1	0.0333	-2.8289	Down
AIA-M *vs.* Con_mRNA_Seq	EGR3	0.0400	-2.6568	Down
AIA-M *vs.* Con_mRNA_Seq	EHD2	0.0323	1.0293	Up
AIA-M *vs.* Con_mRNA_Seq	ELN	0.0335	4.4083	Up
AIA-M *vs.* Con_mRNA_Seq	EMILIN1	0.0492	1.4588	Up
AIA-M *vs.* Con_mRNA_Seq	ENTPD1	0.0003	1.1267	Up
AIA-M *vs.* Con_mRNA_Seq	EPB41L3	0.0001	-3.7357	Down
AIA-M *vs.* Con_mRNA_Seq	EPHA1	0.0033	1.3204	Up

AIA-M *vs.* Con_mRNA_Seq	ERBB3	0.0343	1.9450	Up
AIA-M *vs.* Con_mRNA_Seq	ERFE	0.0315	3.3489	Up
AIA-M *vs.* Con_mRNA_Seq	ESPL1	0.0443	-2.2720	Down
AIA-M *vs.* Con_mRNA_Seq	F11R	0.0037	1.1213	Up
AIA-M *vs.* Con_mRNA_Seq	F2RL3	0.0056	1.1166	Up
AIA-M *vs.* Con_mRNA_Seq	FAAP20	0.0038	1.0066	Up
AIA-M *vs.* Con_mRNA_Seq	FAM166A	0.0304	-1.1430	Down
AIA-M *vs.* Con_mRNA_Seq	FAM183A	0.0211	2.2844	Up
AIA-M *vs.* Con_mRNA_Seq	FAM184A	0.0416	1.6124	Up
AIA-M *vs.* Con_mRNA_Seq	FAM25A	0.0161	-2.7549	Down
AIA-M *vs.* Con_mRNA_Seq	FAM72A	0.0012	-5.2848	Down
AIA-M *vs.* Con_mRNA_Seq	FARS2	0.0219	-1.0924	Down
AIA-M *vs.* Con_mRNA_Seq	FBXO24	0.0077	4.4980	Up
AIA-M *vs.* Con_mRNA_Seq	FCGR2A//LOC108348047	0.0003	1.0137	Up
AIA-M *vs.* Con_mRNA_Seq	FCGRT	0.0315	-1.0021	Down
AIA-M *vs.* Con_mRNA_Seq	FNDC9	0.0044	-1.8445	Down
AIA-M *vs.* Con_mRNA_Seq	FOLR2	0.0297	3.4803	Up
AIA-M *vs.* Con_mRNA_Seq	FOS	0.0000	2.1489	Up
AIA-M *vs.* Con_mRNA_Seq	FRG2	0.0125	-5.1115	Down
AIA-M *vs.* Con_mRNA_Seq	FRMD4B	0.0323	-1.2156	Down
AIA-M *vs.* Con_mRNA_Seq	GAB1	0.0436	-3.1717	Down
AIA-M *vs.* Con_mRNA_Seq	GADD45G	0.0463	1.1221	Up
AIA-M *vs.* Con_mRNA_Seq	GAS2L1	0.0021	1.1011	Up
AIA-M *vs.* Con_mRNA_Seq	GATA1//LOC108348091	0.0076	-1.2542	Down
AIA-M *vs.* Con_mRNA_Seq	GDPD5	0.0028	1.5424	Up

AIA-M *vs.* Con_mRNA_Seq	GFOD2	0.0465	1.9332	Up
AIA-M *vs.* Con_mRNA_Seq	GFY	0.0007	-5.6188	Down
AIA-M *vs.* Con_mRNA_Seq	GIN1	0.0308	1.3586	Up
AIA-M *vs.* Con_mRNA_Seq	GNAZ	0.0086	1.0121	Up
AIA-M *vs.* Con_mRNA_Seq	GP1BB	0.0008	1.9090	Up
AIA-M *vs.* Con_mRNA_Seq	GP9	0.0038	1.0026	Up
AIA-M *vs.* Con_mRNA_Seq	GPNMB	0.0033	1.1974	Up
AIA-M *vs.* Con_mRNA_Seq	GRB10	0.0005	2.6721	Up
AIA-M *vs.* Con_mRNA_Seq	GSTA1//LOC1083480 61	0.0053	1.0067	Up
AIA-M *vs.* Con_mRNA_Seq	GSTM5//GSTM3	0.0056	-1.6182	Down
AIA-M *vs.* Con_mRNA_Seq	HAVCR2	0.0464	-1.2644	Down
AIA-M *vs.* Con_mRNA_Seq	HGF	0.0301	1.0278	Up
AIA-M *vs.* Con_mRNA_Seq	HIVEP3	0.0017	-1.7890	Down
AIA-M *vs.* Con_mRNA_Seq	HLX	0.0030	1.1103	Up
AIA-M *vs.* Con_mRNA_Seq	HSPA1A//HSPA1B//L OC108348108	0.0154	-1.5354	Down
AIA-M *vs.* Con_mRNA_Seq	HSPA1A//LOC108348 108	0.0129	-1.6746	Down
AIA-M *vs.* Con_mRNA_Seq	HSPA2	0.0381	1.8257	Up
AIA-M *vs.* Con_mRNA_Seq	HSPB6	0.0095	-1.4183	Down
AIA-M *vs.* Con_mRNA_Seq	ID1	0.0000	1.1211	Up
AIA-M *vs.* Con_mRNA_Seq	IFIT1	0.0000	1.4917	Up
AIA-M *vs.* Con_mRNA_Seq	IFIT3	0.0000	1.6551	Up
AIA-M *vs.* Con_mRNA_Seq	IKZF5	0.0000	-1.8353	Down
AIA-M *vs.* Con_mRNA_Seq	IL1B	0.0078	1.4138	Up
AIA-M *vs.* Con_mRNA_Seq	IL22RA2	0.0361	2.1436	Up

AIA-M *vs.* Con_mRNA_Seq	IL7	0.0200	3.6803	Up
AIA-M *vs.* Con_mRNA_Seq	ITGA5	0.0001	1.2829	Up
AIA-M *vs.* Con_mRNA_Seq	JAML	0.0001	1.0184	Up
AIA-M *vs.* Con_mRNA_Seq	JUN	0.0001	1.6961	Up
AIA-M *vs.* Con_mRNA_Seq	JUND	0.0000	1.1377	Up
AIA-M *vs.* Con_mRNA_Seq	KATNAL1//LOC100910196//LOC103690050	0.0248	1.5441	Up
AIA-M *vs.* Con_mRNA_Seq	KCNK13	0.0214	2.3729	Up
AIA-M *vs.* Con_mRNA_Seq	KCNK5	0.0166	-1.1022	Down
AIA-M *vs.* Con_mRNA_Seq	KCNS3	0.0105	1.0348	Up
AIA-M *vs.* Con_mRNA_Seq	KCP	0.0035	-1.5882	Down
AIA-M *vs.* Con_mRNA_Seq	KIFAP3	0.0000	-1.5116	Down
AIA-M *vs.* Con_mRNA_Seq	KLHL13	0.0216	2.3729	Up
AIA-M *vs.* Con_mRNA_Seq	KLHL25	0.0019	-1.3820	Down
AIA-M *vs.* Con_mRNA_Seq	KLRA1	0.0005	1.2788	Up
AIA-M *vs.* Con_mRNA_Seq	KLRA22	0.0132	-2.1066	Down
AIA-M *vs.* Con_mRNA_Seq	KRT80	0.0488	4.2739	Up
AIA-M *vs.* Con_mRNA_Seq	LARGE1	0.0112	1.0049	Up
AIA-M *vs.* Con_mRNA_Seq	LARS2	0.0254	1.2180	Up
AIA-M *vs.* Con_mRNA_Seq	LEXM	0.0038	-4.2771	Down
AIA-M *vs.* Con_mRNA_Seq	LHFPL6	0.0305	2.1809	Up
AIA-M *vs.* Con_mRNA_Seq	LIPG	0.0374	-1.2935	Down
AIA-M *vs.* Con_mRNA_Seq	LOC100361934	0.0095	-1.5734	Down
AIA-M *vs.* Con_mRNA_Seq	LOC100362724	0.0134	-1.4664	Down
AIA-M *vs.* Con_mRNA_Seq	LOC100909700	0.0116	-2.0239	Down
AIA-M *vs.* Con_mRNA_Seq	LOC100910792	0.0004	-2.7609	Down
AIA-M *vs.* Con_mRNA_Seq	LOC100911572	0.0339	-2.3851	Down

AIA-M *vs.* Con_mRNA_Seq	LOC100912228	0.0074	1.0055	Up
AIA-M *vs.* Con_mRNA_Seq	LOC100912534	0.0242	1.7074	Up
AIA-M *vs.* Con_mRNA_Seq	LOC102546716	0.0064	-1.5135	Down
AIA-M *vs.* Con_mRNA_Seq	LOC102550455	0.0086	-1.2006	Down
AIA-M *vs.* Con_mRNA_Seq	LOC102550543	0.0429	-3.2803	Down
AIA-M *vs.* Con_mRNA_Seq	LOC102551606	0.0002	-1.0154	Down
AIA-M *vs.* Con_mRNA_Seq	LOC102552166	0.0318	-1.2249	Down
AIA-M *vs.* Con_mRNA_Seq	LOC102555023	0.0380	1.3830	Up
AIA-M *vs.* Con_mRNA_Seq	LOC102555038	0.0015	-1.6568	Down
AIA-M *vs.* Con_mRNA_Seq	LOC102556092	0.0026	1.1719	Up
AIA-M *vs.* Con_mRNA_Seq	LOC103689947	0.0097	-1.1436	Down
AIA-M *vs.* Con_mRNA_Seq	LOC103689949	0.0014	2.0670	Up
AIA-M *vs.* Con_mRNA_Seq	LOC103689954	0.0011	1.0917	Up
AIA-M *vs.* Con_mRNA_Seq	LOC103689960	0.0221	-1.4032	Down
AIA-M *vs.* Con_mRNA_Seq	LOC103692167	0.0225	-1.1241	Down
AIA-M *vs.* Con_mRNA_Seq	LOC103692995	0.0473	-1.2011	Down
AIA-M *vs.* Con_mRNA_Seq	LOC498154	0.0016	1.0649	Up
AIA-M *vs.* Con_mRNA_Seq	LOC680254	0.0278	1.5841	Up
AIA-M *vs.* Con_mRNA_Seq	LOC680322	0.0301	-1.9543	Down
AIA-M *vs.* Con_mRNA_Seq	LOC685157//LOC108348155	0.0398	1.0708	Up
AIA-M *vs.* Con_mRNA_Seq	LOC688282	0.0252	-1.6940	Down
AIA-M *vs.* Con_mRNA_Seq	LOC688925	0.0305	-3.1015	Down
AIA-M *vs.* Con_mRNA_Seq	LOXL3	0.0022	1.3740	Up
AIA-M *vs.* Con_mRNA_Seq	LRATD2	0.0000	1.4946	Up
AIA-M *vs.* Con_mRNA_Seq	LRP8	0.0101	-1.4916	Down
AIA-M *vs.* Con_mRNA_Seq	LRRC71	0.0178	1.4124	Up

AIA-M *vs.* Con_mRNA_Seq	LTC4S	0.0077	4.9559	Up
AIA-M *vs.* Con_mRNA_Seq	LXN	0.0171	1.7025	Up
AIA-M *vs.* Con_mRNA_Seq	LY49SI2//LY49SI3	0.0301	2.4436	Up
AIA-M *vs.* Con_mRNA_Seq	LY6G6F	0.0043	1.0222	Up
AIA-M *vs.* Con_mRNA_Seq	LYPLAL1	0.0113	4.3395	Up
AIA-M *vs.* Con_mRNA_Seq	LYSMD4	0.0375	-1.2065	Down
AIA-M *vs.* Con_mRNA_Seq	LYVE1	0.0023	1.0159	Up
AIA-M *vs.* Con_mRNA_Seq	MAP6	0.0128	1.3777	Up
AIA-M *vs.* Con_mRNA_Seq	MAPK8IP1	0.0317	2.5304	Up
AIA-M *vs.* Con_mRNA_Seq	MARCKS	0.0003	1.0128	Up
AIA-M *vs.* Con_mRNA_Seq	MAST2	0.0044	-1.1431	Down
AIA-M *vs.* Con_mRNA_Seq	MCPT1L1	0.0003	1.8206	Up
AIA-M *vs.* Con_mRNA_Seq	MDM1	0.0017	1.1322	Up
AIA-M *vs.* Con_mRNA_Seq	MED9	0.0134	1.4502	Up
AIA-M *vs.* Con_mRNA_Seq	MEPE	0.0130	4.9576	Up
AIA-M *vs.* Con_mRNA_Seq	MEST	0.0296	1.5656	Up
AIA-M *vs.* Con_mRNA_Seq	METRN	0.0289	-1.2884	Down
AIA-M *vs.* Con_mRNA_Seq	MICALL2	0.0005	-1.3657	Down
AIA-M *vs.* Con_mRNA_Seq	MIR5132	0.0155	-1.4769	Down
AIA-M *vs.* Con_mRNA_Seq	MMRN1	0.0026	1.0609	Up
AIA-M *vs.* Con_mRNA_Seq	MMRN2	0.0275	-2.8201	Down
AIA-M *vs.* Con_mRNA_Seq	MRVI1	0.0113	1.0361	Up
AIA-M *vs.* Con_mRNA_Seq	MSRA	0.0266	1.8396	Up
AIA-M *vs.* Con_mRNA_Seq	MUC1	0.0086	2.3010	Up
AIA-M *vs.* Con_mRNA_Seq	MYCT1	0.0058	1.1718	Up
AIA-M *vs.* Con_mRNA_Seq	MYL9	0.0009	1.2004	Up

AIA-M *vs.* Con_mRNA_Seq	N4BP3//LOC103689949	0.0017	2.0777	Up
AIA-M *vs.* Con_mRNA_Seq	NANP	0.0311	-1.0680	Down
AIA-M *vs.* Con_mRNA_Seq	NCAPG	0.0011	-2.1619	Down
AIA-M *vs.* Con_mRNA_Seq	NCEH1	0.0019	1.0923	Up
AIA-M *vs.* Con_mRNA_Seq	NEDD4L	0.0024	-1.0062	Down
AIA-M *vs.* Con_mRNA_Seq	NGFR	0.0088	-1.0127	Down
AIA-M *vs.* Con_mRNA_Seq	NGP	0.0008	-1.6765	Down
AIA-M *vs.* Con_mRNA_Seq	NIACR1	0.0006	1.5101	Up
AIA-M *vs.* Con_mRNA_Seq	NLRP3	0.0001	1.4900	Up
AIA-M *vs.* Con_mRNA_Seq	NMT2	0.0000	-1.6290	Down
AIA-M *vs.* Con_mRNA_Seq	NR4A2	0.0118	-1.2631	Down
AIA-M *vs.* Con_mRNA_Seq	NR4A3	0.0408	-1.4472	Down
AIA-M *vs.* Con_mRNA_Seq	NRG2	0.0157	4.3541	Up
AIA-M *vs.* Con_mRNA_Seq	NRGN	0.0008	1.1404	Up
AIA-M *vs.* Con_mRNA_Seq	NRIP3	0.0188	1.1768	Up
AIA-M *vs.* Con_mRNA_Seq	NUPR1	0.0106	5.2624	Up
AIA-M *vs.* Con_mRNA_Seq	NUPR1L1	0.0115	5.2231	Up
AIA-M *vs.* Con_mRNA_Seq	OASL	0.0001	1.1970	Up
AIA-M *vs.* Con_mRNA_Seq	OBSL1	0.0488	1.6679	Up
AIA-M *vs.* Con_mRNA_Seq	OLFM4	0.0065	-1.0349	Down
AIA-M *vs.* Con_mRNA_Seq	OPTN	0.0148	-1.1208	Down
AIA-M *vs.* Con_mRNA_Seq	ORAI1	0.0003	1.1646	Up
AIA-M *vs.* Con_mRNA_Seq	OSGIN1	0.0003	1.2142	Up
AIA-M *vs.* Con_mRNA_Seq	OSM	0.0000	1.5136	Up
AIA-M *vs.* Con_mRNA_Seq	P2RX4	0.0002	1.0018	Up
AIA-M *vs.* Con_mRNA_Seq	PAQR3	0.0040	-1.8678	Down

AIA-M *vs.* Con_mRNA_Seq	PCDHGB5	0.0262	-3.9559	Down
AIA-M *vs.* Con_mRNA_Seq	PCGF3	0.0009	1.3573	Up
AIA-M *vs.* Con_mRNA_Seq	PCLO	0.0394	-1.9650	Down
AIA-M *vs.* Con_mRNA_Seq	PDE3A	0.0116	1.0241	Up
AIA-M *vs.* Con_mRNA_Seq	PELI2	0.0371	3.2349	Up
AIA-M *vs.* Con_mRNA_Seq	PEX10	0.0181	2.7096	Up
AIA-M *vs.* Con_mRNA_Seq	PF4	0.0007	1.2675	Up
AIA-M *vs.* Con_mRNA_Seq	PIH1D1	0.0109	-1.0603	Down
AIA-M *vs.* Con_mRNA_Seq	PKIA	0.0016	1.2370	Up
AIA-M *vs.* Con_mRNA_Seq	PLA2G2A	0.0003	1.3067	Up
AIA-M *vs.* Con_mRNA_Seq	PLEKHG1	0.0051	-2.3095	Down
AIA-M *vs.* Con_mRNA_Seq	PLOD2	0.0338	1.3673	Up
AIA-M *vs.* Con_mRNA_Seq	PLPP3	0.0106	2.4407	Up
AIA-M *vs.* Con_mRNA_Seq	PLTP	0.0003	1.4100	Up
AIA-M *vs.* Con_mRNA_Seq	PMP22	0.0031	-1.7572	Down
AIA-M *vs.* Con_mRNA_Seq	POLR2H	0.0088	2.1539	Up
AIA-M *vs.* Con_mRNA_Seq	POMC	0.0449	5.0129	Up
AIA-M *vs.* Con_mRNA_Seq	PPIF	0.0030	1.0631	Up
AIA-M *vs.* Con_mRNA_Seq	PPP1R3B	0.0019	1.0795	Up
AIA-M *vs.* Con_mRNA_Seq	PROS1	0.0156	2.2391	Up
AIA-M *vs.* Con_mRNA_Seq	PROSER2	0.0106	2.4378	Up
AIA-M *vs.* Con_mRNA_Seq	PRXL2A	0.0254	-1.0486	Down
AIA-M *vs.* Con_mRNA_Seq	PTPN13	0.0158	4.1086	Up
AIA-M *vs.* Con_mRNA_Seq	QRSL1	0.0309	-1.3194	Down
AIA-M *vs.* Con_mRNA_Seq	RAB26	0.0460	-1.1139	Down
AIA-M *vs.* Con_mRNA_Seq	RAB3IL1	0.0189	-1.1683	Down
AIA-M *vs.* Con_mRNA_Seq	RAD54L2	0.0061	-1.0560	Down

AIA-M *vs.* Con_mRNA_Seq	RASL10A	0.0028	1.1205	Up
AIA-M *vs.* Con_mRNA_Seq	RBPJL	0.0431	-4.5238	Down
AIA-M *vs.* Con_mRNA_Seq	REEP2	0.0048	1.0181	Up
AIA-M *vs.* Con_mRNA_Seq	REXO5	0.0408	-1.4795	Down
AIA-M *vs.* Con_mRNA_Seq	RFX2	0.0227	-1.0409	Down
AIA-M *vs.* Con_mRNA_Seq	RGD1307621	0.0382	-1.6219	Down
AIA-M *vs.* Con_mRNA_Seq	RGD1560020_PREDICTED	0.0074	-1.3435	Down
AIA-M *vs.* Con_mRNA_Seq	RGD1560455	0.0019	-1.1466	Down
AIA-M *vs.* Con_mRNA_Seq	RGD1564664	0.0486	1.4050	Up
AIA-M *vs.* Con_mRNA_Seq	RGD1565785	0.0391	1.1993	Up
AIA-M *vs.* Con_mRNA_Seq	RGSL1	0.0080	2.6706	Up
AIA-M *vs.* Con_mRNA_Seq	RHOB	0.0000	1.1005	Up
AIA-M *vs.* Con_mRNA_Seq	RHOC	0.0404	1.1268	Up
AIA-M *vs.* Con_mRNA_Seq	RHOV	0.0000	1.3743	Up
AIA-M *vs.* Con_mRNA_Seq	RMDN3	0.0077	-1.1777	Down
AIA-M *vs.* Con_mRNA_Seq	RNF150	0.0319	1.2786	Up
AIA-M *vs.* Con_mRNA_Seq	RNF217	0.0339	1.4001	Up
AIA-M *vs.* Con_mRNA_Seq	RPAP1	0.0027	1.0304	Up
AIA-M *vs.* Con_mRNA_Seq	RPP25	0.0048	1.8057	Up
AIA-M *vs.* Con_mRNA_Seq	RT1-HA	0.0248	-1.3847	Down
AIA-M *vs.* Con_mRNA_Seq	RTP4	0.0350	-1.1421	Down
AIA-M *vs.* Con_mRNA_Seq	S100A13	0.0191	1.0713	Up
AIA-M *vs.* Con_mRNA_Seq	SCAI	0.0173	-1.2765	Down
AIA-M *vs.* Con_mRNA_Seq	SCARF1	0.0006	1.7774	Up
AIA-M *vs.* Con_mRNA_Seq	SCHIP1	0.0188	1.4847	Up
AIA-M *vs.* Con_mRNA_Seq	SCRN3	0.0222	1.0721	Up

AIA-M *vs.* Con_mRNA_Seq	SDC4	0.0381	-1.5525	Down
AIA-M *vs.* Con_mRNA_Seq	SEC14L5	0.0134	1.3750	Up
AIA-M *vs.* Con_mRNA_Seq	SELENBP1	0.0245	-1.1029	Down
AIA-M *vs.* Con_mRNA_Seq	SELP	0.0029	1.2168	Up
AIA-M *vs.* Con_mRNA_Seq	SEMA3G	0.0014	1.0387	Up
AIA-M *vs.* Con_mRNA_Seq	SEMA5A	0.0110	2.8501	Up
AIA-M *vs.* Con_mRNA_Seq	SEMA6B	0.0009	1.3686	Up
AIA-M *vs.* Con_mRNA_Seq	SEPT10	0.0426	-1.7620	Down
AIA-M *vs.* Con_mRNA_Seq	SERINC2	0.0000	1.4189	Up
AIA-M *vs.* Con_mRNA_Seq	SERPINE2	0.0383	2.2082	Up
AIA-M *vs.* Con_mRNA_Seq	SERPINI2	0.0165	3.7645	Up
AIA-M *vs.* Con_mRNA_Seq	SETDB2	0.0350	1.5217	Up
AIA-M *vs.* Con_mRNA_Seq	SGCB	0.0355	-1.0415	Down
AIA-M *vs.* Con_mRNA_Seq	SH3BGR	0.0004	2.4272	Up
AIA-M *vs.* Con_mRNA_Seq	SHPK	0.0005	1.0305	Up
AIA-M *vs.* Con_mRNA_Seq	SIGLEC5	0.0001	2.2340	Up
AIA-M *vs.* Con_mRNA_Seq	SLC12A2	0.0002	1.0832	Up
AIA-M *vs.* Con_mRNA_Seq	SLC13A5	0.0331	1.8593	Up
AIA-M *vs.* Con_mRNA_Seq	SLC24A5	0.0301	1.0299	Up
AIA-M *vs.* Con_mRNA_Seq	SLC26A2	0.0225	1.1763	Up
AIA-M *vs.* Con_mRNA_Seq	SLC45A4	0.0066	1.3026	Up
AIA-M *vs.* Con_mRNA_Seq	SLC48A1	0.0393	-1.0079	Down
AIA-M *vs.* Con_mRNA_Seq	SLC6A4	0.0021	1.0946	Up
AIA-M *vs.* Con_mRNA_Seq	SLC6A9	0.0485	-1.1161	Down
AIA-M *vs.* Con_mRNA_Seq	SMPDL3B	0.0071	1.0133	Up
AIA-M *vs.* Con_mRNA_Seq	SMYD5	0.0397	1.0642	Up
AIA-M *vs.* Con_mRNA_Seq	SOCS3	0.0003	1.0616	Up

AIA-M *vs.* Con_mRNA_Seq	SPAG5	0.0240	-1.2852	Down
AIA-M *vs.* Con_mRNA_Seq	SPDYA	0.0003	-5.7343	Down
AIA-M *vs.* Con_mRNA_Seq	SPNS3	0.0473	1.5093	Up
AIA-M *vs.* Con_mRNA_Seq	SPON2//LOC100910790	0.0471	2.2389	Up
AIA-M *vs.* Con_mRNA_Seq	SPR	0.0376	-1.0121	Down
AIA-M *vs.* Con_mRNA_Seq	SPTB	0.0121	-1.1604	Down
AIA-M *vs.* Con_mRNA_Seq	STAMBP	0.0109	-1.0631	Down
AIA-M *vs.* Con_mRNA_Seq	STOML1	0.0211	1.3810	Up
AIA-M *vs.* Con_mRNA_Seq	STON2	0.0063	1.8772	Up
AIA-M *vs.* Con_mRNA_Seq	STXBP4	0.0417	-2.0812	Down
AIA-M *vs.* Con_mRNA_Seq	SYT5	0.0003	1.1579	Up
AIA-M *vs.* Con_mRNA_Seq	SYTL4	0.0402	1.6712	Up
AIA-M *vs.* Con_mRNA_Seq	TAL1	0.0073	1.0319	Up
AIA-M *vs.* Con_mRNA_Seq	TASP1	0.0356	-1.2363	Down
AIA-M *vs.* Con_mRNA_Seq	TBCEL	0.0280	-1.1219	Down
AIA-M *vs.* Con_mRNA_Seq	TCTEX1D4	0.0462	-3.8619	Down
AIA-M *vs.* Con_mRNA_Seq	TDRKH	0.0121	1.6143	Up
AIA-M *vs.* Con_mRNA_Seq	TEX14	0.0316	-4.5866	Down
AIA-M *vs.* Con_mRNA_Seq	THBS1	0.0086	1.0434	Up
AIA-M *vs.* Con_mRNA_Seq	TLR5	0.0167	1.1053	Up
AIA-M *vs.* Con_mRNA_Seq	TMEM51	0.0065	3.9376	Up
AIA-M *vs.* Con_mRNA_Seq	TMEM56	0.0015	1.7935	Up
AIA-M *vs.* Con_mRNA_Seq	TNFAIP2	0.0009	1.2803	Up
AIA-M *vs.* Con_mRNA_Seq	TP53TG5	0.0109	4.6235	Up
AIA-M *vs.* Con_mRNA_Seq	TPRKB//LOC103690067	0.0010	-1.6372	Down

AIA-M *vs.* Con_mRNA_Seq	TRAIP	0.0140	4.2532	Up
AIA-M *vs.* Con_mRNA_Seq	TREML1	0.0069	1.0076	Up
AIA-M *vs.* Con_mRNA_Seq	TREML4	0.0037	-1.1950	Down
AIA-M *vs.* Con_mRNA_Seq	TRIM13	0.0109	1.3526	Up
AIA-M *vs.* Con_mRNA_Seq	TRIM17	0.0278	1.6098	Up
AIA-M *vs.* Con_mRNA_Seq	TRIM72	0.0105	1.2347	Up
AIA-M *vs.* Con_mRNA_Seq	TSPY26	0.0041	-1.7901	Down
AIA-M *vs.* Con_mRNA_Seq	TTC30B	0.0045	2.4533	Up
AIA-M *vs.* Con_mRNA_Seq	TYRO3	0.0372	1.0424	Up
AIA-M *vs.* Con_mRNA_Seq	UNC13B	0.0047	1.2061	Up
AIA-M *vs.* Con_mRNA_Seq	VEGFA	0.0025	2.3371	Up
AIA-M *vs.* Con_mRNA_Seq	VKORC1L1	0.0031	-1.4402	Down
AIA-M *vs.* Con_mRNA_Seq	VPREB3	0.0360	-1.6525	Down
AIA-M *vs.* Con_mRNA_Seq	VSIG2	0.0204	1.4849	Up
AIA-M *vs.* Con_mRNA_Seq	VWF	0.0005	1.0792	Up
AIA-M *vs.* Con_mRNA_Seq	WIPF3	0.0012	-1.5561	Down
AIA-M *vs.* Con_mRNA_Seq	WNT2	0.0406	2.4945	Up
AIA-M *vs.* Con_mRNA_Seq	XKR8	0.0050	1.0216	Up
AIA-M *vs.* Con_mRNA_Seq	YAP1	0.0060	2.6466	Up
AIA-M *vs.* Con_mRNA_Seq	ZBTB20	0.0344	-1.5200	Down
AIA-M *vs.* Con_mRNA_Seq	ZCCHC12	0.0010	3.7146	Up
AIA-M *vs.* Con_mRNA_Seq	ZFP1	0.0240	1.2585	Up
AIA-M *vs.* Con_mRNA_Seq	ZFP286A	0.0438	-1.4919	Down
AIA-M *vs.* Con_mRNA_Seq	ZFP36	0.0000	1.1974	Up
AIA-M *vs.* Con_mRNA_Seq	ZFP780B-PS1	0.0081	-1.6098	Down
AIA-M *vs.* Con_mRNA_Seq	ZIK1	0.0086	4.5098	Up
AIA-M *vs.* Con_mRNA_Seq	ZIK1//LOC100911224	0.0103	4.4248	Up

AIA-M *vs.* Con_mRNA_Seq	ZSCAN21	0.0000	-1.3327	Down
BHGZD *vs.* AIA-M_mRNA_Seq	AAMP	0.0107	-1.0302	Down
BHGZD *vs.* AIA-M_mRNA_Seq	ABCA4	0.0093	-1.2777	Down
BHGZD *vs.* AIA-M_mRNA_Seq	ABCB6	0.0007	-1.1470	Down
BHGZD *vs.* AIA-M_mRNA_Seq	ABCC4	0.0063	-1.0376	Down
BHGZD *vs.* AIA-M_mRNA_Seq	ABCG4	0.0251	-1.9310	Down
BHGZD *vs.* AIA-M_mRNA_Seq	ABHD4	0.0051	-1.1666	Down
BHGZD *vs.* AIA-M_mRNA_Seq	ABI2	0.0014	-1.1421	Down
BHGZD *vs.* AIA-M_mRNA_Seq	ABTB1	0.0014	-1.2998	Down
BHGZD *vs.* AIA-M_mRNA_Seq	ACER2	0.0000	-1.3738	Down
BHGZD *vs.* AIA-M_mRNA_Seq	ACHE	0.0034	-1.3507	Down
BHGZD *vs.* AIA-M_mRNA_Seq	ADCK5	0.0137	-1.1067	Down
BHGZD *vs.* AIA-M_mRNA_Seq	ADCY1	0.0476	-2.2888	Down
BHGZD *vs.* AIA-M_mRNA_Seq	ADCY5	0.0001	-1.4196	Down
BHGZD *vs.* AIA-M_mRNA_Seq	ADCY9	0.0043	-1.2446	Down
BHGZD *vs.* AIA-M_mRNA_Seq	ADD2	0.0025	-1.3714	Down
BHGZD *vs.* AIA-M_mRNA_Seq	ADGRA2	0.0002	-1.4634	Down
BHGZD *vs.* AIA-M_mRNA_Seq	ADIPOR1	0.0011	-1.4399	Down
BHGZD *vs.* AIA-M_mRNA_Seq	ADK	0.0002	-1.0966	Down
BHGZD *vs.* AIA-M_mRNA_Seq	AEN	0.0032	-1.1175	Down
BHGZD *vs.* AIA-M_mRNA_Seq	AGAP1	0.0007	-1.2636	Down
BHGZD *vs.* AIA-M_mRNA_Seq	AGO2	0.0011	-1.4280	Down
BHGZD *vs.* AIA-M_mRNA_Seq	AGPAT5	0.0001	-1.0730	Down
BHGZD *vs.* AIA-M_mRNA_Seq	AGRP	0.0001	-2.2359	Down
BHGZD *vs.* AIA-M_mRNA_Seq	AHSP	0.0133	-1.3125	Down
BHGZD *vs.* AIA-M_mRNA_Seq	AKR7A3	0.0035	-1.3361	Down
BHGZD *vs.* AIA-M_mRNA_Seq	AKT3	0.0001	-1.2611	Down

BHGZD *vs.* AIA-M_mRNA_Seq	ALAD	0.0066	-1.2525	Down
BHGZD *vs.* AIA-M_mRNA_Seq	ALAS2	0.0010	-1.6221	Down
BHGZD *vs.* AIA-M_mRNA_Seq	ALDOC	0.0036	-1.3832	Down
BHGZD *vs.* AIA-M_mRNA_Seq	ALOX12	0.0000	-1.4374	Down
BHGZD *vs.* AIA-M_mRNA_Seq	ALOX15	0.0220	-1.7222	Down
BHGZD *vs.* AIA-M_mRNA_Seq	AMD1	0.0003	-1.0269	Down
BHGZD *vs.* AIA-M_mRNA_Seq	AMER1	0.0215	1.5514	Up
BHGZD *vs.* AIA-M_mRNA_Seq	AMFR	0.0010	-1.5027	Down
BHGZD *vs.* AIA-M_mRNA_Seq	ANGPT1	0.0001	-1.1717	Down
BHGZD *vs.* AIA-M_mRNA_Seq	ANGPTL2	0.0384	-2.6154	Down
BHGZD *vs.* AIA-M_mRNA_Seq	ANK1	0.0101	-1.2299	Down
BHGZD *vs.* AIA-M_mRNA_Seq	ANKS6	0.0465	2.1130	Up
BHGZD *vs.* AIA-M_mRNA_Seq	ANO6	0.0001	-1.0585	Down
BHGZD *vs.* AIA-M_mRNA_Seq	AP2M1	0.0007	-1.0612	Down
BHGZD *vs.* AIA-M_mRNA_Seq	AP3S1	0.0004	-1.0338	Down
BHGZD *vs.* AIA-M_mRNA_Seq	APCDD1L	0.0482	3.8063	Up
BHGZD *vs.* AIA-M_mRNA_Seq	APH1B//LOC108348064	0.0003	-1.0032	Down
BHGZD *vs.* AIA-M_mRNA_Seq	APOA2	0.0458	2.5232	Up
BHGZD *vs.* AIA-M_mRNA_Seq	AQP1	0.0076	-1.2653	Down
BHGZD *vs.* AIA-M_mRNA_Seq	ARF3//FKBP11	0.0034	-1.0636	Down
BHGZD *vs.* AIA-M_mRNA_Seq	ARF5	0.0007	-1.6146	Down
BHGZD *vs.* AIA-M_mRNA_Seq	ARHGAP10	0.0000	-1.3165	Down
BHGZD *vs.* AIA-M_mRNA_Seq	ARHGAP28	0.0006	-1.1830	Down
BHGZD *vs.* AIA-M_mRNA_Seq	ARHGAP6	0.0022	-1.2358	Down
BHGZD *vs.* AIA-M_mRNA_Seq	ARHGEF12	0.0014	-1.3088	Down
BHGZD *vs.* AIA-M_mRNA_Seq	ARHGEF37	0.0123	-1.3564	Down

BHGZD *vs.* AIA-M_mRNA_Seq	ARHGEF9	0.0257	2.0776	Up
BHGZD *vs.* AIA-M_mRNA_Seq	ARL2	0.0036	-1.2377	Down
BHGZD *vs.* AIA-M_mRNA_Seq	ARPC2	0.0019	-1.1846	Down
BHGZD *vs.* AIA-M_mRNA_Seq	ARPP19//LOC100360828	0.0217	-1.1836	Down
BHGZD *vs.* AIA-M_mRNA_Seq	ARSI	0.0066	2.9481	Up
BHGZD *vs.* AIA-M_mRNA_Seq	ASAP2	0.0225	-1.0137	Down
BHGZD *vs.* AIA-M_mRNA_Seq	ASB1	0.0017	-1.3956	Down
BHGZD *vs.* AIA-M_mRNA_Seq	ASB15	0.0001	-2.4716	Down
BHGZD *vs.* AIA-M_mRNA_Seq	ASF1B	0.0041	-1.2976	Down
BHGZD *vs.* AIA-M_mRNA_Seq	ASNA1	0.0014	-1.2627	Down
BHGZD *vs.* AIA-M_mRNA_Seq	ASTN1	0.0000	-1.4422	Down
BHGZD *vs.* AIA-M_mRNA_Seq	ATF5	0.0144	-1.7147	Down
BHGZD *vs.* AIA-M_mRNA_Seq	ATOX1	0.0012	-1.1513	Down
BHGZD *vs.* AIA-M_mRNA_Seq	ATP1B1	0.0007	1.2697	Up
BHGZD *vs.* AIA-M_mRNA_Seq	ATP1B2	0.0453	-1.0145	Down
BHGZD *vs.* AIA-M_mRNA_Seq	ATP5F1B	0.0023	-1.3325	Down
BHGZD *vs.* AIA-M_mRNA_Seq	ATP5IF1	0.0022	-1.4060	Down
BHGZD *vs.* AIA-M_mRNA_Seq	ATP5MF	0.0120	-1.0865	Down
BHGZD *vs.* AIA-M_mRNA_Seq	ATP5MG	0.0013	-1.4043	Down
BHGZD *vs.* AIA-M_mRNA_Seq	ATP5PD	0.0011	-1.2698	Down
BHGZD *vs.* AIA-M_mRNA_Seq	ATP5PO	0.0044	-1.0386	Down
BHGZD *vs.* AIA-M_mRNA_Seq	ATP6AP1L	0.0017	-1.7003	Down
BHGZD *vs.* AIA-M_mRNA_Seq	ATP6V1F	0.0007	-1.4106	Down
BHGZD *vs.* AIA-M_mRNA_Seq	ATP6V1FNB	0.0397	4.3882	Up
BHGZD *vs.* AIA-M_mRNA_Seq	ATP7B	0.0016	-1.9597	Down
BHGZD *vs.* AIA-M_mRNA_Seq	ATP8A1	0.0001	-1.1367	Down

BHGZD *vs.* AIA-M_mRNA_Seq	ATP8B5P	0.0033	-2.6232	Down
BHGZD *vs.* AIA-M_mRNA_Seq	AUP1	0.0020	-1.2665	Down
BHGZD *vs.* AIA-M_mRNA_Seq	B2M	0.0007	-1.2009	Down
BHGZD *vs.* AIA-M_mRNA_Seq	B3GALNT1	0.0321	3.0816	Up
BHGZD *vs.* AIA-M_mRNA_Seq	BAG6	0.0007	-1.3772	Down
BHGZD *vs.* AIA-M_mRNA_Seq	BARD1	0.0023	-1.1967	Down
BHGZD *vs.* AIA-M_mRNA_Seq	BBIP1	0.0100	-1.0169	Down
BHGZD *vs.* AIA-M_mRNA_Seq	BCAS2	0.0011	-1.5315	Down
BHGZD *vs.* AIA-M_mRNA_Seq	BCL11A	0.0208	1.1655	Up
BHGZD *vs.* AIA-M_mRNA_Seq	BCL2L1	0.0046	-1.2943	Down
BHGZD *vs.* AIA-M_mRNA_Seq	BCO1	0.0020	-1.2627	Down
BHGZD *vs.* AIA-M_mRNA_Seq	BCS1L	0.0334	1.8381	Up
BHGZD *vs.* AIA-M_mRNA_Seq	BEST1	0.0003	-1.3829	Down
BHGZD *vs.* AIA-M_mRNA_Seq	BFSP2	0.0004	-1.5460	Down
BHGZD *vs.* AIA-M_mRNA_Seq	BGLAP	0.0003	-1.3003	Down
BHGZD *vs.* AIA-M_mRNA_Seq	BGN	0.0000	-1.4733	Down
BHGZD *vs.* AIA-M_mRNA_Seq	BIVM	0.0263	1.4631	Up
BHGZD *vs.* AIA-M_mRNA_Seq	BLES03	0.0139	-1.0254	Down
BHGZD *vs.* AIA-M_mRNA_Seq	BLVRB	0.0033	-1.3744	Down
BHGZD *vs.* AIA-M_mRNA_Seq	BNIP3L	0.0011	-1.5070	Down
BHGZD *vs.* AIA-M_mRNA_Seq	BOLA3	0.0015	-1.4669	Down
BHGZD *vs.* AIA-M_mRNA_Seq	BORCS8	0.0028	-1.3322	Down
BHGZD *vs.* AIA-M_mRNA_Seq	BPGM	0.0023	-1.3788	Down
BHGZD *vs.* AIA-M_mRNA_Seq	BPHL	0.0019	4.8637	Up
BHGZD *vs.* AIA-M_mRNA_Seq	BPIFA6	0.0027	-1.3520	Down
BHGZD *vs.* AIA-M_mRNA_Seq	BRICD5	0.0112	-4.5770	Down
BHGZD *vs.* AIA-M_mRNA_Seq	BSDC1	0.0020	-1.5832	Down

BHGZD *vs.* AIA-M_mRNA_Seq	BTLA	0.0059	1.0449	Up
BHGZD *vs.* AIA-M_mRNA_Seq	BUD31	0.0064	-1.2633	Down
BHGZD *vs.* AIA-M_mRNA_Seq	C1QL4	0.0121	-5.9619	Down
BHGZD *vs.* AIA-M_mRNA_Seq	CA11	0.0118	-1.4098	Down
BHGZD *vs.* AIA-M_mRNA_Seq	CABYR	0.0037	-1.2199	Down
BHGZD *vs.* AIA-M_mRNA_Seq	CACNG3	0.0276	-1.1138	Down
BHGZD *vs.* AIA-M_mRNA_Seq	CADM4	0.0163	-4.2839	Down
BHGZD *vs.* AIA-M_mRNA_Seq	CALD1	0.0001	-1.5063	Down
BHGZD *vs.* AIA-M_mRNA_Seq	CALR	0.0049	-1.0703	Down
BHGZD *vs.* AIA-M_mRNA_Seq	CAMKV	0.0340	-2.3783	Down
BHGZD *vs.* AIA-M_mRNA_Seq	CAPN8	0.0457	-1.2357	Down
BHGZD *vs.* AIA-M_mRNA_Seq	CAPNS1//LOC100911363	0.0064	-1.1181	Down
BHGZD *vs.* AIA-M_mRNA_Seq	CAR1	0.0376	-1.1403	Down
BHGZD *vs.* AIA-M_mRNA_Seq	CAR2	0.0018	-1.4741	Down
BHGZD *vs.* AIA-M_mRNA_Seq	CARHSP1	0.0003	-1.6610	Down
BHGZD *vs.* AIA-M_mRNA_Seq	CASP12	0.0236	2.2173	Up
BHGZD *vs.* AIA-M_mRNA_Seq	CAT	0.0085	-1.1279	Down
BHGZD *vs.* AIA-M_mRNA_Seq	CAVIN1	0.0028	-1.8521	Down
BHGZD *vs.* AIA-M_mRNA_Seq	CAVIN2	0.0025	-1.2540	Down
BHGZD *vs.* AIA-M_mRNA_Seq	CBLN2	0.0000	-1.5381	Down
BHGZD *vs.* AIA-M_mRNA_Seq	CBX5	0.0030	1.1093	Up
BHGZD *vs.* AIA-M_mRNA_Seq	CCDC112	0.0179	1.2179	Up
BHGZD *vs.* AIA-M_mRNA_Seq	CCDC126	0.0156	-1.0977	Down
BHGZD *vs.* AIA-M_mRNA_Seq	CCDC127	0.0368	1.4294	Up
BHGZD *vs.* AIA-M_mRNA_Seq	CCDC189	0.0011	-1.5718	Down
BHGZD *vs.* AIA-M_mRNA_Seq	CCDC92	0.0001	-1.7041	Down

BHGZD *vs.* AIA-M_mRNA_Seq	CCN1	0.0067	1.0189	Up
BHGZD *vs.* AIA-M_mRNA_Seq	CCNJL	0.0003	-1.3404	Down
BHGZD *vs.* AIA-M_mRNA_Seq	CCR3	0.0110	-1.0801	Down
BHGZD *vs.* AIA-M_mRNA_Seq	CD151//LOC100911730	0.0000	-1.3641	Down
BHGZD *vs.* AIA-M_mRNA_Seq	CD177	0.0098	2.0804	Up
BHGZD *vs.* AIA-M_mRNA_Seq	CD226	0.0000	-1.3063	Down
BHGZD *vs.* AIA-M_mRNA_Seq	CD34	0.0136	-5.0562	Down
BHGZD *vs.* AIA-M_mRNA_Seq	CD52	0.0003	-1.7161	Down
BHGZD *vs.* AIA-M_mRNA_Seq	CD79AL	0.0045	1.1442	Up
BHGZD *vs.* AIA-M_mRNA_Seq	CD84	0.0005	-1.0392	Down
BHGZD *vs.* AIA-M_mRNA_Seq	CD9	0.0000	-1.2845	Down
BHGZD *vs.* AIA-M_mRNA_Seq	CDC25B	0.0012	-1.4613	Down
BHGZD *vs.* AIA-M_mRNA_Seq	CDC42BPA	0.0001	-1.4030	Down
BHGZD *vs.* AIA-M_mRNA_Seq	CDCA2	0.0013	2.8039	Up
BHGZD *vs.* AIA-M_mRNA_Seq	CDCA3	0.0100	-1.6918	Down
BHGZD *vs.* AIA-M_mRNA_Seq	CDKL1	0.0090	-1.4450	Down
BHGZD *vs.* AIA-M_mRNA_Seq	CDKN3	0.0026	-1.7457	Down
BHGZD *vs.* AIA-M_mRNA_Seq	CDR2	0.0051	-1.2937	Down
BHGZD *vs.* AIA-M_mRNA_Seq	CDS1	0.0014	1.2314	Up
BHGZD *vs.* AIA-M_mRNA_Seq	CEACAM18	0.0386	1.4692	Up
BHGZD *vs.* AIA-M_mRNA_Seq	CELA1	0.0007	-1.6140	Down
BHGZD *vs.* AIA-M_mRNA_Seq	CENPB	0.0071	-1.1725	Down
BHGZD *vs.* AIA-M_mRNA_Seq	CENPT	0.0433	-1.5497	Down
BHGZD *vs.* AIA-M_mRNA_Seq	CENPW	0.0108	-1.6547	Down
BHGZD *vs.* AIA-M_mRNA_Seq	CEP104	0.0063	-1.1266	Down
BHGZD *vs.* AIA-M_mRNA_Seq	CEP295NL	0.0052	4.5164	Up

BHGZD *vs.* AIA-M_mRNA_Seq	CEP83	0.0065	-1.1149	Down
BHGZD *vs.* AIA-M_mRNA_Seq	CEP89	0.0137	-1.1533	Down
BHGZD *vs.* AIA-M_mRNA_Seq	CFAP57	0.0384	-2.4903	Down
BHGZD *vs.* AIA-M_mRNA_Seq	CGAS	0.0410	-1.2327	Down
BHGZD *vs.* AIA-M_mRNA_Seq	CGREF1	0.0308	-6.5602	Down
BHGZD *vs.* AIA-M_mRNA_Seq	CH25H	0.0283	3.8635	Up
BHGZD *vs.* AIA-M_mRNA_Seq	CHAC2	0.0426	-1.0990	Down
BHGZD *vs.* AIA-M_mRNA_Seq	CHCHD2	0.0033	-1.1468	Down
BHGZD *vs.* AIA-M_mRNA_Seq	CHCHD7	0.0224	-1.1458	Down
BHGZD *vs.* AIA-M_mRNA_Seq	CHMP4B	0.0028	-1.3198	Down
BHGZD *vs.* AIA-M_mRNA_Seq	CHMP4BL1	0.0008	-1.4679	Down
BHGZD *vs.* AIA-M_mRNA_Seq	CHN1	0.0053	-1.3082	Down
BHGZD *vs.* AIA-M_mRNA_Seq	CHRDL1	0.0001	-1.4986	Down
BHGZD *vs.* AIA-M_mRNA_Seq	CHST2	0.0008	-1.1119	Down
BHGZD *vs.* AIA-M_mRNA_Seq	CHURC1	0.0007	-1.4608	Down
BHGZD *vs.* AIA-M_mRNA_Seq	CIB2	0.0423	-3.6097	Down
BHGZD *vs.* AIA-M_mRNA_Seq	CISH	0.0000	-1.5474	Down
BHGZD *vs.* AIA-M_mRNA_Seq	CKAP2L	0.0357	-1.6157	Down
BHGZD *vs.* AIA-M_mRNA_Seq	CLDN7	0.0405	-5.9825	Down
BHGZD *vs.* AIA-M_mRNA_Seq	CLEC1B	0.0001	-1.3748	Down
BHGZD *vs.* AIA-M_mRNA_Seq	CLEC4F	0.0006	-1.3655	Down
BHGZD *vs.* AIA-M_mRNA_Seq	CLIC4	0.0000	-1.3496	Down
BHGZD *vs.* AIA-M_mRNA_Seq	CLIP2	0.0001	-1.1604	Down
BHGZD *vs.* AIA-M_mRNA_Seq	CLNK	0.0257	1.3117	Up
BHGZD *vs.* AIA-M_mRNA_Seq	CLOCK	0.0000	-1.5853	Down
BHGZD *vs.* AIA-M_mRNA_Seq	CLSTN2	0.0301	-3.9501	Down
BHGZD *vs.* AIA-M_mRNA_Seq	CLU	0.0000	-1.4820	Down

BHGZD *vs.* AIA-M_mRNA_Seq	CMAS	0.0082	-1.0558	Down
BHGZD *vs.* AIA-M_mRNA_Seq	CMC2	0.0003	-1.5774	Down
BHGZD *vs.* AIA-M_mRNA_Seq	CMPK1	0.0001	-1.0229	Down
BHGZD *vs.* AIA-M_mRNA_Seq	CMPK2	0.0100	-1.1930	Down
BHGZD *vs.* AIA-M_mRNA_Seq	CMTM4	0.0063	-1.4590	Down
BHGZD *vs.* AIA-M_mRNA_Seq	CNIH2	0.0018	-1.2351	Down
BHGZD *vs.* AIA-M_mRNA_Seq	CNPPD1	0.0072	-1.0427	Down
BHGZD *vs.* AIA-M_mRNA_Seq	CNST	0.0002	-1.1812	Down
BHGZD *vs.* AIA-M_mRNA_Seq	COL4A1	0.0051	-2.1515	Down
BHGZD *vs.* AIA-M_mRNA_Seq	COL4A4	0.0006	-1.2211	Down
BHGZD *vs.* AIA-M_mRNA_Seq	COX4I1	0.0012	-1.4314	Down
BHGZD *vs.* AIA-M_mRNA_Seq	COX6A1	0.0014	-1.3820	Down
BHGZD *vs.* AIA-M_mRNA_Seq	COX6B1	0.0040	-1.2271	Down
BHGZD *vs.* AIA-M_mRNA_Seq	COX7A2//COX7A2L2	0.0026	-1.2957	Down
BHGZD *vs.* AIA-M_mRNA_Seq	COX7A2L	0.0028	-1.1900	Down
BHGZD *vs.* AIA-M_mRNA_Seq	COX7B	0.0044	-1.3038	Down
BHGZD *vs.* AIA-M_mRNA_Seq	COX8A	0.0018	-1.3801	Down
BHGZD *vs.* AIA-M_mRNA_Seq	CPA1	0.0115	-3.0935	Down
BHGZD *vs.* AIA-M_mRNA_Seq	CPQ	0.0000	-1.6821	Down
BHGZD *vs.* AIA-M_mRNA_Seq	CRABP1	0.0081	-1.9772	Down
BHGZD *vs.* AIA-M_mRNA_Seq	CRABP2//LOC100911902	0.0101	3.8683	Up
BHGZD *vs.* AIA-M_mRNA_Seq	CRACR2B//LOC100911692	0.0256	-1.8030	Down
BHGZD *vs.* AIA-M_mRNA_Seq	CREG1	0.0049	-1.3873	Down
BHGZD *vs.* AIA-M_mRNA_Seq	CRIM1	0.0009	-1.1336	Down
BHGZD *vs.* AIA-M_mRNA_Seq	CSDC2	0.0151	-1.4032	Down

BHGZD *vs.* AIA-M_mRNA_Seq	CSGALNACT1	0.0001	-1.3017	Down
BHGZD *vs.* AIA-M_mRNA_Seq	CST3	0.0001	-1.3479	Down
BHGZD *vs.* AIA-M_mRNA_Seq	CTBP1	0.0001	-1.0837	Down
BHGZD *vs.* AIA-M_mRNA_Seq	CTDSPL	0.0089	-1.0297	Down
BHGZD *vs.* AIA-M_mRNA_Seq	CTRB1	0.0001	-1.8123	Down
BHGZD *vs.* AIA-M_mRNA_Seq	CTSB	0.0057	-1.2418	Down
BHGZD *vs.* AIA-M_mRNA_Seq	CTSD	0.0048	-1.1633	Down
BHGZD *vs.* AIA-M_mRNA_Seq	CTTN	0.0000	-1.9100	Down
BHGZD *vs.* AIA-M_mRNA_Seq	CTTNBP2NL	0.0004	-1.4626	Down
BHGZD *vs.* AIA-M_mRNA_Seq	CUX1	0.0028	-1.1724	Down
BHGZD *vs.* AIA-M_mRNA_Seq	CXCL2	0.0001	-1.2825	Down
BHGZD *vs.* AIA-M_mRNA_Seq	CXXC4	0.0000	-1.5581	Down
BHGZD *vs.* AIA-M_mRNA_Seq	CYB5R4	0.0001	-1.0035	Down
BHGZD *vs.* AIA-M_mRNA_Seq	CYP27A1	0.0346	-1.9802	Down
BHGZD *vs.* AIA-M_mRNA_Seq	CYP2J4	0.0477	-1.7076	Down
BHGZD *vs.* AIA-M_mRNA_Seq	DACT1	0.0059	-2.7562	Down
BHGZD *vs.* AIA-M_mRNA_Seq	DAND5	0.0001	-1.8524	Down
BHGZD *vs.* AIA-M_mRNA_Seq	DAP	0.0083	-1.1021	Down
BHGZD *vs.* AIA-M_mRNA_Seq	DAPK2	0.0025	-1.2747	Down
BHGZD *vs.* AIA-M_mRNA_Seq	DAZL	0.0355	-4.5690	Down
BHGZD *vs.* AIA-M_mRNA_Seq	DCTN2	0.0017	-1.3521	Down
BHGZD *vs.* AIA-M_mRNA_Seq	DCTPP1	0.0043	-1.8575	Down
BHGZD *vs.* AIA-M_mRNA_Seq	DCUN1D1	0.0056	-1.0872	Down
BHGZD *vs.* AIA-M_mRNA_Seq	DDX25	0.0077	2.9352	Up
BHGZD *vs.* AIA-M_mRNA_Seq	DENND2C	0.0006	-1.3200	Down
BHGZD *vs.* AIA-M_mRNA_Seq	DHDH	0.0211	1.0557	Up
BHGZD *vs.* AIA-M_mRNA_Seq	DHRS11	0.0012	-1.4918	Down

BHGZD *vs.* AIA-M_mRNA_Seq	DMTN	0.0043	-1.2073	Down
BHGZD *vs.* AIA-M_mRNA_Seq	DNAH1	0.0002	-1.6980	Down
BHGZD *vs.* AIA-M_mRNA_Seq	DNAH6	0.0438	2.4776	Up
BHGZD *vs.* AIA-M_mRNA_Seq	DNAJA4	0.0002	-1.1152	Down
BHGZD *vs.* AIA-M_mRNA_Seq	DNAJB12	0.0008	-1.3841	Down
BHGZD *vs.* AIA-M_mRNA_Seq	DNAJB2	0.0007	-1.4800	Down
BHGZD *vs.* AIA-M_mRNA_Seq	DNASE1L2	0.0083	-5.5664	Down
BHGZD *vs.* AIA-M_mRNA_Seq	DOCK4	0.0115	1.1826	Up
BHGZD *vs.* AIA-M_mRNA_Seq	DOK4	0.0079	-1.2531	Down
BHGZD *vs.* AIA-M_mRNA_Seq	DPF3	0.0034	-1.6315	Down
BHGZD *vs.* AIA-M_mRNA_Seq	DPH3	0.0056	-1.0536	Down
BHGZD *vs.* AIA-M_mRNA_Seq	DUSP8	0.0346	-3.3416	Down
BHGZD *vs.* AIA-M_mRNA_Seq	DYNLL1	0.0005	-1.4503	Down
BHGZD *vs.* AIA-M_mRNA_Seq	DYNLL2	0.0003	-1.0204	Down
BHGZD *vs.* AIA-M_mRNA_Seq	DYNLRB1	0.0035	-1.2032	Down
BHGZD *vs.* AIA-M_mRNA_Seq	E2F2	0.0026	-1.2240	Down
BHGZD *vs.* AIA-M_mRNA_Seq	ECE2	0.0000	-7.0144	Down
BHGZD *vs.* AIA-M_mRNA_Seq	ECM1	0.0049	-1.0249	Down
BHGZD *vs.* AIA-M_mRNA_Seq	EEF1D	0.0021	-1.1956	Down
BHGZD *vs.* AIA-M_mRNA_Seq	EEF1E1	0.0176	-1.0218	Down
BHGZD *vs.* AIA-M_mRNA_Seq	EGF//LOC100910178	0.0040	4.1399	Up
BHGZD *vs.* AIA-M_mRNA_Seq	EGLN3	0.0033	-1.0173	Down
BHGZD *vs.* AIA-M_mRNA_Seq	EHD2	0.0001	-1.7746	Down
BHGZD *vs.* AIA-M_mRNA_Seq	EIF1	0.0007	-1.3649	Down
BHGZD *vs.* AIA-M_mRNA_Seq	EIF3H//LOC10091111 0//LOC108348062	0.0015	-1.3678	Down
BHGZD *vs.* AIA-M_mRNA_Seq	EIF4E2	0.0039	-1.0733	Down

BHGZD *vs.* AIA-M_mRNA_Seq	ELL2	0.0040	-1.3275	Down
BHGZD *vs.* AIA-M_mRNA_Seq	ELN	0.0005	-6.1763	Down
BHGZD *vs.* AIA-M_mRNA_Seq	ELOB	0.0008	-1.5477	Down
BHGZD *vs.* AIA-M_mRNA_Seq	EMB	0.0190	-1.0280	Down
BHGZD *vs.* AIA-M_mRNA_Seq	ENY2	0.0030	-1.1007	Down
BHGZD *vs.* AIA-M_mRNA_Seq	EPB41	0.0190	-1.0083	Down
BHGZD *vs.* AIA-M_mRNA_Seq	EPB42	0.0072	-1.3664	Down
BHGZD *vs.* AIA-M_mRNA_Seq	EPDR1	0.0014	-1.4755	Down
BHGZD *vs.* AIA-M_mRNA_Seq	EPN1	0.0008	-1.4475	Down
BHGZD *vs.* AIA-M_mRNA_Seq	EPOR	0.0013	-1.6470	Down
BHGZD *vs.* AIA-M_mRNA_Seq	ERFE	0.0101	-2.7202	Down
BHGZD *vs.* AIA-M_mRNA_Seq	ERI2	0.0337	1.0330	Up
BHGZD *vs.* AIA-M_mRNA_Seq	ERMAP	0.0019	-1.6329	Down
BHGZD *vs.* AIA-M_mRNA_Seq	ESAM	0.0000	-1.6122	Down
BHGZD *vs.* AIA-M_mRNA_Seq	ESPNL	0.0197	-3.5269	Down
BHGZD *vs.* AIA-M_mRNA_Seq	EXOSC5	0.0011	-1.3540	Down
BHGZD *vs.* AIA-M_mRNA_Seq	F11R	0.0000	-1.5369	Down
BHGZD *vs.* AIA-M_mRNA_Seq	F13A1	0.0000	-1.3791	Down
BHGZD *vs.* AIA-M_mRNA_Seq	F2RL2	0.0002	-1.0784	Down
BHGZD *vs.* AIA-M_mRNA_Seq	F2RL3	0.0000	-1.5841	Down
BHGZD *vs.* AIA-M_mRNA_Seq	F5	0.0000	-1.2335	Down
BHGZD *vs.* AIA-M_mRNA_Seq	FADS1	0.0049	-1.1669	Down
BHGZD *vs.* AIA-M_mRNA_Seq	FADS2	0.0048	-1.6028	Down
BHGZD *vs.* AIA-M_mRNA_Seq	FAM104A	0.0009	-1.5021	Down
BHGZD *vs.* AIA-M_mRNA_Seq	FAM104B	0.0062	-1.1555	Down
BHGZD *vs.* AIA-M_mRNA_Seq	FAM110B	0.0329	-2.5418	Down
BHGZD *vs.* AIA-M_mRNA_Seq	FAM111A	0.0027	-1.3945	Down

BHGZD *vs.* AIA-M_mRNA_Seq	FAM117A	0.0007	-1.4519	Down
BHGZD *vs.* AIA-M_mRNA_Seq	FAM167A	0.0403	3.7237	Up
BHGZD *vs.* AIA-M_mRNA_Seq	FAM184A	0.0007	-1.5781	Down
BHGZD *vs.* AIA-M_mRNA_Seq	FAM189A2	0.0041	-1.9042	Down
BHGZD *vs.* AIA-M_mRNA_Seq	FAM214B	0.0067	-1.1506	Down
BHGZD *vs.* AIA-M_mRNA_Seq	FAM220A	0.0017	-1.3480	Down
BHGZD *vs.* AIA-M_mRNA_Seq	FAM228A	0.0078	-1.7052	Down
BHGZD *vs.* AIA-M_mRNA_Seq	FAM229A	0.0392	-1.1223	Down
BHGZD *vs.* AIA-M_mRNA_Seq	FAM57A	0.0001	-1.1605	Down
BHGZD *vs.* AIA-M_mRNA_Seq	FAM57B	0.0027	-1.7906	Down
BHGZD *vs.* AIA-M_mRNA_Seq	FAM89B	0.0002	-1.8017	Down
BHGZD *vs.* AIA-M_mRNA_Seq	FAM92A	0.0047	-1.3112	Down
BHGZD *vs.* AIA-M_mRNA_Seq	FARP2	0.0179	-1.1698	Down
BHGZD *vs.* AIA-M_mRNA_Seq	FAU//LOC687780	0.0005	-1.6260	Down
BHGZD *vs.* AIA-M_mRNA_Seq	FBLL1	0.0293	3.8346	Up
BHGZD *vs.* AIA-M_mRNA_Seq	FBLN5	0.0352	-3.6841	Down
BHGZD *vs.* AIA-M_mRNA_Seq	FBXO7	0.0092	-1.0531	Down
BHGZD *vs.* AIA-M_mRNA_Seq	FCRL1	0.0039	1.0027	Up
BHGZD *vs.* AIA-M_mRNA_Seq	FECH	0.0039	-1.4115	Down
BHGZD *vs.* AIA-M_mRNA_Seq	FGD6	0.0256	1.6158	Up
BHGZD *vs.* AIA-M_mRNA_Seq	FHL1	0.0000	-1.4704	Down
BHGZD *vs.* AIA-M_mRNA_Seq	FMO3	0.0223	-6.4066	Down
BHGZD *vs.* AIA-M_mRNA_Seq	FN3K	0.0053	-1.2664	Down
BHGZD *vs.* AIA-M_mRNA_Seq	FN3KRP	0.0057	-1.4223	Down
BHGZD *vs.* AIA-M_mRNA_Seq	FNTB	0.0020	-1.0347	Down
BHGZD *vs.* AIA-M_mRNA_Seq	FOXO4	0.0063	-1.1047	Down
BHGZD *vs.* AIA-M_mRNA_Seq	FPR3	0.0000	1.0909	Up

BHGZD *vs.* AIA-M_mRNA_Seq	FTH1//LOC679217//LOC689130	0.0005	-1.3350	Down
BHGZD *vs.* AIA-M_mRNA_Seq	FTL1	0.0002	-1.6822	Down
BHGZD *vs.* AIA-M_mRNA_Seq	FXN	0.0026	-1.3956	Down
BHGZD *vs.* AIA-M_mRNA_Seq	FZD3	0.0085	-1.3375	Down
BHGZD *vs.* AIA-M_mRNA_Seq	FZR1	0.0033	-1.3592	Down
BHGZD *vs.* AIA-M_mRNA_Seq	G6PC3	0.0002	-1.1740	Down
BHGZD *vs.* AIA-M_mRNA_Seq	GABARAP	0.0043	-1.1779	Down
BHGZD *vs.* AIA-M_mRNA_Seq	GABARAPL2//LOC100359937	0.0018	-1.4343	Down
BHGZD *vs.* AIA-M_mRNA_Seq	GADD45G	0.0309	-1.1012	Down
BHGZD *vs.* AIA-M_mRNA_Seq	GALNT10	0.0042	-1.1517	Down
BHGZD *vs.* AIA-M_mRNA_Seq	GAS2L1	0.0001	-1.4515	Down
BHGZD *vs.* AIA-M_mRNA_Seq	GAS6	0.0265	-1.2679	Down
BHGZD *vs.* AIA-M_mRNA_Seq	GATA1//LOC108348091	0.0075	-1.1711	Down
BHGZD *vs.* AIA-M_mRNA_Seq	GATA1//NEWGENE_2663//LOC108348091	0.0015	-1.5923	Down
BHGZD *vs.* AIA-M_mRNA_Seq	GATAD1	0.0001	-1.1379	Down
BHGZD *vs.* AIA-M_mRNA_Seq	GCH1	0.0071	-1.1798	Down
BHGZD *vs.* AIA-M_mRNA_Seq	GCLM	0.0022	-1.4144	Down
BHGZD *vs.* AIA-M_mRNA_Seq	GDPD2	0.0003	-1.3230	Down
BHGZD *vs.* AIA-M_mRNA_Seq	GFI1B	0.0003	-1.4703	Down
BHGZD *vs.* AIA-M_mRNA_Seq	GLO1	0.0058	-1.0078	Down
BHGZD *vs.* AIA-M_mRNA_Seq	GLOD5	0.0021	-1.3246	Down
BHGZD *vs.* AIA-M_mRNA_Seq	GLRX5	0.0011	-1.5008	Down
BHGZD *vs.* AIA-M_mRNA_Seq	GLS2	0.0452	-5.1076	Down

BHGZD *vs.* AIA-M_mRNA_Seq	GLTP	0.0035	-1.2329	Down
BHGZD *vs.* AIA-M_mRNA_Seq	GLUL	0.0007	-1.5261	Down
BHGZD *vs.* AIA-M_mRNA_Seq	GM5471	0.0013	-1.3928	Down
BHGZD *vs.* AIA-M_mRNA_Seq	GMPR	0.0000	-1.4888	Down
BHGZD *vs.* AIA-M_mRNA_Seq	GNA12	0.0001	-1.3569	Down
BHGZD *vs.* AIA-M_mRNA_Seq	GNAS	0.0032	-1.3305	Down
BHGZD *vs.* AIA-M_mRNA_Seq	GNAZ	0.0000	-1.4201	Down
BHGZD *vs.* AIA-M_mRNA_Seq	GNG11	0.0002	-1.4213	Down
BHGZD *vs.* AIA-M_mRNA_Seq	GNG12	0.0148	-1.0619	Down
BHGZD *vs.* AIA-M_mRNA_Seq	GP1BA	0.0001	-1.3329	Down
BHGZD *vs.* AIA-M_mRNA_Seq	GP1BB	0.0431	-1.5758	Down
BHGZD *vs.* AIA-M_mRNA_Seq	GP5//LOC100911551//NEWGENE_2724	0.0000	-1.5275	Down
BHGZD *vs.* AIA-M_mRNA_Seq	GP5//NEWGENE_2724	0.0000	-1.5289	Down
BHGZD *vs.* AIA-M_mRNA_Seq	GP6	0.0000	-1.4377	Down
BHGZD *vs.* AIA-M_mRNA_Seq	GP9	0.0002	-1.4034	Down
BHGZD *vs.* AIA-M_mRNA_Seq	GPT	0.0426	-1.4390	Down
BHGZD *vs.* AIA-M_mRNA_Seq	GPX1	0.0178	-1.1503	Down
BHGZD *vs.* AIA-M_mRNA_Seq	GPX3	0.0001	-1.5102	Down
BHGZD *vs.* AIA-M_mRNA_Seq	GPX4	0.0022	-1.4112	Down
BHGZD *vs.* AIA-M_mRNA_Seq	GRB10	0.0028	-1.3209	Down
BHGZD *vs.* AIA-M_mRNA_Seq	GRINA	0.0015	-1.3563	Down
BHGZD *vs.* AIA-M_mRNA_Seq	GRM5	0.0141	-4.2790	Down
BHGZD *vs.* AIA-M_mRNA_Seq	GSN	0.0000	-1.3103	Down
BHGZD *vs.* AIA-M_mRNA_Seq	GSTA1//LOC108348061	0.0008	-1.3929	Down

BHGZD *vs.* AIA-M_mRNA_Seq	GSTA4	0.0013	-1.4648	Down
BHGZD *vs.* AIA-M_mRNA_Seq	GSTM5//GSTM3	0.0054	-1.5083	Down
BHGZD *vs.* AIA-M_mRNA_Seq	GSTP1	0.0002	-1.2988	Down
BHGZD *vs.* AIA-M_mRNA_Seq	GSTT3	0.0026	-1.6639	Down
BHGZD *vs.* AIA-M_mRNA_Seq	GUCY1A1	0.0000	-1.4963	Down
BHGZD *vs.* AIA-M_mRNA_Seq	GUCY1B1	0.0001	-1.3631	Down
BHGZD *vs.* AIA-M_mRNA_Seq	H2AFJ	0.0009	-1.3426	Down
BHGZD *vs.* AIA-M_mRNA_Seq	HAGH	0.0011	-1.5646	Down
BHGZD *vs.* AIA-M_mRNA_Seq	HAUS4	0.0065	-1.3259	Down
BHGZD *vs.* AIA-M_mRNA_Seq	HBA1	0.0001	-1.7903	Down
BHGZD *vs.* AIA-M_mRNA_Seq	HBA-A1	0.0002	-1.7830	Down
BHGZD *vs.* AIA-M_mRNA_Seq	HBA-A2	0.0001	-1.7705	Down
BHGZD *vs.* AIA-M_mRNA_Seq	HBB	0.0002	-1.8271	Down
BHGZD *vs.* AIA-M_mRNA_Seq	HBB-B1//LOC689064	0.0001	-1.7855	Down
BHGZD *vs.* AIA-M_mRNA_Seq	HBE1	0.0240	-1.2836	Down
BHGZD *vs.* AIA-M_mRNA_Seq	HBQ1B	0.0032	-1.4137	Down
BHGZD *vs.* AIA-M_mRNA_Seq	HCFC1R1	0.0012	-1.3137	Down
BHGZD *vs.* AIA-M_mRNA_Seq	HDAC9	0.0009	1.0010	Up
BHGZD *vs.* AIA-M_mRNA_Seq	HDHD3	0.0097	4.4040	Up
BHGZD *vs.* AIA-M_mRNA_Seq	HEMGN	0.0148	-1.2433	Down
BHGZD *vs.* AIA-M_mRNA_Seq	HEXA	0.0045	-1.1952	Down
BHGZD *vs.* AIA-M_mRNA_Seq	HINT1	0.0017	-1.4041	Down
BHGZD *vs.* AIA-M_mRNA_Seq	HIST1H1D	0.0009	-1.2821	Down
BHGZD *vs.* AIA-M_mRNA_Seq	HIST1H2AC//HIST1H2AN//HIST1H2AI//LOC684773//LOC100360754	0.0013	-1.4459	Down

BHGZD *vs.* AIA-M_mRNA_Seq	HIST1H2AF	0.0018	-1.2342	Down
BHGZD *vs.* AIA-M_mRNA_Seq	HIST1H2AH//LOC103690190	0.0003	-1.2617	Down
BHGZD *vs.* AIA-M_mRNA_Seq	HIST1H2AK	0.0277	-1.5185	Down
BHGZD *vs.* AIA-M_mRNA_Seq	HIST1H2AN	0.0240	-1.4373	Down
BHGZD *vs.* AIA-M_mRNA_Seq	HIST1H2AO//HIST2H4A//HIST1H4A//LOC100912564	0.0040	-1.9358	Down
BHGZD *vs.* AIA-M_mRNA_Seq	HIST1H2AO//HIST2H4A//HIST4H4//LOC100912564	0.0169	-1.7315	Down
BHGZD *vs.* AIA-M_mRNA_Seq	HIST1H2AO//HIST2H4A//LOC100912564	0.0001	-1.4359	Down
BHGZD *vs.* AIA-M_mRNA_Seq	HIST1H2AO//HIST2H4A//LOC100912564//LOC102548682	0.0009	-1.5703	Down
BHGZD *vs.* AIA-M_mRNA_Seq	HIST1H2AO//RGD1562378//HIST2H4A//LOC100912564	0.0081	-1.2207	Down
BHGZD *vs.* AIA-M_mRNA_Seq	HIST1H2BD	0.0080	-1.5297	Down
BHGZD *vs.* AIA-M_mRNA_Seq	HIST1H2BH//HIST1H2BC//LOC684797//LOC100364835//LOC100365043//LOC100910200	0.0001	-1.5220	Down
BHGZD *vs.* AIA-M_mRNA_Seq	HIST1H2BK	0.0429	-1.6367	Down
BHGZD *vs.* AIA-M_mRNA_Seq	HIST1H2BO	0.0019	-1.6254	Down

BHGZD *vs.* AIA-M_mRNA_Seq	HIST1H3A//LOC680498//LOC682330	0.0014	-1.6275	Down
BHGZD *vs.* AIA-M_mRNA_Seq	HIST1H4B//HIST1H2AO//HIST2H4A//LOC100912564	0.0008	-1.5210	Down
BHGZD *vs.* AIA-M_mRNA_Seq	HIST2H2AA3//HIST2H2AA2	0.0027	-1.0934	Down
BHGZD *vs.* AIA-M_mRNA_Seq	HIST2H2AC	0.0006	-2.1036	Down
BHGZD *vs.* AIA-M_mRNA_Seq	HIST2H4//HIST1H2AO//HIST2H4A//LOC100912564	0.0020	-2.1042	Down
BHGZD *vs.* AIA-M_mRNA_Seq	HIST3H2BA	0.0009	-1.5433	Down
BHGZD *vs.* AIA-M_mRNA_Seq	HIST3H2BB	0.0060	-1.4021	Down
BHGZD *vs.* AIA-M_mRNA_Seq	HIVEP3	0.0267	1.0399	Up
BHGZD *vs.* AIA-M_mRNA_Seq	HMBS	0.0012	-1.4293	Down
BHGZD *vs.* AIA-M_mRNA_Seq	HMG20B	0.0065	-1.0252	Down
BHGZD *vs.* AIA-M_mRNA_Seq	HNF4A	0.0066	3.9182	Up
BHGZD *vs.* AIA-M_mRNA_Seq	HPSE	0.0000	-1.3065	Down
BHGZD *vs.* AIA-M_mRNA_Seq	HSCB	0.0040	-1.3514	Down
BHGZD *vs.* AIA-M_mRNA_Seq	HSD17B14	0.0006	-1.5480	Down
BHGZD *vs.* AIA-M_mRNA_Seq	HYAL3	0.0010	-1.1748	Down
BHGZD *vs.* AIA-M_mRNA_Seq	HYI	0.0024	-1.4680	Down
BHGZD *vs.* AIA-M_mRNA_Seq	HYPK	0.0020	-1.3888	Down
BHGZD *vs.* AIA-M_mRNA_Seq	ICAM4	0.0046	-1.7062	Down
BHGZD *vs.* AIA-M_mRNA_Seq	IFRD2	0.0047	-1.2586	Down
BHGZD *vs.* AIA-M_mRNA_Seq	IFT22	0.0003	4.1572	Up
BHGZD *vs.* AIA-M_mRNA_Seq	IFT27	0.0010	-1.6092	Down

BHGZD *vs.* AIA-M_mRNA_Seq	IFT46	0.0116	-1.0311	Down
BHGZD *vs.* AIA-M_mRNA_Seq	IFT57	0.0044	-1.2747	Down
BHGZD *vs.* AIA-M_mRNA_Seq	IGF2	0.0432	2.5178	Up
BHGZD *vs.* AIA-M_mRNA_Seq	IGSF10	0.0001	-1.4300	Down
BHGZD *vs.* AIA-M_mRNA_Seq	ILK	0.0001	-1.1246	Down
BHGZD *vs.* AIA-M_mRNA_Seq	INO80C	0.0023	-1.1134	Down
BHGZD *vs.* AIA-M_mRNA_Seq	INPP5A	0.0000	-1.1716	Down
BHGZD *vs.* AIA-M_mRNA_Seq	ISCA1	0.0006	-1.5907	Down
BHGZD *vs.* AIA-M_mRNA_Seq	ISCA2	0.0064	-1.2692	Down
BHGZD *vs.* AIA-M_mRNA_Seq	ISCU	0.0025	-1.4229	Down
BHGZD *vs.* AIA-M_mRNA_Seq	ISG20	0.0012	-1.5247	Down
BHGZD *vs.* AIA-M_mRNA_Seq	ITGA11	0.0002	-1.4992	Down
BHGZD *vs.* AIA-M_mRNA_Seq	ITGA2B	0.0000	-1.5017	Down
BHGZD *vs.* AIA-M_mRNA_Seq	ITGA6	0.0000	-1.4334	Down
BHGZD *vs.* AIA-M_mRNA_Seq	ITGB3	0.0000	-1.3277	Down
BHGZD *vs.* AIA-M_mRNA_Seq	ITM2B	0.0021	-1.0853	Down
BHGZD *vs.* AIA-M_mRNA_Seq	ITSN1	0.0294	-1.1643	Down
BHGZD *vs.* AIA-M_mRNA_Seq	JAZF1	0.0002	-1.3709	Down
BHGZD *vs.* AIA-M_mRNA_Seq	KALRN	0.0015	-1.7831	Down
BHGZD *vs.* AIA-M_mRNA_Seq	KATNAL1//LOC100910196//LOC103690050	0.0069	-1.2866	Down
BHGZD *vs.* AIA-M_mRNA_Seq	KCNAB1	0.0000	-1.3601	Down
BHGZD *vs.* AIA-M_mRNA_Seq	KCNS3	0.0003	-1.4733	Down
BHGZD *vs.* AIA-M_mRNA_Seq	KDR	0.0005	-2.0126	Down
BHGZD *vs.* AIA-M_mRNA_Seq	KEL	0.0020	-1.5788	Down
BHGZD *vs.* AIA-M_mRNA_Seq	KIF18B	0.0155	-1.5961	Down
BHGZD *vs.* AIA-M_mRNA_Seq	KIF21A	0.0084	-1.3138	Down

BHGZD *vs.* AIA-M_mRNA_Seq	KIF2A	0.0000	-1.1164	Down
BHGZD *vs.* AIA-M_mRNA_Seq	KIF4A	0.0080	-1.5340	Down
BHGZD *vs.* AIA-M_mRNA_Seq	KLF1	0.0020	-1.4512	Down
BHGZD *vs.* AIA-M_mRNA_Seq	KLHL13	0.0009	-2.3771	Down
BHGZD *vs.* AIA-M_mRNA_Seq	KLHL42	0.0080	-1.0737	Down
BHGZD *vs.* AIA-M_mRNA_Seq	KLRA22	0.0175	1.6770	Up
BHGZD *vs.* AIA-M_mRNA_Seq	KNG2//KNG2L1//KNG1	0.0152	-4.9917	Down
BHGZD *vs.* AIA-M_mRNA_Seq	KNSTRN	0.0254	-1.1195	Down
BHGZD *vs.* AIA-M_mRNA_Seq	KRT71	0.0056	-1.3524	Down
BHGZD *vs.* AIA-M_mRNA_Seq	LACC1	0.0242	1.0089	Up
BHGZD *vs.* AIA-M_mRNA_Seq	LANCL3	0.0302	-1.9667	Down
BHGZD *vs.* AIA-M_mRNA_Seq	LARGE1	0.0001	-1.4699	Down
BHGZD *vs.* AIA-M_mRNA_Seq	LCORL	0.0396	1.4755	Up
BHGZD *vs.* AIA-M_mRNA_Seq	LEFTY2	0.0426	4.3278	Up
BHGZD *vs.* AIA-M_mRNA_Seq	LEKR1	0.0126	2.1711	Up
BHGZD *vs.* AIA-M_mRNA_Seq	LGALS2	0.0046	-1.1216	Down
BHGZD *vs.* AIA-M_mRNA_Seq	LGALS5	0.0021	-1.4455	Down
BHGZD *vs.* AIA-M_mRNA_Seq	LGALSL	0.0000	-1.4054	Down
BHGZD *vs.* AIA-M_mRNA_Seq	LHFPL6	0.0290	-1.2276	Down
BHGZD *vs.* AIA-M_mRNA_Seq	LIMS1	0.0001	-1.0388	Down
BHGZD *vs.* AIA-M_mRNA_Seq	LIPG	0.0134	1.3060	Up
BHGZD *vs.* AIA-M_mRNA_Seq	LMLN2	0.0242	3.9893	Up
BHGZD *vs.* AIA-M_mRNA_Seq	LMNA	0.0006	-1.3151	Down
BHGZD *vs.* AIA-M_mRNA_Seq	LOC100134871	0.0004	-1.6823	Down
BHGZD *vs.* AIA-M_mRNA_Seq	LOC100359505	0.0009	-1.4242	Down
BHGZD *vs.* AIA-M_mRNA_Seq	LOC100359668	0.0018	-1.4674	Down

BHGZD *vs.* AIA-M_mRNA_Seq	LOC100359922//LOC100360491//LOC100361259//LOC100362400	0.0001	-1.3456	Down
BHGZD *vs.* AIA-M_mRNA_Seq	LOC100359951	0.0016	-1.3311	Down
BHGZD *vs.* AIA-M_mRNA_Seq	LOC100359951//LOC100362149//LOC100362684	0.0028	-1.3161	Down
BHGZD *vs.* AIA-M_mRNA_Seq	LOC100360087	0.0005	-1.6050	Down
BHGZD *vs.* AIA-M_mRNA_Seq	LOC100360491	0.0003	-1.4071	Down
BHGZD *vs.* AIA-M_mRNA_Seq	LOC100360573	0.0014	-1.5465	Down
BHGZD *vs.* AIA-M_mRNA_Seq	LOC100360781	0.0013	-1.4733	Down
BHGZD *vs.* AIA-M_mRNA_Seq	LOC100360791	0.0003	-1.6598	Down
BHGZD *vs.* AIA-M_mRNA_Seq	LOC100361061	0.0015	-1.2138	Down
BHGZD *vs.* AIA-M_mRNA_Seq	LOC100361180//LOC100362366//LOC100365810	0.0045	-1.1100	Down
BHGZD *vs.* AIA-M_mRNA_Seq	LOC100361259	0.0040	-1.3064	Down
BHGZD *vs.* AIA-M_mRNA_Seq	LOC100361756	0.0046	-1.5654	Down
BHGZD *vs.* AIA-M_mRNA_Seq	LOC100361933	0.0042	-1.5045	Down
BHGZD *vs.* AIA-M_mRNA_Seq	LOC100362027	0.0039	-1.1055	Down
BHGZD *vs.* AIA-M_mRNA_Seq	LOC100362339	0.0005	-1.5781	Down
BHGZD *vs.* AIA-M_mRNA_Seq	LOC100362384	0.0003	-1.6340	Down
BHGZD *vs.* AIA-M_mRNA_Seq	LOC100362400	0.0005	-1.2584	Down
BHGZD *vs.* AIA-M_mRNA_Seq	LOC100362583	0.0021	-1.1921	Down
BHGZD *vs.* AIA-M_mRNA_Seq	LOC100362684	0.0016	-1.4161	Down
BHGZD *vs.* AIA-M_mRNA_Seq	LOC100362751	0.0027	-1.4120	Down

BHGZD *vs.* AIA-M_mRNA_Seq	LOC100362830	0.0012	-1.2188	Down
BHGZD *vs.* AIA-M_mRNA_Seq	LOC100363452	0.0016	-1.2069	Down
BHGZD *vs.* AIA-M_mRNA_Seq	LOC100363469	0.0027	-1.2715	Down
BHGZD *vs.* AIA-M_mRNA_Seq	LOC100363537	0.0161	-1.6731	Down
BHGZD *vs.* AIA-M_mRNA_Seq	LOC100364116	0.0154	-1.0954	Down
BHGZD *vs.* AIA-M_mRNA_Seq	LOC100365450	0.0213	-4.7931	Down
BHGZD *vs.* AIA-M_mRNA_Seq	LOC100365839	0.0014	-1.2746	Down
BHGZD *vs.* AIA-M_mRNA_Seq	LOC100909726	0.0046	-1.1102	Down
BHGZD *vs.* AIA-M_mRNA_Seq	LOC100909857	0.0045	-5.5950	Down
BHGZD *vs.* AIA-M_mRNA_Seq	LOC100909878	0.0072	-1.1188	Down
BHGZD *vs.* AIA-M_mRNA_Seq	LOC100909912	0.0020	-1.3189	Down
BHGZD *vs.* AIA-M_mRNA_Seq	LOC100910650	0.0001	-1.3841	Down
BHGZD *vs.* AIA-M_mRNA_Seq	LOC100910978	0.0351	-2.8138	Down
BHGZD *vs.* AIA-M_mRNA_Seq	LOC100911130	0.0003	-1.7290	Down
BHGZD *vs.* AIA-M_mRNA_Seq	LOC100911371	0.0032	-1.0574	Down
BHGZD *vs.* AIA-M_mRNA_Seq	LOC100911516	0.0016	-1.1967	Down
BHGZD *vs.* AIA-M_mRNA_Seq	LOC100911575	0.0432	-1.4293	Down
BHGZD *vs.* AIA-M_mRNA_Seq	LOC100911674	0.0076	-1.2292	Down
BHGZD *vs.* AIA-M_mRNA_Seq	LOC100911692	0.0059	-1.9962	Down
BHGZD *vs.* AIA-M_mRNA_Seq	LOC100912034	0.0001	-1.4206	Down
BHGZD *vs.* AIA-M_mRNA_Seq	LOC100912228	0.0003	-1.4317	Down
BHGZD *vs.* AIA-M_mRNA_Seq	LOC100912502	0.0020	-1.5997	Down
BHGZD *vs.* AIA-M_mRNA_Seq	LOC102549061	0.0002	-1.8583	Down
BHGZD *vs.* AIA-M_mRNA_Seq	LOC102550036	0.0259	-1.0947	Down
BHGZD *vs.* AIA-M_mRNA_Seq	LOC102555453	0.0005	-1.3503	Down
BHGZD *vs.* AIA-M_mRNA_Seq	LOC102556051	0.0028	-1.4675	Down
BHGZD *vs.* AIA-M_mRNA_Seq	LOC102556092	0.0000	-1.5513	Down

BHGZD *vs.* AIA-M_mRNA_Seq	LOC102556337	0.0006	-1.4855	Down
BHGZD *vs.* AIA-M_mRNA_Seq	LOC103689947	0.0110	-1.0580	Down
BHGZD *vs.* AIA-M_mRNA_Seq	LOC103690002	0.0008	-1.3466	Down
BHGZD *vs.* AIA-M_mRNA_Seq	LOC103690015	0.0049	-1.4168	Down
BHGZD *vs.* AIA-M_mRNA_Seq	LOC103690020	0.0094	-1.2767	Down
BHGZD *vs.* AIA-M_mRNA_Seq	LOC103690068	0.0333	-2.2693	Down
BHGZD *vs.* AIA-M_mRNA_Seq	LOC103690821//LOC103692785//LOC103694404	0.0006	-1.4476	Down
BHGZD *vs.* AIA-M_mRNA_Seq	LOC103690821//LOC103692831//LOC103694404	0.0078	-1.3078	Down
BHGZD *vs.* AIA-M_mRNA_Seq	LOC103690821//LOC103693375//LOC103694404	0.0120	-1.2524	Down
BHGZD *vs.* AIA-M_mRNA_Seq	LOC103690821//LOC103694169//LOC103694404	0.0003	-1.4874	Down
BHGZD *vs.* AIA-M_mRNA_Seq	LOC103690821//LOC103694404	0.0135	-1.2256	Down
BHGZD *vs.* AIA-M_mRNA_Seq	LOC103692169	0.0005	-1.5489	Down
BHGZD *vs.* AIA-M_mRNA_Seq	LOC103692173	0.0010	-1.3119	Down
BHGZD *vs.* AIA-M_mRNA_Seq	LOC103692984	0.0236	2.6544	Up
BHGZD *vs.* AIA-M_mRNA_Seq	LOC103694314	0.0204	-1.1254	Down
BHGZD *vs.* AIA-M_mRNA_Seq	LOC108349606//LOC108353795	0.0047	-1.0499	Down
BHGZD *vs.* AIA-M_mRNA_Seq	LOC108350501	0.0007	-1.6288	Down

BHGZD *vs.* AIA-M_mRNA_Seq	LOC108350501//LOC108351482//LOC108352650	0.0003	-1.5678	Down
BHGZD *vs.* AIA-M_mRNA_Seq	LOC108350502//LOC108353446	0.0006	-1.5240	Down
BHGZD *vs.* AIA-M_mRNA_Seq	LOC108351406	0.0304	-4.1433	Down
BHGZD *vs.* AIA-M_mRNA_Seq	LOC108351482	0.0026	-1.3714	Down
BHGZD *vs.* AIA-M_mRNA_Seq	LOC108352861	0.0154	-1.0067	Down
BHGZD *vs.* AIA-M_mRNA_Seq	LOC498222	0.0078	-1.5868	Down
BHGZD *vs.* AIA-M_mRNA_Seq	LOC498424	0.0275	-1.1075	Down
BHGZD *vs.* AIA-M_mRNA_Seq	LOC498555	0.0004	-1.6347	Down
BHGZD *vs.* AIA-M_mRNA_Seq	LOC679739	0.0439	-2.2732	Down
BHGZD *vs.* AIA-M_mRNA_Seq	LOC680700	0.0174	-1.1102	Down
BHGZD *vs.* AIA-M_mRNA_Seq	LOC680959	0.0049	-2.3372	Down
BHGZD *vs.* AIA-M_mRNA_Seq	LOC681182//LOC681341	0.0000	1.0622	Up
BHGZD *vs.* AIA-M_mRNA_Seq	LOC682793//RPL38//LOC690468	0.0000	-1.7773	Down
BHGZD *vs.* AIA-M_mRNA_Seq	LOC684762//LOC102548682//LOC102549173	0.0131	-1.2133	Down
BHGZD *vs.* AIA-M_mRNA_Seq	LOC684762//LOC684841//LOC102548682//LOC102549173	0.0290	-1.2013	Down
BHGZD *vs.* AIA-M_mRNA_Seq	LOC684762//LOC690126//LOC102548682//LOC102549173	0.0358	-1.5651	Down

BHGZD *vs.* AIA-M_mRNA_Seq	LOC685085	0.0022	-1.1059	Down
BHGZD *vs.* AIA-M_mRNA_Seq	LOC685933//TBC1D1 2	0.0414	1.5340	Up
BHGZD *vs.* AIA-M_mRNA_Seq	LOC685963	0.0000	-1.8704	Down
BHGZD *vs.* AIA-M_mRNA_Seq	LOC688815	0.0133	-1.4655	Down
BHGZD *vs.* AIA-M_mRNA_Seq	LOC689574	0.0004	-1.6061	Down
BHGZD *vs.* AIA-M_mRNA_Seq	LOC689899	0.0070	-1.1045	Down
BHGZD *vs.* AIA-M_mRNA_Seq	LOC691418	0.0235	-1.0361	Down
BHGZD *vs.* AIA-M_mRNA_Seq	LOXL3	0.0002	-1.4840	Down
BHGZD *vs.* AIA-M_mRNA_Seq	LOXL4	0.0433	-5.2438	Down
BHGZD *vs.* AIA-M_mRNA_Seq	LRP4	0.0085	-1.0556	Down
BHGZD *vs.* AIA-M_mRNA_Seq	LRR1	0.0357	-5.3247	Down
BHGZD *vs.* AIA-M_mRNA_Seq	LRRC51	0.0204	-1.2492	Down
BHGZD *vs.* AIA-M_mRNA_Seq	LRRC71	0.0003	-1.5799	Down
BHGZD *vs.* AIA-M_mRNA_Seq	LTBP1	0.0009	-1.3291	Down
BHGZD *vs.* AIA-M_mRNA_Seq	LTK	0.0448	-1.8038	Down
BHGZD *vs.* AIA-M_mRNA_Seq	LY6G6C	0.0000	-1.5001	Down
BHGZD *vs.* AIA-M_mRNA_Seq	LY6G6F	0.0000	-1.4740	Down
BHGZD *vs.* AIA-M_mRNA_Seq	LY6L	0.0321	1.3637	Up
BHGZD *vs.* AIA-M_mRNA_Seq	LYL1	0.0011	-1.3306	Down
BHGZD *vs.* AIA-M_mRNA_Seq	LYVE1	0.0000	-1.3934	Down
BHGZD *vs.* AIA-M_mRNA_Seq	LZTS1	0.0151	1.8058	Up
BHGZD *vs.* AIA-M_mRNA_Seq	MAGED2	0.0001	-1.2126	Down
BHGZD *vs.* AIA-M_mRNA_Seq	MANBAL	0.0085	-1.0353	Down
BHGZD *vs.* AIA-M_mRNA_Seq	MAP1B	0.0102	-1.1763	Down
BHGZD *vs.* AIA-M_mRNA_Seq	MAP1LC3A	0.0013	-1.2683	Down
BHGZD *vs.* AIA-M_mRNA_Seq	MAP1LC3B	0.0006	-1.5919	Down

BHGZD *vs.* AIA-M_mRNA_Seq	MAP1LC3B2	0.0175	-1.4347	Down
BHGZD *vs.* AIA-M_mRNA_Seq	MAP2K3//LOC103689956//LOC108348082	0.0047	-1.1809	Down
BHGZD *vs.* AIA-M_mRNA_Seq	MAP2K3//LOC108348082	0.0368	-1.4296	Down
BHGZD *vs.* AIA-M_mRNA_Seq	MAPK10	0.0155	-1.5761	Down
BHGZD *vs.* AIA-M_mRNA_Seq	MAPK8IP1	0.0016	-2.4659	Down
BHGZD *vs.* AIA-M_mRNA_Seq	MARCH2	0.0005	-1.6508	Down
BHGZD *vs.* AIA-M_mRNA_Seq	MARCH8	0.0098	-1.1514	Down
BHGZD *vs.* AIA-M_mRNA_Seq	MARK1	0.0248	1.0751	Up
BHGZD *vs.* AIA-M_mRNA_Seq	MAST1	0.0481	-3.0588	Down
BHGZD *vs.* AIA-M_mRNA_Seq	MAST4	0.0244	-1.2505	Down
BHGZD *vs.* AIA-M_mRNA_Seq	MB21D2	0.0004	-1.4490	Down
BHGZD *vs.* AIA-M_mRNA_Seq	MBOAT2	0.0312	1.3213	Up
BHGZD *vs.* AIA-M_mRNA_Seq	MCPT1L1	0.0000	-1.7125	Down
BHGZD *vs.* AIA-M_mRNA_Seq	MCRIP1	0.0033	-1.0451	Down
BHGZD *vs.* AIA-M_mRNA_Seq	MCUR1	0.0001	-1.0092	Down
BHGZD *vs.* AIA-M_mRNA_Seq	ME1	0.0000	-1.5397	Down
BHGZD *vs.* AIA-M_mRNA_Seq	MEA1	0.0035	-1.1456	Down
BHGZD *vs.* AIA-M_mRNA_Seq	MED12L	0.0000	-1.4364	Down
BHGZD *vs.* AIA-M_mRNA_Seq	MED25	0.0006	-1.5579	Down
BHGZD *vs.* AIA-M_mRNA_Seq	MELK	0.0439	2.0318	Up
BHGZD *vs.* AIA-M_mRNA_Seq	MEMO1	0.0108	-1.1541	Down
BHGZD *vs.* AIA-M_mRNA_Seq	MEST	0.0007	-1.7263	Down
BHGZD *vs.* AIA-M_mRNA_Seq	MFAP3L	0.0000	-1.5052	Down
BHGZD *vs.* AIA-M_mRNA_Seq	MFF	0.0028	-1.3228	Down
BHGZD *vs.* AIA-M_mRNA_Seq	MFSD12	0.0041	-1.2594	Down

BHGZD *vs.* AIA-M_mRNA_Seq	MFSD2B	0.0101	-1.4997	Down
BHGZD *vs.* AIA-M_mRNA_Seq	MFSD6	0.0000	-1.0233	Down
BHGZD *vs.* AIA-M_mRNA_Seq	MGC94199	0.0195	1.5424	Up
BHGZD *vs.* AIA-M_mRNA_Seq	MGC94199//LOC102554232	0.0216	1.9556	Up
BHGZD *vs.* AIA-M_mRNA_Seq	MGC95210	0.0007	-1.4406	Down
BHGZD *vs.* AIA-M_mRNA_Seq	MGLL	0.0005	-1.6987	Down
BHGZD *vs.* AIA-M_mRNA_Seq	MGRN1	0.0000	-1.2031	Down
BHGZD *vs.* AIA-M_mRNA_Seq	MGST3	0.0027	-1.5163	Down
BHGZD *vs.* AIA-M_mRNA_Seq	MICAL2	0.0019	-1.3556	Down
BHGZD *vs.* AIA-M_mRNA_Seq	MICAL3	0.0004	-1.4663	Down
BHGZD *vs.* AIA-M_mRNA_Seq	MKRN1	0.0017	-1.4175	Down
BHGZD *vs.* AIA-M_mRNA_Seq	MMD	0.0004	-1.2623	Down
BHGZD *vs.* AIA-M_mRNA_Seq	MMP28	0.0211	-1.0628	Down
BHGZD *vs.* AIA-M_mRNA_Seq	MMRN1	0.0000	-1.5474	Down
BHGZD *vs.* AIA-M_mRNA_Seq	MMRN2	0.0015	3.0209	Up
BHGZD *vs.* AIA-M_mRNA_Seq	MORC4	0.0001	-1.4301	Down
BHGZD *vs.* AIA-M_mRNA_Seq	MPG	0.0009	-1.5303	Down
BHGZD *vs.* AIA-M_mRNA_Seq	MPIG6B	0.0001	-1.5224	Down
BHGZD *vs.* AIA-M_mRNA_Seq	MPL	0.0000	-1.5075	Down
BHGZD *vs.* AIA-M_mRNA_Seq	MPP2	0.0387	-1.1260	Down
BHGZD *vs.* AIA-M_mRNA_Seq	MPPED2	0.0003	-1.4039	Down
BHGZD *vs.* AIA-M_mRNA_Seq	MPST	0.0005	-1.7733	Down
BHGZD *vs.* AIA-M_mRNA_Seq	MRPL37	0.0014	-1.3539	Down
BHGZD *vs.* AIA-M_mRNA_Seq	MRPL53	0.0048	-1.1671	Down
BHGZD *vs.* AIA-M_mRNA_Seq	MRPS36	0.0062	-1.1319	Down
BHGZD *vs.* AIA-M_mRNA_Seq	MRVI1	0.0000	-1.4740	Down

BHGZD *vs.* AIA-M_mRNA_Seq	MSRA	0.0301	-1.1511	Down
BHGZD *vs.* AIA-M_mRNA_Seq	MTHFD2	0.0007	-1.0831	Down
BHGZD *vs.* AIA-M_mRNA_Seq	MTMR11	0.0440	3.3129	Up
BHGZD *vs.* AIA-M_mRNA_Seq	MUC20	0.0000	-1.5329	Down
BHGZD *vs.* AIA-M_mRNA_Seq	MXI1	0.0046	-1.1420	Down
BHGZD *vs.* AIA-M_mRNA_Seq	MYBL2	0.0208	-1.2279	Down
BHGZD *vs.* AIA-M_mRNA_Seq	MYCT1	0.0000	-1.5488	Down
BHGZD *vs.* AIA-M_mRNA_Seq	MYL6L//MYL6//MYL6B//LOC100909726	0.0002	-1.2505	Down
BHGZD *vs.* AIA-M_mRNA_Seq	MYL9	0.0000	-1.5114	Down
BHGZD *vs.* AIA-M_mRNA_Seq	MYLK	0.0000	-1.4627	Down
BHGZD *vs.* AIA-M_mRNA_Seq	MYLK2	0.0009	-1.5014	Down
BHGZD *vs.* AIA-M_mRNA_Seq	MYO5C	0.0073	-1.3306	Down
BHGZD *vs.* AIA-M_mRNA_Seq	NABP2	0.0060	-1.0200	Down
BHGZD *vs.* AIA-M_mRNA_Seq	NAGS	0.0408	-1.6725	Down
BHGZD *vs.* AIA-M_mRNA_Seq	NAPA	0.0011	-1.5382	Down
BHGZD *vs.* AIA-M_mRNA_Seq	NATD1	0.0030	-1.3747	Down
BHGZD *vs.* AIA-M_mRNA_Seq	NCK2	0.0000	-1.4045	Down
BHGZD *vs.* AIA-M_mRNA_Seq	NCKAP1	0.0000	-1.2797	Down
BHGZD *vs.* AIA-M_mRNA_Seq	NCOA4	0.0077	-1.0773	Down
BHGZD *vs.* AIA-M_mRNA_Seq	NDC80	0.0356	-1.0392	Down
BHGZD *vs.* AIA-M_mRNA_Seq	NDUFA1	0.0003	-1.5318	Down
BHGZD *vs.* AIA-M_mRNA_Seq	NDUFA1//NEWGENE_1560955//LOC108348144	0.0008	-1.4910	Down
BHGZD *vs.* AIA-M_mRNA_Seq	NDUFA11	0.0049	-1.2927	Down
BHGZD *vs.* AIA-M_mRNA_Seq	NDUFA12	0.0057	-1.0922	Down

BHGZD *vs.* AIA-M_mRNA_Seq	NDUFA13	0.0059	-1.1366	Down
BHGZD *vs.* AIA-M_mRNA_Seq	NDUFAF8	0.0105	-1.1756	Down
BHGZD *vs.* AIA-M_mRNA_Seq	NDUFB11	0.0095	-1.1127	Down
BHGZD *vs.* AIA-M_mRNA_Seq	NDUFB2	0.0170	-1.0173	Down
BHGZD *vs.* AIA-M_mRNA_Seq	NDUFC1	0.0170	-1.0310	Down
BHGZD *vs.* AIA-M_mRNA_Seq	NEDD8	0.0011	-1.3824	Down
BHGZD *vs.* AIA-M_mRNA_Seq	NEK2//NEK2L1	0.0257	-1.7163	Down
BHGZD *vs.* AIA-M_mRNA_Seq	NEURL1	0.0000	-1.4923	Down
BHGZD *vs.* AIA-M_mRNA_Seq	NFE2	0.0007	-1.2552	Down
BHGZD *vs.* AIA-M_mRNA_Seq	NFKBIB	0.0009	-1.3369	Down
BHGZD *vs.* AIA-M_mRNA_Seq	NINJ2	0.0021	-1.3729	Down
BHGZD *vs.* AIA-M_mRNA_Seq	NIPA1	0.0087	3.1578	Up
BHGZD *vs.* AIA-M_mRNA_Seq	NLK	0.0000	-1.1014	Down
BHGZD *vs.* AIA-M_mRNA_Seq	NMRK1	0.0092	-2.7614	Down
BHGZD *vs.* AIA-M_mRNA_Seq	NOL4L	0.0414	-1.9413	Down
BHGZD *vs.* AIA-M_mRNA_Seq	NOS1AP//LOC10036 1087	0.0077	-1.2200	Down
BHGZD *vs.* AIA-M_mRNA_Seq	NPTXR	0.0379	-3.6461	Down
BHGZD *vs.* AIA-M_mRNA_Seq	NPY	0.0003	-1.4144	Down
BHGZD *vs.* AIA-M_mRNA_Seq	NR4A3	0.0083	-1.9842	Down
BHGZD *vs.* AIA-M_mRNA_Seq	NRCAM	0.0089	-1.2036	Down
BHGZD *vs.* AIA-M_mRNA_Seq	NRG1	0.0093	-1.3917	Down
BHGZD *vs.* AIA-M_mRNA_Seq	NRGN	0.0001	-1.4248	Down
BHGZD *vs.* AIA-M_mRNA_Seq	NRIP3	0.0016	-1.0978	Down
BHGZD *vs.* AIA-M_mRNA_Seq	NRTN	0.0003	-1.6553	Down
BHGZD *vs.* AIA-M_mRNA_Seq	NT5C3A	0.0000	-1.4062	Down
BHGZD *vs.* AIA-M_mRNA_Seq	NUCB2	0.0001	-1.2429	Down

BHGZD *vs.* AIA-M_mRNA_Seq	NUDT2	0.0185	-1.0576	Down
BHGZD *vs.* AIA-M_mRNA_Seq	NUDT4	0.0015	-1.2969	Down
BHGZD *vs.* AIA-M_mRNA_Seq	NUP62CL	0.0308	-1.0537	Down
BHGZD *vs.* AIA-M_mRNA_Seq	NUPR2	0.0001	-1.8266	Down
BHGZD *vs.* AIA-M_mRNA_Seq	NXF2	0.0093	-5.4044	Down
BHGZD *vs.* AIA-M_mRNA_Seq	NXT1	0.0008	-1.6966	Down
BHGZD *vs.* AIA-M_mRNA_Seq	OAS1G	0.0024	2.4580	Up
BHGZD *vs.* AIA-M_mRNA_Seq	OAZ1	0.0007	-1.4725	Down
BHGZD *vs.* AIA-M_mRNA_Seq	ODC1	0.0000	-1.3426	Down
BHGZD *vs.* AIA-M_mRNA_Seq	OLFM4	0.0336	1.1084	Up
BHGZD *vs.* AIA-M_mRNA_Seq	OLR1637	0.0109	2.8840	Up
BHGZD *vs.* AIA-M_mRNA_Seq	OLR59	0.0047	-1.9336	Down
BHGZD *vs.* AIA-M_mRNA_Seq	OPN3	0.0009	-1.1988	Down
BHGZD *vs.* AIA-M_mRNA_Seq	OPTN	0.0045	-1.1834	Down
BHGZD *vs.* AIA-M_mRNA_Seq	ORC6	0.0344	1.1976	Up
BHGZD *vs.* AIA-M_mRNA_Seq	ORMDL3	0.0002	-1.1673	Down
BHGZD *vs.* AIA-M_mRNA_Seq	OSBPL1A	0.0001	-1.0807	Down
BHGZD *vs.* AIA-M_mRNA_Seq	OST4	0.0006	-1.4842	Down
BHGZD *vs.* AIA-M_mRNA_Seq	OTOA	0.0466	-5.1508	Down
BHGZD *vs.* AIA-M_mRNA_Seq	OTUB2	0.0095	-1.1483	Down
BHGZD *vs.* AIA-M_mRNA_Seq	P2RX1	0.0000	-1.4235	Down
BHGZD *vs.* AIA-M_mRNA_Seq	P2RY1	0.0000	-1.6463	Down
BHGZD *vs.* AIA-M_mRNA_Seq	P2RY12	0.0000	-1.5602	Down
BHGZD *vs.* AIA-M_mRNA_Seq	P3H3	0.0132	1.3148	Up
BHGZD *vs.* AIA-M_mRNA_Seq	PAIP2	0.0024	-1.2009	Down
BHGZD *vs.* AIA-M_mRNA_Seq	PAM	0.0001	-1.2818	Down
BHGZD *vs.* AIA-M_mRNA_Seq	PAQR6	0.0047	-1.4019	Down

BHGZD *vs.* AIA-M_mRNA_Seq	PAQR9	0.0014	-1.4533	Down
BHGZD *vs.* AIA-M_mRNA_Seq	PARVB	0.0001	-1.4990	Down
BHGZD *vs.* AIA-M_mRNA_Seq	PASK	0.0386	1.2195	Up
BHGZD *vs.* AIA-M_mRNA_Seq	PATZ1	0.0001	-1.2687	Down
BHGZD *vs.* AIA-M_mRNA_Seq	PCDHGB5	0.0274	3.5018	Up
BHGZD *vs.* AIA-M_mRNA_Seq	PCMT1	0.0002	-1.2344	Down
BHGZD *vs.* AIA-M_mRNA_Seq	PCNP	0.0008	-1.2251	Down
BHGZD *vs.* AIA-M_mRNA_Seq	PCSK1N//LOC103690091//LOC108348172	0.0302	-1.2303	Down
BHGZD *vs.* AIA-M_mRNA_Seq	PCSK1N//LOC108348172	0.0063	-1.4267	Down
BHGZD *vs.* AIA-M_mRNA_Seq	PCSK6	0.0002	-2.7458	Down
BHGZD *vs.* AIA-M_mRNA_Seq	PDE3A	0.0001	-1.4264	Down
BHGZD *vs.* AIA-M_mRNA_Seq	PDE5A	0.0000	-1.4466	Down
BHGZD *vs.* AIA-M_mRNA_Seq	PDLIM1	0.0000	-1.3929	Down
BHGZD *vs.* AIA-M_mRNA_Seq	PDZK1IP1	0.0140	-1.5762	Down
BHGZD *vs.* AIA-M_mRNA_Seq	PEAR1	0.0000	-1.3727	Down
BHGZD *vs.* AIA-M_mRNA_Seq	PEBP1	0.0008	-1.3536	Down
BHGZD *vs.* AIA-M_mRNA_Seq	PECR	0.0187	1.0183	Up
BHGZD *vs.* AIA-M_mRNA_Seq	PF4	0.0004	-1.4629	Down
BHGZD *vs.* AIA-M_mRNA_Seq	PGAP1	0.0003	1.8565	Up
BHGZD *vs.* AIA-M_mRNA_Seq	PGLYRP3	0.0032	-1.6226	Down
BHGZD *vs.* AIA-M_mRNA_Seq	PGLYRP3B	0.0331	-1.7295	Down
BHGZD *vs.* AIA-M_mRNA_Seq	PGPEP1	0.0429	1.0256	Up
BHGZD *vs.* AIA-M_mRNA_Seq	PHB	0.0016	-1.0872	Down
BHGZD *vs.* AIA-M_mRNA_Seq	PHETA2	0.0091	1.3172	Up
BHGZD *vs.* AIA-M_mRNA_Seq	PHOSPHO1	0.0030	-1.3974	Down

BHGZD *vs.* AIA-M_mRNA_Seq	PI4K2B	0.0006	-1.0575	Down
BHGZD *vs.* AIA-M_mRNA_Seq	PIANP	0.0471	-1.0732	Down
BHGZD *vs.* AIA-M_mRNA_Seq	PIGQ	0.0074	-1.0895	Down
BHGZD *vs.* AIA-M_mRNA_Seq	PIM1	0.0031	-1.1513	Down
BHGZD *vs.* AIA-M_mRNA_Seq	PINK1	0.0003	-1.4308	Down
BHGZD *vs.* AIA-M_mRNA_Seq	PIP5K1B	0.0036	-1.1939	Down
BHGZD *vs.* AIA-M_mRNA_Seq	PJA1	0.0000	-1.0142	Down
BHGZD *vs.* AIA-M_mRNA_Seq	PKD1L2	0.0013	-1.6635	Down
BHGZD *vs.* AIA-M_mRNA_Seq	PKIA	0.0002	-1.3519	Down
BHGZD *vs.* AIA-M_mRNA_Seq	PKLR	0.0007	-1.7943	Down
BHGZD *vs.* AIA-M_mRNA_Seq	PKN2	0.0037	-1.1477	Down
BHGZD *vs.* AIA-M_mRNA_Seq	PLA1A	0.0000	-1.4809	Down
BHGZD *vs.* AIA-M_mRNA_Seq	PLA2G2A	0.0001	-1.5044	Down
BHGZD *vs.* AIA-M_mRNA_Seq	PLAT	0.0005	3.0763	Up
BHGZD *vs.* AIA-M_mRNA_Seq	PLEKHB1	0.0029	-1.5719	Down
BHGZD *vs.* AIA-M_mRNA_Seq	PLEKHH3	0.0216	1.2192	Up
BHGZD *vs.* AIA-M_mRNA_Seq	PLEKHJ1	0.0011	-1.5404	Down
BHGZD *vs.* AIA-M_mRNA_Seq	PLEKHO1	0.0010	-1.5349	Down
BHGZD *vs.* AIA-M_mRNA_Seq	PLOD2	0.0000	-1.8337	Down
BHGZD *vs.* AIA-M_mRNA_Seq	PLP1	0.0000	-1.3573	Down
BHGZD *vs.* AIA-M_mRNA_Seq	PLS1	0.0000	-1.2310	Down
BHGZD *vs.* AIA-M_mRNA_Seq	PLTP	0.0012	-1.3760	Down
BHGZD *vs.* AIA-M_mRNA_Seq	PNKD	0.0023	-1.4333	Down
BHGZD *vs.* AIA-M_mRNA_Seq	PON1	0.0253	1.6109	Up
BHGZD *vs.* AIA-M_mRNA_Seq	PPBP	0.0000	-1.7361	Down
BHGZD *vs.* AIA-M_mRNA_Seq	PPFIA4	0.0002	-1.0912	Down
BHGZD *vs.* AIA-M_mRNA_Seq	PPIF	0.0001	-1.3334	Down

BHGZD *vs.* AIA-M_mRNA_Seq	PPM1E	0.0307	1.0831	Up
BHGZD *vs.* AIA-M_mRNA_Seq	PPP2R5B	0.0119	-1.0650	Down
BHGZD *vs.* AIA-M_mRNA_Seq	PRAF2	0.0011	-1.6593	Down
BHGZD *vs.* AIA-M_mRNA_Seq	PRDX2	0.0008	-1.5057	Down
BHGZD *vs.* AIA-M_mRNA_Seq	PRELID3A	0.0359	-2.6534	Down
BHGZD *vs.* AIA-M_mRNA_Seq	PRKAR2B	0.0000	-1.2695	Down
BHGZD *vs.* AIA-M_mRNA_Seq	PRKD1	0.0450	2.1290	Up
BHGZD *vs.* AIA-M_mRNA_Seq	PROCR	0.0004	-1.4674	Down
BHGZD *vs.* AIA-M_mRNA_Seq	PROSER2	0.0073	-1.4237	Down
BHGZD *vs.* AIA-M_mRNA_Seq	PRTFDC1	0.0001	-1.4336	Down
BHGZD *vs.* AIA-M_mRNA_Seq	PRXL2A	0.0060	-1.2514	Down
BHGZD *vs.* AIA-M_mRNA_Seq	PSENEN	0.0005	-1.5602	Down
BHGZD *vs.* AIA-M_mRNA_Seq	PSMD4	0.0024	-1.2502	Down
BHGZD *vs.* AIA-M_mRNA_Seq	PSMD9	0.0014	-1.3777	Down
BHGZD *vs.* AIA-M_mRNA_Seq	PSME1-PS1	0.0089	-1.4399	Down
BHGZD *vs.* AIA-M_mRNA_Seq	PSMF1	0.0118	-1.0468	Down
BHGZD *vs.* AIA-M_mRNA_Seq	PTDSS2	0.0064	-1.1168	Down
BHGZD *vs.* AIA-M_mRNA_Seq	PTGS1	0.0000	-1.5111	Down
BHGZD *vs.* AIA-M_mRNA_Seq	PTOV1	0.0033	-1.1137	Down
BHGZD *vs.* AIA-M_mRNA_Seq	PTPN13	0.0331	-2.6389	Down
BHGZD *vs.* AIA-M_mRNA_Seq	PTPRJ	0.0001	-1.1227	Down
BHGZD *vs.* AIA-M_mRNA_Seq	PTTG1	0.0024	-1.6067	Down
BHGZD *vs.* AIA-M_mRNA_Seq	PTTG1IP	0.0001	-1.0350	Down
BHGZD *vs.* AIA-M_mRNA_Seq	R3HDM4	0.0014	-1.2220	Down
BHGZD *vs.* AIA-M_mRNA_Seq	RAB11A	0.0016	-1.0218	Down
BHGZD *vs.* AIA-M_mRNA_Seq	RAB27B	0.0005	-1.2478	Down
BHGZD *vs.* AIA-M_mRNA_Seq	RAB3IL1	0.0061	-1.2725	Down

BHGZD *vs.* AIA-M_mRNA_Seq	RAB43	0.0032	-1.1207	Down
BHGZD *vs.* AIA-M_mRNA_Seq	RABEPK	0.0047	-1.1352	Down
BHGZD *vs.* AIA-M_mRNA_Seq	RAC1	0.0002	-1.0531	Down
BHGZD *vs.* AIA-M_mRNA_Seq	RACK1	0.0031	-1.1744	Down
BHGZD *vs.* AIA-M_mRNA_Seq	RAD23A	0.0003	-1.6208	Down
BHGZD *vs.* AIA-M_mRNA_Seq	RAD51AP1	0.0048	1.6318	Up
BHGZD *vs.* AIA-M_mRNA_Seq	RAD51C	0.0409	-1.0536	Down
BHGZD *vs.* AIA-M_mRNA_Seq	RAMP3	0.0000	-1.5240	Down
BHGZD *vs.* AIA-M_mRNA_Seq	RANBP10	0.0240	-1.0807	Down
BHGZD *vs.* AIA-M_mRNA_Seq	RAP1GAP	0.0027	4.0986	Up
BHGZD *vs.* AIA-M_mRNA_Seq	RASGRP3	0.0002	-1.2567	Down
BHGZD *vs.* AIA-M_mRNA_Seq	RASL10A	0.0001	-1.3945	Down
BHGZD *vs.* AIA-M_mRNA_Seq	RASSF7	0.0275	-1.2678	Down
BHGZD *vs.* AIA-M_mRNA_Seq	RBM38	0.0069	-1.3115	Down
BHGZD *vs.* AIA-M_mRNA_Seq	RBMS2	0.0005	-1.2979	Down
BHGZD *vs.* AIA-M_mRNA_Seq	RBPMS2	0.0067	-2.0492	Down
BHGZD *vs.* AIA-M_mRNA_Seq	REC114	0.0011	-1.5782	Down
BHGZD *vs.* AIA-M_mRNA_Seq	REEP2	0.0000	-1.4944	Down
BHGZD *vs.* AIA-M_mRNA_Seq	REPS2	0.0000	-6.6043	Down
BHGZD *vs.* AIA-M_mRNA_Seq	RET	0.0002	-1.1924	Down
BHGZD *vs.* AIA-M_mRNA_Seq	REXO2	0.0008	-1.3899	Down
BHGZD *vs.* AIA-M_mRNA_Seq	RFFL	0.0007	-1.3043	Down
BHGZD *vs.* AIA-M_mRNA_Seq	RGD1309350	0.0006	-1.7082	Down
BHGZD *vs.* AIA-M_mRNA_Seq	RGD1309779	0.0446	1.5398	Up
BHGZD *vs.* AIA-M_mRNA_Seq	RGD1310166	0.0490	-2.9606	Down
BHGZD *vs.* AIA-M_mRNA_Seq	RGD1310507	0.0033	-1.5821	Down
BHGZD *vs.* AIA-M_mRNA_Seq	RGD1311575	0.0001	-1.5037	Down

BHGZD *vs.* AIA-M_mRNA_Seq	RGD1311739	0.0016	-1.4109	Down
BHGZD *vs.* AIA-M_mRNA_Seq	RGD1359634	0.0207	2.9925	Up
BHGZD *vs.* AIA-M_mRNA_Seq	RGD1560015	0.0002	-1.1337	Down
BHGZD *vs.* AIA-M_mRNA_Seq	RGD1562029	0.0108	3.8040	Up
BHGZD *vs.* AIA-M_mRNA_Seq	RGD1562036	0.0089	-3.1653	Down
BHGZD *vs.* AIA-M_mRNA_Seq	RGD1562080	0.0017	-3.0379	Down
BHGZD *vs.* AIA-M_mRNA_Seq	RGD1562402	0.0024	-1.2785	Down
BHGZD *vs.* AIA-M_mRNA_Seq	RGD1562420	0.0208	-1.0369	Down
BHGZD *vs.* AIA-M_mRNA_Seq	RGD1562451	0.0213	-1.2808	Down
BHGZD *vs.* AIA-M_mRNA_Seq	RGD1563222	0.0300	-1.3581	Down
BHGZD *vs.* AIA-M_mRNA_Seq	RGD1563861	0.0255	-1.1131	Down
BHGZD *vs.* AIA-M_mRNA_Seq	RGD1564129	0.0007	-1.2745	Down
BHGZD *vs.* AIA-M_mRNA_Seq	RGD1564480	0.0006	-1.6024	Down
BHGZD *vs.* AIA-M_mRNA_Seq	RGD1565410	0.0011	5.1452	Up
BHGZD *vs.* AIA-M_mRNA_Seq	RGD1566099	0.0012	-1.6080	Down
BHGZD *vs.* AIA-M_mRNA_Seq	RGD1566369	0.0075	-1.0401	Down
BHGZD *vs.* AIA-M_mRNA_Seq	RGL1	0.0010	1.1014	Up
BHGZD *vs.* AIA-M_mRNA_Seq	RGS10	0.0003	-1.1043	Down
BHGZD *vs.* AIA-M_mRNA_Seq	RGS18	0.0000	-1.2328	Down
BHGZD *vs.* AIA-M_mRNA_Seq	RGS6	0.0002	-1.2242	Down
BHGZD *vs.* AIA-M_mRNA_Seq	RHAG	0.0077	-1.2140	Down
BHGZD *vs.* AIA-M_mRNA_Seq	RHBDL3	0.0001	-1.2811	Down
BHGZD *vs.* AIA-M_mRNA_Seq	RHD	0.0078	-1.3186	Down
BHGZD *vs.* AIA-M_mRNA_Seq	RIMKLA	0.0058	3.9068	Up
BHGZD *vs.* AIA-M_mRNA_Seq	RIOK3	0.0017	-1.3843	Down
BHGZD *vs.* AIA-M_mRNA_Seq	RIPOR3	0.0000	-7.2593	Down
BHGZD *vs.* AIA-M_mRNA_Seq	RN7SL1	0.0149	-1.2773	Down

BHGZD *vs.* AIA-M_mRNA_Seq	RNASEK	0.0008	-1.0501	Down
BHGZD *vs.* AIA-M_mRNA_Seq	RND1	0.0460	2.5463	Up
BHGZD *vs.* AIA-M_mRNA_Seq	RNF10	0.0015	-1.4918	Down
BHGZD *vs.* AIA-M_mRNA_Seq	RNF11L1	0.0001	-1.3206	Down
BHGZD *vs.* AIA-M_mRNA_Seq	RNF123	0.0034	-1.2518	Down
BHGZD *vs.* AIA-M_mRNA_Seq	RNF152	0.0459	-1.5736	Down
BHGZD *vs.* AIA-M_mRNA_Seq	RNF187	0.0012	-1.4005	Down
BHGZD *vs.* AIA-M_mRNA_Seq	RNF5	0.0006	-1.5312	Down
BHGZD *vs.* AIA-M_mRNA_Seq	RPAP1	0.0000	-1.4851	Down
BHGZD *vs.* AIA-M_mRNA_Seq	RPL10//RGD1563861	0.0010	-1.4014	Down
BHGZD *vs.* AIA-M_mRNA_Seq	RPL11	0.0010	-1.2118	Down
BHGZD *vs.* AIA-M_mRNA_Seq	RPL12	0.0023	-1.2417	Down
BHGZD *vs.* AIA-M_mRNA_Seq	RPL12-PS1	0.0027	-1.1343	Down
BHGZD *vs.* AIA-M_mRNA_Seq	RPL13	0.0011	-1.2271	Down
BHGZD *vs.* AIA-M_mRNA_Seq	RPL13A	0.0018	-1.2561	Down
BHGZD *vs.* AIA-M_mRNA_Seq	RPL15	0.0037	-1.1421	Down
BHGZD *vs.* AIA-M_mRNA_Seq	RPL17	0.0029	-1.0626	Down
BHGZD *vs.* AIA-M_mRNA_Seq	RPL18A	0.0007	-1.5001	Down
BHGZD *vs.* AIA-M_mRNA_Seq	RPL19	0.0033	-1.1390	Down
BHGZD *vs.* AIA-M_mRNA_Seq	RPL21	0.0003	-1.4967	Down
BHGZD *vs.* AIA-M_mRNA_Seq	RPL24	0.0008	-1.3211	Down
BHGZD *vs.* AIA-M_mRNA_Seq	RPL26	0.0007	-1.5254	Down
BHGZD *vs.* AIA-M_mRNA_Seq	RPL27A	0.0017	-1.2827	Down
BHGZD *vs.* AIA-M_mRNA_Seq	RPL28	0.0017	-1.3151	Down
BHGZD *vs.* AIA-M_mRNA_Seq	RPL29	0.0036	-1.0916	Down
BHGZD *vs.* AIA-M_mRNA_Seq	RPL30	0.0028	-1.4105	Down
BHGZD *vs.* AIA-M_mRNA_Seq	RPL31	0.0012	-1.2807	Down

BHGZD *vs.* AIA-M_mRNA_Seq	RPL31L4	0.0044	-1.0757	Down
BHGZD *vs.* AIA-M_mRNA_Seq	RPL32	0.0028	-1.0470	Down
BHGZD *vs.* AIA-M_mRNA_Seq	RPL35	0.0010	-1.4947	Down
BHGZD *vs.* AIA-M_mRNA_Seq	RPL35A//LOC103690996//LOC108349682	0.0023	-1.1413	Down
BHGZD *vs.* AIA-M_mRNA_Seq	RPL35A//LOC103690996//LOC108349682//LOC108351058	0.0027	-1.1384	Down
BHGZD *vs.* AIA-M_mRNA_Seq	RPL36	0.0028	-1.2333	Down
BHGZD *vs.* AIA-M_mRNA_Seq	RPL36AL//RPL36A-PS2//LOC108348287	0.0089	-1.0490	Down
BHGZD *vs.* AIA-M_mRNA_Seq	RPL37//LOC100360781//LOC100360841	0.0011	-1.6884	Down
BHGZD *vs.* AIA-M_mRNA_Seq	RPL37A-PS1//RPL37A	0.0010	-1.3658	Down
BHGZD *vs.* AIA-M_mRNA_Seq	RPL38-PS1	0.0001	-1.8542	Down
BHGZD *vs.* AIA-M_mRNA_Seq	RPL38-PS2	0.0038	-1.3034	Down
BHGZD *vs.* AIA-M_mRNA_Seq	RPL38-PS3	0.0001	-1.7464	Down
BHGZD *vs.* AIA-M_mRNA_Seq	RPL39//LOC103690821//LOC103692785//LOC103692831//LOC103693375//LOC103694169//LOC103694404	0.0159	-1.1673	Down
BHGZD *vs.* AIA-M_mRNA_Seq	RPL4	0.0023	-1.0453	Down
BHGZD *vs.* AIA-M_mRNA_Seq	RPL41	0.0010	-1.5483	Down
BHGZD *vs.* AIA-M_mRNA_Seq	RPL7	0.0016	-1.1948	Down
BHGZD *vs.* AIA-M_mRNA_Seq	RPLP0	0.0008	-1.4930	Down

BHGZD *vs.* AIA-M_mRNA_Seq	RPLP1//LOC100360522	0.0009	-1.5256	Down
BHGZD *vs.* AIA-M_mRNA_Seq	RPLP2//LOC100911575	0.0002	-1.6001	Down
BHGZD *vs.* AIA-M_mRNA_Seq	RPP25	0.0320	-1.5779	Down
BHGZD *vs.* AIA-M_mRNA_Seq	RPS10	0.0007	-1.4712	Down
BHGZD *vs.* AIA-M_mRNA_Seq	RPS10L1	0.0014	-1.4354	Down
BHGZD *vs.* AIA-M_mRNA_Seq	RPS12//LOC100360573	0.0005	-1.5406	Down
BHGZD *vs.* AIA-M_mRNA_Seq	RPS13//LOC684988	0.0031	-1.0147	Down
BHGZD *vs.* AIA-M_mRNA_Seq	RPS14//LOC100911847	0.0011	-1.4683	Down
BHGZD *vs.* AIA-M_mRNA_Seq	RPS15	0.0015	-1.3394	Down
BHGZD *vs.* AIA-M_mRNA_Seq	RPS15AL4	0.0100	-1.1298	Down
BHGZD *vs.* AIA-M_mRNA_Seq	RPS16	0.0035	-1.2782	Down
BHGZD *vs.* AIA-M_mRNA_Seq	RPS17	0.0019	-1.2422	Down
BHGZD *vs.* AIA-M_mRNA_Seq	RPS18	0.0042	-1.2695	Down
BHGZD *vs.* AIA-M_mRNA_Seq	RPS18L1	0.0005	-1.6961	Down
BHGZD *vs.* AIA-M_mRNA_Seq	RPS19	0.0030	-1.2698	Down
BHGZD *vs.* AIA-M_mRNA_Seq	RPS19//LOC100910336	0.0052	-1.0677	Down
BHGZD *vs.* AIA-M_mRNA_Seq	RPS2	0.0054	-1.0296	Down
BHGZD *vs.* AIA-M_mRNA_Seq	RPS20	0.0018	-1.3284	Down
BHGZD *vs.* AIA-M_mRNA_Seq	RPS23	0.0017	-1.2603	Down
BHGZD *vs.* AIA-M_mRNA_Seq	RPS24	0.0023	-1.2192	Down
BHGZD *vs.* AIA-M_mRNA_Seq	RPS27A	0.0000	-1.8142	Down

BHGZD *vs.* AIA-M_mRNA_Seq	RPS29//LOC108352650	0.0027	-1.4120	Down
BHGZD *vs.* AIA-M_mRNA_Seq	RPS3A	0.0015	-1.2565	Down
BHGZD *vs.* AIA-M_mRNA_Seq	RPS4X	0.0024	-1.1839	Down
BHGZD *vs.* AIA-M_mRNA_Seq	RPS4X-PS9//RPS4X	0.0040	-1.1573	Down
BHGZD *vs.* AIA-M_mRNA_Seq	RPS5	0.0032	-1.1458	Down
BHGZD *vs.* AIA-M_mRNA_Seq	RPS6KB2	0.0027	-1.2097	Down
BHGZD *vs.* AIA-M_mRNA_Seq	RPS7//LOC100362830	0.0040	-1.0188	Down
BHGZD *vs.* AIA-M_mRNA_Seq	RPS8	0.0026	-1.0664	Down
BHGZD *vs.* AIA-M_mRNA_Seq	RPSA	0.0017	-1.2365	Down
BHGZD *vs.* AIA-M_mRNA_Seq	RRM2B	0.0299	1.0285	Up
BHGZD *vs.* AIA-M_mRNA_Seq	RSAD2	0.0101	-1.1132	Down
BHGZD *vs.* AIA-M_mRNA_Seq	RSPH9	0.0169	-4.9822	Down
BHGZD *vs.* AIA-M_mRNA_Seq	RT1-HA	0.0230	1.1581	Up
BHGZD *vs.* AIA-M_mRNA_Seq	RT1-T24-1	0.0012	-1.1816	Down
BHGZD *vs.* AIA-M_mRNA_Seq	RTFDC1	0.0034	-1.0165	Down
BHGZD *vs.* AIA-M_mRNA_Seq	RTP4	0.0025	1.1822	Up
BHGZD *vs.* AIA-M_mRNA_Seq	RWDD2B	0.0063	1.1211	Up
BHGZD *vs.* AIA-M_mRNA_Seq	RXFP1	0.0033	-1.2965	Down
BHGZD *vs.* AIA-M_mRNA_Seq	SAG	0.0128	-4.6040	Down
BHGZD *vs.* AIA-M_mRNA_Seq	SAP30L	0.0001	-1.0851	Down
BHGZD *vs.* AIA-M_mRNA_Seq	SARS	0.0161	-1.0873	Down
BHGZD *vs.* AIA-M_mRNA_Seq	SAT2	0.0151	-1.4751	Down
BHGZD *vs.* AIA-M_mRNA_Seq	SCAI	0.0207	1.1195	Up
BHGZD *vs.* AIA-M_mRNA_Seq	SCAMP5	0.0000	-1.7017	Down
BHGZD *vs.* AIA-M_mRNA_Seq	SCARF1	0.0020	-1.5817	Down

BHGZD *vs.* AIA-M_mRNA_Seq	SCLY	0.0024	-1.4109	Down
BHGZD *vs.* AIA-M_mRNA_Seq	SDC1	0.0391	1.2074	Up
BHGZD *vs.* AIA-M_mRNA_Seq	SEC14L5	0.0013	-1.8066	Down
BHGZD *vs.* AIA-M_mRNA_Seq	SEC61G	0.0013	-1.6278	Down
BHGZD *vs.* AIA-M_mRNA_Seq	SELENBP1	0.0147	-1.1449	Down
BHGZD *vs.* AIA-M_mRNA_Seq	SELENOK	0.0014	-1.3013	Down
BHGZD *vs.* AIA-M_mRNA_Seq	SELENOM	0.0251	-1.5035	Down
BHGZD *vs.* AIA-M_mRNA_Seq	SEMA5A	0.0003	-4.0576	Down
BHGZD *vs.* AIA-M_mRNA_Seq	SEMA6D	0.0003	-1.2306	Down
BHGZD *vs.* AIA-M_mRNA_Seq	SEPT10	0.0271	1.5958	Up
BHGZD *vs.* AIA-M_mRNA_Seq	SEPT8	0.0416	-1.3064	Down
BHGZD *vs.* AIA-M_mRNA_Seq	SERF2	0.0006	-1.3055	Down
BHGZD *vs.* AIA-M_mRNA_Seq	SERP2	0.0007	-1.7643	Down
BHGZD *vs.* AIA-M_mRNA_Seq	SERPINB11	0.0000	-1.2382	Down
BHGZD *vs.* AIA-M_mRNA_Seq	SERPINB8	0.0260	1.1425	Up
BHGZD *vs.* AIA-M_mRNA_Seq	SERPINE2	0.0397	-1.4497	Down
BHGZD *vs.* AIA-M_mRNA_Seq	SERPINF1	0.0046	-2.6737	Down
BHGZD *vs.* AIA-M_mRNA_Seq	SFTPD	0.0021	-1.4149	Down
BHGZD *vs.* AIA-M_mRNA_Seq	SH2D1B2	0.0000	-1.5883	Down
BHGZD *vs.* AIA-M_mRNA_Seq	SH3BGR	0.0077	-1.3007	Down
BHGZD *vs.* AIA-M_mRNA_Seq	SH3GLB1	0.0006	-1.5723	Down
BHGZD *vs.* AIA-M_mRNA_Seq	SH3RF2	0.0442	-2.1533	Down
BHGZD *vs.* AIA-M_mRNA_Seq	SH3YL1	0.0030	-1.2224	Down
BHGZD *vs.* AIA-M_mRNA_Seq	SHPK	0.0000	-1.1246	Down
BHGZD *vs.* AIA-M_mRNA_Seq	SIK3	0.0066	-1.0021	Down
BHGZD *vs.* AIA-M_mRNA_Seq	SIN3B	0.0005	-1.2766	Down
BHGZD *vs.* AIA-M_mRNA_Seq	SIRT2	0.0004	-1.6874	Down

BHGZD *vs.* AIA-M_mRNA_Seq	SIX4	0.0380	3.7416	Up
BHGZD *vs.* AIA-M_mRNA_Seq	SKA1	0.0140	-1.9499	Down
BHGZD *vs.* AIA-M_mRNA_Seq	SKA3	0.0460	-1.0119	Down
BHGZD *vs.* AIA-M_mRNA_Seq	SLC18A2	0.0000	-1.3524	Down
BHGZD *vs.* AIA-M_mRNA_Seq	SLC22A23	0.0000	-2.0738	Down
BHGZD *vs.* AIA-M_mRNA_Seq	SLC24A3	0.0000	-1.6236	Down
BHGZD *vs.* AIA-M_mRNA_Seq	SLC25A18	0.0132	-4.0128	Down
BHGZD *vs.* AIA-M_mRNA_Seq	SLC25A37	0.0096	-1.2201	Down
BHGZD *vs.* AIA-M_mRNA_Seq	SLC25A38	0.0007	-1.4819	Down
BHGZD *vs.* AIA-M_mRNA_Seq	SLC25A39	0.0004	-1.7307	Down
BHGZD *vs.* AIA-M_mRNA_Seq	SLC2A4	0.0005	-1.4783	Down
BHGZD *vs.* AIA-M_mRNA_Seq	SLC30A10	0.0119	-1.3482	Down
BHGZD *vs.* AIA-M_mRNA_Seq	SLC35B1	0.0010	-1.3289	Down
BHGZD *vs.* AIA-M_mRNA_Seq	SLC38A5	0.0006	-1.5494	Down
BHGZD *vs.* AIA-M_mRNA_Seq	SLC3A2	0.0026	-1.4234	Down
BHGZD *vs.* AIA-M_mRNA_Seq	SLC40A1//LOC100911874	0.0000	-1.3934	Down
BHGZD *vs.* AIA-M_mRNA_Seq	SLC40A1//NEWGENE_620180	0.0000	-1.4706	Down
BHGZD *vs.* AIA-M_mRNA_Seq	SLC43A1	0.0173	-1.0722	Down
BHGZD *vs.* AIA-M_mRNA_Seq	SLC48A1	0.0016	-1.4264	Down
BHGZD *vs.* AIA-M_mRNA_Seq	SLC4A1	0.0311	-1.0723	Down
BHGZD *vs.* AIA-M_mRNA_Seq	SLC6A4	0.0000	-1.5201	Down
BHGZD *vs.* AIA-M_mRNA_Seq	SLC7A5	0.0000	-1.1010	Down
BHGZD *vs.* AIA-M_mRNA_Seq	SLIT1	0.0111	-1.0718	Down
BHGZD *vs.* AIA-M_mRNA_Seq	SMAD9//LOC103691556	0.0110	-2.6856	Down

BHGZD *vs.* AIA-M_mRNA_Seq	SMDT1	0.0027	-1.2508	Down
BHGZD *vs.* AIA-M_mRNA_Seq	SMIM1	0.0078	-1.5768	Down
BHGZD *vs.* AIA-M_mRNA_Seq	SMOX	0.0005	-1.5696	Down
BHGZD *vs.* AIA-M_mRNA_Seq	SMPD1	0.0001	-1.1724	Down
BHGZD *vs.* AIA-M_mRNA_Seq	SMPDL3B	0.0042	-1.0129	Down
BHGZD *vs.* AIA-M_mRNA_Seq	SMTN	0.0012	-1.3597	Down
BHGZD *vs.* AIA-M_mRNA_Seq	SNCA	0.0009	-1.5606	Down
BHGZD *vs.* AIA-M_mRNA_Seq	SNX3	0.0007	-1.4851	Down
BHGZD *vs.* AIA-M_mRNA_Seq	SNX33	0.0033	-1.0010	Down
BHGZD *vs.* AIA-M_mRNA_Seq	SOCS1	0.0073	-1.0574	Down
BHGZD *vs.* AIA-M_mRNA_Seq	SOWAHA	0.0024	-1.6062	Down
BHGZD *vs.* AIA-M_mRNA_Seq	SOX15	0.0243	-6.8370	Down
BHGZD *vs.* AIA-M_mRNA_Seq	SPECC1	0.0103	-1.2139	Down
BHGZD *vs.* AIA-M_mRNA_Seq	SPEF1	0.0071	-1.2025	Down
BHGZD *vs.* AIA-M_mRNA_Seq	SPRED2	0.0500	1.4641	Up
BHGZD *vs.* AIA-M_mRNA_Seq	SPRYD7	0.0083	-1.0096	Down
BHGZD *vs.* AIA-M_mRNA_Seq	SPTB	0.0129	-1.1295	Down
BHGZD *vs.* AIA-M_mRNA_Seq	SPTBN4	0.0014	-1.0941	Down
BHGZD *vs.* AIA-M_mRNA_Seq	SRC	0.0001	-1.2069	Down
BHGZD *vs.* AIA-M_mRNA_Seq	SRMS	0.0016	1.4440	Up
BHGZD *vs.* AIA-M_mRNA_Seq	SRPK3	0.0284	1.0772	Up
BHGZD *vs.* AIA-M_mRNA_Seq	SRXN1	0.0433	-1.4114	Down
BHGZD *vs.* AIA-M_mRNA_Seq	SSBP3	0.0006	-1.4987	Down
BHGZD *vs.* AIA-M_mRNA_Seq	SSMEM1	0.0031	-2.2691	Down
BHGZD *vs.* AIA-M_mRNA_Seq	SSR4	0.0011	-1.3273	Down
BHGZD *vs.* AIA-M_mRNA_Seq	ST3GAL2	0.0005	-1.4417	Down
BHGZD *vs.* AIA-M_mRNA_Seq	ST6GALNAC3	0.0177	-1.0250	Down

BHGZD *vs.* AIA-M_mRNA_Seq	STARD13	0.0142	1.1647	Up
BHGZD *vs.* AIA-M_mRNA_Seq	STARD6	0.0174	-1.2440	Down
BHGZD *vs.* AIA-M_mRNA_Seq	STIM1	0.0001	-1.1853	Down
BHGZD *vs.* AIA-M_mRNA_Seq	STOM	0.0000	-1.3455	Down
BHGZD *vs.* AIA-M_mRNA_Seq	STON2	0.0004	-1.6397	Down
BHGZD *vs.* AIA-M_mRNA_Seq	STRA8	0.0029	-1.9002	Down
BHGZD *vs.* AIA-M_mRNA_Seq	STRADB	0.0077	-1.1604	Down
BHGZD *vs.* AIA-M_mRNA_Seq	STX11	0.0000	-1.1858	Down
BHGZD *vs.* AIA-M_mRNA_Seq	STX5	0.0056	-1.0121	Down
BHGZD *vs.* AIA-M_mRNA_Seq	STXBP1	0.0002	-1.5291	Down
BHGZD *vs.* AIA-M_mRNA_Seq	SUDS3	0.0014	-1.2449	Down
BHGZD *vs.* AIA-M_mRNA_Seq	SUSD2	0.0419	1.0372	Up
BHGZD *vs.* AIA-M_mRNA_Seq	SV2A	0.0001	-1.0481	Down
BHGZD *vs.* AIA-M_mRNA_Seq	SVIP	0.0020	-1.5149	Down
BHGZD *vs.* AIA-M_mRNA_Seq	SYNGR1	0.0000	-2.2628	Down
BHGZD *vs.* AIA-M_mRNA_Seq	SYT5	0.0002	-1.3477	Down
BHGZD *vs.* AIA-M_mRNA_Seq	SYTL4	0.0001	-1.6984	Down
BHGZD *vs.* AIA-M_mRNA_Seq	TAL1	0.0000	-1.5858	Down
BHGZD *vs.* AIA-M_mRNA_Seq	TAX1BP1	0.0002	-1.2792	Down
BHGZD *vs.* AIA-M_mRNA_Seq	TAX1BP3	0.0005	-1.0984	Down
BHGZD *vs.* AIA-M_mRNA_Seq	TCF7L2	0.0225	1.1941	Up
BHGZD *vs.* AIA-M_mRNA_Seq	TCP11	0.0159	-1.1927	Down
BHGZD *vs.* AIA-M_mRNA_Seq	TCP11L1	0.0193	-1.1538	Down
BHGZD *vs.* AIA-M_mRNA_Seq	TENT5C	0.0020	-1.2484	Down
BHGZD *vs.* AIA-M_mRNA_Seq	TESC	0.0016	-1.1924	Down
BHGZD *vs.* AIA-M_mRNA_Seq	TEX2	0.0000	-1.0473	Down
BHGZD *vs.* AIA-M_mRNA_Seq	TFDP2	0.0026	-1.3466	Down

BHGZD *vs.* AIA-M_mRNA_Seq	TGFB1I1	0.0264	-1.0061	Down
BHGZD *vs.* AIA-M_mRNA_Seq	TGIF2	0.0005	-1.2433	Down
BHGZD *vs.* AIA-M_mRNA_Seq	THBS1	0.0000	-1.5613	Down
BHGZD *vs.* AIA-M_mRNA_Seq	TIMM17B//LOC100911130	0.0165	-1.4451	Down
BHGZD *vs.* AIA-M_mRNA_Seq	TIMM23//LOC100362432	0.0071	-1.0061	Down
BHGZD *vs.* AIA-M_mRNA_Seq	TIMP3	0.0004	-1.6675	Down
BHGZD *vs.* AIA-M_mRNA_Seq	TINAGL1	0.0006	-1.8323	Down
BHGZD *vs.* AIA-M_mRNA_Seq	TINF2	0.0048	-1.1032	Down
BHGZD *vs.* AIA-M_mRNA_Seq	TK1	0.0013	-1.6205	Down
BHGZD *vs.* AIA-M_mRNA_Seq	TLE5	0.0007	-1.4037	Down
BHGZD *vs.* AIA-M_mRNA_Seq	TLL2	0.0051	-4.4050	Down
BHGZD *vs.* AIA-M_mRNA_Seq	TMA7	0.0062	1.1763	Up
BHGZD *vs.* AIA-M_mRNA_Seq	TMBIM6	0.0006	-1.4265	Down
BHGZD *vs.* AIA-M_mRNA_Seq	TMCC2	0.0010	-1.4845	Down
BHGZD *vs.* AIA-M_mRNA_Seq	TMEFF2	0.0007	-2.5595	Down
BHGZD *vs.* AIA-M_mRNA_Seq	TMEM117	0.0000	-1.4119	Down
BHGZD *vs.* AIA-M_mRNA_Seq	TMEM134	0.0032	-1.1218	Down
BHGZD *vs.* AIA-M_mRNA_Seq	TMEM140	0.0001	-1.3274	Down
BHGZD *vs.* AIA-M_mRNA_Seq	TMEM144	0.0137	-1.1567	Down
BHGZD *vs.* AIA-M_mRNA_Seq	TMEM14C	0.0042	-1.1619	Down
BHGZD *vs.* AIA-M_mRNA_Seq	TMEM184A	0.0084	-1.5175	Down
BHGZD *vs.* AIA-M_mRNA_Seq	TMEM246	0.0016	-1.4807	Down
BHGZD *vs.* AIA-M_mRNA_Seq	TMEM256	0.0071	-1.3970	Down
BHGZD *vs.* AIA-M_mRNA_Seq	TMEM258B	0.0153	-1.7226	Down
BHGZD *vs.* AIA-M_mRNA_Seq	TMEM56	0.0015	-2.3856	Down

BHGZD *vs.* AIA-M_mRNA_Seq	TMEM86B	0.0016	-1.5648	Down
BHGZD *vs.* AIA-M_mRNA_Seq	TMEM9	0.0134	-1.0740	Down
BHGZD *vs.* AIA-M_mRNA_Seq	TMEM97	0.0182	1.0210	Up
BHGZD *vs.* AIA-M_mRNA_Seq	TMIE	0.0037	-1.3898	Down
BHGZD *vs.* AIA-M_mRNA_Seq	TMOD1	0.0053	-1.2447	Down
BHGZD *vs.* AIA-M_mRNA_Seq	TNFRSF11A	0.0003	2.3900	Up
BHGZD *vs.* AIA-M_mRNA_Seq	TP53TG5	0.0168	-3.3568	Down
BHGZD *vs.* AIA-M_mRNA_Seq	TPD52L1	0.0012	-1.2119	Down
BHGZD *vs.* AIA-M_mRNA_Seq	TPM1	0.0021	-1.4927	Down
BHGZD *vs.* AIA-M_mRNA_Seq	TPM4	0.0001	-1.1242	Down
BHGZD *vs.* AIA-M_mRNA_Seq	TPRG1L	0.0019	-1.2176	Down
BHGZD *vs.* AIA-M_mRNA_Seq	TPT1	0.0002	-1.6882	Down
BHGZD *vs.* AIA-M_mRNA_Seq	TPX2	0.0033	-1.3055	Down
BHGZD *vs.* AIA-M_mRNA_Seq	TREML1	0.0002	-1.4372	Down
BHGZD *vs.* AIA-M_mRNA_Seq	TRIAP1//LOC100910137//NEWGENE_1586233	0.0003	-1.1794	Down
BHGZD *vs.* AIA-M_mRNA_Seq	TRIAP1//NEWGENE_1586233	0.0014	-1.0307	Down
BHGZD *vs.* AIA-M_mRNA_Seq	TRIL	0.0289	-4.7701	Down
BHGZD *vs.* AIA-M_mRNA_Seq	TRIM10	0.0045	-1.5615	Down
BHGZD *vs.* AIA-M_mRNA_Seq	TRIM17	0.0370	-1.1120	Down
BHGZD *vs.* AIA-M_mRNA_Seq	TRIM58	0.0051	-1.3158	Down
BHGZD *vs.* AIA-M_mRNA_Seq	TRIR	0.0043	-1.0614	Down
BHGZD *vs.* AIA-M_mRNA_Seq	TRMT9B	0.0007	-1.6915	Down
BHGZD *vs.* AIA-M_mRNA_Seq	TRPC6	0.0001	-1.3142	Down
BHGZD *vs.* AIA-M_mRNA_Seq	TSC22D1	0.0000	-1.3799	Down

BHGZD *vs.* AIA-M_mRNA_Seq	TSPAN12	0.0000	-1.5640	Down
BHGZD *vs.* AIA-M_mRNA_Seq	TSPAN17	0.0003	-1.2472	Down
BHGZD *vs.* AIA-M_mRNA_Seq	TSPAN33	0.0014	-1.4014	Down
BHGZD *vs.* AIA-M_mRNA_Seq	TSPAN5	0.0179	-1.0485	Down
BHGZD *vs.* AIA-M_mRNA_Seq	TSPAN9	0.0004	-1.3575	Down
BHGZD *vs.* AIA-M_mRNA_Seq	TSPO2	0.0007	-1.6432	Down
BHGZD *vs.* AIA-M_mRNA_Seq	TSPYL5	0.0340	-1.5653	Down
BHGZD *vs.* AIA-M_mRNA_Seq	TTC27	0.0001	-1.1400	Down
BHGZD *vs.* AIA-M_mRNA_Seq	TTYH3	0.0001	-1.2176	Down
BHGZD *vs.* AIA-M_mRNA_Seq	TUBA4A	0.0010	-1.3379	Down
BHGZD *vs.* AIA-M_mRNA_Seq	TUBB1	0.0000	-1.7032	Down
BHGZD *vs.* AIA-M_mRNA_Seq	TUBB2A	0.0001	-1.3655	Down
BHGZD *vs.* AIA-M_mRNA_Seq	TUBB2B	0.0017	-1.0895	Down
BHGZD *vs.* AIA-M_mRNA_Seq	TUBB4B	0.0010	-1.4425	Down
BHGZD *vs.* AIA-M_mRNA_Seq	TUSC2	0.0043	-1.3212	Down
BHGZD *vs.* AIA-M_mRNA_Seq	TXN1	0.0077	-1.0425	Down
BHGZD *vs.* AIA-M_mRNA_Seq	TXN2	0.0047	-1.2682	Down
BHGZD *vs.* AIA-M_mRNA_Seq	TYMS	0.0221	-1.0200	Down
BHGZD *vs.* AIA-M_mRNA_Seq	TYRO3	0.0000	-1.7972	Down
BHGZD *vs.* AIA-M_mRNA_Seq	UBA52	0.0002	-1.7399	Down
BHGZD *vs.* AIA-M_mRNA_Seq	UBAC1	0.0024	-1.3939	Down
BHGZD *vs.* AIA-M_mRNA_Seq	UBALD1	0.0005	-1.1965	Down
BHGZD *vs.* AIA-M_mRNA_Seq	UBASH3B	0.0001	-1.0896	Down
BHGZD *vs.* AIA-M_mRNA_Seq	UBB	0.0007	-1.4931	Down
BHGZD *vs.* AIA-M_mRNA_Seq	UBE2B	0.0001	-1.5589	Down
BHGZD *vs.* AIA-M_mRNA_Seq	UBE2C	0.0026	-1.4693	Down
BHGZD *vs.* AIA-M_mRNA_Seq	UBE2E2	0.0016	-1.4357	Down

BHGZD *vs.* AIA-M_mRNA_Seq	UBE2J1	0.0037	-1.1665	Down
BHGZD *vs.* AIA-M_mRNA_Seq	UBE2M	0.0014	-1.3259	Down
BHGZD *vs.* AIA-M_mRNA_Seq	UBE2O	0.0176	-1.0614	Down
BHGZD *vs.* AIA-M_mRNA_Seq	UBE2R2	0.0018	-1.3996	Down
BHGZD *vs.* AIA-M_mRNA_Seq	UBE2S	0.0012	-1.2763	Down
BHGZD *vs.* AIA-M_mRNA_Seq	UBE2V1//LOC679539 //LOC100912618	0.0012	-1.4727	Down
BHGZD *vs.* AIA-M_mRNA_Seq	UBL4A	0.0005	-1.5293	Down
BHGZD *vs.* AIA-M_mRNA_Seq	UBL7	0.0059	-1.2809	Down
BHGZD *vs.* AIA-M_mRNA_Seq	UBXN11	0.0005	-1.6632	Down
BHGZD *vs.* AIA-M_mRNA_Seq	UBXN2A	0.0058	-1.2439	Down
BHGZD *vs.* AIA-M_mRNA_Seq	UBXN6	0.0026	-1.2152	Down
BHGZD *vs.* AIA-M_mRNA_Seq	UCP2	0.0033	-1.3519	Down
BHGZD *vs.* AIA-M_mRNA_Seq	UNC13B	0.0000	-1.4375	Down
BHGZD *vs.* AIA-M_mRNA_Seq	UQCR10	0.0288	-1.2418	Down
BHGZD *vs.* AIA-M_mRNA_Seq	UQCRH	0.0006	-1.5497	Down
BHGZD *vs.* AIA-M_mRNA_Seq	UROD	0.0176	-1.1135	Down
BHGZD *vs.* AIA-M_mRNA_Seq	UROS	0.0032	-1.6156	Down
BHGZD *vs.* AIA-M_mRNA_Seq	USE1	0.0011	-1.4692	Down
BHGZD *vs.* AIA-M_mRNA_Seq	USP15	0.0114	-1.1119	Down
BHGZD *vs.* AIA-M_mRNA_Seq	USP27X	0.0004	4.4683	Up
BHGZD *vs.* AIA-M_mRNA_Seq	USP29	0.0153	1.1940	Up
BHGZD *vs.* AIA-M_mRNA_Seq	USP46	0.0006	-1.2311	Down
BHGZD *vs.* AIA-M_mRNA_Seq	UTS2R	0.0187	-3.7157	Down
BHGZD *vs.* AIA-M_mRNA_Seq	VASH1	0.0000	-1.4343	Down
BHGZD *vs.* AIA-M_mRNA_Seq	VBP1	0.0005	-1.5982	Down
BHGZD *vs.* AIA-M_mRNA_Seq	VIPR2	0.0000	-1.4657	Down

BHGZD *vs.* AIA-M_mRNA_Seq	VNN3	0.0334	1.2322	Up
BHGZD *vs.* AIA-M_mRNA_Seq	VRK2	0.0090	-1.1618	Down
BHGZD *vs.* AIA-M_mRNA_Seq	VSIG2	0.0039	-1.2263	Down
BHGZD *vs.* AIA-M_mRNA_Seq	VWA3A	0.0059	-5.5050	Down
BHGZD *vs.* AIA-M_mRNA_Seq	VWF	0.0000	-1.4591	Down
BHGZD *vs.* AIA-M_mRNA_Seq	WBP1	0.0141	-1.0701	Down
BHGZD *vs.* AIA-M_mRNA_Seq	WBP2	0.0029	-1.2298	Down
BHGZD *vs.* AIA-M_mRNA_Seq	WDR34	0.0094	-1.2365	Down
BHGZD *vs.* AIA-M_mRNA_Seq	WDR45	0.0027	-1.3246	Down
BHGZD *vs.* AIA-M_mRNA_Seq	WDR62//NEWGENE_1306714	0.0265	1.6527	Up
BHGZD *vs.* AIA-M_mRNA_Seq	WNT11	0.0290	-2.5134	Down
BHGZD *vs.* AIA-M_mRNA_Seq	WNT2	0.0055	-2.3451	Down
BHGZD *vs.* AIA-M_mRNA_Seq	WWTR1	0.0010	-1.8317	Down
BHGZD *vs.* AIA-M_mRNA_Seq	XK	0.0000	-1.4285	Down
BHGZD *vs.* AIA-M_mRNA_Seq	XKR8	0.0002	-1.4189	Down
BHGZD *vs.* AIA-M_mRNA_Seq	YAP1	0.0002	-1.9990	Down
BHGZD *vs.* AIA-M_mRNA_Seq	YBX2	0.0003	-1.7178	Down
BHGZD *vs.* AIA-M_mRNA_Seq	YBX3	0.0010	-1.5023	Down
BHGZD *vs.* AIA-M_mRNA_Seq	YIPF3	0.0096	-1.0152	Down
BHGZD *vs.* AIA-M_mRNA_Seq	YPEL3	0.0016	-1.3542	Down
BHGZD *vs.* AIA-M_mRNA_Seq	YPEL5	0.0007	-1.3825	Down
BHGZD *vs.* AIA-M_mRNA_Seq	YWHAH	0.0001	-1.1209	Down
BHGZD *vs.* AIA-M_mRNA_Seq	ZBED5	0.0061	-1.1077	Down
BHGZD *vs.* AIA-M_mRNA_Seq	ZCCHC12	0.0024	-2.1065	Down
BHGZD *vs.* AIA-M_mRNA_Seq	ZDHHC2	0.0063	-1.0562	Down
BHGZD *vs.* AIA-M_mRNA_Seq	ZFAND2B	0.0004	-1.3023	Down

BHGZD *vs.* AIA-M_mRNA_Seq	ZFP780B-PS1	0.0131	1.3243	Up
BHGZD *vs.* AIA-M_mRNA_Seq	ZWINT	0.0002	-1.0175	Down

Table S6 The topological feature of nodes in the interaction network of RA-candidate-marker gene-BHGZD-effective gene

Node	Degree	Betweenness	Closeness
COX4I1	17.0000	0.0033	0.2943
COX6C	15.0000	0.0044	0.2659
TNFRSF14	2.0000	0.0002	0.2877
BTLA	3.0000	0.0000	0.2493
FOS	45.0000	0.0333	0.3907
JUND	7.0000	0.0002	0.3234
SERPINC1	30.0000	0.0064	0.3454
F2	48.0000	0.0340	0.3900
COX7C	14.0000	0.0003	0.2620
COX5B	17.0000	0.0049	0.3002
COX5A	20.0000	0.0100	0.3057
FGG	38.0000	0.0039	0.3673
FGA	40.0000	0.0066	0.3694
FGB	28.0000	0.0018	0.3578
ATP5A1	14.0000	0.0027	0.2969
ATP5B	13.0000	0.0027	0.2983
MT-CO2	23.0000	0.0162	0.3199
NCOA2	22.0000	0.0032	0.3338
AR	45.0000	0.0312	0.3936
ASS1	6.0000	0.0011	0.2783
ASL	6.0000	0.0001	0.2784
COX6B1	15.0000	0.0001	0.2636
JUN	55.0000	0.0347	0.4046

**NFKB1**	29.0000	0.0064	0.3618
**NFKBIA**	23.0000	0.0057	0.3574
IFIT3	4.0000	0.0075	0.2981
IFIT1	5.0000	0.0027	0.2695
CSNK2B	5.0000	0.0001	0.2783
CSNK2A1	12.0000	0.0048	0.3195
MT-CO3	12.0000	0.0000	0.2597
ITGB2	28.0000	0.0144	0.3472
ITGAL	17.0000	0.0102	0.3330
CDC20	24.0000	0.0052	0.2981
PLK1	20.0000	0.0028	0.3040
MT-CO1	14.0000	0.0008	0.2748
**PIK3R1**	52.0000	0.0170	0.3845
**PIK3CA**	49.0000	0.0130	0.3845
ATP5C1	12.0000	0.0061	0.2672
CDK1	36.0000	0.0384	0.3501
ATF3	13.0000	0.0006	0.3286
S100A9	3.0000	0.0002	0.2483
S100A8	6.0000	0.0083	0.3154
NCAPG	14.0000	0.0003	0.2680
AURKB	18.0000	0.0039	0.2873
ESPL1	14.0000	0.0034	0.2736
SERPIND1	22.0000	0.0006	0.3370
**AKT1**	77.0000	0.0734	0.4198
HSP90AA1	51.0000	0.0542	0.3921
NR3C1	35.0000	0.0184	0.3782
CFB	2.0000	0.0000	0.2710

C3	46.0000	0.0197	0.3562
YARS	6.0000	0.0014	0.2670
EPRS	16.0000	0.0258	0.3199
CACNB1	8.0000	0.0025	0.2566
CACNA2D1	8.0000	0.0002	0.2570
NCOA1	30.0000	0.0195	0.3612
PPARG	28.0000	0.0107	0.3624
HSPA1A	12.0000	0.0010	0.3296
CS	19.0000	0.0044	0.3056
MDH2	15.0000	0.0028	0.2834
ECT2	11.0000	0.0010	0.2761
F10	16.0000	0.0031	0.3077
OASL	4.0000	0.0040	0.2317
ESR1	42.0000	0.0169	0.3875
CACNB2	11.0000	0.0049	0.2658
CACNA1C	17.0000	0.0337	0.3194
COX6A2	14.0000	0.0003	0.2620
GRIN2B	16.0000	0.0063	0.3311
DLG4	26.0000	0.0300	0.3450
CACNG1	7.0000	0.0001	0.2569
CYP1A1	42.0000	0.0104	0.3247
AHR	22.0000	0.0085	0.3679
SKA1	14.0000	0.0015	0.2765
TOP2A	16.0000	0.0008	0.2734
PROC	24.0000	0.0008	0.3410
LSM6	2.0000	0.0000	0.2423
SF3B3	3.0000	0.0031	0.2423

CDC25B	8.0000	0.0001	0.2814
EIF3F	3.0000	0.0011	0.2765
RPS26	5.0000	0.0048	0.2568
CTTN	13.0000	0.0067	0.3340
SRC	83.0000	0.1233	0.4292
GRIN2A	14.0000	0.0062	0.3255
EGLN1	5.0000	0.0024	0.3143
AKR1C1	11.0000	0.0001	0.2771
AKR1C3	21.0000	0.0046	0.3141
HDAC2	16.0000	0.0104	0.3150
SMARCA5	7.0000	0.0010	0.2890
TPI1	22.0000	0.0267	0.3326
GAPDHS	4.0000	0.0007	0.3114
ACO2	11.0000	0.0010	0.2931
PIK3CD	15.0000	0.0005	0.3286
ESR2	20.0000	0.0037	0.3576
NR1H4	14.0000	0.0070	0.3123
NFKB2	9.0000	0.0001	0.3218
GATA1	7.0000	0.0037	0.3184
TAL1	1.0000	0.0000	0.2416
PPARA	22.0000	0.0069	0.3556
COX7B	12.0000	0.0000	0.2597
POMC	41.0000	0.0237	0.3704
AGRP	2.0000	0.0000	0.2922
HSD3B1	21.0000	0.0090	0.3126
CYP11A1	10.0000	0.0036	0.2865
UGT1A3	29.0000	0.0019	0.2899

UGT1A1	39.0000	0.0061	0.3085
GRIN1	20.0000	0.0346	0.3432
NRG2	7.0000	0.0000	0.3242
ERBB3	20.0000	0.0007	0.3523
VEGFA	75.0000	0.0477	0.4021
EGF	62.0000	0.0318	0.4001
IGF1	53.0000	0.0134	0.3905
ADH1B	14.0000	0.0005	0.2694
ALDH2	10.0000	0.0081	0.2918
RUVBL2	6.0000	0.0042	0.2918
PIH1D1	2.0000	0.0000	0.2827
**IL6**	90.0000	0.0783	0.4214
SOCS3	22.0000	0.0149	0.3495
**TNF**	62.0000	0.0352	0.4031
PTPN1	23.0000	0.0071	0.3527
CDC6	15.0000	0.0020	0.2992
HGF	36.0000	0.0028	0.3702
SLC51B	4.0000	0.0000	0.2406
SLC51A	4.0000	0.0000	0.2419
FKBP5	12.0000	0.0008	0.3235
AKR1C2	7.0000	0.0002	0.2622
GABRG2	17.0000	0.0080	0.2617
GABRA1	18.0000	0.0115	0.2605
CACNA1S	9.0000	0.0008	0.2663
CCL5	35.0000	0.0099	0.3521
PF4	39.0000	0.0031	0.3543
THBS1	24.0000	0.0084	0.3423

CENPU	14.0000	0.0005	0.2766
CENPT	9.0000	0.0003	0.2750
PARP1	11.0000	0.0050	0.3335
CASP3	31.0000	0.0252	0.3852
ACTB	26.0000	0.0146	0.3728
GABRB2	17.0000	0.0170	0.2692
**IL1B**	47.0000	0.0117	0.3829
QDPR	5.0000	0.0007	0.2633
SPR	9.0000	0.0031	0.3188
CEBPB	20.0000	0.0017	0.3421
KLHL13	8.0000	0.0001	0.2701
ALOX5	16.0000	0.0106	0.3202
LTC4S	1.0000	0.0000	0.2426
COX8A	12.0000	0.0007	0.2623
NLRP3	8.0000	0.0002	0.3191
ALB	74.0000	0.0715	0.4155
CLU	30.0000	0.0119	0.3566
APP	75.0000	0.0497	0.4100
RYR1	8.0000	0.0007	0.2595
F2RL3	14.0000	0.0023	0.3252
F11	12.0000	0.0000	0.2917
ITGB3	27.0000	0.0088	0.3562
ITGA5	12.0000	0.0024	0.3263
GABRA5	15.0000	0.0018	0.2235
CYP19A1	25.0000	0.0103	0.3461
DNMT1	9.0000	0.0009	0.3389
HSPA2	14.0000	0.0108	0.3120

MAOB	14.0000	0.0035	0.2789
COMT	15.0000	0.0021	0.2837
RARG	6.0000	0.0023	0.3090
VWF	42.0000	0.0155	0.3713
SERPINE2	8.0000	0.0001	0.2969
PGR	22.0000	0.0037	0.3590
SNCA	8.0000	0.0014	0.3335
HSPA1B	8.0000	0.0000	0.3054
NOS2	20.0000	0.0095	0.3628
HSD17B1	14.0000	0.0004	0.2953
GABRB3	13.0000	0.0000	0.2151
GABRA6	14.0000	0.0000	0.2170
CCL4	36.0000	0.0058	0.3726
CCL3	11.0000	0.0000	0.3207
ALDOC	13.0000	0.0015	0.3028
JAK1	23.0000	0.0052	0.3531
SERPINA1	35.0000	0.0139	0.3545
FCGRT	1.0000	0.0000	0.2937
NGFR	19.0000	0.0015	0.3387
ADH1A	14.0000	0.0005	0.2694
YAP1	10.0000	0.0014	0.3333
ABL1	20.0000	0.0056	0.3626
TNFSF13B	4.0000	0.0000	0.3170
CXCL2	24.0000	0.0010	0.3447
GAB1	18.0000	0.0006	0.3487
GABRA4	15.0000	0.0031	0.2171
PIK3CG	4.0000	0.0000	0.3057

IDH2	9.0000	0.0006	0.2895
FGF1	14.0000	0.0034	0.3175
FGF2	41.0000	0.0196	0.3751
PPBP	40.0000	0.0074	0.3634
GABRB1	15.0000	0.0031	0.2171
GRIN2C	8.0000	0.0000	0.2725
SLC25A4	5.0000	0.0001	0.2505
LPL	18.0000	0.0134	0.3405
EDN1	41.0000	0.0232	0.3843
PLAT	9.0000	0.0017	0.3253
IDH1	19.0000	0.0054	0.3293
HCK	24.0000	0.0058	0.3527
KHSRP	2.0000	0.0000	0.2962
SDC4	12.0000	0.0095	0.3352
GNAS	15.0000	0.0016	0.3410
ADCY2	28.0000	0.0054	0.3368
AHRR	3.0000	0.0002	0.2765
GABRA2	15.0000	0.0000	0.2171
GABRA3	14.0000	0.0000	0.2170
CYP2C19	28.0000	0.0021	0.3111
CYP2C9	31.0000	0.0021	0.3113
DHFR	11.0000	0.0038	0.3061
CSF1	27.0000	0.0018	0.3543
UNC13B	3.0000	0.0062	0.1957
PPFIA1	2.0000	0.0000	0.1638
CYP3A4	47.0000	0.0160	0.3299
GRIN2D	10.0000	0.0005	0.2968

APRT	8.0000	0.0023	0.2863
ATIC	8.0000	0.0050	0.3140
PTGS2	43.0000	0.0177	0.3815
VDR	10.0000	0.0025	0.3111
GP9	14.0000	0.0003	0.3071
CAT	35.0000	0.0356	0.3806
DAO	8.0000	0.0039	0.3046
PTGS1	13.0000	0.0021	0.3030
ADH1C	14.0000	0.0005	0.2694
CBR1	12.0000	0.0016	0.2835
FABP6	8.0000	0.0038	0.3046
HAO1	9.0000	0.0008	0.3128
GRM5	20.0000	0.0252	0.3380
YWHAE	13.0000	0.0043	0.3211
LARS2	3.0000	0.0000	0.2438
PLOD2	8.0000	0.0010	0.2425
PLOD1	8.0000	0.0010	0.2425
CEBPD	9.0000	0.0002	0.3095
GRIN3A	9.0000	0.0011	0.2748
HCAR3	17.0000	0.0000	0.3137
HCAR2	17.0000	0.0000	0.3137
RUNX2	18.0000	0.0050	0.3445
BGLAP	13.0000	0.0017	0.3373
RHOC	11.0000	0.0052	0.3273
ADCY5	28.0000	0.0054	0.3368
MTNR1B	19.0000	0.0026	0.3175
TTR	18.0000	0.0054	0.3419

CYP1B1	23.0000	0.0055	0.3266
RAB9A	2.0000	1.0000	1.0000
DENND2C	1.0000	0.0000	0.6667
UGT2B7	31.0000	0.0029	0.2991
FCGR2A	8.0000	0.0002	0.3347
CYP3A7	18.0000	0.0003	0.2810
CYP3A5	32.0000	0.0018	0.3020
GRIK1	2.0000	0.0000	0.2676
PPIF	7.0000	0.0022	0.3028
PPP1CC	23.0000	0.0165	0.3005
S100A13	2.0000	0.0001	0.2455
RET	9.0000	0.0032	0.3222
NR0B1	6.0000	0.0038	0.2984
CDK6	9.0000	0.0011	0.3195
LEPRE1	10.0000	0.0035	0.2443
COL1A1	19.0000	0.0280	0.3170
DBH	4.0000	0.0000	0.2408
NR3C2	13.0000	0.0024	0.3255
CRABP1	3.0000	0.0000	0.2444
CYP26A1	19.0000	0.0044	0.2860
PRDX5	3.0000	0.0001	0.2816
CD3G	16.0000	0.0073	0.3252
ITK	8.0000	0.0029	0.3200
GSTP1	22.0000	0.0070	0.3255
PRDX6	13.0000	0.0064	0.3095
GP1BB	10.0000	0.0001	0.2994
RHOB	12.0000	0.0086	0.3421

NR1I3	15.0000	0.0003	0.2947
CYP1A2	32.0000	0.0024	0.3044
GRB10	6.0000	0.0062	0.3184
F7	7.0000	0.0001	0.3023
PPP1R3B	4.0000	0.0019	0.2449
PTK2B	13.0000	0.0039	0.3456
CRABP2	3.0000	0.0004	0.2487
SLCO1B1	20.0000	0.0040	0.3132
SELP	18.0000	0.0072	0.3539
HSD11B1	13.0000	0.0108	0.3095
IL7	17.0000	0.0037	0.3313
SULT1E1	10.0000	0.0000	0.2770
NR1I2	25.0000	0.0086	0.3108
KCNA2	6.0000	0.0063	0.2728
NEDD4L	8.0000	0.0003	0.2754
UBA1	10.0000	0.0030	0.2874
F11R	6.0000	0.0001	0.3037
PYGL	6.0000	0.0027	0.2572
PYGM	6.0000	0.0021	0.2562
CD8A	6.0000	0.0002	0.3119
DUSP1	13.0000	0.0012	0.3271
ALOX15	15.0000	0.0013	0.3270
GSTM4	19.0000	0.0009	0.2985
GSTM5	17.0000	0.0001	0.2757
ITPA	11.0000	0.0024	0.2903
PCYT1B	3.0000	0.0002	0.2220
PCYT1A	4.0000	0.0027	0.2726

ELN	11.0000	0.0043	0.3284
LOXL3	1.0000	0.0000	0.2473
MYL9	6.0000	0.0045	0.2914
CES1	6.0000	0.0041	0.2525
ACTA1	10.0000	0.0018	0.3418
AKR1B1	14.0000	0.0081	0.3174
PEX10	7.0000	0.0039	0.3023
SFTPA1	1.0000	0.0000	0.2030
SFTPD	2.0000	0.0031	0.2546
MNDA	11.0000	0.0020	0.2918
LYZ	18.0000	0.0031	0.3189
OSM	12.0000	0.0002	0.3239
ABCB11	12.0000	0.0023	0.2504
PROS1	20.0000	0.0031	0.3382
PTPN13	4.0000	0.0031	0.3080
PLEKHA1	1.0000	0.0000	0.2356
TTC30B	2.0000	0.0031	0.2272
KIFAP3	1.0000	0.0000	0.1852
ANXA1	31.0000	0.0067	0.3574
TRIM72	1.0000	0.0000	0.2634
PRF1	9.0000	0.0038	0.3120
GNLY	2.0000	0.0000	0.2380
GSTM3	19.0000	0.0028	0.3167
GNPDA1	3.0000	0.0002	0.2670
HK1	12.0000	0.0090	0.3364
DOK4	1.0000	0.0000	0.2438
CEL	9.0000	0.0034	0.2737

GABRQ	13.0000	0.0000	0.2151
LCN2	10.0000	0.0005	0.3323
OLFM4	7.0000	0.0001	0.2953
UTS2R	12.0000	0.0005	0.3222
SPAG5	11.0000	0.0000	0.2650
CYP2A6	24.0000	0.0007	0.2788
PLOD3	8.0000	0.0010	0.2425
ARHGEF4	4.0000	0.0031	0.2560
IFNB1	16.0000	0.0025	0.3380
CYP2B6	36.0000	0.0065	0.3313
OPTN	6.0000	0.0007	0.2981
CYP2C8	30.0000	0.0019	0.3111
PLD1	16.0000	0.0077	0.3335
CYP2D6	15.0000	0.0006	0.2899
SULT2A1	16.0000	0.0019	0.3237
ENTPD1	11.0000	0.0041	0.2931
HRC	15.0000	0.0002	0.3313
C7	3.0000	0.0002	0.2751
P4HA1	7.0000	0.0004	0.2472
ARHGAP18	4.0000	0.0022	0.2568
ZFP36	7.0000	0.0003	0.3260
IRAK2	5.0000	0.0000	0.2878
STS	19.0000	0.0069	0.2874
SULT2B1	12.0000	0.0017	0.2742
AOC3	5.0000	0.0011	0.2704
ABCC1	2.0000	0.0000	0.3014
ABCG2	19.0000	0.0139	0.3323

GABRG3	12.0000	0.0000	0.2168
HSD11B2	4.0000	0.0001	0.2851
RHOV	6.0000	0.0014	0.3017
ESRRA	7.0000	0.0011	0.2998
TRAIP	7.0000	0.0000	0.2691
LRP8	8.0000	0.0008	0.3137
CYP26C1	14.0000	0.0003	0.2647
COX7A1	10.0000	0.0000	0.2583
GPER1	24.0000	0.0018	0.3482
DHFRL1	6.0000	0.0001	0.2663
CTSB	5.0000	0.0001	0.3132
MMRN1	16.0000	0.0000	0.3330
GNAZ	11.0000	0.0037	0.3398
WIPF3	1.0000	0.0000	0.2717
MTNR1A	18.0000	0.0015	0.3138
SLCO1A2	12.0000	0.0075	0.3098
EBF1	8.0000	0.0002	0.2883
PLD2	7.0000	0.0009	0.3126
CFTR	16.0000	0.0184	0.3385
GRIN3B	9.0000	0.0011	0.2748
FARS2	4.0000	0.0002	0.2464
CLEC5A	8.0000	0.0031	0.2719
PHYH	1.0000	0.0000	0.2322
NOC2L	4.0000	0.0035	0.2579
HGS	8.0000	0.0011	0.3191
EGLN2	2.0000	0.0000	0.2417
EGLN3	6.0000	0.0050	0.2962

CD177	7.0000	0.0000	0.2718
RNASE3	10.0000	0.0036	0.2812
PCGF3	2.0000	0.0000	0.2424
SCML2	3.0000	0.0003	0.2652
LGALS1	15.0000	0.0031	0.3298
PRSS3P2	14.0000	0.0065	0.3079
NCAN	3.0000	0.0011	0.2566
CYP27B1	4.0000	0.0002	0.3053
POLR2H	13.0000	0.0113	0.3192
COL4A3	12.0000	0.0037	0.2751
P2RY2	16.0000	0.0032	0.3394
BBOX1	2.0000	0.0022	0.2268
ARSI	2.0000	0.0000	0.2379
CEP83	5.0000	0.0000	0.2897
PHOSPHO1	3.0000	0.0004	0.2454
LDHA	12.0000	0.0045	0.3270
ANXA5	16.0000	0.0047	0.3525
PTPRB	8.0000	0.0015	0.3117
FSHR	11.0000	0.0009	0.3086
FGF4	10.0000	0.0000	0.3102
SHBG	8.0000	0.0001	0.3211
LCT	7.0000	0.0062	0.2674
GCK	8.0000	0.0015	0.2929
GPBAR1	7.0000	0.0006	0.2957
TAS1R2	17.0000	0.0000	0.3137
ARSA	10.0000	0.0025	0.2839
DUSP6	3.0000	0.0031	0.2877

LEPREL1	6.0000	0.0006	0.2494
HS3ST3A1	1.0000	0.0000	0.2512
PTPN22	3.0000	0.0031	0.2549
GAS2	5.0000	0.0124	0.2829
ID1	4.0000	0.0005	0.2925
IL22RA2	6.0000	0.0011	0.3003
MCHR2	26.0000	0.0014	0.3342
ESRRG	3.0000	0.0031	0.2674
C8G	2.0000	0.0000	0.2634
STAMBP	2.0000	0.0018	0.2872
SEMA6B	2.0000	0.0008	0.2376
PLXNC1	2.0000	0.0000	0.2084
CTSH	7.0000	0.0013	0.2849
S100A7	8.0000	0.0000	0.2772
CCKAR	13.0000	0.0032	0.3270
KLHL25	7.0000	0.0000	0.2691
EPHA7	4.0000	0.0000	0.3044
EPHA1	6.0000	0.0039	0.3107
NNMT	2.0000	0.0000	0.2441
BST1	10.0000	0.0067	0.2722
SPON2	3.0000	0.0002	0.2727
TPO	2.0000	0.0000	0.2490
TYR	7.0000	0.0071	0.3203
SLC6A4	6.0000	0.0017	0.2870
MEPE	15.0000	0.0001	0.3306
RAB26	1.0000	0.0000	0.6667
PAPSS1	7.0000	0.0031	0.3138

HIVEP3	5.0000	0.0031	0.2943
COL20A1	9.0000	0.0001	0.2433
STC2	14.0000	0.0000	0.3296
IL4I1	11.0000	0.0048	0.2846
LEPREL2	5.0000	0.0000	0.2423
CYP2U1	11.0000	0.0000	0.2913
TRIM17	3.0000	0.0032	0.1974
CEP70	6.0000	0.0000	0.2908
SEMA5A	3.0000	0.0023	0.2568
NR2F1	6.0000	0.0001	0.3069
SYT5	3.0000	0.0092	0.2429
KRT80	1.0000	0.0000	1.0000
KRT79	1.0000	0.0000	1.0000
ZNF521	6.0000	0.0002	0.2901
RNF217	7.0000	0.0000	0.2691
SPDYA	2.0000	0.0031	0.2596
UCK2	2.0000	0.0001	0.2351
GNMT	4.0000	0.0021	0.2535
ART4	2.0000	0.0000	0.2141
ABCC2	14.0000	0.0011	0.2727
SPTB	1.0000	0.0000	0.1888
SCN1A	6.0000	0.0062	0.2326
LIPG	6.0000	0.0010	0.2696
STON2	5.0000	0.0000	0.2947
GAS2L1	1.0000	0.0000	0.1809
RASL10A	2.0000	0.0031	0.2207
CD1D	3.0000	0.0001	0.3071

CTSL	4.0000	0.0003	0.3041
B4GALT1	2.0000	0.0004	0.2278
EGR3	1.0000	0.0000	0.1784
SLCO4C1	10.0000	0.0036	0.2759
TOP2B	4.0000	0.0002	0.2653
ABLIM2	1.0000	0.0000	0.3005
DIEXF	3.0000	0.0002	0.2120
RPP25	2.0000	0.0000	0.2059
TDRKH	1.0000	0.0000	0.2818
BHMT	3.0000	0.0004	0.2278
MIF	4.0000	0.0023	0.3096
PDE3A	5.0000	0.0001	0.2667
FOLR2	2.0000	0.0000	0.2141
MED9	7.0000	0.0000	0.2859
ACHE	5.0000	0.0036	0.3177
RGSL1	1.0000	0.0000	0.2537
HINT1	4.0000	0.0005	0.2527
PPAP2B	6.0000	0.0013	0.2749
CDCA2	8.0000	0.0000	0.2637
TLR5	8.0000	0.0001	0.3107
GADD45G	1.0000	0.0000	0.2594
FST	2.0000	0.0031	0.2298
CHRD	1.0000	0.0000	0.1869
NKG7	3.0000	0.0002	0.2626
PLA2G1B	17.0000	0.0057	0.3298
LSS	3.0000	0.0016	0.2443
HMGCR	3.0000	0.0002	0.2299

MAPK8IP1	4.0000	0.0001	0.3234
KCNMA1	8.0000	0.0040	0.2533
RTP4	3.0000	0.0000	0.2308
PRLR	5.0000	0.0000	0.3053
SLC2A1	10.0000	0.0084	0.3184
ABCB1	20.0000	0.0143	0.3456
MMRN2	3.0000	0.0031	0.2239
CLEC14A	3.0000	0.0047	0.2779
CTDSPL	3.0000	0.0031	0.2401
CTDSP1	1.0000	0.0000	0.1937
PRSS1	5.0000	0.0007	0.2860
APC2	1.0000	0.0000	0.2039
SLC24A5	2.0000	0.0001	0.2480
GABRD	13.0000	0.0023	0.2299
SLC22A7	8.0000	0.0008	0.2595
SLC22A8	12.0000	0.0049	0.2738
ATP1A2	3.0000	0.0010	0.1973
GSTA1	18.0000	0.0002	0.2813
PLTP	4.0000	0.0004	0.2989
PLA2G2A	14.0000	0.0011	0.3096
P2RX4	2.0000	0.0000	0.2721
CPB1	5.0000	0.0014	0.2657
SOAT1	4.0000	0.0000	0.2330
GABRG1	15.0000	0.0061	0.2606
BATF	3.0000	0.0000	0.2961
KCNA3	4.0000	0.0004	0.2713
PRKAB1	2.0000	0.0005	0.2544

ESRRB	1.0000	0.0000	0.2654
SERPINA6	4.0000	0.0000	0.3071
MB	3.0000	0.0000	0.3047
ATP1A1	4.0000	0.0040	0.2190
SOAT2	4.0000	0.0000	0.2330
CTRB1	4.0000	0.0009	0.2267
ATP1A3	2.0000	0.0000	0.1831
SLCO1B3	9.0000	0.0005	0.2810
GABRE	9.0000	0.0000	0.2153
SIRT5	5.0000	0.0004	0.3099
VKORC1L1	2.0000	0.0006	0.2435
AMY1A	5.0000	0.0004	0.2280
RMDN3	1.0000	0.0000	0.2609
ARC	4.0000	0.0000	0.2930
KCNK5	2.0000	0.0003	0.2482
AMY2B	6.0000	0.0010	0.2208
TYRO3	1.0000	0.0000	0.2528
AMY2A	6.0000	0.0024	0.2476
BRMS1L	2.0000	0.0000	0.2473
QRSL1	2.0000	0.0000	0.2433
ACSBG1	3.0000	0.0000	0.2591
MUC1	13.0000	0.0095	0.3410
LGALS3	5.0000	0.0001	0.3080
RBPJL	1.0000	0.0000	0.2397
ABO	2.0000	0.0002	0.2743
KCNA5	4.0000	0.0021	0.2823
KCNA4	4.0000	0.0025	0.2634

SCD	4.0000	0.0022	0.2859
GAMT	3.0000	0.0003	0.2179
GLRA3	4.0000	0.0064	0.2619
PLA2G2E	13.0000	0.0007	0.3013
UBD	2.0000	0.0001	0.2351
PDE5A	7.0000	0.0064	0.2937
LYVE1	3.0000	0.0000	0.2977
ART1	1.0000	0.0000	0.2502
SIGMAR1	2.0000	0.0004	0.2205
KCNA1	8.0000	0.0113	0.2851
MBL2	4.0000	0.0003	0.3026
NR4A2	2.0000	0.0031	0.2813
KCNA10	1.0000	0.0000	0.2220
C1S	2.0000	0.0000	0.2629
MAP2	8.0000	0.0049	0.3325
CUBN	1.0000	0.0000	0.2937
FECH	3.0000	0.0002	0.2862
VSNL1	3.0000	0.0062	0.2114
KCND1	1.0000	0.0000	0.1746
RAD54L2	1.0000	0.0000	0.2825
ASTN1	2.0000	0.0031	0.1916
MAP6	1.0000	0.0000	0.1608
ENSG00000258947	1.0000	0.0000	0.2496
AKAP12	3.0000	0.0025	0.3131
KCND2	4.0000	0.0094	0.2675
HAVCR2	3.0000	0.0003	0.2882
BCHE	5.0000	0.0009	0.3239

WNT2	3.0000	0.0000	0.3066
NQO1	11.0000	0.0028	0.3170
KCND3	3.0000	0.0000	0.2424
SLC6A9	2.0000	0.0031	0.2077
SLC12A2	1.0000	0.0000	0.2530
NUDT9	3.0000	0.0000	0.2129
OGFOD1	3.0000	0.0033	0.2456
OGFOD2	3.0000	0.0031	0.2050
SCARF1	1.0000	0.0000	0.2432
SLC22A11	3.0000	0.0006	0.2558
GPNMB	1.0000	0.0000	0.2607
HLX	1.0000	0.0000	0.2111
LYPLAL1	3.0000	0.0002	0.2330
MAN2C1	1.0000	0.0000	1.0000
MAN1B1	1.0000	0.0000	1.0000
TMLHE	1.0000	0.0000	0.1649
ANG	4.0000	0.0068	0.2980
NCEH1	1.0000	0.0000	0.2017
NMT2	1.0000	0.0000	0.2782
RNASE1	4.0000	0.0005	0.3028
PCLO	2.0000	0.0000	0.1638
GABRP	10.0000	0.0000	0.2147
ORAI1	1.0000	0.0000	0.2422
KCNB1	1.0000	0.0000	0.3005
PADI4	1.0000	0.0000	0.2032
SLCO2B1	7.0000	0.0004	0.2685
UGT3A1	1.0000	0.0000	0.2366

RPAP1	1.0000	0.0000	0.2260
SLC15A1	4.0000	0.0000	0.2386
SIGLEC1	1.0000	0.0000	0.2544
CMA1	1.0000	0.0000	0.2777
SLC26A2	1.0000	0.0000	0.2390
RNF150	1.0000	0.0000	0.1784
TREML1	2.0000	0.0031	0.2617
MSRA	2.0000	0.0019	0.2770
PTGR1	1.0000	0.0000	0.2364
SLC22A6	6.0000	0.0001	0.2565
KCNC1	1.0000	0.0000	0.1746
CELA1	1.0000	0.0000	0.2618
SLC5A2	2.0000	0.0000	0.2417
SLC16A7	2.0000	0.0000	0.2417
PLAGL1	2.0000	0.0000	0.2417
SLC16A1	2.0000	0.0000	0.2575
EPB41L3	1.0000	0.0000	0.2144
PMP22	2.0000	0.0031	0.2207
PRX	1.0000	0.0000	0.1809
FAM25A	1.0000	0.0000	0.1830
NPPB	1.0000	0.0000	0.2777
MTTP	2.0000	0.0000	0.2617
EFTUD1	1.0000	0.0000	0.1951
SLCO1C1	2.0000	0.0000	0.2190
SLC23A1	1.0000	0.0000	0.2111
NQO2	2.0000	0.0000	0.2412
RFX2	1.0000	0.0000	0.2149

TRIM13	2.0000	0.0002	0.2824
TREML4	1.0000	0.0000	0.2075
PCDH15	2.0000	0.0003	0.2824
ATP2B2	2.0000	0.0001	0.2586
RERG	1.0000	0.0000	0.2235
MEST	2.0000	0.0000	0.2417
PI4K2B	1.0000	0.0000	0.2778
KCNB2	1.0000	0.0000	0.2100
CBLN2	1.0000	0.0000	0.2196
HSPB6	2.0000	0.0031	0.2821
SETDB2	3.0000	0.0000	0.1954
SLCO4A1	3.0000	0.0000	0.2191
SLCO6A1	1.0000	0.0000	0.1721
ECE2	1.0000	0.0000	0.2777
EHD2	1.0000	0.0000	0.2062
CAMK1	1.0000	0.0000	0.2022
CA2	3.0000	0.0031	0.2682
PKIA	2.0000	0.0062	0.2368
ACKR4	1.0000	0.0000	0.2605
NANP	1.0000	0.0000	0.2201
PIM1	2.0000	0.0000	0.2777
ADAD1	1.0000	0.0000	0.2481
SCHIP1	1.0000	0.0000	1.0000
NRGN	1.0000	0.0000	1.0000
CHL1	1.0000	0.0000	0.2488
METRN	1.0000	0.0000	0.2286
CLC	1.0000	0.0000	0.2196

AQP3	1.0000	0.0000	0.2874
PAM	1.0000	0.0000	0.2704
CA4	1.0000	0.0000	0.2869
MAST2	1.0000	0.0000	0.2275
RORA	1.0000	0.0000	0.2654
OBSL1	1.0000	0.0000	0.2257
TNMD	1.0000	0.0000	0.2408
CD300LB	1.0000	0.0000	0.2138
LHFP	1.0000	0.0000	0.2101
SYTL4	1.0000	0.0000	0.2465
CD101	1.0000	0.0000	0.2455
MRVI1	1.0000	0.0000	0.2115
ANGPTL2	1.0000	0.0000	0.2966
RTCB	1.0000	0.0000	0.2424
P4HTM	1.0000	0.0000	0.1702

Table S7 The chemical profiling of Baihu-Guizhi Decoction (BHGZD) using UFLC-Q-TOF-MS/MS system

Bioactive compounds (BACs)	Cas No.	Molecular formula	Molecular Weight/Da	t_R (min)	Negative ion mode	Error (ppm)	Positive ion mode	Error (ppm)	Herbs
citric acid	77-92-9	$C_6H_8O_7$	192.03	1.5	191.02	4.2	—	—	*Glycyrrhizae Radix et Rhizoma*
*neomangiferin	64809-67-2	$C_{25}H_{28}O_{16}$	584.14	4.97	583.13	-0.2	585.15	0.7	*Anemarrhenae Rhizoma*
*mangiferin	4773-96-0	$C_{19}H_{18}O_{11}$	422.08	7.13	421.08	0.3	423.09	0.9	*Anemarrhenae Rhizoma*
schaftoside	51938-32-0	$C_{26}H_{28}O_{14}$	564.15	9.79	—	—	565.16	1.7	*Glycyrrhizae Radix et Rhizoma*
*isoschaftoside	52012-29-0	$C_{26}H_{28}O_{14}$	564.15	10.47	563.14	1	565.16	1.7	*Glycyrrhizae Radix et Rhizoma*
*7-O-methyl mangiferin	31002-12-7	$C_{20}H_{20}O_{11}$	436.1	10.7	435.09	0.9	437.11	0.9	*Anemarrhenae Rhizoma*
liquiritin	551-15-5	$C_{21}H_{22}O_9$	418.13	11.16	417.12	1	—	—	*Glycyrrhizae Radix et Rhizoma*
*vitexin	3681-93-4	$C_{21}H_{20}O_{10}$	432.11	11.31	431.1	-0.3	433.11	0.5	*Anemarrhenae Rhizoma*
*isovitexin	29702-25-8	$C_{21}H_{20}O_{10}$	432.11	11.56	431.1	0.6	433.11	0.5	*Anemarrhenae Rhizoma*
liquiritin apioside	199796-12-8	$C_{26}H_{30}O_{13}$	550.17	11.58	549.16	0.1	551.18	1.8	*Glycyrrhizae Radix et Rhizoma*
hydroxygenkwanin	20243-59-8	$C_{16}H_{12}O_6$	300.06	13.98	299.06	0.7	—	—	NA
*isoliquiritin apioside	120926-46-7	$C_{26}H_{30}O_{13}$	550.17	16.74	549.16	0.1	551.18	1.8	*Glycyrrhizae Radix et Rhizoma*
Glycyroside	125310-04-5	$C_{27}H_{30}O_{13}$	562.17	16.82	—	—	563.18	1.5	*Glycyrrhizae Radix et Rhizoma*

*isoliquiritin	5041-81-6	$C_{21}H_{22}O_9$	418.13	16.96	417.12	1	419.13	-0.1	*Glycyrrhizae Radix et Rhizoma*
ononin	486-62-4	$C_{22}H_{22}O_9$	430.13	17.08	—	—	431.13	0.4	*Glycyrrhizae Radix et Rhizoma*
officinalisinin Ⅰ	57944-18-0	$C_{45}H_{76}O_{19}$	920.5	17.26	—	—	921.5	-7.3	*Anemarrhenae Rhizoma*
*liquiritigenin	578-86-9	$C_{15}H_{12}O_4$	256.07	17.29	255.07	0.8	—	—	*Glycyrrhizae Radix et Rhizoma*
anemarsaponin E	136565-73-6	$C_{45}H_{76}O_{20}$	936.49	17.32	935.49	-0.1	—	—	*Anemarrhenae Rhizoma*
neoisoliquiritin	7014-39-3/59122-93-9	$C_{21}H_{22}O_9$	418.13	17.81	417.12	1	419.13	-0.1	*Glycyrrhizae Radix et Rhizoma*
cinnamic acid	621-82-9/140-10-3	$C_9H_8O_2$	148.05	18.6	—	—	149.06	-2.6	*Cinnamomi Ramulus*
xilingsaponin B	256642-89-4	$C_{45}H_{74}O_{18}$	902.49	20.76	—	—	903.5	2.5	*Anemarrhenae Rhizoma*
*timosaponin BII	136656-07-0	$C_{45}H_{76}O_{19}$	920.5	20.77	919.49	1	—	—	*Anemarrhenae Rhizoma*
timosaponin D	220095-97-6	$C_{45}H_{74}O_{19}$	918.48	23.04	917.48	1.7	919.49	3.4	*Anemarrhenae Rhizoma*
isoliquiritigenin	961-29-5	$C_{15}H_{12}O_4$	256.07	25.94	255.07	0.8	—	—	*Glycyrrhizae Radix et Rhizoma*
*formononetin	485-72-3	$C_{16}H_{12}O_4$	268.07	26.55	267.07	3.4	269.08	0	*Glycyrrhizae Radix et Rhizoma*
pseudoprototimosaponin AⅢ	142759-74-8	$C_{45}H_{74}O_{18}$	902.49	27.18	901.48	1.1	903.5	2.5	*Anemarrhenae Rhizoma*

glabrolide	10401-33-9	$C_{30}H_{44}O_4$	468.32	29.56	—	—	469.33	0.7	*Glycyrrhizae Radix et Rhizoma*
*anemarrhensaponin Ⅰ	163047-21-0	$C_{39}H_{66}O_{14}$	758.45	31.61	757.44	0.6	—	—	*Anemarrhenae Rhizoma*
glycyrrhetic acid 3-O-glucuronide	34096-83-8	$C_{36}H_{54}O_{10}$	646.37	31.74	—	—	647.38	1.1	*Glycyrrhizae Radix et Rhizoma*
*glycyrrhizic acid	1405-86-3	$C_{42}H_{62}O_{16}$	822.4	31.78	821.4	0.8	823.41	3.3	*Glycyrrhizae Radix et Rhizoma*
timosaponin AⅡ	117210-12-5	$C_{39}H_{64}O_{14}$	756.43	36.6	755.42	1.3	757.44	2	*Anemarrhenae Rhizoma*
timosaponin AⅣ	942621-71-8	$C_{39}H_{64}O_{13}$	740.43	40.08	739.43	1	—	—	*Anemarrhenae Rhizoma*

*Quantitative bioactive compounds (BACs)

Table S8 13 chemical compounds were quantitatively determined using UFLC-Q-TOF-MS/MS system

Bioactive compounds (ng/mL)	Time							
	30min	1h	2h	4h	6h	8h	12h	24h
timosaponin BⅡ	170964.00	652.22	62955.68	1051.32	29345.93	1214.54	—	—
mangiferin	149928.80	3113.73	249631.85	14345.01	74135.91	18821.78	8535.02	—
glycyrrhizic acid	43346.92	331.02	12301.35	2002.37	3882.31	3036.35	2253.26	145.49
neomangiferin	17115.79	—	—	—	—	54.54	36.61	—
7-O-methyl mangiferin	7683.26	527.96	22650.62	2471.67	29457.59	3495.38	1854.79	—
anemarrhensaponin Ⅰ	6903.70	—	2311.89	—	790.29	—	—	—
vitexin	1265.33	—	—	—	—	—	—	—
formononetin	523.60	—	—	—	—	—	—	—
liquiritigenin	188.15	10.05	97.25	24.13	60.18	56.51	—	—
isoliquiritin	177.85	—	—	—	160.76	—	—	—
isoliquiritin apioside	166.88	—	205.44	—	174.09	—	—	—
isoschaftoside	123.12	—	183.21	—	172.01	19.86	—	—

isovitexin	74.17	—	—	—	—	—	—	—

Table S9 The absorption distribution metabolism excretion (ADME) information on 19 bioactive compounds (BACs) included in Baihu-Guizhi Decoction (BHGZD)

Bioactive compounds	CAS No.	ADMET Absorption Level	ADMET BBB Level	ADMET BBB	ADMET Solubility	ADMET Solubility Level	ADMET Hepatotoxicit	ADMET Hepatotoxicity Probability	ADMET CYP2D6	ADMET CYP2D6 Probability	ADMET PPB Level	Druglikeness Weight	Druglikeness Grading	Source	Herbs
formononetin	485-72-3	0	2	-0.234	-3.564	3	1	0.986	1	0.693	1	0.909	Good	ETCM	*Glycyrrhizae Radix et Rhizoma*
liquiritigenin	578-86-9	0	2	-0.419	-3.234	3	1	0.986	0	0.257	2	0.823	Good	ETCM	*Glycyrrhizae Radix et Rhizoma*
hydroxygenkwanin	20243-59-8	0	3	-0.959	-3.211	3	1	0.966	1	0.594	2	0.737	Good	ETCM	NA
cinnamic acid	621-82-9/140-10-3	0	2	-0.161	-1.867	4	0	0.178	0	0.029	2	0.65	Moderate	ETCM	*Cinnamomi Ramulus*
isoliquiritigenin	961-29-5	0	2	-0.496	-2.736	3	1	0.927	0	0.376	2	0.582	Moderate	ETCM	*Glycyrrhizae Radix et Rhizoma*
ononin	486-62-4	1	4		-2.291	3	TRUE	NA	FALSE	NA	FALSE	0.54	NA	Prediction	*Glycyrrhizae Radix et Rhizoma*
glabrolide	10401-33-9	0	1	0.416	-7.427	1	FALSE	NA	FALSE	NA	TRUE	0.5	Moderate	Prediction	*Glycyrrhizae Radix et Rhizoma*
liquiritin	551-15-5	2	4	Undefined	-2.432	3	1	0.854	1	0.623	2	0.484	Weak	ETCM	*Glycyrrhizae Radix et Rhizoma*
citric acid	77-92-9	2	4	Undefined	0.981	5	0	0.006	0	0.059	0	0.397	Weak	ETCM	*Glycyrrhizae Radix et Rhizoma*
vitexin	3681-93-4	3	4	Undefined	-2.746	3	1	0.933	1	0.653	2	0.35	Weak	ETCM	*Anemarrhenae Rhizoma*

isovitexin	29702-25-8	3	4	Undefined	-2.659	3	1	0.933	1	0.574	2	0.35	Weak	ETCM	*Anemarrhenae Rhizoma*
7-O-methyl mangiferin	31002-12-7	3	4		-3.169	3	TRUE	NA	FALSE	NA	FALSE	0.339	NA	Prediction	*Anemarrhenae Rhizoma*
isoliquiritin	5041-81-6	3	4	Undefined	-2.288	3	1	0.854	0	0.277	0	0.298	Weak	ETCM	*Glycyrrhizae Radix et Rhizoma*
neoisoliquiritin	59122-93-9	3	4	Undefined	-2.311	3	1	0.841	0	0.237	0	0.298	Weak	ETCM	*Glycyrrhizae Radix et Rhizoma*
mangiferin	4773-96-0	3	4	Undefined	-3.291	3	1	0.887	0	0.495	2	0.293	Weak	ETCM	*Anemarrhenae Rhizoma*
glycyrrhetic acid 3-O-glucuronide	34096-83-8	3	4		-5.986	2	FALSE	NA	FALSE	NA	TRUE	0.281	NA	Prediction	*Glycyrrhizae Radix et Rhizoma*
Glycyroside	125310-04-5	3	4		-2.504	3	TRUE	NA	FALSE	NA	FALSE	0.236	NA	Prediction	*Glycyrrhizae Radix et Rhizoma*
liquiritin apioside	74639-14-8	3	4		-3.037	3	TRUE	NA	FALSE	NA	FALSE	0.228	NA	Prediction	*Glycyrrhizae Radix et Rhizoma*
isoschaftoside	52012-29-0	3	4	Undefined	-4.499	2	1	0.86	0	0.277	0	0.196	Weak	ETCM	*Glycyrrhizae Radix et Rhizoma*
schaftoside	51938-32-0	3	4	Undefined	-6.145	1	1	0.98	0	0.267	2	0.183	Weak	ETCM	*Glycyrrhizae Radix et Rhizoma*
neomangiferin	64809-67-2	3	4		-6.016	1	TRUE	NA	FALSE	NA	FALSE	0.176	NA	Prediction	*Anemarrhenae Rhizoma*
timosaponin A II	117210-12-5	0	0	0.97	-7.533	1	FALSE	NA	FALSE	NA	FALSE	0.161	NA	Prediction	*Anemarrhenae Rhizoma*
anemarrhensaponin I	163047-21-0	3	4	Undefined	-4.236	2	0	0.052	0	0.346	0	0.149	Weak	ETCM	*Anemarrhenae Rhizoma*

isoliquiritin apioside	120926-46-7	3	4		-3.176	3	TRUE	NA	FALSE	NA	FALSE	0.148	NA	Prediction	*Glycyrrhizae Radix et Rhizoma*
timosaponin A IV	942621-71-8	2	0	1.66	-9.093	0	TRUE	NA	FALSE	NA	FALSE	0.139	NA	Prediction	*Anemarrhenae Rhizoma*
glycyrrhizic acid	1405-86-3	3	4	Undefined	-6.703	1	1	0.675	0	0.277	0	0.135	Weak	ETCM	*Glycyrrhizae Radix et Rhizoma*
xilingsaponin B	N/A	3	4	Undefined	-4.661	2	0	0.066	0	0.128	0	0.132	Weak	ETCM	*Anemarrhenae Rhizoma*
pseudoprototimosaponin AIII	142759-74-8	3	4		-3.933	3	FALSE	NA	FALSE	NA	FALSE	0.106	NA	Prediction	*Anemarrhenae Rhizoma*
officinalisinin I	57944-18-0	3	4		-4.728	2	FALSE	NA	FALSE	NA	FALSE	0.097	NA	Prediction	*Anemarrhenae Rhizoma*
timosaponin BII	136656-07-0	3	4	Undefined	-4.728	2	0	0.059	0	0.128	0	0.097	Weak	ETCM	*Anemarrhenae Rhizoma*
anemarsaponin E	136565-73-6	3	4	Undefined	-3.978	3	0	0.086	0	0.128	0	0.096	Weak	ETCM	*Anemarrhenae Rhizoma*
timosaponin D	N/A	3	4		-4.612	2	FALSE		FALSE		FALSE	0.096		Prediction	*Anemarrhenae Rhizoma*

Table S10 Molecular docking results by Autodock Vina

Proteins	Herbs	Bioactive compounds	Binding Affinity (kcal/mol)
AKT1	*Glycyrrhizae Radix et Rhizoma*	Citric acid	-4.3
NFKB1	*Cinnamomi Ramulus*	Cinnamic acid	-4.5
NFKB1	*Glycyrrhizae Radix et Rhizoma*	Citric acid	-4.6
TLR4	*Glycyrrhizae Radix et Rhizoma*	Citric acid	-4.7
AKT1	*Cinnamomi Ramulus*	Cinnamic acid	-4.8
TLR4	*Cinnamomi Ramulus*	Cinnamic acid	-5.8
NFKB1	*Glycyrrhizae Radix et Rhizoma*	Liquiritigenin	-5.8
NFKB1	*Anemarrhenae Rhizoma*	Mangiferin	-5.8
AKT1	*Glycyrrhizae Radix et Rhizoma*	Formononetin	-5.9
AKT1	*Glycyrrhizae Radix et Rhizoma*	Isoliquiritigenin	-5.9
NFKB1	*Glycyrrhizae Radix et Rhizoma*	Isoliquiritigenin	-6.0
AKT1	*Glycyrrhizae Radix et Rhizoma*	Liquiritigenin	-6.1
NFKB1	*Glycyrrhizae Radix et Rhizoma*	Formononetin	-6.3
NFKB1	*Anemarrhenae Rhizoma*	Vitexin	-6.5
TLR4	*Glycyrrhizae Radix et Rhizoma*	Formononetin	-6.6
AKT1	*Anemarrhenae Rhizoma*	Mangiferin	-6.6
AKT1	*Glycyrrhizae Radix et Rhizoma*	Schaftoside	-6.7
TLR4	*Glycyrrhizae Radix et Rhizoma*	Liquiritigenin	-6.8
TLR4	*Glycyrrhizae Radix et Rhizoma*	Isoliquiritigenin	-6.9
TLR4	*Anemarrhenae Rhizoma*	Mangiferin	-6.9
NFKB1	*Glycyrrhizae Radix et Rhizoma*	Schaftoside	-6.9
AKT1	*Anemarrhenae Rhizoma*	Vitexin	-6.9
TLR4	*Glycyrrhizae Radix et Rhizoma*	Schaftoside	-7.0
TLR4	*Glycyrrhizae Radix et Rhizoma*	Glycyrrhizic acid	-7.2

TLR4	*Anemarrhenae Rhizoma*	Vitexin	-7.2
AKT1	*Glycyrrhizae Radix et Rhizoma*	Glycyrrhizic acid	-7.5
NFKB1	*Glycyrrhizae Radix et Rhizoma*	Glycyrrhizic acid	-7.7
NLRP3	*Cinnamomi Ramulus*	Cinnamic acid	-6.4
NLRP3	*Anemarrhenae Rhizoma*	Mangiferin	-8.5
NLRP3	*Glycyrrhizae Radix et Rhizoma*	Glycyrrhizic acid	-13.9
NFκB_p65	*Cinnamomi Ramulus*	Cinnamic acid	-5.3
NFκB_p65	*Anemarrhenae Rhizoma*	Mangiferin	-7.7
NFκB_p65	*Glycyrrhizae Radix et Rhizoma*	Glycyrrhizic acid	-12.2

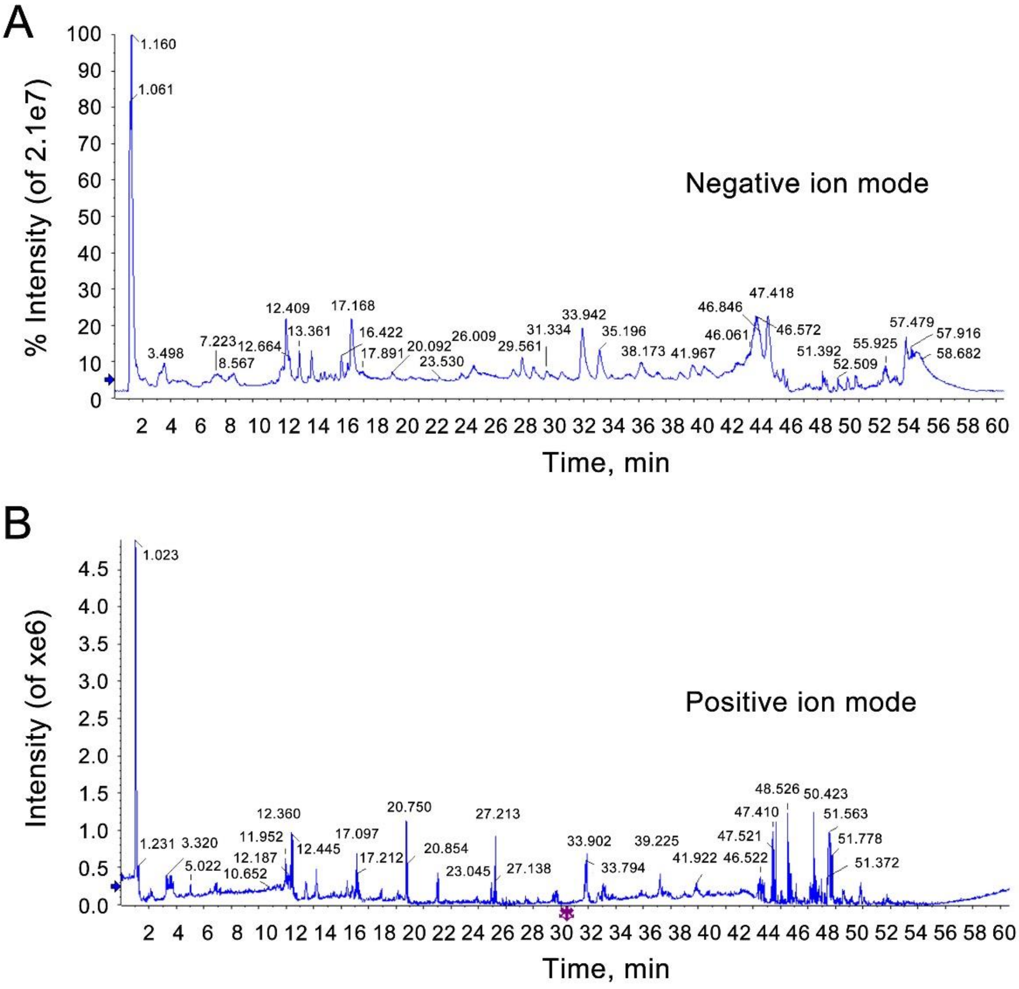

Figure S1 Total ion chromatogram for sera in normal control group after the treatment of Baihu-Guizhi decoction (BHGZD) by UHPLC-QTRAP-MS/MS system. (**A**) Negative ion mode. (**B**) Positive ion mode.

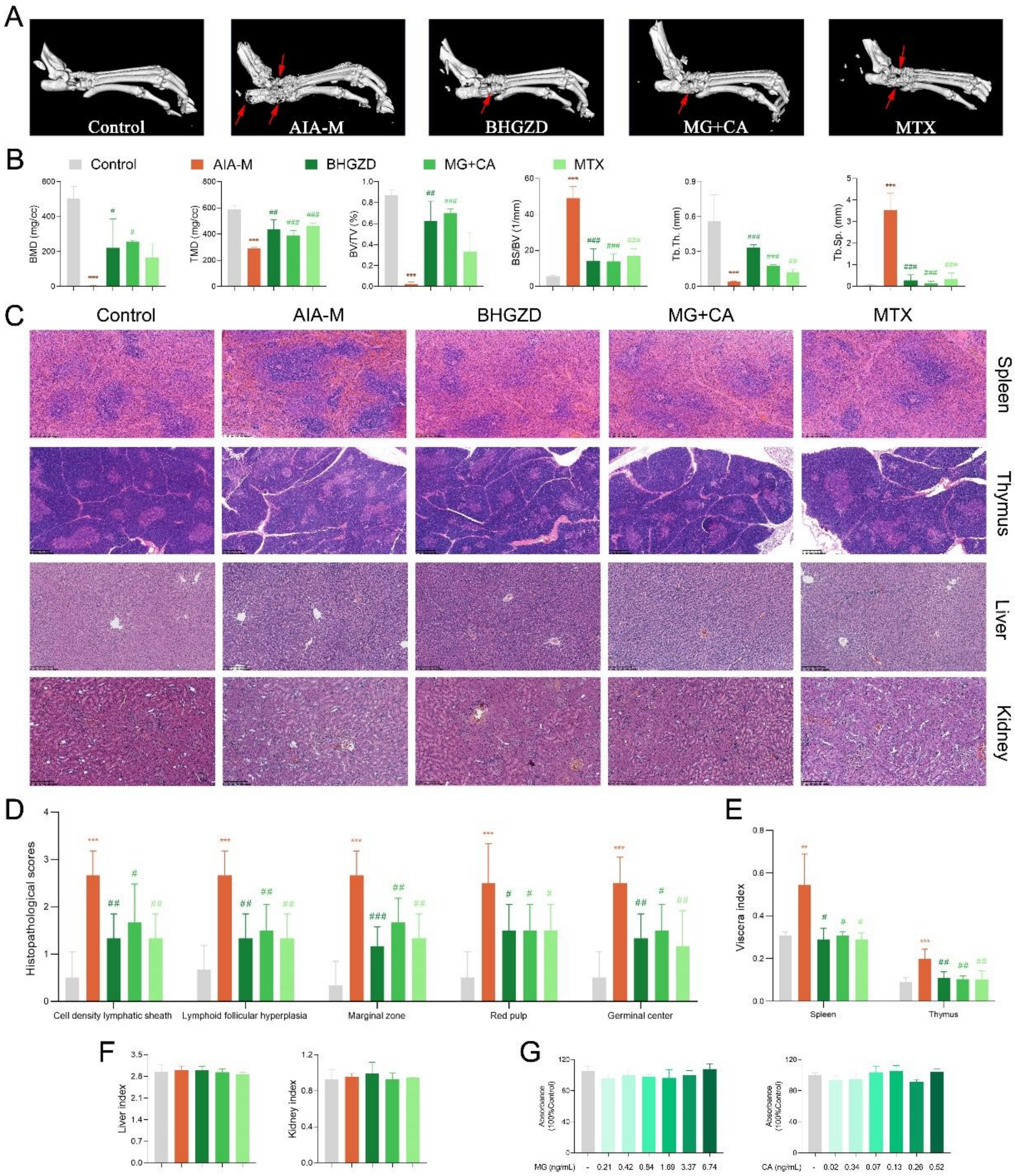

A
Control
AIA-M
BHGZD
MG+CA
MTX
B
Control
AIA-M
BHGZD
MG+CA
MTX
BMD (mg/cc)
TMD (mg/cc)
BV/TV (%)
BS/BV (1/mm)
Tb.Th. (mm)
Tb.Sp. (mm)
C
Spleen
Thymus
Liver
Kidney
D
Histopathological scores
Cell density lymphatic sheath
Lymphoid follicular hyperplasia
Marginal zone
Red pulp
Germinal center
E
Viscera index
Spleen
Thymus
F
Liver index
Kidney index
G
Absorbance (100%Control)
MG (ng/mL)
- 0.21 0.42 0.84 1.69 3.37 6.74
CA (ng/mL)
- 0.02 0.34 0.07 0.13 0.26 0.52

Figure S2 Micro-computed tomography (micro-CT) analysis and pathological changes in the liver, kidney, spleen, and thymus of different groups using hematoxylin and eosin (H&E) staining based on AIA-M rats. And the cell viability on Raw264.7 cells with the treatment of MG and CA tested using cell counting kit-8 (CCK8) assay. (**A**) Representative micro-CT images of ankle joints showing bone erosion levels in different groups (The red arrow indicates the position of the bone destruction). (**B**) Quantitative micro-CT analysis of bone mineral density (BMD), tissue mineral density (TMD), bone volume/tissue volume (BV/TV), bone surface density (BS/BV), trabecular separation (Tb.Sp), trabecular thickness (Tb.Th). (**C**) Pathological changes in the liver, kidney, spleen and thymus of AIA-M rats using H&E. (**D**) Histopathological scores in cell density lymphatic sheath, lymphoid follicular hyperplasia, marginal zone, red pulp, and germinal center. (**E**) Spleen and thymus indexes in AIA-M rats. (**F**) Liver and kidney indexes in AIA-M rats. (**G**) The cell viabilities on Raw264.7 cells with the treatment of MG/CA were test using CCK8 assay. Data are expressed as the mean±S.D. **, ***, $p<0.01$, $p<0.001$, respectively, comparison with normal control group; #, ##, ###, $p<0.05$, $p<0.01$, $p<0.001$, respectively, comparison with AIA-M model group.

References:

1. Li W, Mao X, Wang X, Liu Y, Wang K, Li C, et al. Disease-Modifying Anti-Rheumatic Drug Prescription Baihu-Guizhi Decoction Attenuates Rheumatoid Arthritis Via Suppressing Toll-Like Receptor 4-Mediated Nlrp3 Inflammasome Activation. *Frontiers in pharmacology* (2021) 12:743086. Epub 2021/10/23. doi: 10.3389/fphar.2021.743086.
2. Li W, Mao X, Wu H, Guo M, Su X, Lu J, et al. Deciphering the Chemical Profile and Pharmacological Mechanisms of Baihu-Guizhi Decoction Using Ultra-Fast Liquid Chromatography-Quadrupole-Time-of-Flight Tandem Mass Spectrometry Coupled with Network Pharmacology-Based Investigation. *Phytomedicine : international journal of phytotherapy and phytopharmacology* (2020) 67:153156. Epub 2020/01/07. doi: 10.1016/j.phymed.2019.153156.
3. Mao X, Li W, Chen W, Li Y, Wang Q, Wang X, et al. Exploring and Characterizing a Novel Combination of Paeoniflorin and Talatizidine for the Treatment of Rheumatoid Arthritis. *Pharmacol Res* (2020) 153:104658. Epub 2020/02/01. doi: 10.1016/j.phrs.2020.104658.
4. Dixon WJ. Efficient Analysis of Experimental Observations. *Annu Rev Pharmacol Toxicol* (1980) 20:441-62. Epub 1980/01/01. doi: 10.1146/annurev.pa.20.040180.002301.
5. Wang C, Kong X, Zhu C, Liu C, Sun D, Xu Q, et al. Wu-Tou Decoction Attenuates Neuropathic Pain Via Suppressing Spinal Astrocytic Il-1r1/Traf6/Jnk Signaling. *Oncotarget* (2017) 8(54):92864-79. Epub 2017/12/02. doi: 10.18632/oncotarget.21638.
6. Yoon C, Wook YY, Sik NH, Ho KS, Mo CJ. Behavioral Signs of Ongoing Pain and Cold Allodynia in a Rat Model of Neuropathic Pain. *Pain* (1994)

59(3):369-76. Epub 1994/12/01. doi: 10.1016/0304-3959(94)90023-X.

7. Hargreaves K, Dubner R, Brown F, Flores C, Joris J. A New and Sensitive Method for Measuring Thermal Nociception in Cutaneous Hyperalgesia. *Pain* (1988) 32(1):77-88. Epub 1988/01/01. doi: 10.1016/0304-3959(88)90026-7.

8. Wang Y, Liu Z, Li C, Li D, Ouyang Y, Yu J, et al. Drug Target Prediction Based on the Herbs Components: The Study on the Multitargets Pharmacological Mechanism of Qishenkeli Acting on the Coronary Heart Disease. *Evid Based Complement Alternat Med* (2012) 2012:698531. Epub 2012/04/05. doi: 10.1155/2012/698531.

9. He Y, Su W, Chen T, Zeng X, Yan Z, Guo J, et al. Identification of Prototype Compounds and Derived Metabolites of Naoxintong Capsule in Beagle Dog Urine and Feces by Uflc-Q-Tof-Ms/Ms. *Journal of pharmaceutical and biomedical analysis* (2019) 176:112806. Epub 2019/08/20. doi: 10.1016/j.jpba.2019.112806.

10. Huang CF, Chen L, Li YC, Wu L, Yu GT, Zhang WF, et al. Nlrp3 Inflammasome Activation Promotes Inflammation-Induced Carcinogenesis in Head and Neck Squamous Cell Carcinoma. *J Exp Clin Cancer Res* (2017) 36(1):116. Epub 2017/09/04. doi: 10.1186/s13046-017-0589-y.

11. Zhao LR, Xing RL, Wang PM, Zhang NS, Yin SJ, Li XC, et al. Nlrp1 and Nlrp3 Inflammasomes Mediate Lps/Atp-Induced Pyroptosis in Knee Osteoarthritis. *Molecular Medicine Reports* (2018) 17(4):5463-9. doi: 10.3892/mmr.2018.8520.
